# Identification of relevant differential genes to the divergent development of pectoral muscle in ducks by transcriptomic analysis

**DOI:** 10.5713/ab.23.0505

**Published:** 2024-04-01

**Authors:** Fan Li, Zongliang He, Yinglin Lu, Jing Zhou, Heng Cao, Xingyu Zhang, Hongjie Ji, Kunpeng Lv, Debing Yu, Minli Yu

**Affiliations:** 1 Department of Animal Genetics, Breeding and Reproduction, College of Animal Science and Technology, Nanjing Agricultural University, Nanjing, Jiangsu 210095, China; 2 Nanjing Institute of Animal Husbandry and Poultry Science, Nanjing, Jiangsu 210036, China

**Keywords:** Ducks, Gene Expression, Myofiber Characteristics, Skeletal Muscles, Transcriptomics

## Abstract

**Objective:**

The objective of this study was to identify candidate genes that play important roles in skeletal muscle development in ducks.

**Methods:**

In this study, we investigated the transcriptional sequencing of embryonic pectoral muscles from two specialized lines: Liancheng white ducks (female) and Cherry valley ducks (male) hybrid Line A (LCA) and Line C (LCC) ducks. In addition, prediction of target genes for the differentially expressed mRNAs was conducted and the enriched gene ontology (GO) terms and Kyoto encyclopedia of genes and genomes signaling pathways were further analyzed. Finally, a protein-to-protein interaction network was analyzed by using the target genes to gain insights into their potential functional association.

**Results:**

A total of 1,428 differentially expressed genes (DEGs) with 762 being up-regulated genes and 666 being down-regulated genes in pectoral muscle of LCA and LCC ducks identified by RNA-seq (p<0.05). Meanwhile, 23 GO terms in the down-regulated genes and 75 GO terms in up-regulated genes were significantly enriched (p<0.05). Furthermore, the top 5 most enriched pathways were ECM-receptor interaction, fatty acid degradation, pyruvate degradation, PPAR signaling pathway, and glycolysis/gluconeogenesis. Finally, the candidate genes including integrin b3 (*Itgb3*), pyruvate kinase M1/2 (*Pkm*), insulin-like growth factor 1 (*Igf1*), glucose-6-phosphate isomerase (*Gpi*), GABA type A receptor-associated protein-like 1 (*Gabarapl1*), and thyroid hormone receptor beta (*Thrb*) showed the most expression difference, and then were selected to verification by quantitative real-time polymerase chain reaction (qRT-PCR). The result of qRT-PCR was consistent with that of transcriptome sequencing.

**Conclusion:**

This study provided information of molecular mechanisms underlying the developmental differences in skeletal muscles between specialized duck lines.

## INTRODUCTION

The quality of meat is influenced by the development of skeletal muscles during the embryonic stage. The interplay between myoblast proliferation and differentiation, as well as the establishment of functional motor units, contributes to skeletal muscle development [1]. Embryonic and postnatal muscle development is influenced by the number, cross-sectional area, and length of myofibers [2]. During the embryonic stage, myoblasts proliferate, fuse, and finally form into muscle fibers. In this process, the morphological structure and quantity of myofibers are completely determined, affecting postnatal muscle growth [3,4]. Abnormal muscle fiber arrangement, insufficient size, or changes in fiber type negatively impact meat tenderness, juiciness, flavor, and palatability [5]. The development of skeletal muscles is influenced by various genetic and environmental factors, which ultimately determine the composition and texture of the meat. Enhancing our understanding of these processes will enable targeted interventions for improved meat characteristics.

Liancheng white ducks (LW), a rare local waterfowl breed in China known for its delicious flavor and succulent flesh, were limited due to slow growth and significantly high production cost [6]. Cherry valley ducks (CV) are a fast-growing breed of large meat duck with disadvantages such as thick subcutaneous fat and poor meat quality [7]. LW (♂)×CV (♀) F6 hybrid population was selected into two different directions: Line A (LCA) and Line C (LCC). The selection of the LCA was mainly based on growth rate, while in LCC the selection focused on meat quality and flavor. The aim of this study was trying to identify the relevant differential genes to the divergent development of pectoral muscle in LCA and LCC ducks by transcriptomic analysis. Previous studies investigated the meat quality differences among five different chicken breeds and found that the commercial broiler line (Lingnanhuang) exhibited superior meat tenderness, as indicated by lower shear force values, compared to the other four breeds [8]. Additionally, there are differences in the biochemical characteristics of the muscle tissues of turkeys and broilers that lead to differences in meat quality [9]. These findings highlight the significance of accounting for breed variations in the quality of poultry meat to achieve effective production and consumer contentment.

Embryonic skeletal muscle development is precisely regu lated by a series of genes and signal pathways expressed [10]. Transcriptome sequencing has developed rapidly in recent years and is a technique for analyzing the transcriptome using deep sequencing technology [11]. In recent years, RNA-seq has been widely used in research on livestock and poultry transcriptomes [12]. Previous studies reported the transcriptome expression of pectoral and leg muscle of poultry at different growth stages [13,14]. The differentially expressed genes (DEGs) such as *Myl4*, *Igf2bp1*, and the regulatory pathways such as focal adhesion and ECM-receptor interaction play crucial roles in muscle development in ducks [15]. RNA-Seq is a useful tool to measure gene transcription and better understand the physiology behind specific phenotypes [16].

Phenotypic differences in pectoral muscles were observed between LCA and LCC. To investigate these variations, a precise and reliable digital gene expression technology was utilized to obtain a plentiful sequence of transcript-levels. This will allow for the exploration of the genetic background and key regulating genes that play important roles in embryonic pectoral muscle development within lines that exhibit differences in meat production.

## MATERIALS AND METHODS

### Experimental animals

All procedures were implemented according to the Local Experimental Animal Care Committee and approved by the ethics committee of Nanjing Agricultural University (NO. SYXK (SU) 2021-0085).

### Sample collection

Before the incubation, all the eggs and the incubator were fumigated with 42 mL/m^3^ of formalin. The eggs were then kept at 18°C for a day. All eggs were incubated for 24 days at 37.8°C and 60% relative humidity in a fully automated egg incubator (JT35, Jitan, China). Next, from the 25th to the 28th day, all eggs were transferred to the hatcher tray in the same incubator, which had a temperature of 36.9°C and 70% relative humidity [3]. On embryonic day 28 (E28), six male ducks of each specialized line were randomly chosen. The pectoral muscles were collected and frozen at −80°C for subsequent section preparation and total RNA extraction.

### Histological determination of muscle fibers

Paraffin sections of the collected pectoral muscle samples were made and stained with hematoxylin and eosin (HE). Five fields were randomly selected from each section for image acquisition, and the myofiber density (MFN), myofiber diameter (MFD), and myofiber cross-sectional area (CSA) was measured by using Image-Pro Plus 6.0 software.

### Sequencing data processing

The clustering of the index-coded samples was performed on a cBot Cluster Generation System using TruSeq PE Cluster Kit v3-cBot-HS (Illumia, San Diego, CA, USA) according to the manufacturer’s instructions. After cluster generation, the library preparations were sequenced on an Illumina Novaseq platform (novogene.com). Reference genome and gene model annotation files were downloaded from genome website directly. Index of the reference genome was built using HISAT2 (daehwankimlab.github.io) and paired-end clean reads were aligned to the reference genome. Based on the selected reference genome sequence, the mapped reads were spliced by StringTie (jhu.edu) to find the original unannotated transcripts and new genes of the species to supply and improve the original genome annotation information.

### Analysis and enrichment of differentially expressed genes

The clean-mapped reads were utilized to analyze gene expression through DEseq2, which is available online (https://github.com/mikelove/DESeq2). Then, log2 Fold Change ≥0 and p-values <0.05 were defined to obtain DEGs. The DEGs set was analyzed for gene ontology (GO) functional enrichment and Kyoto encyclopedia of genes and genomes (KEGG) pathway enrichment using cluster Profiler (3.8.1) software. Using DAVID analysis (https://david.abcc.ncifcrf.gov/), GO functional categorization and enrichment studies were carried out to find phrases that were significantly enriched in DEGs. STRING (https://string-db.org) was used to conduct the gene inter-action network prediction study and visualized using Cytoscape 3.1.0 (https://www.cytoscape.org/).

### RNA extraction and quantitative real time polymerase chain reaction

Total RNA was extracted by Trizol reagent, and the quality and concentration of RNA samples were detected by UV spectrophotometer, respectively. Relative miRNA expression levels were determined through quantitative real time polymerase chain reaction (qRT-PCR) analysis, utilizing the 2×T5 Fast qPCR Mix (TsingKe, Beijing, China), following the manufacturer’s instructions. The qRT-PCR program was conducted on an Applied Biosystems (ABI, Foster City, CA, USA) Quant Studio 5 system (Thermo Fisher, Waltham, MA, USA) according to the protocol: 95°C for 10 min; 40 cycles of 95°C for 2 s, 60°C for 1 min; and collection of fluorescence at 65°C to 95°C. Every sample was performed in triplicate. Primers were designed using Primer-BLAST according to the gene sequences published in GenBank in NCBI, and the designed primers were synthesized by Shanghai Biotechnology Company (Shanghai, China). The sequence of primers is shown in Table 1.

### Data processing and statistical analysis

Significant discrepancies in the histological analysis of muscle fibers between different specialized lines were assessed via independent sample T-tests using SPSS 26.0. The results were plotted using GraphPad Prism 8.0 software, “*” was considered statistically difference (p<0.05); “**” was considered statistically significant (p<0.01); “***” was considered highly significant difference (p<0.001). The relative expression level of the pertinent genes were calculated using the 2^−ΔΔCT^ statistical analysis method.

## RESULTS

### Comparison of morphological characteristics of muscle fibers

**Figure 1:**
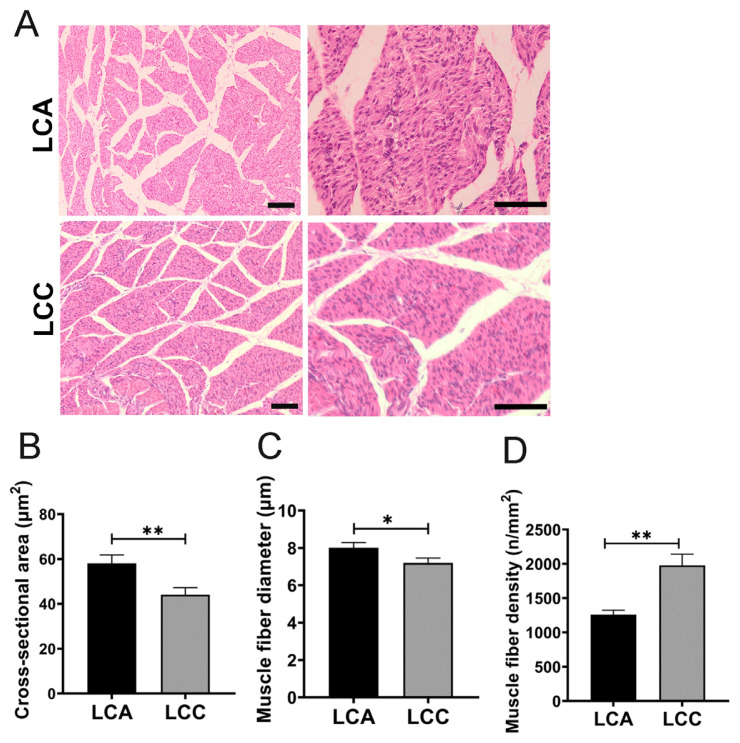
The morphology difference of pectoral muscles in Liancheng white ducks (female) and Cherry valley ducks (male) hybrid Line A (LCA) and Line C (LCC) ducks. (A) The morphology of pectoral muscles in E28 LCA and LCC was measured by hematoxylin and eosin (HE) staining. (Scale bar = 100 μm). (B) Analysis of cross-sectional area, (C) myofiber diameter, and (D) density of myofiber were conducted. Vertical bars represent mean±standard error of the mean (n = 6). * p<0.05, ** p<0.01, *** p<0.001.

To compare the pectoral muscle fiber traits, the characteristics of muscle fibers in E28 LCA and LCC were analyzed by HE staining. Results showed that LCA had significantly larger area and diameter of myofiber than those of LCA (p<0.01) (Figure 1B, 1C). In contrast, myofiber density in LCC was significantly higher than that of LCA (p<0.01) (Figure 1D). This result indicated that LCA had significantly more intense embryonic pectoral muscle development than LCC.

### Transcriptome sequencing data and quality control

The transcriptomic libraries of three pectoral muscle samples from each of the group were constructed, resulting in a total of 249,902,400 high-quality data. As shown in Table 2, the clean reads of each sample after filtration were more than 5.86 Gb, resulting in a total of 37.47 G of clean data being obtained: with Q30 ≥92.38% and GC content ranging from 47.87% to 50.98%. The results indicate that the sequencing data are of reliable quality and can be used for further analysis.

### Screening and clustering analysis of differentially expressed genes

**Figure 2:**
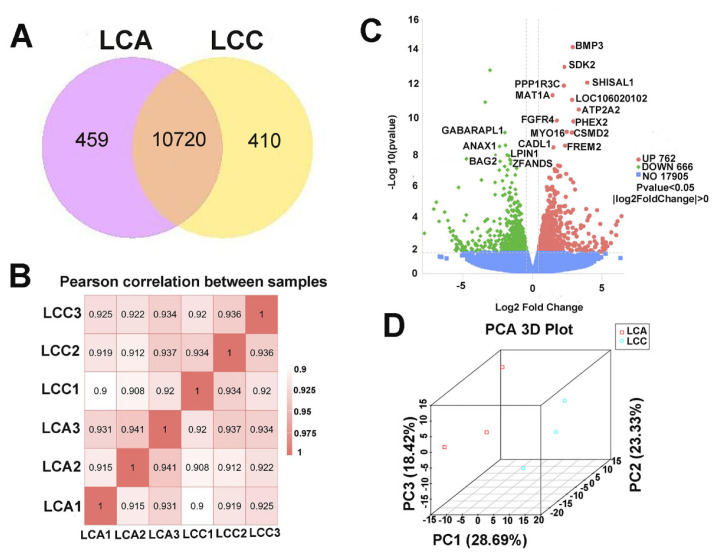
Differentially expressed mRNAs in pectoral muscles between Liancheng white ducks (female) and Cherry valley ducks (male) hybrid Line A (LCA) and Line C (LCC) ducks. (A) Venn diagrams of differentially expressed mRNAs in pectoral muscles of LCC vs LCA. (B) Ideal sampling and experimental conditions with squared Pearson’s correlation coefficient (R^2^) greater than 0.8 were the criterion and R^2^ was greater than 0.9 between groups. (C) Volcano chart of mRNA expressed at LCC vs LCA. Each dot in the volcano plot represents a gene. Green dot: significantly down-regulated genes; Red dot: significantly up-regulated genes; Blue dot: genes without differential ex-pression. (D) Principal component analysis (PCA) of the gene expression data (the principal components 1, 2, and 3 accounted for 28.69, 23.33, and 18.42 of variance).

A number of genes were filtered for FPKM≥1. There were 10,720 in LCA compared to LCC common DEGs and 459, and 410 distinct DEGs between LCA and LCC at E28 (Figure 2A). When the correlation coefficients of gene expression were analyzed for each pair of biological replicates of the same condition (Figure 2B), the values of the three different replicates within the LCA group and the three different replicates within the LCC group were all greater than 0.9, indicating that the reliability and reproducibility of the experiment were both high. The DEGs between the two duck lines were screened using DESeq2 software, with log2 Fold Change ≥0 and p<0.05 as the screening criteria. A total of 1,428 DEGs were detected, of which 762 were up-regulated genes and 666 were down-regulated genes (Figure 2C). Volcano plots visualized the differences in gene expression levels and statistical significance between the two groups of pectoral muscle samples from LCA and LCC. The genes with differential expression significance in the top 20 positions are listed in Table 3. More importantly, the principal component analysis revealed a divergent expression pattern between LCA and LCC (Figure 2D).

### Gene ontology analysis of differentially expressed genes

To investigate the differential expression patterns of DEGs between LCA and LCC, all DEGs were enriched and analyzed by the GO database. Cellular component (CC), molecular function (MF), and biological process (BP) are the three basic divisions of the GO annotation system. There were 700 enriched GO terms in down-regulated and up-regulated genes (Supplementary Table S1). However, 23 GO terms in the down-regulated and 75 GO terms in up-regulated genes were significantly enriched (p<0.05) (Supplementary Table S2, Table S3). The DEGs were mainly enriched in early development related BP and pathways, followed by CC and finally MF. Filtered by keywords “Development”, “Growth” and analyzed in relation to the following pathways. The most significantly enriched terms are displayed in Table 4. The BP category was primarily mainly in insulin-like growth factor binding, Wnt signaling pathway, growth factor binding, animal organ development, protein phosphorylation, oxidation-reduction process, and carbohydrate metabolic process.

### Kyoto encyclopedia of genes and genomes annotation of differentially expressed genes and network analysis

**Figure 3:**
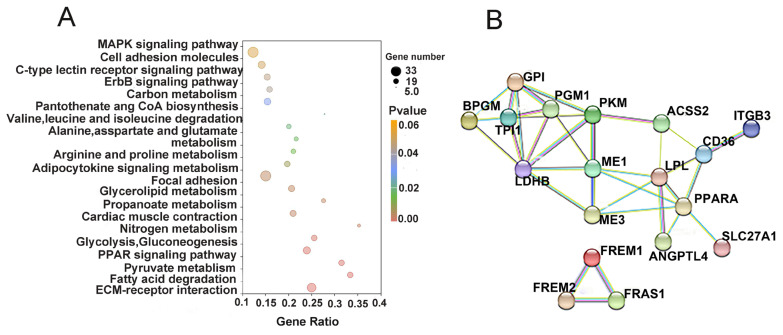
Enrichment analysis of the differentially expressed mRNAs. (A) The top 20 enriched Kyoto encyclopedia of genes and genomes signaling pathways of the target genes of differentially expressed mRNA. (B) The size of a node in an inter working network graph is proportional to the degree of this node, the more edges are connected to this node, the larger its degree, and the larg-er the node, which may be in a more central position in the network.

KEGG enrichment analysis was performed used to identify significantly enriched entries in DEGs, the differential genes compared between the two groups were significantly enriched in 19 KEGG terms (p<0.05) (Supplementary Table S4). The most significantly up-regulated and down-regulated KEGG-enriched terms are shown in (Figure 3A, Table 5). The top 5 most enriched pathways were ECM-receptor interaction (22 genes), fatty acid degradation (11 genes), pyruvate degradation (11 genes), PPAR signaling pathway (16 genes), and glycolysis/gluconeogenesis (12 genes) (Figure 3A, Table 5). Functional enrichment analysis of DEGs showed two signaling pathways —pyruvate degradation and glycolysis/gluconeogenesis—to be related to energy metabolism. This suggests that energy metabolism activities in duck pectoral muscles are more intense during the later stages of embryonic development.

Maps of protein interaction network analysis were created to better comprehend the relationships between diverse genes. Twenty differential genes were chosen from the differential genes in the LCA and LCC’s pectoral muscles, and a total of eighteen known encoded proteins were discovered, participating in a total of two sets of protein interactions (Figure 3B). The analysis revealed that *Pkm*, *Gpi*, and malic enzyme 1 (*Me1*) were central hubs within the interaction network.

### Validation of differentially expressed genes by quantitative real time polymerase chain reaction

**Figure 4:**
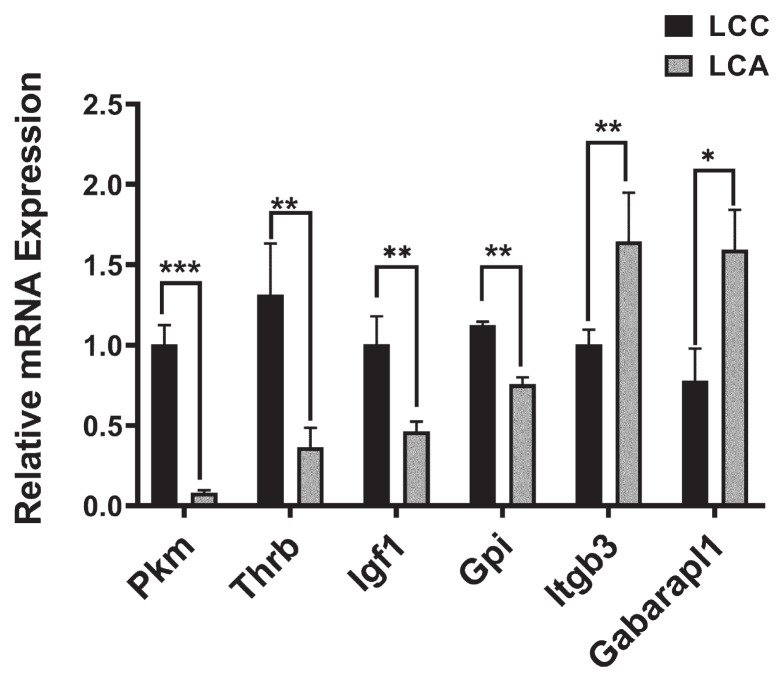
Validation of candidate genes by quantitative real time polymerase chain reaction (qRT-PCR). The mRNA expression of *Pkm*, *Gpi*, *Thrb*, *Igf1*, *Itgb3*, and *Gabarapl1* in Liancheng white ducks (female) and Cherry valley ducks (male) hybrid Line A (LCA) and Line C (LCC) ducks was tested by qRT-PCR. Data are presented as the means± standard error of the mean. * p<0.05, ** p<0.01, *** p<0.001.

The expression of 6 selected DEGs, including 2 down-regulated genes (*Itgb3* and *Gabarapl1*) and 4 up-regulated genes (*Pkm*, *Thrb*, *Igf1*, and *Gpi*), was verified through qRT-PCR. The result suggested that the mRNA expression of down-regulated gene *Gabarapl1* was lower in LCC than that of LCA (p<0.05), and *Itgb3* was significantly lower in LCC than that of LCA (p<0.01) (Figure 4). Moreover, the mRNA expression of up-regulated genes (*Pkm*, *Thrb*, *Igf1*, and *Gpi*) showed remarkably higher in LCC than that of LCA (p<0.01) (Figure 4). These results were consistent with sequencing data. The expression trend of these six genes corresponded with the findings from the RNA-seq experiment, providing evidence for the reliability of the RNA-seq results.

## DISCUSSION

The characteristics of muscle fiber are commonly used as essential parameters to evaluate meat quality during growth and development [2,17]. During embryonic development, myoblasts undergo proliferation, fusion, and ultimately differentiation into muscle fibers. This process fully establishes the structure and quantity of myofibers, hence directly influencing postnatal muscle growth and development [3,18–20]. In the present study, LCC had significantly higher myofiber density at E28, which suggested that LCC pectoral muscles contain more myofibers than LCA. It is commonly accepted that a decrease in the amount of muscle fibers is linked to hypertrophic fibers, which are inclined to have inferior meat quality when compared to muscles with a smaller fiber CSA [21]. The present study found that LCA had more robust embryonic pectoral muscle development than LCC.

Our study brings attention to the noteworthy disparities in DEGs between two lines, with a particular focus on the E28 stage. This observation indicates that these DEGs may play a crucial role in the striking variations between the two strains. Our aim was to investigate the gene regulatory mechanisms associated with the development of embryonic pectoral muscles by performing GO and KEGG pathway analyses on the identified DEGs. The growth of ducks is regulated by multiple genes, which are involved in a variety of BPs and distinct regulatory pathways [22]. The KEGG pathway enrichment results showed that the first several pathways that were the most reliable were linked to muscle development and fat deposition. The glycolysis/gluconeogenesis and pyruvate metabolism pathways have established functions in embryonic muscle regulation, whereas the ECM receptor interaction, fatty acid degradation and PPAR signaling pathways have critical roles in maintaining muscle mass [23–25]. The differential expression of collagens of ECM was found in different duck lines in this study, indicating the different composition of collagens in different types of muscles. The signaling pathway of ECM-receptor interaction, which is thought to influence growth, lipid production and metabolism of intramuscular adipocytes, intramuscular fat content and ultimately meat flavor in ducks, includes numerous genes associated with meat quality [23,24]. The specific roles of the genes involved in these pathways will require further analysis in future studies.

The ECM receptor interaction pathway contains more of the genes *Thbs2* and *Itgb3* [25]. It has been demonstrated that various pig breeds express the gene differently during embryonic development. The *Itgb3* gene is involved in myogenic differentiation [25,26]. To gain further insights into the interactions among the genes, a genes interaction network was constructed. Three muscle structural protein-coding genes (*Itgb3*, *Gpi*, and *Pkm*) were identified. *Pkm2* is involved in the energy metabolism of the nucleus and cytoplasm [27], leading to the production of adenosine triphosphate and adenosine monophosphate, which in turn leads to the synthesis of inosincacid, inosinemonphosphate [28–31]. *Gpi* are lipid-anchored proteins expressed by eukaryotes outside the plasma membrane [32]. It has a wide range of functions which are involved in important life processes such as cell recognition, growth, programmed cell death and the transfer of transmembrane information [33]. Insulin like growth factors (IGFs) are synthesized in tissues and play an important role in avian embryonic development via autocrine/paracrine mechanisms [34,35]. Higher levels of *Igf1* expression were found in muscle from E15–E18 with a peak at E17 followed by a decline during embryonic development [36]. *Gabarapl1* is associated with glycolytic potential and is controlled by hormones like adrenaline, glucagon, and insulin. It is also closely related to some characteristics of meat quality [37,38]. Further investigation is required to determine whether these functional genes interact with muscle structural protein coding genes in the network to influence the differences in embryonic pectoral muscle development between LCA and LCC.

## CONCLUSION

In conclusion, results showed that there were significant differences in muscle fiber characteristics between E28 LCA and LCC. LCA had more robust pectoral muscle development than that of LCC in the embryonic stage. The transcriptomic study of pectoral muscles showed that a total of 1,428 DEGs were identified. The pathway analysis involved in ECM-receptor interaction, fatty acid degradation, pyruvate degradation, PPAR signaling pathway, and glycolysis/gluconeogenesis, were highly related to muscle development energy metabolism. Additionally, the interaction network of DEGs using STRING highlighted *Pkm*, *Gpi*, and *Me1* as central nodes. Functional analyses of the genes identified several key genes, including *Itgb3*, *Pkm*, *Igf1*, *Gpi*, *Gabarapl1*, and *Thrb*. Further annotation and function of the key genes still need to be well studied.

## Figures and Tables

**Table 1:** The primer sequences for quantitative real time polymerase chain reaction^1)^

Gene	Accession No.	Primer sequences (5′-3′)	Product size (bp)
*Gabarapl1*	XM_005012678.5	F: GGCCAGAGGATGCACTGTTCTTC	143
		R: CCAGTTGCCATAGACGCTCTCATC	
*Gpi*	XM_027466953.2	F: GGTGGTCGCTATTCTCTGTGGTC	123
		R: TGGAGCCGTATGGAAGTGGTTATC	
*Igf1*	XM_038187451.1	F: CCTTAACCAGTTCTGTTGCTGCTG	91
		R: AAGCCTCTGTCTCCACATACGAAC	
*Itgb3*	XM_038168829.1	F: ATTATTGAGTGCTCTCGGTTGC	100
		R: TGTCTTGGGTTTGAAGTGTTGA	
*Pkm*	XM_038184779.1	F: GCCAACCATTGCGAGGAACAC	131
		R: CGTGGGTGCCGTGAGAGAAG	
*Thrb*	XM_038174585.1	F: ATAGACTGGCAAACTGGGGC	152
		R: CGAAATCGAACCTTGCGGTC	
*β-actin*	NM_001310421.1	F: ATTGTCCACCGCAAATGCTTC	115
		R: AAATAAAGCCATGCCAATCTCGTC	

1) All the primers are designed with Primer 5.0 software and synthesized by Nanjing Qingke bioengineering company.

F, forward primer. R, reverse primer.

**Table 2:** Sequencing and mapped data statistics

Sample	Clean_reads	Clean_bases	Q20	Q30	GC_pct
E28A1	39054978	5.86G	97.07	92.38	49.27
E28A2	39819776	5.97G	97.29	92.75	48.74
E28A3	42412756	6.36G	97.31	92.83	48.45
E28C1	43566338	6.53G	97.44	93.1	47.87
E28C2	43697776	6.55G	97.17	92.55	48.04
E28C3	41350776	6.2G	97.09	92.43	50.98

Clean reads: the number of reads after filtering the original data; Clean bases: the number of bases after filtering the original data; Q20: the percentage of bases with a Phred value greater than 20 to the total bases; Q30: the percentage of bases with a Phred value greater than 30 to the total bases; GC_pct: the percentage of G and C in the four bases in clean reads.

**Table 3:** Top 20 differentially expressed genes list between Liancheng white ducks (female) and Cherry valley ducks (male) hybrid Line A (LCA) and Line C (LCC) ducks

Code	Gene ID	Gene	log_2_ Fold Change	p-value	padj	Up/Down LCC vs LCA
1	101799577	*Shisal1*	3.92	1.02E-12	3.68E-09	Up
2	101800940	*Loc101800940*	3.31	4.92E-11	7.89E-08	Up
3	101791511	*Phex*	2.91	2.75E-10	3.60E-07	Up
4	101798302	*Bmp3*	2.88	5.83E-15	8.41E-11	Up
5	101797855	*Csmd2*	2.81	1.39E-09	1.34E-06	Up
6	101804531	*Myo16*	2.43	1.25E-09	1.34E-06	Up
7	101802858	*Frem2*	2.32	9.27E-09	7.87E-06	Up
8	101797778	*Sdk2*	2.26	1.04E-13	7.47E-10	Up
9	101793994	*Pppir3c*	2.23	1.55E-12	8.21E-05	Up
10	101798444	*Mat1a*	1.82	1.65E-07	3.53E-07	Up
11	101799171	*Fgfr4*	1.73	2.45E-10	4.46E-09	Up
12	101798008	*Gadl1*	1.48	6.21E-08	4.07E-05	Up
13	101805261	*Atp2a2*	1.42	1.04E-08	9.04E-06	Up
14	119714098	*Loc119714098*	−4.76	1.19E-08	1.51E-08	Down
15	101790249	*Anxa1*	−2.37	6.26E-12	8.33E-06	Down
16	101791448	*Gabarapl1*	−1.99	1.39E-09	1.34E-06	Down
17	101797414	*Loc101797414*	−1.96	8.70E-09	7.84E-06	Down
18	101798444	*Bag2*	−1.83	3.59E-08	2.59E-05	Down
19	101795185	*Lpin1*	−1.7	4.00E-08	2.75E-05	Down
20	101801981	*Zfand5*	−1.63	6.79E-08	4.26E-05	Down

**Table 4:** Significantly enriched terms of gene ontology pathways

ID	Description	p. Adjust	Meat quality related genes	DEGs number
Down-regulated path-ways				
GO:0005520	Insulin-like growth factor binding	0.014772065	*Ccn2, Ccn1, Igfbp33*	3
GO:0016055	Wnt signaling pathway	0.028405045	*Wnt2, Bambi, Wnt5b*	3
GO:0019838	Growth factor binding	0.014772065	*Ccn2, Ccn1, Igfbp3*	3
GO:0048513	Animal organ development	0.006156477	*Loc101800783, Myf5, Myod1*	3
GO:0006468	protein phosphorylation	0.035506697	*Cdk10, Pxk, Phkg1*	21
Up-regulated pathways				
GO:0055114	oxidation-reduction process	0.00109252	*Me1, Ptgr2, Ldhb, Hadha, Aldh1l2, Fh, Etfdh*	25
GO:0005975	Carbohydrate metabolic pro-cess	0.005668783	*Pgm2l1, Pgm1, Pkm, Mdh2, Pgm5, Gpi*	10

DEG, differentially expressed genes; GO, gene ontology.

**Table 5:** The significantly enriched terms of Kyoto encyclopedia of genes and genomes pathways

Code	Pathway name	Differentially expressed genes	

		Down-regulated	Up-regulated
1	ECM-receptor interaction	*Thbs1,Itga4,Sdc4,Itgb3,Loc101794275*	*Frem2,Fras1,Freme1,Agrm,Itga7,Lama4,Col4a5,Lamb2, Itga8,Lamb4,Cd36,Col4a6,Col6a3,Chad,Itga6,Lama5*
2	Fatty acid degradation	*Acsbg,Acsl4,Ldh7a1,Acsbg1*	*Acadm,Acaa2,Eci1,Acsi1,Cpt2*
3	Pyruvate metabolism	*Me3,Aldh7a1*	*Me1,Ldhb,Fh,Acss2,Pkm,Mdh2*
4	PPAR signaling pathway	*Lpl,Acsbg2,Slc27a1,Acsl4,Me3,Acsbg1*	*Me1,Cpt2,Acox2,Slc27a3,Cd36,Ppara,Acsl1,Scd5,Angptl4*
5	Glycoly-sis/Gluconeogenesis	*Scd,Aldh7a1,Hk2*	*Bpgm,Ldhb,Acss2,Gpi,Tpi1,Pgm1,Pkm*

## SUPPLEMENTARY MATERIAL

**Supplementary Table S1**. All gene ontology (GO) pathways enrichment

**Supplementary Table S2**. Upregulation of gene-related gene ontology (GO) pathways

**Supplementary Table S3**. Downregulation of gene-related gene ontology (GO) pathways

**Supplementary Table S4**. All Kyoto Encyclopedia of Genes and Genomes (KEGG) pathways enrichment
