# Supplementary material for: Identification of relevant differential genes to the divergent development of pectoral muscle in ducks by transcriptomic analysis
Source: Anim Biosci. 2024 Apr 1;37(8):1345–54. doi: 10.5713/ab.23.0505 (PMC11222850; doi:10.5713/ab.23.0505)
Supplement: Supplementary file 1 [file ab-23-0505-Supplementary-Table-1.pdf]

Supplemental Table 1 : All gene ontology (GO) pathways enrichment

Category	GOID	Description	GeneRatic	BgRatio	pvalue	padj	geneID	geneName	Count	Up	Up_Gene_id	Down	Down_Gene_id
BP	GO:0055114	oxidation-reduction process	39/329	281/3792	0.001686	0.575436	101790158/101793380/101803853/101794631/113839590/101790348/101801152/101799809/101803074/101789838/101789492/101794957/101802594/101797675/101799210/101798440/101801759/101805057/101805119/101789745/101793275/119713271/119714639/101798614/101804343/101789907/101793939/119717912/101792573/101804610/101792912/101804444/101801193/101791774/101791081/101797377/5405820/101804478/101801058	HIBADH/AASS/ME1/LOC101794631/LOC113839590/PTGR2/LDHB/HADHA/ALDH1L2/ETFDH/FH/EGLN1/DMGDH/D2HGDH/LOC101799210/LOC101798440/ALDH1A2/HMOX1/NDUFS1/ME3/ALDH7A1/LOC119713271/ACADM/BCO1/ACOX2/IVD/ALDH5A1/LOC119717912/LOC101792573/MDH2/LOC101792912/L2HGDH/ALDH8A1/ALDH4A1/LOC101791081/GLUD1/ND5/PRDX6/LOC101801058	39	25	101803853/101790348/101801152/101799809/101803074/101789838/101789492/101794957/101802594/101797675/101799210/101801759/101805119/119714639/101798614/101804343/101793939/119717912/101804610/101792912/101804444/101791081/5405820/101804478/101801058	14	101790158/101793380/101794631/113839590/101798440/101805057/101789745/101793275/119713271/101789907/101792573/101801193/101791774/101797377
BP	GO:0017144	drug metabolic process	9/329	39/3792	0.005089	0.575436	101799171/101789492/101790726/119714948/110351624/5405820/101797601/101800658/101790402	MAT1A/FH/PKM/LOC119714948/LOC110351624/ND5/GPI/PCBD1/ATP5PO	9	8	101799171/101789492/101790726/119714948/110351624/5405820/101797601/101790402	1	101800658
BP	GO:0044283	small molecule biosynthetic process	8/329	34/3792	0.007191	0.575436	101799450/101797455/101790726/101803472/110351624/101797078/101797601/101800658	GLUL/ASNS/PKM/ITPA/LOC110351624/IP6K2/GPI/PCBD1	8	3	101790726/110351624/101797601	5	101799450/101797455/101803472/101797078/101800658
BP	GO:0015711	organic anion transport	5/329	17/3792	0.012308	0.575436	101802251/119713349/101798013/101804872/101798131	PITPNM1/SLC52A3/MPC1/LOC101804872/PITPNC1	5	2	119713349/101798131	3	101802251/101798013/101804872
BP	GO:0032787	monocarboxylic acid metabolic process	5/329	17/3792	0.012308	0.575436	101799809/101804343/101797455/101790726/101797601	HADHA/ACOX2/ASNS/PKM/GPI	5	4	101799809/101804343/101790726/101797601	1	101797455
BP	GO:0005975	carbohydrate metabolic process	14/329	85/3792	0.01346	0.575436	101795985/101801152/101799210/101798440/101798581/101790428/113845291/101790726/101804610/113840111/101795379/101797601/101792144/101800183	LOC101795985/LDHB/LOC101799210/LOC101798440/PGM2L1/PGM1/LOC113845291/PKM/MDH2/LOC113840111/PGM5/GPI/FUCA2/HYAL2	14	10	101801152/101799210/101798581/101790428/113845291/101790726/101804610/113840111/101795379/101797601	4	101795985/101798440/101792144/101800183
BP	GO:0007267	cell-cell signaling	5/329	20/3792	0.024864	0.575436	101791711/101799824/101792754/101792356/101799859	WNT4/WNT2/BAMBI/WNT5B/RAPSN	5	1	101791711	4	101799824/101792754/101792356/101799859
BP	GO:0006164	purine nucleotide biosynthetic process	7/329	35/3792	0.027922	0.575436	101801928/101803512/101790726/101796516/119714948/101797601/101790402	ADSS1/ADCY2/PKM/LOC101796516/LOC119714948/GPI/ATP5PO	7	7	101801928/101803512/101790726/101796516/119714948/101797601/101790402	0	
BP	GO:0072522	purine-containing compound biosynthetic process	7/329	36/3792	0.032179	0.575436	101801928/101803512/101790726/101796516/119714948/101797601/101790402	ADSS1/ADCY2/PKM/LOC101796516/LOC119714948/GPI/ATP5PO	7	7	101801928/101803512/101790726/101796516/119714948/101797601/101790402	0	
BP	GO:0009141	nucleoside triphosphate metabolic process	6/329	29/3792	0.034985	0.575436	101790726/101803472/119714948/5405820/101797601/101790402	PKM/ITPA/LOC119714948/ND5/GPI/ATP5PO	6	5	101790726/119714948/5405820/101797601/101790402	1	101803472

BP	GO:0006163	purine nucleotide metabolic process	8/329	46/3792	0.041654	0.575436	101801928/101803512/101790726/101796516/119714948/5405820/101797601/101790402	ADSS1/ADCY2/PKM/LOC101796516/LOC119714948/ND5/GPI/ATP5PO	8	8	101801928/101803512/101790726/101796516/119714948/5405820/101797601/101790402	0	
BP	GO:0016053	organic acid biosynthetic process	4/329	16/3792	0.043846	0.575436	101799450/101797455/101790726/101797601	GLUL/ASNS/PKM/GPI	4	2	101790726/101797601	2	101799450/101797455
BP	GO:0046394	carboxylic acid biosynthetic process	4/329	16/3792	0.043846	0.575436	101799450/101797455/101790726/101797601	GLUL/ASNS/PKM/GPI	4	2	101790726/101797601	2	101799450/101797455
BP	GO:0072521	purine-containing compound metabolic process	8/329	47/3792	0.046571	0.575436	101801928/101803512/101790726/101796516/119714948/5405820/101797601/101790402	ADSS1/ADCY2/PKM/LOC101796516/LOC119714948/ND5/GPI/ATP5PO	8	8	101801928/101803512/101790726/101796516/119714948/5405820/101797601/101790402	0	
BP	GO:0016311	dephosphorylation	10/329	64/3792	0.046837	0.575436	101789439/101802393/101804352/101803221/101803683/101792804/101803878/101791271/101804166/101803609	LOC101789439/DUSP1/PTPRD/PTPN14/DUSP28/DUSP15/PTPRT/PTPN6/DUSP3/PTPRM	10	6	101789439/101804352/101803683/101803878/101804166/101803609	4	101802393/101803221/101792804/101791271
BP	GO:0048513	animal organ development	3/329	10/3792	0.049044	0.575436	101800783/101801755/101789522	LOC101800783/MYF5/MYOD1	3	0		3	101800783/101801755/101789522
BP	GO:1901292	nucleoside phosphate catabolic process	3/329	10/3792	0.049044	0.575436	101790726/101803472/101797601	PKM/ITPA/GPI	3	2	101790726/101797601	1	101803472
BP	GO:0006869	lipid transport	5/329	24/3792	0.051291	0.575436	101802251/101804872/101797331/101798726/101798131	PITPNM1/LOC101804872/APOA4/APOLD1/PITPNC1	5	2	101797331/101798131	3	101802251/101804872/101798726
BP	GO:0010876	lipid localization	5/329	24/3792	0.051291	0.575436	101802251/101804872/101797331/101798726/101798131	PITPNM1/LOC101804872/APOA4/APOLD1/PITPNC1	5	2	101797331/101798131	3	101802251/101804872/101798726
BP	GO:0006091	generation of precursor metabolites and energy	4/329	17/3792	0.05355	0.575436	101789492/101790726/5405820/101797601	FH/PKM/ND5/GPI	4	4	101789492/101790726/5405820/101797601	0	
BP	GO:0016055	Wnt signaling pathway	4/329	17/3792	0.05355	0.575436	101791711/101799824/101792754/101792356	WNT4/WNT2/BAMBI/WNT5B	4	1	101791711	3	101799824/101792754/101792356
BP	GO:0198738	cell-cell signaling by wnt	4/329	17/3792	0.05355	0.575436	101791711/101799824/101792754/101792356	WNT4/WNT2/BAMBI/WNT5B	4	1	101791711	3	101799824/101792754/101792356
BP	GO:1905114	cell surface receptor signaling pathway involved in cell-cell signaling	4/329	17/3792	0.05355	0.575436	101791711/101799824/101792754/101792356	WNT4/WNT2/BAMBI/WNT5B	4	1	101791711	3	101799824/101792754/101792356
BP	GO:0006470	protein dephosphorylation	6/329	32/3792	0.053671	0.575436	101789439/101804352/101803221/101803878/101791271/101803609	LOC101789439/PTPRD/PTPN14/PTPRT/PTPN6/PTPRM	6	4	101789439/101804352/101803878/101803609	2	101803221/101791271
BP	GO:0009152	purine ribonucleotide biosynthetic process	6/329	33/3792	0.061022	0.575436	101803512/101790726/101796516/119714948/101797601/101790402	ADCY2/PKM/LOC101796516/LOC119714948/GPI/ATP5PO	6	6	101803512/101790726/101796516/119714948/101797601/101790402	0	
BP	GO:0009260	ribonucleotide biosynthetic process	6/329	33/3792	0.061022	0.575436	101803512/101790726/101796516/119714948/101797601/101790402	ADCY2/PKM/LOC101796516/LOC119714948/GPI/ATP5PO	6	6	101803512/101790726/101796516/119714948/101797601/101790402	0	
BP	GO:0046390	ribose phosphate biosynthetic process	6/329	33/3792	0.061022	0.575436	101803512/101790726/101796516/119714948/101797601/101790402	ADCY2/PKM/LOC101796516/LOC119714948/GPI/ATP5PO	6	6	101803512/101790726/101796516/119714948/101797601/101790402	0	
BP	GO:0034404	nucleobase-containing small molecule biosynthetic process	3/329	11/3792	0.063225	0.575436	101790726/101803472/101797601	PKM/ITPA/GPI	3	2	101790726/101797601	1	101803472
BP	GO:0072330	monocarboxylic acid biosynthetic process	3/329	11/3792	0.063225	0.575436	101797455/101790726/101797601	ASNS/PKM/GPI	3	2	101790726/101797601	1	101797455

BP	GO:0044281	small molecule metabolic process	22/329	181/3792	0.063244	0.575436	101798008/101795985/119714249/101799809/101789492/101801928/101799450/101803512/101804343/101797455/101790726/101803472/101796516/119714948/101795091/110351624/101797377/5405820/101797078/101797601/101800658/101790402	GADL1/LOC101795985/LOC119714249/HADHA/FH/ADSS1/GLUL/ADCY2/ACOX2/ASNS/PKM/ITPA/LOC101796516/LOC119714948/TARS3/LOC110351624/GLUD1/ND5/IP6K2/GPI/PCBD1/ATP5PO	22	15	101798008/119714249/101799809/101789492/101801928/101803512/101804343/101790726/101796516/119714948/101795091/110351624/5405820/101797601/101790402	7	101795985/101799450/101797455/101803472/101797377/101797078/101800658
BP	GO:0006754	ATP biosynthetic process	4/329	18/3792	0.064319	0.575436	101790726/119714948/101797601/101790402	PKM/LOC119714948/GPI/ATP5PO	4	4	101790726/119714948/101797601/101790402	0	
BP	GO:0009142	nucleoside triphosphate biosynthetic process	4/329	18/3792	0.064319	0.575436	101790726/119714948/101797601/101790402	PKM/LOC119714948/GPI/ATP5PO	4	4	101790726/119714948/101797601/101790402	0	
BP	GO:0009145	purine nucleoside triphosphate biosynthetic process	4/329	18/3792	0.064319	0.575436	101790726/119714948/101797601/101790402	PKM/LOC119714948/GPI/ATP5PO	4	4	101790726/119714948/101797601/101790402	0	
BP	GO:0009201	ribonucleoside triphosphate biosynthetic process	4/329	18/3792	0.064319	0.575436	101790726/119714948/101797601/101790402	PKM/LOC119714948/GPI/ATP5PO	4	4	101790726/119714948/101797601/101790402	0	
BP	GO:0009206	purine ribonucleoside triphosphate biosynthetic process	4/329	18/3792	0.064319	0.575436	101790726/119714948/101797601/101790402	PKM/LOC119714948/GPI/ATP5PO	4	4	101790726/119714948/101797601/101790402	0	
BP	GO:0044270	cellular nitrogen compound catabolic process	4/329	18/3792	0.064319	0.575436	101805057/101790726/101803472/101797601	HMOX1/PKM/ITPA/GPI	4	2	101790726/101797601	2	101805057/101803472
BP	GO:0046700	heterocycle catabolic process	4/329	18/3792	0.064319	0.575436	101805057/101790726/101803472/101797601	HMOX1/PKM/ITPA/GPI	4	2	101790726/101797601	2	101805057/101803472
BP	GO:0019439	aromatic compound catabolic process	4/329	19/3792	0.076129	0.575436	101805057/101790726/101803472/101797601	HMOX1/PKM/ITPA/GPI	4	2	101790726/101797601	2	101805057/101803472
BP	GO:1901361	organic cyclic compound catabolic process	4/329	19/3792	0.076129	0.575436	101805057/101790726/101803472/101797601	HMOX1/PKM/ITPA/GPI	4	2	101790726/101797601	2	101805057/101803472
BP	GO:0046034	ATP metabolic process	5/329	27/3792	0.078875	0.575436	101790726/119714948/5405820/101797601/101790402	PKM/LOC119714948/ND5/GPI/ATP5PO	5	5	101790726/119714948/5405820/101797601/101790402	0	
BP	GO:0045786	negative regulation of cell cycle	3/329	12/3792	0.079062	0.575436	101791694/101791211/101795780	CDKN1B/ZWILCH/MAD1L1	3	1	101795780	2	101791694/101791211
BP	GO:0009150	purine ribonucleotide metabolic process	7/329	44/3792	0.081903	0.575436	101803512/101790726/101796516/119714948/5405820/101797601/101790402	ADCY2/PKM/LOC101796516/LOC119714948/ND5/GPI/ATP5PO	7	7	101803512/101790726/101796516/119714948/5405820/101797601/101790402	0	
BP	GO:0009259	ribonucleotide metabolic process	7/329	44/3792	0.081903	0.575436	101803512/101790726/101796516/119714948/5405820/101797601/101790402	ADCY2/PKM/LOC101796516/LOC119714948/ND5/GPI/ATP5PO	7	7	101803512/101790726/101796516/119714948/5405820/101797601/101790402	0	
BP	GO:0007275	multicellular organism development	8/329	53/3792	0.083922	0.575436	101791711/101800783/101804245/101800985/101801755/101789522/101799824/101792356	WNT4/LOC101800783/PPDPFL/LAMA4/MYF5/MYOD1/WNT2/WNT5B	8	3	101791711/101804245/101800985	5	101800783/101801755/101789522/101799824/101792356
BP	GO:0009124	nucleoside monophosphate biosynthetic process	4/329	20/3792	0.088945	0.575436	101790726/119714948/101797601/101790402	PKM/LOC119714948/GPI/ATP5PO	4	4	101790726/119714948/101797601/101790402	0	
BP	GO:0009127	purine nucleoside monophosphate biosynthetic process	4/329	20/3792	0.088945	0.575436	101790726/119714948/101797601/101790402	PKM/LOC119714948/GPI/ATP5PO	4	4	101790726/119714948/101797601/101790402	0	
BP	GO:0009156	ribonucleoside monophosphate biosynthetic process	4/329	20/3792	0.088945	0.575436	101790726/119714948/101797601/101790402	PKM/LOC119714948/GPI/ATP5PO	4	4	101790726/119714948/101797601/101790402	0	

BP	GO:0009168	purine ribonucleoside monophosphate biosynthetic process	4/329	20/3792	0.088945	0.575436	101790726/119714948/101797601/101790402	PKM/LOC119714948/GPI/ATP5PO	4	4	101790726/119714948/101797601/101790402	0	
BP	GO:0009144	purine nucleoside triphosphate metabolic process	5/329	28/3792	0.089534	0.575436	101790726/119714948/5405820/101797601/101790402	PKM/LOC119714948/ND5/GPI/ATP5PO	5	5	101790726/119714948/5405820/101797601/101790402	0	
BP	GO:0009199	ribonucleoside triphosphate metabolic process	5/329	28/3792	0.089534	0.575436	101790726/119714948/5405820/101797601/101790402	PKM/LOC119714948/ND5/GPI/ATP5PO	5	5	101790726/119714948/5405820/101797601/101790402	0	
BP	GO:0009205	purine ribonucleoside triphosphate metabolic process	5/329	28/3792	0.089534	0.575436	101790726/119714948/5405820/101797601/101790402	PKM/LOC119714948/ND5/GPI/ATP5PO	5	5	101790726/119714948/5405820/101797601/101790402	0	
BP	GO:0006629	lipid metabolic process	15/329	120/3792	0.093278	0.575436	101796431/101795498/101799809/119713935/101803647/101790966/101796497/101804872/101804343/101805442/101799246/101804047/101796880/101799397/101790208	PNPLA8/PIP4K2A/HADHA/PNPLA1/PLCL2/PLCB1/PNPLA2/LOC101804872/ACOX2/IMPA2/PLCG2/PTDSS1/PLCD3/SRD5A2/SCD5	15	10	101795498/101799809/119713935/101803647/101790966/101804343/101805442/101804047/101799397/101790208	5	101796431/101796497/101804872/101799246/101796880
BP	GO:0019752	carboxylic acid metabolic process	11/329	82/3792	0.094695	0.575436	101798008/119714249/101799809/101789492/101799450/101804343/101797455/101790726/101795091/101797377/101797601	GADL1/LOC119714249/HADHA/FH/GLUL/ACOX2/ASNS/PKM/TARS3/GLUD1/GPI	11	8	101798008/119714249/101799809/101789492/101804343/101790726/101795091/101797601	3	101799450/101797455/101797377
BP	GO:0019693	ribose phosphate metabolic process	7/329	46/3792	0.098863	0.575436	101803512/101790726/101796516/119714948/5405820/101797601/101790402	ADCY2/PKM/LOC101796516/LOC119714948/ND5/GPI/ATP5PO	7	7	101803512/101790726/101796516/119714948/5405820/101797601/101790402	0	
BP	GO:0035556	intracellular signal transduction	21/329	181/3792	0.100378	0.575436	119717440/101796231/101803647/101790966/101795576/101803512/101795713/101792952/101799246/101799960/101796516/101802044/101795631/101795633/101801143/101790388/101796880/101795945/101799739/101792679/101791200	LOC119717440/TNS3/PLCL2/PLCB1/DCX/ADCY2/KALRN/ARHGEF3/PLCG2/RCAN1/LOC101796516/GPR155/LOC101795631/RAF1/DGKI/ARHGEF17/PLCD3/ASB4/RAB40B/PLEKHG1/SOCS3	21	14	119717440/101796231/101803647/101790966/101795576/101803512/101792952/101796516/101802044/101801143/101790388/101795945/101799739/101792679	7	101795713/101799246/101799960/101795631/101795633/101796880/101791200
BP	GO:0009123	nucleoside monophosphate metabolic process	5/329	29/3792	0.100898	0.575436	101790726/119714948/5405820/101797601/101790402	PKM/LOC119714948/ND5/GPI/ATP5PO	5	5	101790726/119714948/5405820/101797601/101790402	0	
BP	GO:0009126	purine nucleoside monophosphate metabolic process	5/329	29/3792	0.100898	0.575436	101790726/119714948/5405820/101797601/101790402	PKM/LOC119714948/ND5/GPI/ATP5PO	5	5	101790726/119714948/5405820/101797601/101790402	0	
BP	GO:0009161	ribonucleoside monophosphate metabolic process	5/329	29/3792	0.100898	0.575436	101790726/119714948/5405820/101797601/101790402	PKM/LOC119714948/ND5/GPI/ATP5PO	5	5	101790726/119714948/5405820/101797601/101790402	0	
BP	GO:0009167	purine ribonucleoside monophosphate metabolic process	5/329	29/3792	0.100898	0.575436	101790726/119714948/5405820/101797601/101790402	PKM/LOC119714948/ND5/GPI/ATP5PO	5	5	101790726/119714948/5405820/101797601/101790402	0	

BP	GO:0006082	organic acid metabolic process	11/329	83/3792	0.101157	0.575436	101798008/119714249/101799809/101789492/101799450/101804343/101797455/101790726/101795091/101797377/101797601	GADL1/LOC119714249/HADHA/FH/GLUL/ACOX2/ASNS/PKM/TARS3/GLUD1/GPI	11	8	101798008/119714249/101799809/101789492/101804343/101790726/101795091/101797601	3	101799450/101797455/101797377
BP	GO:0043436	oxoacid metabolic process	11/329	83/3792	0.101157	0.575436	101798008/119714249/101799809/101789492/101799450/101804343/101797455/101790726/101795091/101797377/101797601	GADL1/LOC119714249/HADHA/FH/GLUL/ACOX2/ASNS/PKM/TARS3/GLUD1/GPI	11	8	101798008/119714249/101799809/101789492/101804343/101790726/101795091/101797601	3	101799450/101797455/101797377
BP	GO:0048856	anatomical structure development	8/329	57/3792	0.116422	0.646706	101791711/101800783/101804245/101800985/101801755/101789522/101799824/101792356	WNT4/LOC101800783/PPDPFL/LAMA4/MYF5/MYOD1/WNT2/WNT5B	8	3	101791711/101804245/101800985	5	101800783/101801755/101789522/101799824/101792356
BP	GO:0051726	regulation of cell cycle	4/329	22/3792	0.117414	0.646706	101803833/101791694/101791211/101795780	RGCC/CDKN1B/ZWILCH/MAD1L1	4	1	101795780	3	101803833/101791694/101791211
BP	GO:0032502	developmental process	8/329	58/3792	0.125474	0.680305	101791711/101800783/101804245/101800985/101801755/101789522/101799824/101792356	WNT4/LOC101800783/PPDPFL/LAMA4/MYF5/MYOD1/WNT2/WNT5B	8	3	101791711/101804245/101800985	5	101800783/101801755/101789522/101799824/101792356
BP	GO:0046434	organophosphate catabolic process	4/329	23/3792	0.132956	0.700062	101798440/101790726/101803472/101797601	LOC101798440/PKM/ITPA/GPI	4	2	101790726/101797601	2	101798440/101803472
BP	GO:0015672	monovalent inorganic cation transport	8/329	59/3792	0.134884	0.700062	119714187/119714808/101793897/119717511/119714948/101799054/101797930/101790402	KCNN1/ATP1B2/ATP6V1E1/ASIC4/LOC119714948/SCN5A/TMEM38A/ATP5PO	8	7	119714187/119714808/119717511/119714948/101799054/101797930/101790402	1	101793897
BP	GO:0034655	nucleobase-containing compound catabolic process	3/329	15/3792	0.135171	0.700062	101790726/101803472/101797601	PKM/ITPA/GPI	3	2	101790726/101797601	1	101803472
BP	GO:0048523	negative regulation of cellular process	6/329	41/3792	0.139816	0.713473	101798286/101792754/101791694/101797044/101791211/101795780	EIF4EBP1/BAMBI/CDKN1B/LMOD2/ZWILCH/MAD1L1	6	1	101795780	5	101798286/101792754/101791694/101797044/101791211
BP	GO:1901137	carbohydrate derivative biosynthetic process	11/329	89/3792	0.145218	0.730297	101803248/101803512/101793948/101790726/101796516/101793440/119714948/101797601/101792011/101800484/101790402	B3GALNT2/ADCY2/B3GNT2/PKM/LOC101796516/ST6GALNAC6/LOC119714948/GPI/UGGT2/ST8SIA6/ATP5PO	11	9	101803512/101790726/101796516/101793440/119714948/101797601/101792011/101800484/101790402	2	101803248/101793948
BP	GO:0007166	cell surface receptor signaling pathway	7/329	51/3792	0.148882	0.738029	101791711/101791406/101799597/101799824/101792754/101792356/101791584	WNT4/CD247/FZD9/WNT2/BAMBI/WNT5B/SMAD6	7	2	101791711/101799597	5	101791406/101799824/101792754/101792356/101791584
BP	GO:0019637	organophosphate metabolic process	15/329	131/3792	0.160311	0.783492	101795498/101798440/101801928/101803512/101804872/101790726/101805442/101803472/101796516/119714948/101804047/5405820/101797078/101797601/101790402	PIP4K2A/LOC101798440/ADSS1/ADCY2/LOC101804872/PKM/IMPA2/ITPA/LOC119714948/PTDSS1/ND5/IP6K2/GPI/ATP5PO	15	11	101795498/101801928/101803512/101790726/101805442/101796516/119714948/101804047/5405820/101797601/101790402	4	101798440/101804872/101803472/101797078
BP	GO:0042221	response to chemical	4/329	25/3792	0.166346	0.786219	101800755/119713349/101792754/101791584	SESN1/SLC52A3/BAMBI/SMAD6	4	1	119713349	3	101800755/101792754/101791584
BP	GO:0006753	nucleoside phosphate metabolic process	9/329	72/3792	0.168202	0.786219	101801928/101803512/101790726/101803472/101796516/119714948/5405820/101797601/101790402	ADSS1/ADCY2/PKM/ITPA/LOC101796516/LOC119714948/ND5/GPI/ATP5PO	9	8	101801928/101803512/101790726/101796516/119714948/5405820/101797601/101790402	1	101803472

BP	GO:0009165	nucleotide biosynthetic process	7/329	53/3792	0.171696	0.786219	101801928/101803512/101790726/101796516/119714948/101797601/101790402	ADSS1/ADCY2/PKM/LOC101796516/LOC119714948/GPI/ATP5PO	7	7	101801928/101803512/101790726/101796516/119714948/101797601/101790402	0	
BP	GO:1901293	nucleoside phosphate biosynthetic process	7/329	53/3792	0.171696	0.786219	101801928/101803512/101790726/101796516/119714948/101797601/101790402	ADSS1/ADCY2/PKM/LOC101796516/LOC119714948/GPI/ATP5PO	7	7	101801928/101803512/101790726/101796516/119714948/101797601/101790402	0	
BP	GO:0055085	transmembrane transport	30/329	290/3792	0.172198	0.786219	101801029/101790978/101798538/101802086/119715418/101801744/101795198/101805016/101798013/101796355/101791662/101804595/101790476/101793897/101795224/101802044/119718656/119714948/101801311/101804200/101801967/101804688/101795086/101799219/101799054/101795852/119713969/101802999/101790402/101800270	SLC16A6/FLVCR1/AQP4/AQP9/KCNH2/KCNH6/SLC16A1/SLC7A2/MPC1/SLC16A3/SLC9A6/SLC39A13/ABCB6/ATP6V1E1/SLC43A2/GPR155/LOC119718656/LOC119714948/LOC101801311/SLC15A4/SLC2A12/ABCC8/KCNQ4/SLC22A4/SCN5A/SLC16A10/KCNC4/ABCC1/ATP5PO/KCNH7	30	18	101798538/101802086/119715418/101801744/101795198/101805016/101796355/101790476/101802044/119714948/101801311/101801967/101804688/101799219/101799054/101802999/101790402/101800270	12	101801029/101790978/101798013/101791662/101804595/101793897/101795224/119718656/101804200/101795086/101795852/119713969
BP	GO:0006814	sodium ion transport	3/329	17/3792	0.178251	0.803289	119714808/119717511/101799054	ATP1B2/ASIC4/SCN5A	3	3	119714808/119717511/101799054	0	
BP	GO:0016310	phosphorylation	37/329	367/3792	0.180572	0.803314	101801853/101793905/101793152/101802614/101803371/101802855/101797616/101799089/101803562/101803181/101791030/101804342/101791519/101799561/101800942/101802737/101793068/101795713/101790169/101790726/101805442/101792645/101797446/101800595/101804452/101795633/101801648/101801641/101791769/101793707/106017602/101791037/101796806/5405820/101797601/101797327/101789868	TNIK/ULK2/WNK2/PRKG1/MAP2K3/CAMK1G/LOC101797616/MAST4/TRIB1/MAP3K6/STYK1/SIK1/MAP3K15/PRKAA2/RPS6KA1/CLK1/PHKG1/KALRN/LOC101790169/PKM/IMPA2/PIM3/OXSR1/RNASEL/MUSK/RAF1/CAMK2D/LOC101801641/MAP3K8/BMPR2/TSSK2/MAP2K1/CDK10/ND5/GPI/LOC101797327/PXK	37	16	101801853/101793152/101802614/101802855/101799089/101791519/101799561/101790726/101805442/101800595/101804452/101801648/101793707/5405820/101797601/101797327	21	101793905/101803371/101797616/101803562/101803181/101791030/101804342/101800942/101802737/101793068/101795713/101790169/101792645/101797446/101795633/101801641/101791769/106017602/101791037/101796806/101789868
BP	GO:0009108	coenzyme biosynthetic process	4/329	27/3792	0.202348	0.869751	101799171/101790726/101797601/101800658	MAT1A/PKM/GPI/PCBD1	4	3	101799171/101790726/101797601	1	101800658
BP	GO:0006811	ion transport	26/329	253/3792	0.203273	0.869751	106020272/119714187/101802251/101795864/119713349/119715418/101801744/119714808/101798013/101804872/101791662/101804595/101800466/101793897/119717511/119714948/101801311/101793641/101795086/101798131/101799054/101798462/119713969/101797930/101790402/101800270	CHRNG/KCNN1/PITPNM1/KCNE4/SLC52A3/KCNH2/KCNH6/ATP1B2/MPC1/LOC101804872/SLC9A6/SLC39A13/CHRNA10/ATP6V1E1/ASIC4/LOC119714948/LOC101801311/ATP13A2/KCNQ4/PITPNC1/SCN5A/GABRG3/KCNC4/TMEM38A/ATP5PO/KCNH7	26	16	106020272/119714187/101795864/119713349/119715418/101801744/119714808/119717511/119714948/101801311/101798131/101799054/101798462/101797930/101790402/101800270	10	101802251/101798013/101804872/101791662/101804595/101800466/101793897/101793641/101795086/119713969

BP	GO:0048519	negative regulation of biological process	6/329	46/3792	0.204991	0.869751	101798286/101792754/101791694/101797044/101791211/101795780	EIF4EBP1/BAMBI/CDKN1B/LMOD2/ZWILCH/MAD1L1	6	1	101795780	5	101798286/101792754/101791694/101797044/101791211
BP	GO:0006066	alcohol metabolic process	2/329	10/3792	0.213051	0.869751	101797078/101800658	IP6K2/PCBD1	2	0		2	101797078/101800658
BP	GO:1901605	alpha-amino acid metabolic process	2/329	10/3792	0.213051	0.869751	101799450/101797455	GLUL/ASNS	2	0		2	101799450/101797455
BP	GO:1901615	organic hydroxy compound metabolic process	2/329	10/3792	0.213051	0.869751	101797078/101800658	IP6K2/PCBD1	2	0		2	101797078/101800658
BP	GO:1903047	mitotic cell cycle process	2/329	10/3792	0.213051	0.869751	101791211/101795780	ZWILCH/MAD1L1	2	1	101795780	1	101791211
BP	GO:0006928	movement of cell or subcellular component	8/329	67/3792	0.221741	0.881259	101797173/101800985/101795653/101790443/119714941/106020577/101803768/101790717	DNAH5/LAMA4/DNAH3/KIF26A/LOC119714941/KIF3C/KIF16B/DNAH9	8	6	101797173/101800985/101795653/101790443/106020577/101790717	2	119714941/101803768
BP	GO:0048731	system development	3/329	19/3792	0.224458	0.881259	101800783/101801755/101789522	LOC101800783/MYF5/MYOD1	3	0		3	101800783/101801755/101789522
BP	GO:0000278	mitotic cell cycle	2/329	11/3792	0.246346	0.881259	101791211/101795780	ZWILCH/MAD1L1	2	1	101795780	1	101791211
BP	GO:0006413	translational initiation	2/329	11/3792	0.246346	0.881259	101798286/101793553	EIF4EBP1/EIF1	2	0		2	101798286/101793553
BP	GO:0015718	monocarboxylic acid transport	2/329	11/3792	0.246346	0.881259	101798013/101804872	MPC1/LOC101804872	2	0		2	101798013/101804872
BP	GO:0015849	organic acid transport	2/329	11/3792	0.246346	0.881259	101798013/101804872	MPC1/LOC101804872	2	0		2	101798013/101804872
BP	GO:0015985	energy coupled proton transport, down electrochemical gradient	2/329	11/3792	0.246346	0.881259	119714948/101790402	LOC119714948/ATP5PO	2	2	119714948/101790402	0	
BP	GO:0015986	ATP synthesis coupled proton transport	2/329	11/3792	0.246346	0.881259	119714948/101790402	LOC119714948/ATP5PO	2	2	119714948/101790402	0	
BP	GO:0019359	nicotinamide nucleotide biosynthetic process	2/329	11/3792	0.246346	0.881259	101790726/101797601	PKM/GPI	2	2	101790726/101797601	0	
BP	GO:0019363	pyridine nucleotide biosynthetic process	2/329	11/3792	0.246346	0.881259	101790726/101797601	PKM/GPI	2	2	101790726/101797601	0	
BP	GO:0046942	carboxylic acid transport	2/329	11/3792	0.246346	0.881259	101798013/101804872	MPC1/LOC101804872	2	0		2	101798013/101804872
BP	GO:0072525	pyridine-containing compound biosynthetic process	2/329	11/3792	0.246346	0.881259	101790726/101797601	PKM/GPI	2	2	101790726/101797601	0	
BP	GO:1901135	carbohydrate derivative metabolic process	13/329	122/3792	0.257265	0.89384	101798440/101803248/101803512/101793948/101790726/101796516/101793440/119714948/5405820/101797601/101792011/101800484/101790402	LOC101798440/B3GALNT2/ADCY2/B3GNT2/PKM/LOC101796516/ST6GALNAC6/LOC119714948/ND5/GPI/UGGT2/ST8SIA6/ATP5PO	13	10	101803512/101790726/101796516/101793440/119714948/5405820/101797601/101792011/101800484/101790402	3	101798440/101803248/101793948
BP	GO:0007018	microtubule-based movement	7/329	60/3792	0.261661	0.89384	101797173/101795653/101790443/119714941/106020577/101803768/101790717	DNAH5/DNAH3/KIF26A/LOC119714941/KIF3C/KIF16B/DNAH9	7	5	101797173/101795653/101790443/106020577/101790717	2	119714941/101803768
BP	GO:0009117	nucleotide metabolic process	8/329	71/3792	0.271449	0.89384	101801928/101803512/101790726/101796516/119714948/5405820/101797601/101790402	ADSS1/ADCY2/PKM/LOC101796516/LOC119714948/ND5/GPI/ATP5PO	8	8	101801928/101803512/101790726/101796516/119714948/5405820/101797601/101790402	0	
BP	GO:0022402	cell cycle process	3/329	21/3792	0.272647	0.89384	101791694/101791211/101795780	CDKN1B/ZWILCH/MAD1L1	3	1	101795780	2	101791694/101791211
BP	GO:0055086	nucleobase-containing small molecule metabolic process	9/329	82/3792	0.278944	0.89384	101801928/101803512/101790726/101803472/101796516/119714948/5405820/101797601/101790402	ADSS1/ADCY2/PKM/ITPA/LOC101796516/LOC119714948/ND5/GPI/ATP5PO	9	8	101801928/101803512/101790726/101796516/119714948/5405820/101797601/101790402	1	101803472
BP	GO:0006471	protein ADP-ribosylation	2/329	12/3792	0.279803	0.89384	101800656/119716831	LOC101800656/LOC119716831	2	1	101800656	1	119716831
BP	GO:0006732	coenzyme metabolic process	4/329	31/3792	0.279901	0.89384	101799171/101790726/101797601/101800658	MAT1A/PKM/GPI/PCBD1	4	3	101799171/101790726/101797601	1	101800658
BP	GO:0006486	protein glycosylation	5/329	41/3792	0.280774	0.89384	101803248/101793948/101793440/101792011/101800484	B3GALNT2/B3GNT2/ST6GALNAC6/UGGT2/ST8SIA6	5	3	101793440/101792011/101800484	2	101803248/101793948

BP	GO:0009100	glycoprotein metabolic process	5/329	41/3792	0.280774	0.89384	101803248/101793948/101793440/101792011/101800484	B3GALNT2/B3GNT2/ST6GALNAC6/UGGT2/ST8SIA6	5	3	101793440/101792011/101800484	2	101803248/101793948
BP	GO:0009101	glycoprotein biosynthetic process	5/329	41/3792	0.280774	0.89384	101803248/101793948/101793440/101792011/101800484	B3GALNT2/B3GNT2/ST6GALNAC6/UGGT2/ST8SIA6	5	3	101793440/101792011/101800484	2	101803248/101793948
BP	GO:0043413	macromolecule glycosylation	5/329	41/3792	0.280774	0.89384	101803248/101793948/101793440/101792011/101800484	B3GALNT2/B3GNT2/ST6GALNAC6/UGGT2/ST8SIA6	5	3	101793440/101792011/101800484	2	101803248/101793948
BP	GO:0070085	glycosylation	5/329	41/3792	0.280774	0.89384	101803248/101793948/101793440/101792011/101800484	B3GALNT2/B3GNT2/ST6GALNAC6/UGGT2/ST8SIA6	5	3	101793440/101792011/101800484	2	101803248/101793948
BP	GO:0032501	multicellular organismal process	8/329	72/3792	0.284355	0.89701	101791711/101800783/101804245/101800985/101801755/101789522/101799824/101792356	WNT4/LOC101800783/PPDPFL/LAMA4/MYF5/MYOD1/WNT2/WNT5B	8	3	101791711/101804245/101800985	5	101800783/101801755/101789522/101799824/101792356
BP	GO:0006820	anion transport	5/329	42/3792	0.298177	0.913118	101802251/119713349/101798013/101804872/101798131	PITPNM1/SLC52A3/MPC1/LOC101804872/PITPNC1	5	2	119713349/101798131	3	101802251/101798013/101804872
BP	GO:0090407	organophosphate biosynthetic process	9/329	84/3792	0.303327	0.913118	101801928/101803512/101790726/101796516/119714948/101804047/101797078/101797601/101790402	ADSS1/ADCY2/PKM/LOC101796516/LOC119714948/PTDSS1/IP6K2/GPI/ATP5PO	9	8	101801928/101803512/101790726/101796516/119714948/101804047/101797601/101790402	1	101797078
BP	GO:1901575	organic substance catabolic process	9/329	84/3792	0.303327	0.913118	101799480/101798440/101790966/101805057/101804343/101790726/101803472/101803700/101797601	UCHL1/LOC101798440/PLCB1/HMOX1/ACOX2/PKM/ITPA/PSMB1/GPI	9	4	101790966/101804343/101790726/101797601	5	101799480/101798440/101805057/101803472/101803700
BP	GO:0007167	enzyme linked receptor protein signaling pathway	2/329	13/3792	0.313144	0.913118	101792754/101791584	BAMBI/SMAD6	2	0		2	101792754/101791584
BP	GO:0010639	negative regulation of organelle organization	2/329	13/3792	0.313144	0.913118	101797044/101795780	LMOD2/MAD1L1	2	1	101795780	1	101797044
BP	GO:0019362	pyridine nucleotide metabolic process	2/329	13/3792	0.313144	0.913118	101790726/101797601	PKM/GPI	2	2	101790726/101797601	0	
BP	GO:0030154	cell differentiation	2/329	13/3792	0.313144	0.913118	101800783/101804245	LOC101800783/PPDPFL	2	1	101804245	1	101800783
BP	GO:0046496	nicotinamide nucleotide metabolic process	2/329	13/3792	0.313144	0.913118	101790726/101797601	PKM/GPI	2	2	101790726/101797601	0	
BP	GO:0072524	pyridine-containing compound metabolic process	2/329	13/3792	0.313144	0.913118	101790726/101797601	PKM/GPI	2	2	101790726/101797601	0	
BP	GO:0006468	protein phosphorylation	33/329	348/3792	0.316132	0.914149	101801853/101793905/101793152/101802614/101803371/101802855/101797616/101799089/101803562/101803181/101791030/101804342/101791519/101799561/101800942/101802737/101793068/101795713/101790169/101792645/101797446/101800595/101804452/101795633/101801648/101801641/101791769/101793707/106017602/101791037/101796806/101797327/101789868	TNIK/ULK2/WNK2/PRKG1/MAP2K3/CAMK1G/LOC101797616/MAST4/TRIB1/MAP3K6/STYK1/SIK1/MAP3K15/PRKAA2/RPS6KA1/CLK1/PHKG1/KALRN/LOC101790169/PIM3/OXSR1/RNASEL/MUSK/RAF1/CAMK2D/LOC101801641/MAP3K8/BMPR2/TSSK2/MAP2K1/CDK10/LOC101797327/PXK	33	12	101801853/101793152/101802614/101802855/101799089/101791519/101799561/101800595/101804452/101801648/101793707/101797327	21	101793905/101803371/101797616/101803562/101803181/101791030/101804342/101800942/101802737/101793068/101795713/101790169/101792645/101797446/101795633/101801641/101791769/106017602/101791037/101796806/101789868
BP	GO:0009190	cyclic nucleotide biosynthetic process	2/329	14/3792	0.346138	0.946547	101803512/101796516	ADCY2/LOC101796516	2	2	101803512/101796516	0	
BP	GO:0009719	response to endogenous stimulus	2/329	14/3792	0.346138	0.946547	101792754/101791584	BAMBI/SMAD6	2	0		2	101792754/101791584
BP	GO:0048869	cellular developmental process	2/329	14/3792	0.346138	0.946547	101800783/101804245	LOC101800783/PPDPFL	2	1	101804245	1	101800783

BP	GO:0051129	negative regulation of cellular component organization	2/329	14/3792	0.346138	0.946547	101797044/101795780	LMOD2/MAD1L1	2	1	101795780	1	101797044
BP	GO:0071495	cellular response to endogenous stimulus	2/329	14/3792	0.346138	0.946547	101792754/101791584	BAMBI/SMAD6	2	0		2	101792754/101791584
BP	GO:0006650	glycerophospholipid metabolic process	3/329	24/3792	0.346431	0.946547	101795498/101805442/101804047	PIP4K2A/IMPA2/PTDSS1	3	3	101795498/101805442/101804047	0	
BP	GO:0046486	glycerolipid metabolic process	3/329	24/3792	0.346431	0.946547	101795498/101805442/101804047	PIP4K2A/IMPA2/PTDSS1	3	3	101795498/101805442/101804047	0	
BP	GO:0007049	cell cycle	4/329	35/3792	0.361153	0.966797	101803833/101791694/101791211/101795780	RGCC/CDKN1B/ZWILCH/MAD1L1	4	1	101795780	3	101803833/101791694/101791211
BP	GO:0051188	cofactor biosynthetic process	4/329	35/3792	0.361153	0.966797	101799171/101790726/101797601/101800658	MAT1A/PKM/GPI/PCBD1	4	3	101799171/101790726/101797601	1	101800658
BP	GO:0006950	response to stress	9/329	89/3792	0.36615	0.966797	101800755/101794631/101800783/101795336/novel.277/101790058/101796914/101802532/101797870	SESN1/LOC101794631/LOC101800783/SHLD1/-/BLM/LIG1/MSH2/XRCC5	9	2	101790058/101797870	7	101800755/101794631/101800783/101795336/novel.277/101796914/101802532
BP	GO:0006733	oxidoreduction coenzyme metabolic process	2/329	15/3792	0.378595	0.966797	101790726/101797601	PKM/GPI	2	2	101790726/101797601	0	
BP	GO:0006813	potassium ion transport	2/329	15/3792	0.378595	0.966797	119714187/119714808	KCNN1/ATP1B2	2	2	119714187/119714808	0	
BP	GO:0009187	cyclic nucleotide metabolic process	2/329	15/3792	0.378595	0.966797	101803512/101796516	ADCY2/LOC101796516	2	2	101803512/101796516	0	
BP	GO:0070887	cellular response to chemical stimulus	2/329	15/3792	0.378595	0.966797	101792754/101791584	BAMBI/SMAD6	2	0		2	101792754/101791584
BP	GO:0071310	cellular response to organic substance	2/329	15/3792	0.378595	0.966797	101792754/101791584	BAMBI/SMAD6	2	0		2	101792754/101791584
BP	GO:0048583	regulation of response to stimulus	9/329	90/3792	0.378918	0.966797	101800755/101803486/101795336/101795713/101792952/106015345/101792754/101790388/101792679	SESN1/LOC101803486/SHLD1/KALRN/ARHGEF3/GPC1/BAMBI/ARHGEF17/PLEKHG1	9	5	101803486/101792952/106015345/101790388/101792679	4	101800755/101795336/101795713/101792754
BP	GO:0051186	cofactor metabolic process	5/329	47/3792	0.386918	0.979954	101799171/101805057/101790726/101797601/101800658	MAT1A/HMOX1/PKM/GPI/PCBD1	5	3	101799171/101790726/101797601	2	101805057/101800658
BP	GO:0009056	catabolic process	9/329	91/3792	0.39172	0.979954	101799480/101798440/101790966/101805057/101804343/101790726/101803472/101803700/101797601	UCHL1/LOC101798440/PLCB1/HMOX1/ACOX2/PKM/ITPA/PSMB1/GPI	9	4	101790966/101804343/101790726/101797601	5	101799480/101798440/101805057/101803472/101803700
BP	GO:0050790	regulation of catalytic activity	3/329	26/3792	0.39537	0.979954	101795631/101794268/119714472	LOC101795631/CTSB/LOC119714472	3	0		3	101795631/101794268/119714472
BP	GO:0065009	regulation of molecular function	3/329	26/3792	0.39537	0.979954	101795631/101794268/119714472	LOC101795631/CTSB/LOC119714472	3	0		3	101795631/101794268/119714472
BP	GO:0006508	proteolysis	24/329	260/3792	0.404839	0.996306	101791511/101803168/101794528/101799480/101794584/101792575/101804552/101804246/113839605/101794820/101791397/101797901/101794268/119714472/101800115/119714444/101791234/101797577/101794402/101803700/101804308/101790574/101792943/101800508	PHEX/CTSL/LOC101794528/UCHL1/ADAMTS17/USP44/PLAU/CTSZ/LOC113839605/LOC101794820/DPP7/DPEP1/CTSB/LOC119714472/ADAMTS18/ADAMTS19/USP49/SCPEP1/TINAG/PSMB1/MMP23B/ADAMTS5/PIGK/LOC101800508	24	7	101791511/101797901/101800115/119714444/101804308/101792943/101800508	17	101803168/101794528/101799480/101794584/101792575/101804552/101804246/113839605/101794820/101791397/101794268/119714472/101791234/101797577/101794402/101803700/101790574
BP	GO:0042157	lipoprotein metabolic process	2/329	16/3792	0.410359	0.999979	101797331/101798726	APOA4/APOLD1	2	1	101797331	1	101798726
BP	GO:0006812	cation transport	11/329	116/3792	0.425244	0.999979	119714187/119714808/101791662/101804595/101793897/119717511/119714948/101793641/101799054/101797930/101790402	KCNN1/ATP1B2/SLC9A6/SLC39A13/ATP6V1E1/ASIC4/LOC119714948/ATP13A2/SCN5A/TMEM38A/ATP5PO	11	7	119714187/119714808/119717511/119714948/101799054/101797930/101790402	4	101791662/101804595/101793897/101793641

BP	GO:0010033	response to organic substance	2/329	17/3792	0.441306	0.999979	101792754/101791584	BAMBI/SMAD6	2	0		2	101792754/101791584
BP	GO:0061024	membrane organization	2/329	17/3792	0.441306	0.999979	101792227/101801562	CAV3/APOOL	2	2	101792227/101801562	0	
BP	GO:0010941	regulation of cell death	4/329	40/3792	0.462036	0.999979	101798341/101797616/101789899/101796820	BNIP3/LOC101797616/LOC101789899/LOC101796820	4	0		4	101798341/101797616/101789899/101796820
BP	GO:0042981	regulation of apoptotic process	4/329	40/3792	0.462036	0.999979	101798341/101797616/101789899/101796820	BNIP3/LOC101797616/LOC101789899/LOC101796820	4	0		4	101798341/101797616/101789899/101796820
BP	GO:0043067	regulation of programmed cell death	4/329	40/3792	0.462036	0.999979	101798341/101797616/101789899/101796820	BNIP3/LOC101797616/LOC101789899/LOC101796820	4	0		4	101798341/101797616/101789899/101796820
BP	GO:1902600	proton transmembrane transport	3/329	29/3792	0.466712	0.999979	101793897/119714948/101790402	ATP6V1E1/LOC119714948/ATP5PO	3	2	119714948/101790402	1	101793897
BP	GO:0034220	ion transmembrane transport	4/329	42/3792	0.500943	0.999979	101798013/101793897/119714948/101790402	MPC1/ATP6V1E1/LOC119714948/ATP5PO	4	2	119714948/101790402	2	101798013/101793897
BP	GO:1901566	organonitrogen compound biosynthetic process	23/329	261/3792	0.502295	0.999979	101798286/119714249/101801928/101803248/101799450/101802140/101803512/101793948/101797455/101793553/101790726/101796516/101793440/119714948/101795091/110351624/106017870/101804047/101797601/101792011/101800484/101800658/101790402	EIF4EBP1/LOC119714249/ADSS1/B3GALNT2/GLUL/LOC101802140/ADCY2/B3GNT2/ASNS/EIF1/PKM/LOC101796516/ST6GALNAC6/LOC119714948/TARS3/LOC110351624/MRPL2/PTDSS1/GPI/UGGT2/ST8SIA6/PCBD1/ATP5PO	23	16	119714249/101801928/101802140/101803512/101790726/101796516/101793440/119714948/101795091/110351624/106017870/101804047/101797601/101792011/101800484/101790402	7	101798286/101803248/101799450/101793948/101797455/101793553/101800658
BP	GO:0044248	cellular catabolic process	7/329	78/3792	0.521588	0.999979	101799480/101805057/101804343/101790726/101803472/101803700/101797601	UCHL1/HMOX1/ACOX2/PKM/ITPA/PSMB1/GPI	7	3	101804343/101790726/101797601	4	101799480/101805057/101803472/101803700
BP	GO:0006281	DNA repair	5/329	55/3792	0.525994	0.999979	101795336/101790058/101796914/101802532/101797870	SHLD1/BLM/LIG1/MSH2/XRCC5	5	2	101790058/101797870	3	101795336/101796914/101802532
BP	GO:0016042	lipid catabolic process	2/329	20/3792	0.528386	0.999979	101790966/101804343	PLCB1/ACOX2	2	2	101790966/101804343	0	
BP	GO:0044255	cellular lipid metabolic process	6/329	68/3792	0.546045	0.999979	101795498/101799809/101804872/101804343/101805442/101804047	PIP4K2A/HADHA/LOC101804872/ACOX2/IMPA2/PTDSS1	6	5	101795498/101799809/101804343/101805442/101804047	1	101804872
BP	GO:0006457	protein folding	2/329	21/3792	0.555306	0.999979	101801226/101804490	DNAJA4/GRPEL2	2	1	101804490	1	101801226
BP	GO:0046488	phosphatidylinositol metabolic process	2/329	21/3792	0.555306	0.999979	101795498/101805442	PIP4K2A/IMPA2	2	2	101795498/101805442	0	
BP	GO:0007266	Rho protein signal transduction	4/329	45/3792	0.556863	0.999979	101795713/101792952/101790388/101792679	KALRN/ARHGEF3/ARHGEF17/PLEKHG1	4	3	101792952/101790388/101792679	1	101795713
BP	GO:0035023	regulation of Rho protein signal transduction	4/329	45/3792	0.556863	0.999979	101795713/101792952/101790388/101792679	KALRN/ARHGEF3/ARHGEF17/PLEKHG1	4	3	101792952/101790388/101792679	1	101795713
BP	GO:0006520	cellular amino acid metabolic process	5/329	57/3792	0.558727	0.999979	119714249/101799450/101797455/101795091/101797377	LOC119714249/GLUL/ASNS/TARS3/GLUD1	5	2	119714249/101795091	3	101799450/101797455/101797377
BP	GO:0007017	microtubule-based process	7/329	81/3792	0.562948	0.999979	101797173/101795653/101790443/119714941/106020577/101803768/101790717	DNAH5/DNAH3/KIF26A/LOC119714941/KIF3C/KIF16B/DNAH9	7	5	101797173/101795653/101790443/106020577/101790717	2	119714941/101803768
BP	GO:0009966	regulation of signal transduction	7/329	83/3792	0.589608	0.999979	101803486/101795713/101792952/106015345/101792754/101790388/101792679	LOC101803486/KALRN/ARHGEF3/GPC1/BAMBI/ARHGEF17/PLEKHG1	7	5	101803486/101792952/106015345/101790388/101792679	2	101795713/101792754

BP	GO:0010646	regulation of cell communication	7/329	83/3792	0.589608	0.999979	101803486/101795713/101792952/106015345/101792754/101790388/101792679	LOC101803486/KALRN/ARHGEF3/GPC1/BAMBI/ARHGEF17/PLEKHG1	7	5	101803486/101792952/106015345/101790388/101792679	2	101795713/101792754
BP	GO:0023051	regulation of signaling	7/329	83/3792	0.589608	0.999979	101803486/101795713/101792952/106015345/101792754/101790388/101792679	LOC101803486/KALRN/ARHGEF3/GPC1/BAMBI/ARHGEF17/PLEKHG1	7	5	101803486/101792952/106015345/101790388/101792679	2	101795713/101792754
BP	GO:0006915	apoptotic process	4/329	47/3792	0.59222	0.999979	101798341/101797616/101789899/101796820	BNIP3/LOC101797616/LOC101789899/LOC101796820	4	0		4	101798341/101797616/101789899/101796820
BP	GO:0008219	cell death	4/329	47/3792	0.59222	0.999979	101798341/101797616/101789899/101796820	BNIP3/LOC101797616/LOC101789899/LOC101796820	4	0		4	101798341/101797616/101789899/101796820
BP	GO:0012501	programmed cell death	4/329	47/3792	0.59222	0.999979	101798341/101797616/101789899/101796820	BNIP3/LOC101797616/LOC101789899/LOC101796820	4	0		4	101798341/101797616/101789899/101796820
BP	GO:0006605	protein targeting	1/329	10/3792	0.596957	0.999979	101793444	AKAP12	1	1	101793444	0	
BP	GO:0006952	defense response	1/329	10/3792	0.596957	0.999979	novel.277	-	1	0		1	novel.277
BP	GO:0007005	mitochondrion organization	1/329	10/3792	0.596957	0.999979	101801562	APOOL	1	1	101801562	0	
BP	GO:0007059	chromosome segregation	1/329	10/3792	0.596957	0.999979	101795780	MAD1L1	1	1	101795780	0	
BP	GO:0010256	endomembrane system organization	1/329	10/3792	0.596957	0.999979	101792227	CAV3	1	1	101792227	0	
BP	GO:0010558	negative regulation of macromolecule biosynthetic process	1/329	10/3792	0.596957	0.999979	101798286	EIF4EBP1	1	0		1	101798286
BP	GO:0031327	negative regulation of cellular biosynthetic process	1/329	10/3792	0.596957	0.999979	101798286	EIF4EBP1	1	0		1	101798286
BP	GO:0043244	regulation of protein complex disassembly	1/329	10/3792	0.596957	0.999979	101797044	LMOD2	1	0		1	101797044
BP	GO:0050793	regulation of developmental process	1/329	10/3792	0.596957	0.999979	101800985	LAMA4	1	1	101800985	0	
BP	GO:2000113	negative regulation of cellular macromolecule biosynthetic process	1/329	10/3792	0.596957	0.999979	101798286	EIF4EBP1	1	0		1	101798286
BP	GO:0006644	phospholipid metabolic process	4/329	48/3792	0.609265	0.999979	101795498/101804872/101805442/101804047	PIP4K2A/LOC101804872/IMPA2/PTDSS1	4	3	101795498/101805442/101804047	1	101804872
BP	GO:0030001	metal ion transport	5/329	61/3792	0.620611	0.999979	119714187/119714808/101804595/119717511/101799054	KCNN1/ATP1B2/SLC39A13/ASIC4/SCN5A	5	4	119714187/119714808/119717511/101799054	1	101804595
BP	GO:0048522	positive regulation of cellular process	2/329	24/3792	0.6294	0.999979	101798341/101792754	BNIP3/BAMBI	2	0		2	101798341/101792754
BP	GO:0009890	negative regulation of biosynthetic process	1/329	11/3792	0.632018	0.999979	101798286	EIF4EBP1	1	0		1	101798286
BP	GO:0032268	regulation of cellular protein metabolic process	1/329	11/3792	0.632018	0.999979	101798286	EIF4EBP1	1	0		1	101798286
BP	GO:0033013	tetrapyrrole metabolic process	1/329	11/3792	0.632018	0.999979	101805057	HMOX1	1	0		1	101805057
BP	GO:0048285	organelle fission	1/329	11/3792	0.632018	0.999979	101795780	MAD1L1	1	1	101795780	0	
BP	GO:0051172	negative regulation of nitrogen compound metabolic process	1/329	11/3792	0.632018	0.999979	101798286	EIF4EBP1	1	0		1	101798286
BP	GO:0051494	negative regulation of cytoskeleton organization	1/329	11/3792	0.632018	0.999979	101797044	LMOD2	1	0		1	101797044
BP	GO:0002376	immune system process	3/329	37/3792	0.634847	0.999979	101795038/101803777/101800542	CX3CL1/TNFSF13B/ENPP3	3	2	101795038/101800542	1	101803777
BP	GO:0006955	immune response	3/329	37/3792	0.634847	0.999979	101795038/101803777/101800542	CX3CL1/TNFSF13B/ENPP3	3	2	101795038/101800542	1	101803777
BP	GO:0006974	cellular response to DNA damage stimulus	5/329	62/3792	0.635257	0.999979	101795336/101790058/101796914/101802532/101797870	SHLD1/BLM/LIG1/MSH2/XRCC5	5	2	101790058/101797870	3	101795336/101796914/101802532
BP	GO:0033554	cellular response to stress	5/329	62/3792	0.635257	0.999979	101795336/101790058/101796914/101802532/101797870	SHLD1/BLM/LIG1/MSH2/XRCC5	5	2	101790058/101797870	3	101795336/101796914/101802532
BP	GO:0033043	regulation of organelle organization	2/329	25/3792	0.651871	0.999979	101797044/101795780	LMOD2/MAD1L1	2	1	101795780	1	101797044
BP	GO:0071702	organic substance transport	10/329	125/3792	0.655291	0.999979	101802251/119713349/101798013/101804872/101797331/101792755/101793444/101798726/101801245/101798131	PITPNM1/SLC52A3/MPC1/LOC101804872/APOA4/LOC101792755/AKAP12/APOLD1/SELENOS/PITPNC1	10	4	119713349/101797331/101793444/101798131	6	101802251/101798013/101804872/101792755/101798726/101801245

BP	GO:0031324	negative regulation of cellular metabolic process	1/329	12/3792	0.664038	0.999979	101798286	EIF4EBP1	1	0		1	101798286
BP	GO:0051239	regulation of multicellular organismal process	1/329	12/3792	0.664038	0.999979	101800985	LAMA4	1	1	101800985	0	
BP	GO:0051246	regulation of protein metabolic process	1/329	12/3792	0.664038	0.999979	101798286	EIF4EBP1	1	0		1	101798286
BP	GO:0048518	positive regulation of biological process	2/329	26/3792	0.673246	0.999979	101798341/101792754	BNIP3/BAMBI	2	0		2	101798341/101792754
BP	GO:0098655	cation transmembrane transport	3/329	40/3792	0.687569	0.999979	101793897/119714948/101790402	ATP6V1E1/LOC119714948/ATP5PO	3	2	119714948/101790402	1	101793897
BP	GO:0098660	inorganic ion transmembrane transport	3/329	40/3792	0.687569	0.999979	101793897/119714948/101790402	ATP6V1E1/LOC119714948/ATP5PO	3	2	119714948/101790402	1	101793897
BP	GO:0098662	inorganic cation transmembrane transport	3/329	40/3792	0.687569	0.999979	101793897/119714948/101790402	ATP6V1E1/LOC119714948/ATP5PO	3	2	119714948/101790402	1	101793897
BP	GO:0007265	Ras protein signal transduction	4/329	53/3792	0.687674	0.999979	101795713/101792952/101790388/101792679	KALRN/ARHGEF3/ARHGEF17/PLEKHG1	4	3	101792952/101790388/101792679	1	101795713
BP	GO:0046578	regulation of Ras protein signal transduction	4/329	53/3792	0.687674	0.999979	101795713/101792952/101790388/101792679	KALRN/ARHGEF3/ARHGEF17/PLEKHG1	4	3	101792952/101790388/101792679	1	101795713
BP	GO:0006334	nucleosome assembly	1/329	13/3792	0.693279	0.999979	101798499	LOC101798499	1	0		1	101798499
BP	GO:0010629	negative regulation of gene expression	1/329	13/3792	0.693279	0.999979	101798286	EIF4EBP1	1	0		1	101798286
BP	GO:0015988	energy coupled proton transmembrane transport, against electrochemical gradient	1/329	13/3792	0.693279	0.999979	101793897	ATP6V1E1	1	0		1	101793897
BP	GO:0015991	ATP hydrolysis coupled proton transport	1/329	13/3792	0.693279	0.999979	101793897	ATP6V1E1	1	0		1	101793897
BP	GO:0031497	chromatin assembly	1/329	13/3792	0.693279	0.999979	101798499	LOC101798499	1	0		1	101798499
BP	GO:0090662	ATP hydrolysis coupled transmembrane transport	1/329	13/3792	0.693279	0.999979	101793897	ATP6V1E1	1	0		1	101793897
BP	GO:0099131	ATP hydrolysis coupled ion transmembrane transport	1/329	13/3792	0.693279	0.999979	101793897	ATP6V1E1	1	0		1	101793897
BP	GO:0099132	ATP hydrolysis coupled cation transmembrane transport	1/329	13/3792	0.693279	0.999979	101793897	ATP6V1E1	1	0		1	101793897
BP	GO:0030029	actin filament-based process	2/329	27/3792	0.693546	0.999979	101799876/101797044	LOC101799876/LMOD2	2	1	101799876	1	101797044
BP	GO:0030036	actin cytoskeleton organization	2/329	27/3792	0.693546	0.999979	101799876/101797044	LOC101799876/LMOD2	2	1	101799876	1	101797044
BP	GO:0051128	regulation of cellular component organization	2/329	27/3792	0.693546	0.999979	101797044/101795780	LMOD2/MAD1L1	2	1	101795780	1	101797044
BP	GO:0006323	DNA packaging	1/329	14/3792	0.719982	0.999979	101798499	LOC101798499	1	0		1	101798499
BP	GO:0006333	chromatin assembly or disassembly	1/329	14/3792	0.719982	0.999979	101798499	LOC101798499	1	0		1	101798499
BP	GO:0006790	sulfur compound metabolic process	1/329	14/3792	0.719982	0.999979	101799171	MAT1A	1	1	101799171	0	
BP	GO:0008064	regulation of actin polymerization or depolymerization	1/329	14/3792	0.719982	0.999979	101797044	LMOD2	1	0		1	101797044
BP	GO:0008154	actin polymerization or depolymerization	1/329	14/3792	0.719982	0.999979	101797044	LMOD2	1	0		1	101797044
BP	GO:0030041	actin filament polymerization	1/329	14/3792	0.719982	0.999979	101797044	LMOD2	1	0		1	101797044
BP	GO:0030832	regulation of actin filament length	1/329	14/3792	0.719982	0.999979	101797044	LMOD2	1	0		1	101797044
BP	GO:0030833	regulation of actin filament polymerization	1/329	14/3792	0.719982	0.999979	101797044	LMOD2	1	0		1	101797044
BP	GO:0032271	regulation of protein polymerization	1/329	14/3792	0.719982	0.999979	101797044	LMOD2	1	0		1	101797044
BP	GO:0032535	regulation of cellular component size	1/329	14/3792	0.719982	0.999979	101797044	LMOD2	1	0		1	101797044
BP	GO:0032956	regulation of actin cytoskeleton organization	1/329	14/3792	0.719982	0.999979	101797044	LMOD2	1	0		1	101797044
BP	GO:0032970	regulation of actin filament-based process	1/329	14/3792	0.719982	0.999979	101797044	LMOD2	1	0		1	101797044
BP	GO:0032984	protein-containing complex disassembly	1/329	14/3792	0.719982	0.999979	101797044	LMOD2	1	0		1	101797044
BP	GO:0034728	nucleosome organization	1/329	14/3792	0.719982	0.999979	101798499	LOC101798499	1	0		1	101798499
BP	GO:0043087	regulation of GTPase activity	1/329	14/3792	0.719982	0.999979	101795631	LOC101795631	1	0		1	101795631
BP	GO:0043254	regulation of protein complex assembly	1/329	14/3792	0.719982	0.999979	101797044	LMOD2	1	0		1	101797044
BP	GO:0043624	cellular protein complex disassembly	1/329	14/3792	0.719982	0.999979	101797044	LMOD2	1	0		1	101797044

BP	GO:0044242	cellular lipid catabolic process	1/329	14/3792	0.719982	0.999979	101804343	ACOX2	1	1	101804343	0	
BP	GO:0110053	regulation of actin filament organization	1/329	14/3792	0.719982	0.999979	101797044	LMOD2	1	0		1	101797044
BP	GO:1902903	regulation of supramolecular fiber organization	1/329	14/3792	0.719982	0.999979	101797044	LMOD2	1	0		1	101797044
BP	GO:0010605	negative regulation of macromolecule metabolic process	1/329	15/3792	0.744367	0.999979	101798286	EIF4EBP1	1	0		1	101798286
BP	GO:0022411	cellular component disassembly	1/329	15/3792	0.744367	0.999979	101797044	LMOD2	1	0		1	101797044
BP	GO:0044087	regulation of cellular component biogenesis	1/329	15/3792	0.744367	0.999979	101797044	LMOD2	1	0		1	101797044
BP	GO:0045017	glycerolipid biosynthetic process	1/329	15/3792	0.744367	0.999979	101804047	PTDSS1	1	1	101804047	0	
BP	GO:0046474	glycerophospholipid biosynthetic process	1/329	15/3792	0.744367	0.999979	101804047	PTDSS1	1	1	101804047	0	
BP	GO:0007186	G-protein-coupled receptor signaling pathway	19/329	249/3792	0.760957	0.999979	106020638/101789607/101789648/101796159/101803645/101804355/101793595/101802295/101803152/101802610/101797638/101793610/101790005/101803029/101794938/101801352/101801143/101804306/101801041	APLNR/HTR5A/GPR157/LOC101796159/ADGRL3/GPR176/LPAR3/UTS2R/GPR161/CCR9/LOC101797638/GPR182/LOC101790005/CELSR1/ADGRD1/CNR1/DGKI/PTGFR/VIPR2	19	14	106020638/101789607/101796159/101803645/101804355/101793595/101802295/101803152/101797638/101793610/101803029/101801352/101801143/101804306	5	101789648/101802610/101790005/101794938/101801041
BP	GO:0007015	actin filament organization	1/329	16/3792	0.766634	0.999979	101797044	LMOD2	1	0		1	101797044
BP	GO:0016567	protein ubiquitination	1/329	16/3792	0.766634	0.999979	101802075	MIB1	1	1	101802075	0	
BP	GO:0097435	supramolecular fiber organization	1/329	16/3792	0.766634	0.999979	101797044	LMOD2	1	0		1	101797044
BP	GO:0051056	regulation of small GTPase mediated signal transduction	4/329	60/3792	0.777725	0.999979	101795713/101792952/101790388/101792679	KALRN/ARHGEF3/ARHGEF17/PLEKHG1	4	3	101792952/101790388/101792679	1	101795713
BP	GO:0009892	negative regulation of metabolic process	1/329	17/3792	0.786967	0.999979	101798286	EIF4EBP1	1	0		1	101798286
BP	GO:0032446	protein modification by small protein conjugation	1/329	17/3792	0.786967	0.999979	101802075	MIB1	1	1	101802075	0	
BP	GO:0048193	Golgi vesicle transport	1/329	17/3792	0.786967	0.999979	101792755	LOC101792755	1	0		1	101792755
BP	GO:0051258	protein polymerization	1/329	17/3792	0.786967	0.999979	101797044	LMOD2	1	0		1	101797044
BP	GO:0090066	regulation of anatomical structure size	1/329	17/3792	0.786967	0.999979	101797044	LMOD2	1	0		1	101797044
BP	GO:1901565	organonitrogen compound catabolic process	3/329	47/3792	0.788019	0.999979	101799480/101805057/101803700	UCHL1/HMOX1/PSMB1	3	0		3	101799480/101805057/101803700
BP	GO:1902531	regulation of intracellular signal transduction	4/329	63/3792	0.809491	0.999979	101795713/101792952/101790388/101792679	KALRN/ARHGEF3/ARHGEF17/PLEKHG1	4	3	101792952/101790388/101792679	1	101795713
BP	GO:0007156	homophilic cell adhesion via plasma membrane adhesion molecules	5/329	78/3792	0.820045	0.999979	101803002/101803029/101789534/101800667/101796230	PCDH7/CELSR1/CDHR5/CDH2/CDH8	5	3	101803002/101803029/101796230	2	101789534/101800667
BP	GO:0098609	cell-cell adhesion	5/329	78/3792	0.820045	0.999979	101803002/101803029/101789534/101800667/101796230	PCDH7/CELSR1/CDHR5/CDH2/CDH8	5	3	101803002/101803029/101796230	2	101789534/101800667
BP	GO:0098742	cell-cell adhesion via plasma-membrane adhesion molecules	5/329	78/3792	0.820045	0.999979	101803002/101803029/101789534/101800667/101796230	PCDH7/CELSR1/CDHR5/CDH2/CDH8	5	3	101803002/101803029/101796230	2	101789534/101800667
BP	GO:0006418	tRNA aminoacylation for protein translation	2/329	35/3792	0.820845	0.999979	119714249/101795091	LOC119714249/TARS3	2	2	119714249/101795091	0	
BP	GO:0044257	cellular protein catabolic process	2/329	35/3792	0.820845	0.999979	101799480/101803700	UCHL1/PSMB1	2	0		2	101799480/101803700
BP	GO:0051603	proteolysis involved in cellular protein catabolic process	2/329	35/3792	0.820845	0.999979	101799480/101803700	UCHL1/PSMB1	2	0		2	101799480/101803700
BP	GO:0051336	regulation of hydrolase activity	1/329	19/3792	0.822486	0.999979	101795631	LOC101795631	1	0		1	101795631
BP	GO:0065004	protein-DNA complex assembly	1/329	19/3792	0.822486	0.999979	101798499	LOC101798499	1	0		1	101798499
BP	GO:0007155	cell adhesion	8/329	119/3792	0.824035	0.999979	101800985/101793322/101798168/101803002/101803029/101789534/101800667/101796230	LAMA4/THBS1/TNFAIP6/PCDH7/CELSR1/CDHR5/CDH2/CDH8	8	4	101800985/101803002/101803029/101796230	4	101793322/101798168/101789534/101800667

BP	GO:0022610	biological adhesion	8/329	119/3792	0.824035	0.999979	101800985/101793322/101798168/101803002/101803029/101789534/101800667/101796230	LAMA4/THBS1/TNFAIP6/PCDH7/CELSR1/CDHR5/CDH2/CDH8	8	4	101800985/101803002/101803029/101796230	4	101793322/101798168/101789534/101800667
BP	GO:0033036	macromolecule localization	8/329	119/3792	0.824035	0.999979	101802251/101804872/101797331/101792755/101793444/101798726/101801245/101798131	PITPNM1/LOC101804872/APOA4/LOC101792755/AKAP12/APOLD1/SELENOS/PITPNC1	8	3	101797331/101793444/101798131	5	101802251/101804872/101792755/101798726/101801245
BP	GO:0051493	regulation of cytoskeleton organization	1/329	20/3792	0.837965	0.999979	101797044	LMOD2	1	0		1	101797044
BP	GO:0071824	protein-DNA complex subunit organization	1/329	20/3792	0.837965	0.999979	101798499	LOC101798499	1	0		1	101798499
BP	GO:0006260	DNA replication	2/329	37/3792	0.844214	0.999979	101790058/101797382	BLM/POLD3	2	2	101790058/101797382	0	
BP	GO:0030163	protein catabolic process	2/329	37/3792	0.844214	0.999979	101799480/101803700	UCHL1/PSMB1	2	0		2	101799480/101803700
BP	GO:0000413	protein peptidyl-prolyl isomerization	1/329	21/3792	0.852098	0.999979	101802609	PPIF	1	1	101802609	0	
BP	GO:0018208	peptidyl-proline modification	1/329	21/3792	0.852098	0.999979	101802609	PPIF	1	1	101802609	0	
BP	GO:0071103	DNA conformation change	1/329	21/3792	0.852098	0.999979	101798499	LOC101798499	1	0		1	101798499
BP	GO:0043038	amino acid activation	2/329	38/3792	0.854832	0.999979	119714249/101795091	LOC119714249/TARS3	2	2	119714249/101795091	0	
BP	GO:0043039	tRNA aminoacylation	2/329	38/3792	0.854832	0.999979	119714249/101795091	LOC119714249/TARS3	2	2	119714249/101795091	0	
BP	GO:0016579	protein deubiquitination	2/329	39/3792	0.864788	0.999979	101792575/101791234	USP44/USP49	2	0		2	101792575/101791234
BP	GO:0070646	protein modification by small protein removal	2/329	39/3792	0.864788	0.999979	101792575/101791234	USP44/USP49	2	0		2	101792575/101791234
BP	GO:0008654	phospholipid biosynthetic process	1/329	22/3792	0.865002	0.999979	101804047	PTDSS1	1	1	101804047	0	
BP	GO:0051171	regulation of nitrogen compound metabolic process	25/329	348/3792	0.874045	0.999979	101805187/101798286/101794377/101799581/101799370/101801567/101790639/119715483/106019185/101801755/101801698/101795336/101794314/119714375/101797962/101802147/101792591/101789522/101789572/101796626/101801576/101795523/113845655/101791584/119714007	ATF3/EIF4EBP1/MAFK/LOC101799581/LOC101799370/TAF1D/CREM/LOC119715483/CEBPB/MYF5/FOS/SHLD1/NFIL3/LOC119714375/CREB5/L3MBTL3/LRRFIP1/MYOD1/PPARA/JUN/E2F1/MEIS1/JUND/SMAD6/LOC119714007	25	7	101799370/119715483/119714375/101789572/101801576/101795523/119714007	18	101805187/101798286/101794377/101799581/101801567/101790639/106019185/101801755/101801698/101795336/101794314/101797962/101802147/101792591/101789522/101796626/113845655/101791584
BP	GO:0080090	regulation of primary metabolic process	25/329	348/3792	0.874045	0.999979	101805187/101798286/101794377/101799581/101799370/101801567/101790639/119715483/106019185/101801755/101801698/101795336/101794314/119714375/101797962/101802147/101792591/101789522/101789572/101796626/101801576/101795523/113845655/101791584/119714007	ATF3/EIF4EBP1/MAFK/LOC101799581/LOC101799370/TAF1D/CREM/LOC119715483/CEBPB/MYF5/FOS/SHLD1/NFIL3/LOC119714375/CREB5/L3MBTL3/LRRFIP1/MYOD1/PPARA/JUN/E2F1/MEIS1/JUND/SMAD6/LOC119714007	25	7	101799370/119715483/119714375/101789572/101801576/101795523/119714007	18	101805187/101798286/101794377/101799581/101801567/101790639/106019185/101801755/101801698/101795336/101794314/101797962/101802147/101792591/101789522/101796626/113845655/101791584
BP	GO:0044265	cellular macromolecule catabolic process	2/329	40/3792	0.874115	0.999979	101799480/101803700	UCHL1/PSMB1	2	0		2	101799480/101803700

BP	GO:0019219	regulation of nucleobase-containing compound metabolic process	24/329	336/3792	0.876221	0.999979	101805187/101794377/101799581/101799370/101801567/101790639/119715483/106019185/101801755/101801698/101795336/101794314/119714375/101797962/101802147/101792591/101789522/101789572/101796626/101801576/101795523/113845655/101791584/119714007	ATF3/MAFK/LOC101799581/LOC101799370/TAF1D/CREM/LOC119715483/CEBPB/MYF5/FOS/SHLD1/NFIL3/LOC119714375/CREB5/L3MBTL3/LRRFIP1/MYOD1/PPARA/JUN/E2F1/MEIS1/JUND/SMAD6/LOC119714007	24	7	101799370/119715483/119714375/101789572/101801576/101795523/119714007	17	101805187/101794377/101799581/101801567/101790639/106019185/101801755/101801698/101795336/101794314/101797962/101802147/101792591/101789522/101796626/113845655/101791584
BP	GO:0070647	protein modification by small protein conjugation or removal	3/329	56/3792	0.876414	0.999979	101792575/101791234/101802075	USP44/USP49/MIB1	3	1	101802075	2	101792575/101791234
BP	GO:0010556	regulation of macromolecule biosynthetic process	24/329	339/3792	0.886321	0.999979	101805187/101798286/101794377/101799581/101799370/101801567/101790639/119715483/106019185/101801755/101801698/101794314/119714375/101797962/101802147/101792591/101789522/101789572/101796626/101801576/101795523/113845655/101791584/119714007	ATF3/EIF4EBP1/MAFK/LOC101799581/LOC101799370/TAF1D/CREM/LOC119715483/CEBPB/MYF5/FOS/NFIL3/LOC119714375/CREB5/L3MBTL3/LRRFIP1/MYOD1/PPARA/JUN/E2F1/MEIS1/JUND/SMAD6/LOC119714007	24	7	101799370/119715483/119714375/101789572/101801576/101795523/119714007	17	101805187/101798286/101794377/101799581/101801567/101790639/106019185/101801755/101801698/101794314/101797962/101802147/101792591/101789522/101796626/113845655/101791584
BP	GO:0031326	regulation of cellular biosynthetic process	24/329	339/3792	0.886321	0.999979	101805187/101798286/101794377/101799581/101799370/101801567/101790639/119715483/106019185/101801755/101801698/101794314/119714375/101797962/101802147/101792591/101789522/101789572/101796626/101801576/101795523/113845655/101791584/119714007	ATF3/EIF4EBP1/MAFK/LOC101799581/LOC101799370/TAF1D/CREM/LOC119715483/CEBPB/MYF5/FOS/NFIL3/LOC119714375/CREB5/L3MBTL3/LRRFIP1/MYOD1/PPARA/JUN/E2F1/MEIS1/JUND/SMAD6/LOC119714007	24	7	101799370/119715483/119714375/101789572/101801576/101795523/119714007	17	101805187/101798286/101794377/101799581/101801567/101790639/106019185/101801755/101801698/101794314/101797962/101802147/101792591/101789522/101796626/113845655/101791584
BP	GO:2000112	regulation of cellular macromolecule biosynthetic process	24/329	339/3792	0.886321	0.999979	101805187/101798286/101794377/101799581/101799370/101801567/101790639/119715483/106019185/101801755/101801698/101794314/119714375/101797962/101802147/101792591/101789522/101789572/101796626/101801576/101795523/113845655/101791584/119714007	ATF3/EIF4EBP1/MAFK/LOC101799581/LOC101799370/TAF1D/CREM/LOC119715483/CEBPB/MYF5/FOS/NFIL3/LOC119714375/CREB5/L3MBTL3/LRRFIP1/MYOD1/PPARA/JUN/E2F1/MEIS1/JUND/SMAD6/LOC119714007	24	7	101799370/119715483/119714375/101789572/101801576/101795523/119714007	17	101805187/101798286/101794377/101799581/101801567/101790639/106019185/101801755/101801698/101794314/101797962/101802147/101792591/101789522/101796626/113845655/101791584
BP	GO:0006357	regulation of transcription by RNA polymerase II	1/329	24/3792	0.887539	0.999979	101799581	LOC101799581	1	0		1	101799581
BP	GO:0009889	regulation of biosynthetic process	24/329	340/3792	0.889541	0.999979	101805187/101798286/101794377/101799581/101799370/101801567/101790639/119715483/106019185/101801755/101801698/101794314/119714375/101797962/101802147/101792591/101789522/101789572/101796626/101801576/101795523/113845655/101791584/119714007	ATF3/EIF4EBP1/MAFK/LOC101799581/LOC101799370/TAF1D/CREM/LOC119715483/CEBPB/MYF5/FOS/NFIL3/LOC119714375/CREB5/L3MBTL3/LRRFIP1/MYOD1/PPARA/JUN/E2F1/MEIS1/JUND/SMAD6/LOC119714007	24	7	101799370/119715483/119714375/101789572/101801576/101795523/119714007	17	101805187/101798286/101794377/101799581/101801567/101790639/106019185/101801755/101801698/101794314/101797962/101802147/101792591/101789522/101796626/113845655/101791584

BP	GO:0031323	regulation of cellular metabolic process	25/329	353/3792	0.890496	0.999979	101805187/101798286/101794377/101799581/101799370/101801567/101790639/119715483/106019185/101801755/101801698/101795336/101794314/119714375/101797962/101802147/101792591/101789522/101789572/101796626/101801576/101795523/113845655/101791584/119714007	ATF3/EIF4EBP1/MAFK/LOC101799581/LOC101799370/TAF1D/CREM/LOC119715483/CEBPB/MYF5/FOS/SHLD1/NFIL3/LOC119714375/CREB5/L3MBTL3/LRRFIP1/MYOD1/PPARA/JUN/E2F1/MEIS1/JUND/SMAD6/LOC119714007	25	7	101799370/119715483/119714375/101789572/101801576/101795523/119714007	18	101805187/101798286/101794377/101799581/101801567/101790639/106019185/101801755/101801698/101795336/101794314/101797962/101802147/101792591/101789522/101796626/113845655/101791584
BP	GO:0060255	regulation of macromolecule metabolic process	25/329	353/3792	0.890496	0.999979	101805187/101798286/101794377/101799581/101799370/101801567/101790639/119715483/106019185/101801755/101801698/101795336/101794314/119714375/101797962/101802147/101792591/101789522/101789572/101796626/101801576/101795523/113845655/101791584/119714007	ATF3/EIF4EBP1/MAFK/LOC101799581/LOC101799370/TAF1D/CREM/LOC119715483/CEBPB/MYF5/FOS/SHLD1/NFIL3/LOC119714375/CREB5/L3MBTL3/LRRFIP1/MYOD1/PPARA/JUN/E2F1/MEIS1/JUND/SMAD6/LOC119714007	25	7	101799370/119715483/119714375/101789572/101801576/101795523/119714007	18	101805187/101798286/101794377/101799581/101801567/101790639/106019185/101801755/101801698/101795336/101794314/101797962/101802147/101792591/101789522/101796626/113845655/101791584
BP	GO:0010468	regulation of gene expression	24/329	341/3792	0.892691	0.999979	101805187/101798286/101794377/101799581/101799370/101801567/101790639/119715483/106019185/101801755/101801698/101794314/119714375/101797962/101802147/101792591/101789522/101789572/101796626/101801576/101795523/113845655/101791584/119714007	ATF3/EIF4EBP1/MAFK/LOC101799581/LOC101799370/TAF1D/CREM/LOC119715483/CEBPB/MYF5/FOS/NFIL3/LOC119714375/CREB5/L3MBTL3/LRRFIP1/MYOD1/PPARA/JUN/E2F1/MEIS1/JUND/SMAD6/LOC119714007	24	7	101799370/119715483/119714375/101789572/101801576/101795523/119714007	17	101805187/101798286/101794377/101799581/101801567/101790639/106019185/101801755/101801698/101794314/101797962/101802147/101792591/101789522/101796626/113845655/101791584
BP	GO:0006259	DNA metabolic process	6/329	103/3792	0.894536	0.999979	101795336/101790058/101796914/101802532/101797870/101797382	SHLD1/BLM/LIG1/MSH2/XRCC5/POLD3	6	3	101790058/101797870/101797382	3	101795336/101796914/101802532
BP	GO:0018130	heterocycle biosynthetic process	34/329	469/3792	0.89839	0.999979	101805187/101794377/101799581/101799370/101801567/101790639/119715483/106019185/101801755/101801928/101801698/101803512/101794314/101792149/119714375/101797962/101802147/101792591/101790726/101789522/101803472/101796516/101789572/101796626/119714948/110351624/101801576/101795523/113845655/101797601/101800658/101791584/101790402/119714007	ATF3/MAFK/LOC101799581/LOC101799370/TAF1D/CREM/LOC119715483/CEBPB/MYF5/ADSS1/FOS/ADCY2/NFIL3/POLR2B/LOC119714375/CREB5/L3MBTL3/LRRFIP1/PKM/MYOD1/ITPA/LOC101796516/PPARA/JUN/LOC119714948/LOC110351624/E2F1/MEIS1/JUND/GPI/PCBD1/SMAD6/ATP5PO/LOC119714007	34	16	101799370/119715483/101801928/101803512/101792149/119714375/101790726/101796516/101789572/119714948/110351624/101801576/101795523/101797601/101790402/119714007	18	101805187/101794377/101799581/101801567/101790639/106019185/101801755/101801698/101794314/101797962/101802147/101792591/101789522/101803472/101796626/113845655/101800658/101791584
BP	GO:0009057	macromolecule catabolic process	2/329	43/3792	0.898659	0.999979	101799480/101803700	UCHL1/PSMB1	2	0		2	101799480/101803700

BP	GO:0006355	regulation of transcription, DNA-templated	23/329	332/3792	0.904033	0.999979	101805187/101794377/101799581/101799370/101801567/101790639/119715483/106019185/101801755/101801698/101794314/119714375/101797962/101802147/101792591/101789522/101789572/101796626/101801576/101795523/113845655/101791584/119714007	ATF3/MAFK/LOC101799581/LOC101799370/TAF1D/CREM/LOC119715483/CEBPB/MYF5/FOS/NFIL3/LOC119714375/CREB5/L3MBTL3/LRRFIP1/MYOD1/PPARA/JUN/E2F1/MEIS1/JUND/SMAD6/LOC119714007	23	7	101799370/119715483/119714375/101789572/101801576/101795523/119714007	16	101805187/101794377/101799581/101801567/101790639/106019185/101801755/101801698/101794314/101797962/101802147/101792591/101789522/101796626/113845655/101791584
BP	GO:0051252	regulation of RNA metabolic process	23/329	332/3792	0.904033	0.999979	101805187/101794377/101799581/101799370/101801567/101790639/119715483/106019185/101801755/101801698/101794314/119714375/101797962/101802147/101792591/101789522/101789572/101796626/101801576/101795523/113845655/101791584/119714007	ATF3/MAFK/LOC101799581/LOC101799370/TAF1D/CREM/LOC119715483/CEBPB/MYF5/FOS/NFIL3/LOC119714375/CREB5/L3MBTL3/LRRFIP1/MYOD1/PPARA/JUN/E2F1/MEIS1/JUND/SMAD6/LOC119714007	23	7	101799370/119715483/119714375/101789572/101801576/101795523/119714007	16	101805187/101794377/101799581/101801567/101790639/106019185/101801755/101801698/101794314/101797962/101802147/101792591/101789522/101796626/113845655/101791584
BP	GO:1903506	regulation of nucleic acid-templated transcription	23/329	332/3792	0.904033	0.999979	101805187/101794377/101799581/101799370/101801567/101790639/119715483/106019185/101801755/101801698/101794314/119714375/101797962/101802147/101792591/101789522/101789572/101796626/101801576/101795523/113845655/101791584/119714007	ATF3/MAFK/LOC101799581/LOC101799370/TAF1D/CREM/LOC119715483/CEBPB/MYF5/FOS/NFIL3/LOC119714375/CREB5/L3MBTL3/LRRFIP1/MYOD1/PPARA/JUN/E2F1/MEIS1/JUND/SMAD6/LOC119714007	23	7	101799370/119715483/119714375/101789572/101801576/101795523/119714007	16	101805187/101794377/101799581/101801567/101790639/106019185/101801755/101801698/101794314/101797962/101802147/101792591/101789522/101796626/113845655/101791584
BP	GO:2001141	regulation of RNA biosynthetic process	23/329	332/3792	0.904033	0.999979	101805187/101794377/101799581/101799370/101801567/101790639/119715483/106019185/101801755/101801698/101794314/119714375/101797962/101802147/101792591/101789522/101789572/101796626/101801576/101795523/113845655/101791584/119714007	ATF3/MAFK/LOC101799581/LOC101799370/TAF1D/CREM/LOC119715483/CEBPB/MYF5/FOS/NFIL3/LOC119714375/CREB5/L3MBTL3/LRRFIP1/MYOD1/PPARA/JUN/E2F1/MEIS1/JUND/SMAD6/LOC119714007	23	7	101799370/119715483/119714375/101789572/101801576/101795523/119714007	16	101805187/101794377/101799581/101801567/101790639/106019185/101801755/101801698/101794314/101797962/101802147/101792591/101789522/101796626/113845655/101791584
BP	GO:0046903	secretion	1/329	26/3792	0.906323	0.999979	101804872	LOC101804872	1	0		1	101804872
BP	GO:0019222	regulation of metabolic process	25/329	360/3792	0.910655	0.999979	101805187/101798286/101794377/101799581/101799370/101801567/101790639/119715483/106019185/101801755/101801698/101795336/101794314/119714375/101797962/101802147/101792591/101789522/101789572/101796626/101801576/101795523/113845655/101791584/119714007	ATF3/EIF4EBP1/MAFK/LOC101799581/LOC101799370/TAF1D/CREM/LOC119715483/CEBPB/MYF5/FOS/SHLD1/NFIL3/LOC119714375/CREB5/L3MBTL3/LRRFIP1/MYOD1/PPARA/JUN/E2F1/MEIS1/JUND/SMAD6/LOC119714007	25	7	101799370/119715483/119714375/101789572/101801576/101795523/119714007	18	101805187/101798286/101794377/101799581/101801567/101790639/106019185/101801755/101801698/101795336/101794314/101797962/101802147/101792591/101789522/101796626/113845655/101791584

BP	GO:1901362	organic cyclic compound biosynthetic process	34/329	475/3792	0.91318	0.999979	101805187/101794377/101799581/101799370/101801567/101790639/119715483/106019185/101801755/101801928/101801698/101803512/101794314/101792149/119714375/101797962/101802147/101792591/101790726/101789522/101803472/101796516/101789572/101796626/119714948/110351624/101801576/101795523/113845655/101797601/101800658/101791584/101790402/119714007	ATF3/MAFK/LOC101799581/LOC101799370/TAF1D/CREM/LOC119715483/CEBPB/MYF5/ADSS1/FOS/ADCY2/NFIL3/POLR2B/LOC119714375/CREB5/L3MBTL3/LRRFIP1/PKM/MYOD1/ITPA/LOC101796516/PPARA/JUN/LOC119714948/LOC110351624/E2F1/MEIS1/JUND/GPI/PCBD1/SMAD6/ATP5PO/LOC119714007	34	16	101799370/119715483/101801928/101803512/101792149/119714375/101790726/101796516/101789572/119714948/110351624/101801576/101795523/101797601/101790402/119714007	18	101805187/101794377/101799581/101801567/101790639/106019185/101801755/101801698/101794314/101797962/101802147/101792591/101789522/101803472/101796626/113845655/101800658/101791584
BP	GO:0019438	aromatic compound biosynthetic process	33/329	464/3792	0.916785	0.999979	101805187/101794377/101799581/101799370/101801567/101790639/119715483/106019185/101801755/101801928/101801698/101803512/101794314/101792149/119714375/101797962/101802147/101792591/101790726/101789522/101803472/101796516/101789572/101796626/119714948/101801576/101795523/113845655/101797601/101800658/101791584/101790402/119714007	ATF3/MAFK/LOC101799581/LOC101799370/TAF1D/CREM/LOC119715483/CEBPB/MYF5/ADSS1/FOS/ADCY2/NFIL3/POLR2B/LOC119714375/CREB5/L3MBTL3/LRRFIP1/PKM/MYOD1/ITPA/LOC101796516/PPARA/JUN/LOC119714948/E2F1/MEIS1/JUND/GPI/PCBD1/SMAD6/ATP5PO/LOC119714007	33	15	101799370/119715483/101801928/101803512/101792149/119714375/101790726/101796516/101789572/119714948/101801576/101795523/101797601/101790402/119714007	18	101805187/101794377/101799581/101801567/101790639/106019185/101801755/101801698/101794314/101797962/101802147/101792591/101789522/101803472/101796626/113845655/101800658/101791584
BP	GO:0034654	nucleobase-containing compound biosynthetic process	32/329	452/3792	0.918166	0.999979	101805187/101794377/101799581/101799370/101801567/101790639/119715483/106019185/101801755/101801928/101801698/101803512/101794314/101792149/119714375/101797962/101802147/101792591/101790726/101789522/101803472/101796516/101789572/101796626/119714948/101801576/101795523/113845655/101797601/101791584/101790402/119714007	ATF3/MAFK/LOC101799581/LOC101799370/TAF1D/CREM/LOC119715483/CEBPB/MYF5/ADSS1/FOS/ADCY2/NFIL3/POLR2B/LOC119714375/CREB5/L3MBTL3/LRRFIP1/PKM/MYOD1/ITPA/LOC101796516/PPARA/JUN/LOC119714948/E2F1/MEIS1/JUND/GPI/SMAD6/ATP5PO/LOC119714007	32	15	101799370/119715483/101801928/101803512/101792149/119714375/101790726/101796516/101789572/119714948/101801576/101795523/101797601/101790402/119714007	17	101805187/101794377/101799581/101801567/101790639/106019185/101801755/101801698/101794314/101797962/101802147/101792591/101789522/101803472/101796626/113845655/101791584
BP	GO:0007264	small GTPase mediated signal transduction	4/329	79/3792	0.92205	0.999979	101795713/101792952/101790388/101792679	KALRN/ARHGEF3/ARHGEF17/PLEKHG1	4	3	101792952/101790388/101792679	1	101795713
BP	GO:0006511	ubiquitin-dependent protein catabolic process	1/329	30/3792	0.935022	0.999979	101799480	UCHL1	1	0		1	101799480
BP	GO:0019941	modification-dependent protein catabolic process	1/329	30/3792	0.935022	0.999979	101799480	UCHL1	1	0		1	101799480
BP	GO:0043632	modification-dependent macromolecule catabolic process	1/329	30/3792	0.935022	0.999979	101799480	UCHL1	1	0		1	101799480
BP	GO:0006325	chromatin organization	1/329	32/3792	0.945892	0.999979	101798499	LOC101798499	1	0		1	101798499
BP	GO:0006397	mRNA processing	1/329	33/3792	0.950626	0.999979	101800595	RNASEL	1	1	101800595	0	
BP	GO:0045454	cell redox homeostasis	1/329	33/3792	0.950626	0.999979	101804092	TXN	1	0		1	101804092
BP	GO:0051260	protein homooligomerization	1/329	33/3792	0.950626	0.999979	119713969	KCNC4	1	0		1	119713969

BP	GO:0007010	cytoskeleton organization	2/329	55/3792	0.958724	0.999979	101799876/101797044	LOC101799876/LMOD2	2	1	101799876	1	101797044
BP	GO:0006366	transcription by RNA polymerase II	1/329	37/3792	0.965777	0.999979	101799581	LOC101799581	1	0		1	101799581
BP	GO:0019725	cellular homeostasis	1/329	37/3792	0.965777	0.999979	101804092	TXN	1	0		1	101804092
BP	GO:0006886	intracellular protein transport	3/329	76/3792	0.966958	0.999979	101792755/101793444/101801245	LOC101792755/AKAP12/SELENOS	3	1	101793444	2	101792755/101801245
BP	GO:0034622	cellular protein-containing complex assembly	2/329	58/3792	0.967235	0.999979	101798499/101797044	LOC101798499/LMOD2	2	0		2	101798499/101797044
BP	GO:0016071	mRNA metabolic process	1/329	38/3792	0.968775	0.999979	101800595	RNASEL	1	1	101800595	0	
BP	GO:0051276	chromosome organization	2/329	59/3792	0.969677	0.999979	101798499/101795780	LOC101798499/MAD1L1	2	1	101795780	1	101798499
BP	GO:0043604	amide biosynthetic process	7/329	143/3792	0.971483	0.999979	101798286/119714249/101802140/101797455/101793553/101795091/106017870	EIF4EBP1/LOC119714249/LOC101802140/ASNS/EIF1/TARS3/MRPL2	7	4	119714249/101802140/101795091/106017870	3	101798286/101797455/101793553
BP	GO:0018193	peptidyl-amino acid modification	1/329	39/3792	0.971512	0.999979	101802609	PPIF	1	1	101802609	0	
BP	GO:0051259	protein complex oligomerization	1/329	40/3792	0.974009	0.999979	119713969	KCNC4	1	0		1	119713969
BP	GO:0006399	tRNA metabolic process	2/329	61/3792	0.974047	0.999979	119714249/101795091	LOC119714249/TARS3	2	2	119714249/101795091	0	
BP	GO:0042592	homeostatic process	1/329	41/3792	0.976288	0.999979	101804092	TXN	1	0		1	101804092
BP	GO:0008610	lipid biosynthetic process	1/329	42/3792	0.978368	0.999979	101804047	PTDSS1	1	1	101804047	0	
BP	GO:0043603	cellular amide metabolic process	7/329	149/3792	0.979509	0.999979	101798286/119714249/101802140/101797455/101793553/101795091/106017870	EIF4EBP1/LOC119714249/LOC101802140/ASNS/EIF1/TARS3/MRPL2	7	4	119714249/101802140/101795091/106017870	3	101798286/101797455/101793553
BP	GO:0034613	cellular protein localization	3/329	83/3792	0.979784	0.999979	101792755/101793444/101801245	LOC101792755/AKAP12/SELENOS	3	1	101793444	2	101792755/101801245
BP	GO:0070727	cellular macromolecule localization	3/329	83/3792	0.979784	0.999979	101792755/101793444/101801245	LOC101792755/AKAP12/SELENOS	3	1	101793444	2	101792755/101801245
BP	GO:0006351	transcription, DNA-templated	24/329	393/3792	0.981256	0.999979	101805187/101794377/101799581/101799370/101801567/101790639/119715483/106019185/101801755/101801698/101794314/101792149/119714375/101797962/101802147/101792591/101789522/101789572/101796626/101801576/101795523/113845655/101791584/119714007	ATF3/MAFK/LOC101799581/LOC101799370/TAF1D/CREM/LOC119715483/CEBPB/MYF5/FOS/NFIL3/POLR2B/LOC119714375/CREB5/L3MBTL3/LRRFIP1/MYOD1/PPARA/JUN/E2F1/MEIS1/JUND/SMAD6/LOC119714007	24	8	101799370/119715483/101792149/119714375/101789572/101801576/101795523/119714007	16	101805187/101794377/101799581/101801567/101790639/106019185/101801755/101801698/101794314/101797962/101802147/101792591/101789522/101796626/113845655/101791584
BP	GO:0097659	nucleic acid-templated transcription	24/329	393/3792	0.981256	0.999979	101805187/101794377/101799581/101799370/101801567/101790639/119715483/106019185/101801755/101801698/101794314/101792149/119714375/101797962/101802147/101792591/101789522/101789572/101796626/101801576/101795523/113845655/101791584/119714007	ATF3/MAFK/LOC101799581/LOC101799370/TAF1D/CREM/LOC119715483/CEBPB/MYF5/FOS/NFIL3/POLR2B/LOC119714375/CREB5/L3MBTL3/LRRFIP1/MYOD1/PPARA/JUN/E2F1/MEIS1/JUND/SMAD6/LOC119714007	24	8	101799370/119715483/101792149/119714375/101789572/101801576/101795523/119714007	16	101805187/101794377/101799581/101801567/101790639/106019185/101801755/101801698/101794314/101797962/101802147/101792591/101789522/101796626/113845655/101791584
BP	GO:0071705	nitrogen compound transport	4/329	102/3792	0.981644	0.999979	119713349/101792755/101793444/101801245	SLC52A3/LOC101792755/AKAP12/SELENOS	4	2	119713349/101793444	2	101792755/101801245

BP	GO:0032774	RNA biosynthetic process	24/329	395/3792	0.982629	0.999979	101805187/101794377/101799581/101799370/101801567/101790639/119715483/106019185/101801755/101801698/101794314/101792149/119714375/101797962/101802147/101792591/101789522/101789572/101796626/101801576/101795523/113845655/101791584/119714007	ATF3/MAFK/LOC101799581/LOC101799370/TAF1D/CREM/LOC119715483/CEBPB/MYF5/FOS/NFIL3/POLR2B/LOC119714375/CREB5/L3MBTL3/LRRFIP1/MYOD1/PPARA/JUN/E2F1/MEIS1/JUND/SMAD6/LOC119714007	24	8	101799370/119715483/101792149/119714375/101789572/101801576/101795523/119714007	16	101805187/101794377/101799581/101801567/101790639/106019185/101801755/101801698/101794314/101797962/101802147/101792591/101789522/101796626/113845655/101791584
BP	GO:0006412	translation	6/329	137/3792	0.983583	0.999979	101798286/119714249/101802140/101793553/101795091/106017870	EIF4EBP1/LOC119714249/LOC101802140/EIF1/TARS3/MRPL2	6	4	119714249/101802140/101795091/106017870	2	101798286/101793553
BP	GO:0015031	protein transport	3/329	88/3792	0.985874	0.999979	101792755/101793444/101801245	LOC101792755/AKAP12/SELENOS	3	1	101793444	2	101792755/101801245
BP	GO:0015833	peptide transport	3/329	88/3792	0.985874	0.999979	101792755/101793444/101801245	LOC101792755/AKAP12/SELENOS	3	1	101793444	2	101792755/101801245
BP	GO:0043043	peptide biosynthetic process	6/329	140/3792	0.986279	0.999979	101798286/119714249/101802140/101793553/101795091/106017870	EIF4EBP1/LOC119714249/LOC101802140/EIF1/TARS3/MRPL2	6	4	119714249/101802140/101795091/106017870	2	101798286/101793553
BP	GO:0042886	amide transport	3/329	89/3792	0.98686	0.999979	101792755/101793444/101801245	LOC101792755/AKAP12/SELENOS	3	1	101793444	2	101792755/101801245
BP	GO:0045184	establishment of protein localization	3/329	89/3792	0.98686	0.999979	101792755/101793444/101801245	LOC101792755/AKAP12/SELENOS	3	1	101793444	2	101792755/101801245
BP	GO:0065008	regulation of biological quality	2/329	70/3792	0.987249	0.999979	101797044/101804092	LMOD2/TXN	2	0		2	101797044/101804092
BP	GO:0046907	intracellular transport	3/329	90/3792	0.987781	0.999979	101792755/101793444/101801245	LOC101792755/AKAP12/SELENOS	3	1	101793444	2	101792755/101801245
BP	GO:0006518	peptide metabolic process	6/329	143/3792	0.988555	0.999979	101798286/119714249/101802140/101793553/101795091/106017870	EIF4EBP1/LOC119714249/LOC101802140/EIF1/TARS3/MRPL2	6	4	119714249/101802140/101795091/106017870	2	101798286/101793553
BP	GO:0051649	establishment of localization in cell	3/329	91/3792	0.988639	0.999979	101792755/101793444/101801245	LOC101792755/AKAP12/SELENOS	3	1	101793444	2	101792755/101801245
BP	GO:0008104	protein localization	3/329	94/3792	0.99088	0.999979	101792755/101793444/101801245	LOC101792755/AKAP12/SELENOS	3	1	101793444	2	101792755/101801245
BP	GO:0022607	cellular component assembly	4/329	113/3792	0.991261	0.999979	101798499/101792227/101797044/119713969	LOC101798499/CAV3/LMOD2/KCNC4	4	1	101792227	3	101798499/101797044/119713969
BP	GO:0034660	ncRNA metabolic process	2/329	77/3792	0.992738	0.999979	119714249/101795091	LOC119714249/TARS3	2	2	119714249/101795091	0	
BP	GO:0065003	protein-containing complex assembly	3/329	100/3792	0.994156	0.999979	101798499/101797044/119713969	LOC101798499/LMOD2/KCNC4	3	0		3	101798499/101797044/119713969
BP	GO:0006996	organelle organization	5/329	142/3792	0.995909	0.999979	101798499/101801562/101799876/101797044/101795780	LOC101798499/APOOL/LOC101799876/LMOD2/MAD1L1	5	3	101801562/101799876/101795780	2	101798499/101797044
BP	GO:0043933	protein-containing complex subunit organization	3/329	106/3792	0.99628	0.999979	101798499/101797044/119713969	LOC101798499/LMOD2/KCNC4	3	0		3	101798499/101797044/119713969
BP	GO:0051641	cellular localization	3/329	109/3792	0.997039	0.999979	101792755/101793444/101801245	LOC101792755/AKAP12/SELENOS	3	1	101793444	2	101792755/101801245
BP	GO:0016192	vesicle-mediated transport	2/329	91/3792	0.997696	0.999979	101800849/101792755	VAMP4/LOC101792755	2	0		2	101800849/101792755
BP	GO:0044085	cellular component biogenesis	4/329	137/3792	0.998412	0.999979	101798499/101792227/101797044/119713969	LOC101798499/CAV3/LMOD2/KCNC4	4	1	101792227	3	101798499/101797044/119713969
BP	GO:0006396	RNA processing	2/329	105/3792	0.999287	0.999979	101800595/101792961	RNASEL/TRNT1	2	2	101800595/101792961	0	
BP	GO:0016043	cellular component organization	7/329	230/3792	0.999883	0.999979	101798499/101792227/101801562/101799876/101797044/119713969/101795780	LOC101798499/CAV3/APOOL/LOC101799876/LMOD2/KCNC4/MAD1L1	7	4	101792227/101801562/101799876/101795780	3	101798499/101797044/119713969
BP	GO:0071840	cellular component organization or biogenesis	7/329	254/3792	0.999979	0.999979	101798499/101792227/101801562/101799876/101797044/119713969/101795780	LOC101798499/CAV3/APOOL/LOC101799876/LMOD2/KCNC4/MAD1L1	7	4	101792227/101801562/101799876/101795780	3	101798499/101797044/119713969

CC	GO:0031226	intrinsic component of plasma membrane	5/192	17/2288	0.0106	0.996835	119713349/119714808/101803486/106015345/101799054	SLC52A3/ATP1B2/LOC101803486/GPC1/SCN5A	5	5	119713349/119714808/101803486/106015345/101799054	0	
CC	GO:0005886	plasma membrane	7/192	40/2288	0.045346	0.996835	119713349/119714808/101803486/101803512/106015345/101799054/101798389	SLC52A3/ATP1B2/LOC101803486/ADCY2/GPC1/SCN5A/SGCG	7	7	119713349/119714808/101803486/101803512/106015345/101799054/101798389	0	
CC	GO:0044459	plasma membrane part	6/192	34/2288	0.059858	0.996835	119713349/119714808/101803486/106015345/101799054/101798389	SLC52A3/ATP1B2/LOC101803486/GPC1/SCN5A/SGCG	6	6	119713349/119714808/101803486/106015345/101799054/101798389	0	
CC	GO:0005576	extracellular region	19/192	158/2288	0.064554	0.996835	101803411/101794528/101791711/101790193/101795038/101799001/101793322/101803050/113839877/101803685/101793480/101797331/101793597/novel.277/101799111/101799824/101792356/101798726/101804308	IGFBP5/LOC101794528/WNT4/CCN2/CX3CL1/CCN1/THBS1/CRH/ECM1/IGFBP3/ADA2/APOA4/IGF1/-/IGF2/WNT2/WNT5B/APOLD1/MMP23B	19	8	101803411/101791711/101795038/101793480/101797331/101793597/101799111/101804308	11	101794528/101790193/101799001/101793322/101803050/113839877/101803685/novel.277/101799824/101792356/101798726
CC	GO:0005887	integral component of plasma membrane	3/192	13/2288	0.088923	0.996835	119713349/119714808/101799054	SLC52A3/ATP1B2/SCN5A	3	3	119713349/119714808/101799054	0	
CC	GO:0071944	cell periphery	7/192	50/2288	0.120504	0.996835	119713349/119714808/101803486/101803512/106015345/101799054/101798389	SLC52A3/ATP1B2/LOC101803486/ADCY2/GPC1/SCN5A/SGCG	7	7	119713349/119714808/101803486/101803512/106015345/101799054/101798389	0	
CC	GO:0030286	dynein complex	3/192	17/2288	0.165866	0.996835	101797173/101795653/101790717	DNAH5/DNAH3/DNAH9	3	3	101797173/101795653/101790717	0	
CC	GO:0005875	microtubule associated complex	3/192	18/2288	0.187473	0.996835	101797173/101795653/101790717	DNAH5/DNAH3/DNAH9	3	3	101797173/101795653/101790717	0	
CC	GO:0031300	intrinsic component of organelle membrane	2/192	11/2288	0.234304	0.996835	101801562/101789523	APOOL/CHCHD3	2	2	101801562/101789523	0	
CC	GO:0031301	integral component of organelle membrane	2/192	11/2288	0.234304	0.996835	101801562/101789523	APOOL/CHCHD3	2	2	101801562/101789523	0	
CC	GO:0005856	cytoskeleton	12/192	119/2288	0.29289	0.996835	101804531/119717442/101797173/101795653/110353953/101796751/101803548/101790717/101802165/113841953/101790909/101798589	MYO16/DES/DNAH5/DNAH3/LOC110353953/DST/TNNI1/DNAH9/LOC101802165/LOC113841953/LOC101790909/MYO5C	12	11	101804531/101797173/101795653/110353953/101796751/101803548/101790717/101802165/113841953/101790909/101798589	1	119717442
CC	GO:0044430	cytoskeletal part	10/192	100/2288	0.327281	0.996835	101804531/119717442/101797173/101795653/110353953/101803548/101790717/101802165/101790909/101798589	MYO16/DES/DNAH5/DNAH3/LOC110353953/TNNI1/DNAH9/LOC101802165/LOC101790909/MYO5C	10	9	101804531/101797173/101795653/110353953/101803548/101790717/101802165/101790909/101798589	1	119717442
CC	GO:0031012	extracellular matrix	2/192	14/2288	0.331002	0.996835	101794528/101804308	LOC101794528/MMP23B	2	1	101804308	1	101794528
CC	GO:1902495	transmembrane transporter complex	2/192	15/2288	0.36266	0.996835	119714808/101799054	ATP1B2/SCN5A	2	2	119714808/101799054	0	
CC	GO:1990351	transporter complex	2/192	15/2288	0.36266	0.996835	119714808/101799054	ATP1B2/SCN5A	2	2	119714808/101799054	0	

CC	GO:0005743	mitochondrial inner membrane	3/192	27/2288	0.39817	0.996835	101801562/101798013/101789523	APOOL/MPC1/CHCHD3	3	2	101801562/101789523	1	101798013
CC	GO:0019866	organelle inner membrane	3/192	27/2288	0.39817	0.996835	101801562/101798013/101789523	APOOL/MPC1/CHCHD3	3	2	101801562/101789523	1	101798013
CC	GO:0016459	myosin complex	5/192	50/2288	0.41119	0.996835	101804531/110353953/101802165/101790909/101798589	MYO16/LOC110353953/LOC101802165/LOC101790909/MYO5C	5	5	101804531/110353953/101802165/101790909/101798589	0	
CC	GO:0044421	extracellular region part	4/192	39/2288	0.416576	0.996835	101794528/113839877/101793480/101804308	LOC101794528/ECM1/ADA2/MMP23B	4	2	101793480/101804308	2	101794528/113839877
CC	GO:0015629	actin cytoskeleton	6/192	62/2288	0.421347	0.996835	101804531/110353953/101803548/101802165/101790909/101798589	MYO16/LOC110353953/TNNI1/LOC101802165/LOC101790909/MYO5C	6	6	101804531/110353953/101803548/101802165/101790909/101798589	0	
CC	GO:0015630	microtubule cytoskeleton	3/192	28/2288	0.421507	0.996835	101797173/101795653/101790717	DNAH5/DNAH3/DNAH9	3	3	101797173/101795653/101790717	0	
CC	GO:0098797	plasma membrane protein complex	3/192	28/2288	0.421507	0.996835	119714808/101799054/101798389	ATP1B2/SCN5A/SGCG	3	3	119714808/101799054/101798389	0	
CC	GO:0005667	transcription factor complex	2/192	17/2288	0.424128	0.996835	101801567/101801576	TAF1D/E2F1	2	1	101801576	1	101801567
CC	GO:0044429	mitochondrial part	5/192	52/2288	0.445494	0.996835	101798341/101790565/101801562/101798013/101789523	BNIP3/LOC101790565/APOOL/MPC1/CHCHD3	5	2	101801562/101789523	3	101798341/101790565/101798013
CC	GO:0098798	mitochondrial protein complex	3/192	31/2288	0.489457	0.996835	101790565/101801562/101789523	LOC101790565/APOOL/CHCHD3	3	2	101801562/101789523	1	101790565
CC	GO:0005740	mitochondrial envelope	4/192	44/2288	0.511881	0.996835	101798341/101801562/101798013/101789523	BNIP3/APOOL/MPC1/CHCHD3	4	2	101801562/101789523	2	101798341/101798013
CC	GO:0098800	inner mitochondrial membrane protein complex	2/192	21/2288	0.536939	0.996835	101801562/101789523	APOOL/CHCHD3	2	2	101801562/101789523	0	
CC	GO:0005777	peroxisome	1/192	10/2288	0.584503	0.996835	101804343	ACOX2	1	1	101804343	0	
CC	GO:0042579	microbody	1/192	10/2288	0.584503	0.996835	101804343	ACOX2	1	1	101804343	0	
CC	GO:0016469	proton-transporting two-sector ATPase complex	2/192	23/2288	0.587411	0.996835	101793897/119714948	ATP6V1E1/LOC119714948	2	1	119714948	1	101793897
CC	GO:0031410	cytoplasmic vesicle	2/192	23/2288	0.587411	0.996835	101790234/101792755	CHGA/LOC101792755	2	0		2	101790234/101792755
CC	GO:0031982	vesicle	2/192	23/2288	0.587411	0.996835	101790234/101792755	CHGA/LOC101792755	2	0		2	101790234/101792755
CC	GO:0097708	intracellular vesicle	2/192	23/2288	0.587411	0.996835	101790234/101792755	CHGA/LOC101792755	2	0		2	101790234/101792755
CC	GO:0099080	supramolecular complex	2/192	23/2288	0.587411	0.996835	119717442/101803548	DES/TNNI1	2	1	101803548	1	119717442
CC	GO:0099081	supramolecular polymer	2/192	23/2288	0.587411	0.996835	119717442/101803548	DES/TNNI1	2	1	101803548	1	119717442
CC	GO:0099512	supramolecular fiber	2/192	23/2288	0.587411	0.996835	119717442/101803548	DES/TNNI1	2	1	101803548	1	119717442
CC	GO:0031967	organelle envelope	4/192	50/2288	0.615946	0.996835	101798341/101801562/101798013/101789523	BNIP3/APOOL/MPC1/CHCHD3	4	2	101801562/101789523	2	101798341/101798013
CC	GO:0031975	envelope	4/192	50/2288	0.615946	0.996835	101798341/101801562/101798013/101789523	BNIP3/APOOL/MPC1/CHCHD3	4	2	101801562/101789523	2	101798341/101798013
CC	GO:0000139	Golgi membrane	1/192	11/2288	0.619523	0.996835	101799131	CSGALNACT1	1	1	101799131	0	
CC	GO:0000775	chromosome, centromeric region	1/192	11/2288	0.619523	0.996835	101791211	ZWILCH	1	0		1	101791211
CC	GO:0030658	transport vesicle membrane	1/192	11/2288	0.619523	0.996835	101792755	LOC101792755	1	0		1	101792755
CC	GO:0034702	ion channel complex	1/192	11/2288	0.619523	0.996835	101799054	SCN5A	1	1	101799054	0	
CC	GO:0034703	cation channel complex	1/192	11/2288	0.619523	0.996835	101799054	SCN5A	1	1	101799054	0	
CC	GO:0005615	extracellular space	2/192	25/2288	0.633715	0.996835	113839877/101793480	ECM1/ADA2	2	1	101793480	1	113839877
CC	GO:0005739	mitochondrion	5/192	65/2288	0.648694	0.996835	101798341/101790565/101801562/101798013/101789523	BNIP3/LOC101790565/APOOL/MPC1/CHCHD3	5	2	101801562/101789523	3	101798341/101790565/101798013
CC	GO:0005839	proteasome core complex	1/192	12/2288	0.651605	0.996835	101803700	PSMB1	1	0		1	101803700
CC	GO:0030133	transport vesicle	1/192	12/2288	0.651605	0.996835	101792755	LOC101792755	1	0		1	101792755
CC	GO:0098791	Golgi subcompartment	1/192	12/2288	0.651605	0.996835	101799131	CSGALNACT1	1	1	101799131	0	
CC	GO:0031966	mitochondrial membrane	3/192	40/2288	0.665788	0.996835	101801562/101798013/101789523	APOOL/MPC1/CHCHD3	3	2	101801562/101789523	1	101798013
CC	GO:0044455	mitochondrial membrane part	2/192	27/2288	0.67588	0.996835	101801562/101789523	APOOL/CHCHD3	2	2	101801562/101789523	0	
CC	GO:0098687	chromosomal region	1/192	13/2288	0.680995	0.996835	101791211	ZWILCH	1	0		1	101791211

CC	GO:0098796	membrane protein complex	8/192	109/2288	0.709613	0.996835	119714808/101801562/101793897/101792755/119714948/101789523/101799054/101798389	ATP1B2/APOOL/ATP6V1E1/LOC101792755/LOC119714948/CHCHD3/SCN5A/SGCG	8	6	119714808/101801562/119714948/101789523/101799054/101798389	2	101793897/101792755
CC	GO:0000502	proteasome complex	1/192	15/2288	0.732579	0.996835	101803700	PSMB1	1	0		1	101803700
CC	GO:0005798	Golgi-associated vesicle	1/192	15/2288	0.732579	0.996835	101792755	LOC101792755	1	0		1	101792755
CC	GO:0005882	intermediate filament	1/192	15/2288	0.732579	0.996835	119717442	DES	1	0		1	119717442
CC	GO:0012506	vesicle membrane	1/192	15/2288	0.732579	0.996835	101792755	LOC101792755	1	0		1	101792755
CC	GO:0030120	vesicle coat	1/192	15/2288	0.732579	0.996835	101792755	LOC101792755	1	0		1	101792755
CC	GO:0030135	coated vesicle	1/192	15/2288	0.732579	0.996835	101792755	LOC101792755	1	0		1	101792755
CC	GO:0030659	cytoplasmic vesicle membrane	1/192	15/2288	0.732579	0.996835	101792755	LOC101792755	1	0		1	101792755
CC	GO:0030660	Golgi-associated vesicle membrane	1/192	15/2288	0.732579	0.996835	101792755	LOC101792755	1	0		1	101792755
CC	GO:0030662	coated vesicle membrane	1/192	15/2288	0.732579	0.996835	101792755	LOC101792755	1	0		1	101792755
CC	GO:0045111	intermediate filament cytoskeleton	1/192	15/2288	0.732579	0.996835	119717442	DES	1	0		1	119717442
CC	GO:1905369	endopeptidase complex	1/192	15/2288	0.732579	0.996835	101803700	PSMB1	1	0		1	101803700
CC	GO:0033177	proton-transporting two-sector ATPase complex, proton-transporting domain	1/192	16/2288	0.755168	0.996835	119714948	LOC119714948	1	1	119714948	0	
CC	GO:0099513	polymeric cytoskeletal fiber	1/192	17/2288	0.775858	0.996835	119717442	DES	1	0		1	119717442
CC	GO:1905368	peptidase complex	1/192	17/2288	0.775858	0.996835	101803700	PSMB1	1	0		1	101803700
CC	GO:0031090	organelle membrane	5/192	76/2288	0.779094	0.996835	101801562/101798013/101799131/101792755/101789523	APOOL/MPC1/CSGALNACT1/LOC101792755/CHCHD3	5	3	101801562/101799131/101789523	2	101798013/101792755
CC	GO:0044433	cytoplasmic vesicle part	1/192	19/2288	0.812164	0.996835	101792755	LOC101792755	1	0		1	101792755
CC	GO:0031984	organelle subcompartment	2/192	38/2288	0.842034	0.996835	101799131/101801245	CSGALNACT1/SELENOS	2	1	101799131	1	101801245
CC	GO:0005789	endoplasmic reticulum membrane	1/192	26/2288	0.89893	0.996835	101801245	SELENOS	1	0		1	101801245
CC	GO:0030117	membrane coat	1/192	26/2288	0.89893	0.996835	101792755	LOC101792755	1	0		1	101792755
CC	GO:0044431	Golgi apparatus part	1/192	26/2288	0.89893	0.996835	101799131	CSGALNACT1	1	1	101799131	0	
CC	GO:0048475	coated membrane	1/192	26/2288	0.89893	0.996835	101792755	LOC101792755	1	0		1	101792755
CC	GO:0098827	endoplasmic reticulum subcompartment	1/192	26/2288	0.89893	0.996835	101801245	SELENOS	1	0		1	101801245
CC	GO:0042175	nuclear outer membrane-endoplasmic reticulum membrane network	1/192	27/2288	0.907509	0.996835	101801245	SELENOS	1	0		1	101801245
CC	GO:0044432	endoplasmic reticulum part	1/192	27/2288	0.907509	0.996835	101801245	SELENOS	1	0		1	101801245
CC	GO:1902494	catalytic complex	5/192	95/2288	0.913197	0.996835	101797173/101795653/119714808/101790717/101803700	DNAH5/DNAH3/ATP1B2/DNAH9/PSMB1	5	4	101797173/101795653/119714808/101790717	1	101803700
CC	GO:0005794	Golgi apparatus	1/192	28/2288	0.915363	0.996835	101799131	CSGALNACT1	1	1	101799131	0	
CC	GO:0005634	nucleus	22/192	334/2288	0.921793	0.996835	101800755/101801468/101794377/101800783/101799581/101798499/101801567/101801755/101792368/101794314/119714375/101802147/101789522/101789572/101793627/101802149/101790058/106018371/101797382/101791694/101796848/119714007	SESN1/CBFB/MAFK/LOC101800783/LOC101799581/LOC101798499/TAF1D/MYF5/CCNA2/NFIL3/LOC119714375/L3MBTL3/MYOD1/PPARA/BABAM2/ADRM1/BLM/NOL7/POLD3/CDKN1B/CCNL2/LOC119714007	22	6	119714375/101789572/101793627/101790058/101797382/119714007	16	101800755/101801468/101794377/101800783/101799581/101798499/101801567/101801755/101792368/101794314/101802147/101789522/101802149/106018371/101791694/101796848
CC	GO:0098588	bounding membrane of organelle	2/192	49/2288	0.92726	0.996835	101799131/101792755	CSGALNACT1/LOC101792755	2	1	101799131	1	101792755
CC	GO:0043228	non-membrane-bounded organelle	18/192	292/2288	0.947877	0.996835	101804531/119717442/101797173/101790565/101798499/101795653/110353953/101801567/101796751/101802140/101803548/106017870/101790717/101802165/113841953/101791211/101790909/101798589	MYO16/DES/DNAH5/LOC101790565/LOC101798499/DNAH3/LOC110353953/TAF1D/DST/LOC101802140/TNNI1/MRPL2/DNAH9/LOC101802165/LOC113841953/ZWILCH/LOC101790909/MYO5C	18	13	101804531/101797173/101795653/110353953/101796751/101802140/101803548/106017870/101790717/101802165/113841953/101790909/101798589	5	119717442/101790565/101798499/101801567/101791211

CC	GO:0043232	intracellular non-membrane-bounded organelle	18/192	292/2288	0.947877	0.996835	101804531/119717442/101797173/101790565/101798499/101795653/110353953/101801567/101796751/101802140/101803548/106017870/101790717/101802165/113841953/101791211/101790909/101798589	MYO16/DES/DNAH5/LOC101790565/LOC101798499/DNAH3/LOC110353953/TAF1D/DST/LOC101802140/TNNI1/MRPL2/DNAH9/LOC101802165/LOC113841953/ZWILCH/LOC101790909/MYO5C	18	13	101804531/101797173/101795653/110353953/101796751/101802140/101803548/106017870/101790717/101802165/113841953/101790909/101798589	5	119717442/101790565/101798499/101801567/101791211
CC	GO:0012505	endomembrane system	4/192	90/2288	0.953188	0.996835	101799131/101790234/101792755/101801245	CSGALNACT1/CHGA/LOC101792755/SELENOS	4	1	101799131	3	101790234/101792755/101801245
CC	GO:0098805	whole membrane	1/192	38/2288	0.965232	0.996835	101792755	LOC101792755	1	0		1	101792755
CC	GO:0005783	endoplasmic reticulum	1/192	40/2288	0.970914	0.996835	101801245	SELENOS	1	0		1	101801245
CC	GO:0043227	membrane-bounded organelle	32/192	498/2288	0.972723	0.996835	101798341/101800755/101801468/101794377/101800783/101799581/101790565/101798499/101801567/101801562/101798013/101801755/101792368/101794314/119714375/101799131/101804343/101790234/101802147/101792755/101789522/101789572/101793627/101802149/101790058/101789523/106018371/101797382/101791694/101796848/101801245/119714007	BNIP3/SESN1/CBFB/MAFK/LOC101800783/LOC101799581/LOC101790565/LOC101798499/TAF1D/APOOL/MPC1/MYF5/CCNA2/NFIL3/LOC119714375/CSGALNACT1/ACOX2/CHGA/L3MBTL3/LOC101792755/MYOD1/PPARA/BABAM2/ADRM1/BLM/CHCHD3/NOL7/POLD3/CDKN1B/CCNL2/SELENOS/LOC119714007	32	10	101801562/119714375/101799131/101804343/101789572/101793627/101790058/101789523/101797382/119714007	22	101798341/101800755/101801468/101794377/101800783/101799581/101790565/101798499/101801567/101798013/101801755/101792368/101794314/101790234/101802147/101792755/101789522/101802149/106018371/101791694/101796848/101801245
CC	GO:0000786	nucleosome	1/192	41/2288	0.973398	0.996835	101798499	LOC101798499	1	0		1	101798499
CC	GO:0031974	membrane-enclosed lumen	3/192	82/2288	0.9744	0.996835	101799581/101790565/101801567	LOC101799581/LOC101790565/TAF1D	3	0		3	101799581/101790565/101801567
CC	GO:0043233	organelle lumen	3/192	82/2288	0.9744	0.996835	101799581/101790565/101801567	LOC101799581/LOC101790565/TAF1D	3	0		3	101799581/101790565/101801567
CC	GO:0070013	intracellular organelle lumen	3/192	82/2288	0.9744	0.996835	101799581/101790565/101801567	LOC101799581/LOC101790565/TAF1D	3	0		3	101799581/101790565/101801567
CC	GO:0032993	protein-DNA complex	1/192	42/2288	0.975671	0.996835	101798499	LOC101798499	1	0		1	101798499
CC	GO:0044815	DNA packaging complex	1/192	42/2288	0.975671	0.996835	101798499	LOC101798499	1	0		1	101798499
CC	GO:0044446	intracellular organelle part	23/192	381/2288	0.975963	0.996835	101804531/101798341/119717442/101797173/101799581/101790565/101798499/101795653/110353953/101801567/101801562/101798013/101803548/101799131/101792755/101793627/101789523/101790717/101802165/101801245/101791211/101790909/101798589	MYO16/BNIP3/DES/DNAH5/LOC101799581/LOC101790565/LOC101798499/DNAH3/LOC110353953/TAF1D/APOOL/MPC1/TNNI1/CSGALNACT1/LOC101792755/BABAM2/CHCHD3/DNAH9/LOC101802165/SELENOS/ZWILCH/LOC101790909/MYO5C	23	13	101804531/101797173/101795653/110353953/101801562/101803548/101799131/101793627/101789523/101790717/101802165/101790909/101798589	10	101798341/119717442/101799581/101790565/101798499/101801567/101798013/101792755/101801245/101791211
CC	GO:0044422	organelle part	23/192	383/2288	0.977731	0.996835	101804531/101798341/119717442/101797173/101799581/101790565/101798499/101795653/110353953/101801567/101801562/101798013/101803548/101799131/101792755/101793627/101789523/101790717/101802165/101801245/101791211/101790909/101798589	MYO16/BNIP3/DES/DNAH5/LOC101799581/LOC101790565/LOC101798499/DNAH3/LOC110353953/TAF1D/APOOL/MPC1/TNNI1/CSGALNACT1/LOC101792755/BABAM2/CHCHD3/DNAH9/LOC101802165/SELENOS/ZWILCH/LOC101790909/MYO5C	23	13	101804531/101797173/101795653/110353953/101801562/101803548/101799131/101793627/101789523/101790717/101802165/101790909/101798589	10	101798341/119717442/101799581/101790565/101798499/101801567/101798013/101792755/101801245/101791211

CC	GO:0043231	intracellular membrane-bounded organelle	30/192	483/2288	0.981835	0.996835	101798341/101800755/101801468/101794377/101800783/101799581/101790565/101798499/101801567/101801562/101798013/101801755/101792368/101794314/119714375/101799131/101804343/101802147/101789522/101789572/101793627/101802149/101790058/101789523/106018371/101797382/101791694/101796848/101801245/119714007	BNIP3/SESN1/CBFB/MAFK/LOC101800783/LOC101799581/LOC101790565/LOC101798499/TAF1D/APOOL/MPC1/MYF5/CCNA2/NFIL3/LOC119714375/CSGALNACT1/ACOX2/L3MBTL3/MYOD1/PPARA/BABAM2/ADRM1/BLM/CHCHD3/NOL7/POLD3/CDKN1B/CCNL2/SELENOS/LOC119714007	30	10	101801562/119714375/101799131/101804343/101789572/101793627/101790058/101789523/101797382/119714007	20	101798341/101800755/101801468/101794377/101800783/101799581/101790565/101798499/101801567/101798013/101801755/101792368/101794314/101802147/101789522/101802149/106018371/101791694/101796848/101801245
CC	GO:0000785	chromatin	1/192	46/2288	0.982987	0.996835	101798499	LOC101798499	1	0		1	101798499
CC	GO:0044427	chromosomal part	2/192	69/2288	0.983826	0.996835	101798499/101791211	LOC101798499/ZWILCH	2	0		2	101798499/101791211
CC	GO:0005840	ribosome	3/192	89/2288	0.984278	0.996835	101790565/101802140/106017870	LOC101790565/LOC101802140/MRPL2	3	2	101802140/106017870	1	101790565
CC	GO:0031981	nuclear lumen	2/192	72/2288	0.987192	0.996835	101799581/101801567	LOC101799581/TAF1D	2	0		2	101799581/101801567
CC	GO:0005654	nucleoplasm	1/192	53/2288	0.990917	0.996835	101799581	LOC101799581	1	0		1	101799581
CC	GO:0044451	nucleoplasm part	1/192	53/2288	0.990917	0.996835	101799581	LOC101799581	1	0		1	101799581
CC	GO:0044428	nuclear part	3/192	98/2288	0.991741	0.996835	101799581/101801567/101793627	LOC101799581/TAF1D/BABAM2	3	1	101793627	2	101799581/101801567
CC	GO:0005737	cytoplasm	19/192	351/2288	0.991758	0.996835	101798341/101794693/101790565/119714249/101803288/101801562/101798013/119716581/101802140/101803548/101799131/101804343/101790234/106014280/101792755/101802149/101789523/106017870/101801245	BNIP3/DBNDD2/LOC101790565/LOC119714249/LOC101803288/APOOL/MPC1/LOC119716581/LOC101802140/TNNI1/CSGALNACT1/ACOX2/CHGA/PPA2/LOC101792755/ADRM1/CHCHD3/MRPL2/SELENOS	19	9	119714249/101801562/119716581/101802140/101803548/101799131/101804343/101789523/106017870	10	101798341/101794693/101790565/101803288/101798013/101790234/106014280/101792755/101802149/101801245
CC	GO:0005694	chromosome	2/192	78/2288	0.992008	0.996835	101798499/101791211	LOC101798499/ZWILCH	2	0		2	101798499/101791211
CC	GO:0044444	cytoplasmic part	13/192	281/2288	0.996723	0.996835	101798341/101790565/101801562/101798013/101802140/101803548/101799131/101804343/101790234/101792755/101789523/106017870/101801245	BNIP3/LOC101790565/APOOL/MPC1/LOC101802140/TNNI1/CSGALNACT1/ACOX2/CHGA/LOC101792755/CHCHD3/MRPL2/SELENOS	13	7	101801562/101802140/101803548/101799131/101804343/101789523/106017870	6	101798341/101790565/101798013/101790234/101792755/101801245
CC	GO:1990904	ribonucleoprotein complex	3/192	111/2288	0.996835	0.996835	101790565/101802140/106017870	LOC101790565/LOC101802140/MRPL2	3	2	101802140/106017870	1	101790565

MF	GO:0016491	oxidoreductase activity	38/543	286/6527	0.002372	0.583399	101793380/101803853/101794631/113839590/101801152/101799809/101803074/101789838/101794957/101802594/101795743/101797675/101799210/101798440/101801759/101805057/101805119/101789745/101793275/119713271/119714639/101798614/101804343/101789907/101793939/119717912/101792573/101804610/101792912/101804444/101801193/101791774/101791081/101797377/5405820/101804478/101799397/101801058	AASS/ME1/LOC101794631/LOC113839590/LDHB/HADHA/ALDH1L2/ETFDH/EGLN1/DMGDH/NDUFAF2/D2HGDH/LOC101799210/LOC101798440/ALDH1A2/HMOX1/NDUFS1/ME3/ALDH7A1/LOC119713271/ACADM/BCO1/ACOX2/IVD/ALDH5A1/LOC119717912/LOC101792573/MDH2/LOC101792912/L2HGDH/ALDH8A1/ALDH4A1/LOC101791081/GLUD1/ND5/PRDX6/SRD5A2/LOC101801058	38	24	101803853/101801152/101799809/101803074/101789838/101794957/101802594/101797675/101799210/101801759/101805119/119714639/101798614/101804343/101793939/119717912/101804610/101792912/101804444/101791081/5405820/101804478/101799397/101801058	14	101793380/101794631/113839590/101795743/101798440/101805057/101789745/101793275/119713271/101789907/101792573/101801193/101791774/101797377
MF	GO:0016887	ATPase activity	12/543	67/6527	0.008425	0.743482	101799966/101790476/119713166/101793897/101804022/119718656/101790058/101797870/101793641/101804688/101802999/101790402	ABCG8/ABCB6/LOC119713166/ATP6V1E1/VWA8/LOC119718656/BLM/XRCC5/ATP13A2/ABCC8/ABCC1/ATP5PO	12	8	101799966/101790476/101804022/101790058/101797870/101804688/101802999/101790402	4	119713166/101793897/119718656/101793641
MF	GO:0004435	phosphatidylinositol phospholipase C activity	4/543	12/6527	0.013659	0.743482	101803647/101790966/101799246/101796880	PLCL2/PLCB1/PLCG2/PLCD3	4	2	101803647/101790966	2	101799246/101796880
MF	GO:0004629	phospholipase C activity	4/543	12/6527	0.013659	0.743482	101803647/101790966/101799246/101796880	PLCL2/PLCB1/PLCG2/PLCD3	4	2	101803647/101790966	2	101799246/101796880
MF	GO:0016616	oxidoreductase activity, acting on the CH-OH group of donors, NAD or NADP as acceptor	7/543	33/6527	0.016843	0.743482	101803853/101801152/101799809/101799210/101798440/101789745/101804610	ME1/LDHB/HADHA/LOC101799210/LOC101798440/ME3/MDH2	7	5	101803853/101801152/101799809/101799210/101804610	2	101798440/101789745
MF	GO:0016614	oxidoreductase activity, acting on CH-OH group of donors	7/543	35/6527	0.022961	0.743482	101803853/101801152/101799809/101799210/101798440/101789745/101804610	ME1/LDHB/HADHA/LOC101799210/LOC101798440/ME3/MDH2	7	5	101803853/101801152/101799809/101799210/101804610	2	101798440/101789745
MF	GO:0005520	insulin-like growth factor binding	4/543	14/6527	0.024178	0.743482	101803411/101790193/101799001/101803685	IGFBP5/CCN2/CCN1/IGFBP3	4	1	101803411	3	101790193/101799001/101803685
MF	GO:0019838	growth factor binding	4/543	14/6527	0.024178	0.743482	101803411/101790193/101799001/101803685	IGFBP5/CCN2/CCN1/IGFBP3	4	1	101803411	3	101790193/101799001/101803685
MF	GO:0004721	phosphoprotein phosphatase activity	10/543	63/6527	0.033804	0.802505	101789439/101802393/101804352/101803221/101803683/101792804/101803878/101791271/101804166/101803609	LOC101789439/DUSP1/PTPRD/PTPN14/DUSP28/DUSP15/PTPRT/PTPN6/DUSP3/PTPRM	10	6	101789439/101804352/101803683/101803878/101804166/101803609	4	101802393/101803221/101792804/101791271
MF	GO:0042623	ATPase activity, coupled	8/543	46/6527	0.033966	0.802505	101790476/101793897/119718656/101790058/101797870/101804688/101802999/101790402	ABCB6/ATP6V1E1/LOC119718656/BLM/XRCC5/ABCC8/ABCC1/ATP5PO	8	6	101790476/101790058/101797870/101804688/101802999/101790402	2	101793897/119718656
MF	GO:0004725	protein tyrosine phosphatase activity	6/543	31/6527	0.039625	0.802505	101789439/101804352/101803221/101803878/101791271/101803609	LOC101789439/PTPRD/PTPN14/PTPRT/PTPN6/PTPRM	6	4	101789439/101804352/101803878/101803609	2	101803221/101791271
MF	GO:0019239	deaminase activity	3/543	10/6527	0.044163	0.802505	101803104/101794720/101793480	AMPD3/AMPD1/ADA2	3	2	101794720/101793480	1	101803104
MF	GO:0042578	phosphoric ester hydrolase activity	15/543	113/6527	0.046631	0.802505	101789439/101802393/101803647/101804352/101790966/101796581/101803221/101803683/101792804/101803878/101799246/101791271/101804166/101803609/101796880	LOC101789439/DUSP1/PLCL2/PTPRD/PLCB1/PDE9A/PTPN14/DUSP28/DUSP15/PTPRT/PLCG2/PTPN6/DUSP3/PTPRM/PLCD3	15	9	101789439/101803647/101804352/101790966/101796581/101803683/101803878/101804166/101803609	6	101802393/101803221/101792804/101799246/101791271/101796880

MF	GO:0016651	oxidoreductase activity, acting on NAD(P)H	3/543	11/6527	0.057073	0.802505	101795743/101805119/5405820	NDUFAF2/NDUFS1/ND5	3	2	101805119/5405820	1	101795743
MF	GO:0015399	primary active transmembrane transporter activity	6/543	34/6527	0.05872	0.802505	101790476/101793897/119718656/101804688/101802999/101790402	ABCB6/ATP6V1E1/LOC119718656/ABCC8/ABCC1/ATP5PO	6	4	101790476/101804688/101802999/101790402	2	101793897/119718656
MF	GO:0015405	P-P-bond-hydrolysis-driven transmembrane transporter activity	6/543	34/6527	0.05872	0.802505	101790476/101793897/119718656/101804688/101802999/101790402	ABCB6/ATP6V1E1/LOC119718656/ABCC8/ABCC1/ATP5PO	6	4	101790476/101804688/101802999/101790402	2	101793897/119718656
MF	GO:0042626	ATPase activity, coupled to transmembrane movement of substances	6/543	34/6527	0.05872	0.802505	101790476/101793897/119718656/101804688/101802999/101790402	ABCB6/ATP6V1E1/LOC119718656/ABCC8/ABCC1/ATP5PO	6	4	101790476/101804688/101802999/101790402	2	101793897/119718656
MF	GO:0043492	ATPase activity, coupled to movement of substances	6/543	34/6527	0.05872	0.802505	101790476/101793897/119718656/101804688/101802999/101790402	ABCB6/ATP6V1E1/LOC119718656/ABCC8/ABCC1/ATP5PO	6	4	101790476/101804688/101802999/101790402	2	101793897/119718656
MF	GO:0016829	lyase activity	7/543	44/6527	0.068951	0.848256	101798008/101789492/101803512/101803045/101796516/101799385/101800658	GADL1/FH/ADCY2/DERA/LOC101796516/CENPV/PCBD1	7	5	101798008/101789492/101803512/101803045/101796516	2	101799385/101800658
MF	GO:0004674	protein serine/threonine kinase activity	8/543	53/6527	0.06969	0.848256	101793905/101793152/101799089/101800942/101797446/101801648/101801641/101793707	ULK2/WNK2/MAST4/RPS6KA1/OXSR1/CAMK2D/LOC101801641/BMPR2	8	4	101793152/101799089/101801648/101793707	4	101793905/101800942/101797446/101801641
MF	GO:0016791	phosphatase activity	10/543	72/6527	0.073173	0.848256	101789439/101802393/101804352/101803221/101803683/101792804/101803878/101791271/101804166/101803609	LOC101789439/DUSP1/PTPRD/PTPN14/DUSP28/DUSP15/PTPRT/PTPN6/DUSP3/PTPRM	10	6	101789439/101804352/101803683/101803878/101804166/101803609	4	101802393/101803221/101792804/101791271
MF	GO:0004866	endopeptidase inhibitor activity	6/543	38/6527	0.091414	0.848256	101792619/101794822/101796096/101794158/101790881/101795277	TIMP4/TFPI/LOC101796096/COL6A3/KNG1/APLP2	6	3	101794158/101790881/101795277	3	101792619/101794822/101796096
MF	GO:0061135	endopeptidase regulator activity	6/543	38/6527	0.091414	0.848256	101792619/101794822/101796096/101794158/101790881/101795277	TIMP4/TFPI/LOC101796096/COL6A3/KNG1/APLP2	6	3	101794158/101790881/101795277	3	101792619/101794822/101796096
MF	GO:0004620	phospholipase activity	5/543	30/6527	0.098895	0.848256	101803647/101790966/101804872/101799246/101796880	PLCL2/PLCB1/LOC101804872/PLCG2/PLCD3	5	2	101803647/101790966	3	101804872/101799246/101796880
MF	GO:0016298	lipase activity	5/543	30/6527	0.098895	0.848256	101803647/101790966/101804872/101799246/101796880	PLCL2/PLCB1/LOC101804872/PLCG2/PLCD3	5	2	101803647/101790966	3	101804872/101799246/101796880
MF	GO:0030414	peptidase inhibitor activity	6/543	39/6527	0.100862	0.848256	101792619/101794822/101796096/101794158/101790881/101795277	TIMP4/TFPI/LOC101796096/COL6A3/KNG1/APLP2	6	3	101794158/101790881/101795277	3	101792619/101794822/101796096
MF	GO:0061134	peptidase regulator activity	6/543	39/6527	0.100862	0.848256	101792619/101794822/101796096/101794158/101790881/101795277	TIMP4/TFPI/LOC101796096/COL6A3/KNG1/APLP2	6	3	101794158/101790881/101795277	3	101792619/101794822/101796096
MF	GO:0008289	lipid binding	10/543	77/6527	0.104004	0.848256	101790249/101794730/101792273/101800869/101791151/101792242/101797331/101803768/101798726/101789868	ANXA1/ANXA11/ANXA2/ANXA7/STARD6/SNX16/APOA4/KIF16B/APOLD1/PXK	10	1	101797331	9	101790249/101794730/101792273/101800869/101791151/101792242/101803768/101798726/101789868
MF	GO:0016788	hydrolase activity, acting on ester bonds	22/543	201/6527	0.110223	0.848256	101802979/101789439/119715945/101802393/101803288/101803647/101804352/101790966/101796581/119716581/101803221/101804872/101803683/101792804/101803878/101799246/101791271/101800595/101804166/101803609/101796880/101791157	ARSI/LOC101789439/LOC119715945/DUSP1/LOC101803288/PLCL2/PTPRD/PLCB1/PDE9A/LOC119716581/PTPN14/LOC101804872/DUSP28/DUSP15/PTPRT/PLCG2/PTPN6/RNASEL/DUSP3/PTPRM/PLCD3/TATDN3	22	12	101802979/101789439/101803647/101804352/101790966/101796581/119716581/101803683/101803878/101800595/101804166/101803609	10	119715945/101802393/101803288/101803221/101804872/101792804/101799246/101791271/101796880/101791157

MF	GO:0004222	metalloendopeptidase activity	8/543	59/6527	0.113734	0.848256	101791511/101794528/101794584/101800115/119714444/101804308/101790574/101800508	PHEX/LOC101794528/ADAMTS17/ADAMTS18/ADAMTS19/MMP23B/ADAMTS5/LOC101800508	8	5	101791511/101800115/119714444/101804308/101800508	3	101794528/101794584/101790574
MF	GO:0004857	enzyme inhibitor activity	7/543	50/6527	0.118004	0.848256	101792619/101794822/101796096/101794158/101790881/101791694/101795277	TIMP4/TFPI/LOC101796096/COL6A3/KNG1/CDKN1B/APLP2	7	3	101794158/101790881/101795277	4	101792619/101794822/101796096/101791694
MF	GO:0051287	NAD binding	4/543	23/6527	0.118852	0.848256	101790158/101803853/101798440/101789745	HIBADH/ME1/LOC101798440/ME3	4	1	101803853	3	101790158/101798440/101789745
MF	GO:0022804	active transmembrane transporter activity	8/543	60/6527	0.122269	0.848256	101791662/101790476/101793897/119718656/101795056/101804688/101802999/101790402	SLC9A6/ABCB6/ATP6V1E1/LOC119718656/SLC6A1/ABCC8/ABCC1/ATP5PO	8	5	101790476/101795056/101804688/101802999/101790402	3	101791662/101793897/119718656
MF	GO:0030246	carbohydrate binding	5/543	32/6527	0.122296	0.848256	101803645/113845291/101791338/101790157/101800542	ADGRL3/LOC113845291/EVA1C/STBD1/ENPP3	5	4	101803645/113845291/101790157/101800542	1	101791338
MF	GO:0005201	extracellular matrix structural constituent	3/543	15/6527	0.123199	0.848256	101799662/101800228/101793387	COL4A5/COL4A6/COL5A1	3	3	101799662/101800228/101793387	0	
MF	GO:0016462	pyrophosphatase activity	36/543	356/6527	0.124184	0.848256	101804531/101797173/101795653/110353953/101799698/101799966/119714718/101798141/101790443/106014280/101790476/119713166/101793897/101804022/119714941/106020577/101803472/101803543/101803768/119718656/101790058/101797870/101790717/101802165/119718018/101793641/101803065/101790303/101802364/101804688/101790909/101804925/101798589/101802999/101799739/101790402	MYO16/DNAH5/DNAH3/LOC110353953/DIRAS2/ABCG8/LOC119714718/RRAD/KIF26A/PPA2/ABCB6/LOC119713166/ATP6V1E1/VWA8/LOC119714941/KIF3C/ITPA/RASEF/KIF16B/LOC119718656/BLM/XRCC5/DNAH9/LOC101802165/LOC119718018/ATP13A2/RASL11A/RAB39A/MRAS/ABCC8/LOC101790909/RAB9A/MYO5C/ABCC1/RAB40B/ATP5PO	36	21	101804531/101797173/101795653/110353953/101799698/101799966/101790443/101790476/101804022/106020577/101790058/101797870/101790717/101802165/101802364/101804688/101790909/101798589/101802999/101799739/101790402	15	119714718/101798141/106014280/119713166/101793897/119714941/101803472/101803543/101803768/119718656/119718018/101793641/101803065/101790303/101804925
MF	GO:0005543	phospholipid binding	7/543	51/6527	0.127583	0.848256	101790249/101794730/101792273/101800869/101792242/101803768/101789868	ANXA1/ANXA11/ANXA2/ANXA7/SNX16/KIF16B/PXK	7	0		7	101790249/101794730/101792273/101800869/101792242/101803768/101789868
MF	GO:0016818	hydrolase activity, acting on acid anhydrides, in phosphorus-containing anhydrides	36/543	358/6527	0.131287	0.849913	101804531/101797173/101795653/110353953/101799698/101799966/119714718/101798141/101790443/106014280/101790476/119713166/101793897/101804022/119714941/106020577/101803472/101803543/101803768/119718656/101790058/101797870/101790717/101802165/119718018/101793641/101803065/101790303/101802364/101804688/101790909/101804925/101798589/101802999/101799739/101790402	MYO16/DNAH5/DNAH3/LOC110353953/DIRAS2/ABCG8/LOC119714718/RRAD/KIF26A/PPA2/ABCB6/LOC119713166/ATP6V1E1/VWA8/LOC119714941/KIF3C/ITPA/RASEF/KIF16B/LOC119718656/BLM/XRCC5/DNAH9/LOC101802165/LOC119718018/ATP13A2/RASL11A/RAB39A/MRAS/ABCC8/LOC101790909/RAB9A/MYO5C/ABCC1/RAB40B/ATP5PO	36	21	101804531/101797173/101795653/110353953/101799698/101799966/101790443/101790476/101804022/106020577/101790058/101797870/101790717/101802165/101802364/101804688/101790909/101798589/101802999/101799739/101790402	15	119714718/101798141/106014280/119713166/101793897/119714941/101803472/101803543/101803768/119718656/119718018/101793641/101803065/101790303/101804925
MF	GO:0004867	serine-type endopeptidase inhibitor activity	3/543	16/6527	0.142742	0.853022	101794822/101794158/101795277	TFPI/COL6A3/APLP2	3	2	101794158/101795277	1	101794822

MF	GO:0016879	ligase activity, forming carbon-nitrogen bonds	3/543	16/6527	0.142742	0.853022	101801928/101799450/101797455	ADSS1/GLUL/ASNS	3	1	101801928	2	101799450/101797455
MF	GO:0016817	hydrolase activity, acting on acid anhydrides	36/543	362/6527	0.146235	0.853022	101804531/101797173/101795653/110353953/101799698/101799966/119714718/101798141/101790443/106014280/101790476/119713166/101793897/101804022/119714941/106020577/101803472/101803543/101803768/119718656/101790058/101797870/101790717/101802165/119718018/101793641/101803065/101790303/101802364/101804688/101790909/101804925/101798589/101802999/101799739/101790402	MYO16/DNAH5/DNAH3/LOC110353953/DIRAS2/ABCG8/LOC119714718/RRAD/KIF26A/PPA2/ABCB6/LOC119713166/ATP6V1E1/VWA8/LOC119714941/KIF3C/ITPA/RASEF/KIF16B/LOC119718656/BLM/XRCC5/DNAH9/LOC101802165/LOC119718018/ATP13A2/RASL11A/RAB39A/MRAS/ABCC8/LOC101790909/RAB9A/MYO5C/ABCC1/RAB40B/ATP5PO	36	21	101804531/101797173/101795653/110353953/101799698/101799966/101790443/101790476/101804022/106020577/101790058/101797870/101790717/101802165/101802364/101804688/101790909/101798589/101802999/101799739/101790402	15	119714718/101798141/106014280/119713166/101793897/119714941/101803472/101803543/101803768/119718656/119718018/101793641/101803065/101790303/101804925
MF	GO:0016627	oxidoreductase activity, acting on the CH-CH group of donors	4/543	25/6527	0.149419	0.853022	119714639/101804343/101789907/101799397	ACADM/ACOX2/IVD/SRD5A2	4	3	119714639/101804343/101799397	1	101789907
MF	GO:0016773	phosphotransferase activity, alcohol group as acceptor	38/543	387/6527	0.156933	0.853022	101801853/101793905/101793152/101802614/101803371/101795985/101802855/101797616/101799089/101795498/101803562/101803181/101791030/101804342/101791519/101799561/101800942/101802737/101793068/101795713/101790169/101790726/101792645/101797446/101800595/101804452/101795633/110351624/101801648/101801641/101791769/101793707/101801143/106017602/101791037/101796806/101797327/101789868	TNIK/ULK2/WNK2/PRKG1/MAP2K3/LOC101795985/CAMK1G/LOC101797616/MAST4/PIP4K2A/TRIB1/MAP3K6/STYK1/SIK1/MAP3K15/PRKAA2/RPS6KA1/CLK1/PHKG1/KALRN/LOC101790169/PKM/PIM3/OXSR1/RNASEL/MUSK/RAF1/LOC110351624/CAMK2D/LOC101801641/MAP3K8/BMPR2/DGKI/TSSK2/MAP2K1/CDK10/LOC101797327/PXK	38	16	101801853/101793152/101802614/101802855/101799089/101795498/101791519/101799561/101790726/101800595/101804452/110351624/101801648/101793707/101801143/101797327	22	101793905/101803371/101795985/101797616/101803562/101803181/101791030/101804342/101800942/101802737/101793068/101795713/101790169/101792645/101797446/101795633/101801641/101791769/106017602/101791037/101796806/101789868
MF	GO:0003774	motor activity	12/543	105/6527	0.161221	0.853022	101804531/101797173/101795653/110353953/101790443/119714941/106020577/101803768/101790717/101802165/101790909/101798589	MYO16/DNAH5/DNAH3/LOC110353953/KIF26A/LOC119714941/KIF3C/KIF16B/DNAH9/LOC101802165/LOC101790909/MYO5C	12	10	101804531/101797173/101795653/110353953/101790443/106020577/101790717/101802165/101790909/101798589	2	119714941/101803768
MF	GO:0005261	cation channel activity	5/543	35/6527	0.161657	0.853022	119714187/101795864/119717511/101799054/101797930	KCNN1/KCNE4/ASIC4/SCN5A/TMEM38A	5	5	119714187/101795864/119717511/101799054/101797930	0	
MF	GO:0052689	carboxylic ester hydrolase activity	3/543	17/6527	0.163224	0.853022	101803288/119716581/101804872	LOC101803288/LOC119716581/LOC101804872	3	1	119716581	2	101803288/101804872
MF	GO:0015077	monovalent inorganic cation transmembrane transporter activity	9/543	75/6527	0.168556	0.853022	119714187/101795864/101791662/101793897/119717511/119714948/101795056/101799054/101790402	KCNN1/KCNE4/SLC9A6/ATP6V1E1/ASIC4/LOC119714948/SLC6A1/SCN5A/ATP5PO	9	7	119714187/101795864/119717511/119714948/101795056/101799054/101790402	2	101791662/101793897

MF	GO:0003777	microtubule motor activity	7/543	55/6527	0.169566	0.853022	101797173/101795653/101790443/119714941/106020577/101803768/101790717	DNAH5/DNAH3/KIF26A/LOC119714941/KIF3C/KIF16B/DNAH9	7	5	101797173/101795653/101790443/106020577/101790717	2	119714941/101803768
MF	GO:0015267	channel activity	16/543	149/6527	0.173997	0.853022	101798538/106020272/119714187/101802086/101795864/119715418/101801744/101800466/119717511/101801311/101795086/101799054/101798462/119713969/101797930/101800270	AQP4/CHRNG/KCNN1/AQP9/KCNE4/KCNH2/KCNH6/CHRNA10/ASIC4/LOC101801311/KCNQ4/SCN5A/GABRG3/KCNC4/TMEM38A/KCNH7	16	13	101798538/106020272/119714187/101802086/101795864/119715418/101801744/119717511/101801311/101799054/101798462/101797930/101800270	3	101800466/101795086/119713969
MF	GO:0022803	passive transmembrane transporter activity	16/543	149/6527	0.173997	0.853022	101798538/106020272/119714187/101802086/101795864/119715418/101801744/101800466/119717511/101801311/101795086/101799054/101798462/119713969/101797930/101800270	AQP4/CHRNG/KCNN1/AQP9/KCNE4/KCNH2/KCNH6/CHRNA10/ASIC4/LOC101801311/KCNQ4/SCN5A/GABRG3/KCNC4/TMEM38A/KCNH7	16	13	101798538/106020272/119714187/101802086/101795864/119715418/101801744/119717511/101801311/101799054/101798462/101797930/101800270	3	101800466/101795086/119713969
MF	GO:0016866	intramolecular transferase activity	3/543	18/6527	0.184512	0.853022	101798581/101790428/101795379	PGM2L1/PGM1/PGM5	3	3	101798581/101790428/101795379	0	
MF	GO:0017111	nucleoside-triphosphatase activity	34/543	350/6527	0.189895	0.853022	101804531/101797173/101795653/110353953/101799698/101799966/119714718/101798141/101790443/101790476/119713166/101793897/101804022/119714941/106020577/101803543/101803768/119718656/101790058/101797870/101790717/101802165/119718018/101793641/101803065/101790303/101802364/101804688/101790909/101804925/101798589/101802999/101799739/101790402	MYO16/DNAH5/DNAH3/LOC110353953/DIRAS2/ABCG8/LOC119714718/RRAD/KIF26A/ABCB6/LOC119713166/ATP6V1E1/VWA8/LOC119714941/KIF3C/RASEF/KIF16B/LOC119718656/BLM/XRCC5/DNAH9/LOC101802165/LOC119718018/ATP13A2/RASL11A/RAB39A/MRAS/ABCC8/LOC101790909/RAB9A/MYO5C/ABCC1/RAB40B/ATP5PO	34	21	101804531/101797173/101795653/110353953/101799698/101799966/101790443/101790476/101804022/106020577/101790058/101797870/101790717/101802165/101802364/101804688/101790909/101798589/101802999/101799739/101790402	13	119714718/101798141/119713166/101793897/119714941/101803543/101803768/119718656/119718018/101793641/101803065/101790303/101804925
MF	GO:0016301	kinase activity	38/543	395/6527	0.19002	0.853022	101801853/101793905/101793152/101802614/101803371/101795985/101802855/101797616/101799089/101795498/101803562/101803181/101791030/101804342/101791519/101799561/101800942/101802737/101793068/101795713/101790169/101792645/101797446/101800595/101804452/101795633/110351624/101801648/101801641/101791769/101793707/101801143/106017602/101791037/101796806/101797078/101797327/101789868	TNIK/ULK2/WNK2/PRKG1/MAP2K3/LOC101795985/CAMK1G/LOC101797616/MAST4/PIP4K2A/TRIB1/MAP3K6/STYK1/SIK1/MAP3K15/PRKAA2/RPS6KA1/CLK1/PHKG1/KALRN/LOC101790169/PIM3/OXSR1/RNASEL/MUSK/RAF1/LOC110351624/CAMK2D/LOC101801641/MAP3K8/BMPR2/DGKI/TSSK2/MAP2K1/CDK10/IP6K2/LOC101797327/PXK	38	15	101801853/101793152/101802614/101802855/101799089/101795498/101791519/101799561/101800595/101804452/110351624/101801648/101793707/101801143/101797327	23	101793905/101803371/101795985/101797616/101803562/101803181/101791030/101804342/101800942/101802737/101793068/101795713/101790169/101792645/101797446/101795633/101801641/101791769/106017602/101791037/101796806/101797078/101789868
MF	GO:0008081	phosphoric diester hydrolase activity	5/543	37/6527	0.190371	0.853022	101803647/101790966/101796581/101799246/101796880	PLCL2/PLCB1/PDE9A/PLCG2/PLCD3	5	3	101803647/101790966/101796581	2	101799246/101796880

MF	GO:0008234	cysteine-type peptidase activity	8/543	67/6527	0.190716	0.853022	101803168/101799480/101792575/101804246/113839605/101794268/101791234/101794402	CTSL/UCHL1/USP44/CTSZ/LOC113839605/CTSB/USP49/TINAG	8	0		8	101803168/101799480/101792575/101804246/113839605/101794268/101791234/101794402
MF	GO:0048037	cofactor binding	19/543	185/6527	0.197394	0.861765	101798008/101790158/101803853/101794631/113845564/101797675/101798440/101790101/101805119/101789745/119714639/101789907/101792573/101794024/101792912/101796516/101799937/101791081/101801058	GADL1/HIBADH/ME1/LOC101794631/RSAD1/D2HGDH/LOC101798440/MOCOS/NDUFS1/ME3/ACADM/IVD/LOC101792573/OAT/LOC101792912/LOC101796516/SUCLG1/LOC101791081/LOC101801058	19	12	101798008/101803853/113845564/101797675/101790101/101805119/119714639/101792912/101796516/101799937/101791081/101801058	7	101790158/101794631/101798440/101789745/101789907/101792573/101794024
MF	GO:0008378	galactosyltransferase activity	2/543	10/6527	0.199677	0.861765	101803248/101793948	B3GALNT2/B3GNT2	2	0		2	101803248/101793948
MF	GO:0000287	magnesium ion binding	3/543	20/6527	0.228982	0.933055	101799089/106014280/101790726	MAST4/PPA2/PKM	3	2	101799089/101790726	1	106014280
MF	GO:0005272	sodium channel activity	2/543	11/6527	0.231367	0.933055	119717511/101799054	ASIC4/SCN5A	2	2	119717511/101799054	0	
MF	GO:0005319	lipid transporter activity	2/543	11/6527	0.231367	0.933055	101802251/101798131	PITPNM1/PITPNC1	2	1	101798131	1	101802251
MF	GO:0016684	oxidoreductase activity, acting on peroxide as acceptor	2/543	11/6527	0.231367	0.933055	101794631/101804478	LOC101794631/PRDX6	2	1	101804478	1	101794631
MF	GO:0008138	protein tyrosine/serine/threonine phosphatase activity	4/543	30/6527	0.236287	0.937525	101802393/101803683/101792804/101804166	DUSP1/DUSP28/DUSP15/DUSP3	4	2	101803683/101804166	2	101802393/101792804
MF	GO:0022890	inorganic cation transmembrane transporter activity	11/543	104/6527	0.245467	0.938112	119714187/101795864/101791662/101804595/101793897/119717511/119714948/101795056/101799054/101797930/101790402	KCNN1/KCNE4/SLC9A6/SLC39A13/ATP6V1E1/ASIC4/LOC119714948/SLC6A1/SCN5A/TMEM38A/ATP5PO	11	8	119714187/101795864/119717511/119714948/101795056/101799054/101797930/101790402	3	101791662/101804595/101793897
MF	GO:0005216	ion channel activity	14/543	137/6527	0.247875	0.938112	106020272/119714187/101795864/119715418/101801744/101800466/119717511/101801311/101795086/101799054/101798462/119713969/101797930/101800270	CHRNG/KCNN1/KCNE4/KCNH2/KCNH6/CHRNA10/ASIC4/LOC101801311/KCNQ4/SCN5A/GABRG3/KCNC4/TMEM38A/KCNH7	14	11	106020272/119714187/101795864/119715418/101801744/119717511/101801311/101799054/101798462/101797930/101800270	3	101800466/101795086/119713969
MF	GO:0022838	substrate-specific channel activity	14/543	137/6527	0.247875	0.938112	106020272/119714187/101795864/119715418/101801744/101800466/119717511/101801311/101795086/101799054/101798462/119713969/101797930/101800270	CHRNG/KCNN1/KCNE4/KCNH2/KCNH6/CHRNA10/ASIC4/LOC101801311/KCNQ4/SCN5A/GABRG3/KCNC4/TMEM38A/KCNH7	14	11	106020272/119714187/101795864/119715418/101801744/119717511/101801311/101799054/101798462/101797930/101800270	3	101800466/101795086/119713969
MF	GO:0004672	protein kinase activity	33/543	353/6527	0.262438	0.939257	101801853/101793905/101793152/101802614/101803371/101802855/101797616/101799089/101803562/101803181/101791030/101804342/101791519/101799561/101800942/101802737/101793068/101795713/101790169/101792645/101797446/101800595/101804452/101795633/101801648/101801641/101791769/101793707/106017602/101791037/101796806/101797327/101789868	TNIK/ULK2/WNK2/PRKG1/MAP2K3/CAMK1G/LOC101797616/MAST4/TRIB1/MAP3K6/STYK1/SIK1/MAP3K15/PRKAA2/RPS6KA1/CLK1/PHKG1/KALRN/LOC101790169/PIM3/OXSR1/RNASEL/MUSK/RAF1/CAMK2D/LOC101801641/MAP3K8/BMPR2/TSSK2/MAP2K1/CDK10/LOC101797327/PXK	33	12	101801853/101793152/101802614/101802855/101799089/101791519/101799561/101800595/101804452/101801648/101793707/101797327	21	101793905/101803371/101797616/101803562/101803181/101791030/101804342/101800942/101802737/101793068/101795713/101790169/101792645/101797446/101795633/101801641/101791769/106017602/101791037/101796806/101789868

MF	GO:0008237	metallopeptidase activity	9/543	84/6527	0.26249	0.939257	101791511/101794528/101794584/101804534/101800115/119714444/101804308/101790574/101800508	PHEX/LOC101794528/ADAMTS17/LOC101804534/ADAMTS18/ADAMTS19/MMP23B/ADAMTS5/LOC101800508	9	6	101791511/101804534/101800115/119714444/101804308/101800508	3	101794528/101794584/101790574
MF	GO:0016209	antioxidant activity	2/543	12/6527	0.263332	0.939257	101794631/101804478	LOC101794631/PRDX6	2	1	101804478	1	101794631
MF	GO:0016772	transferase activity, transferring phosphorus-containing groups	43/543	471/6527	0.278231	0.939257	101801853/101793905/101793152/101802614/101803371/101795985/101802855/101797616/101799089/101795498/101803562/101803181/101791030/101804342/101800366/101791519/101799561/101800942/101792149/101802737/101793068/101795713/101790169/101793407/101790726/101792645/101797446/101800595/101804452/101795633/110351624/101801648/101801641/101791769/101793707/101801143/106017602/101792961/101791037/101796806/101797078/101797327/101789868	TNIK/ULK2/WNK2/PRKG1/MAP2K3/LOC101795985/CAMK1G/LOC101797616/MAST4/PIP4K2A/TRIB1/MAP3K6/STYK1/SIK1/TENT5B/MAP3K15/PRKAA2/RPS6KA1/POLR2B/CLK1/PHKG1/KALRN/LOC101790169/TENT5C/PKM/PIM3/OXSR1/RNASEL/MUSK/RAF1/LOC110351624/CAMK2D/LOC101801641/MAP3K8/BMPR2/DGKI/TSSK2/TRNT1/MAP2K1/CDK10/IP6K2/LOC101797327/PXK	43	20	101801853/101793152/101802614/101802855/101799089/101795498/101800366/101791519/101799561/101792149/101793407/101790726/101800595/101804452/110351624/101801648/101793707/101801143/101792961/101797327	23	101793905/101803371/101795985/101797616/101803562/101803181/101791030/101804342/101800942/101802737/101793068/101795713/101790169/101792645/101797446/101795633/101801641/101791769/106017602/101791037/101796806/101797078/101789868
MF	GO:0022857	transmembrane transporter activity	31/543	333/6527	0.278688	0.939257	101798538/106020272/119714187/101802086/101795864/119713349/119715418/101801744/101805016/101791662/101804595/101800466/101790476/101793897/119717511/119718656/119714948/101801311/101804200/101795056/101801967/101804688/101795086/101799219/101799054/101798462/119713969/101797930/101802999/101790402/101800270	AQP4/CHRNG/KCNN1/AQP9/KCNE4/SLC52A3/KCNH2/KCNH6/SLC7A2/SLC9A6/SLC39A13/CHRNA10/ABCB6/ATP6V1E1/ASIC4/LOC119718656/LOC119714948/LOC101801311/SLC15A4/SLC6A1/SLC2A12/ABCC8/KCNQ4/SLC22A4/SCN5A/GABRG3/KCNC4/TMEM38A/ABCC1/ATP5PO/KCNH7	31	23	101798538/106020272/119714187/101802086/101795864/119713349/119715418/101801744/101805016/101790476/119717511/119714948/101801311/101795056/101801967/101804688/101799219/101799054/101798462/101797930/101802999/101790402/101800270	8	101791662/101804595/101800466/101793897/119718656/101804200/101795086/119713969
MF	GO:0008194	UDP-glycosyltransferase activity	3/543	23/6527	0.298608	0.939257	101799131/101796875/101792011	CSGALNACT1/GCNT1/UGGT2	3	2	101799131/101792011	1	101796875
MF	GO:0016853	isomerase activity	6/543	55/6527	0.306291	0.939257	101798581/101790428/101795379/101790798/101797601/101802609	PGM2L1/PGM1/PGM5/TPI1/GPI/PPIF	6	6	101798581/101790428/101795379/101790798/101797601/101802609	0	
MF	GO:0020037	heme binding	6/543	56/6527	0.321336	0.939257	101794631/101792573/101792912/101796516/101791081/101801058	LOC101794631/LOC101792573/LOC101792912/LOC101796516/LOC101791081/LOC101801058	6	4	101792912/101796516/101791081/101801058	2	101794631/101792573

MF	GO:0005215	transporter activity	33/543	364/6527	0.325418	0.939257	101798538/106020272/119714187/101802251/101802086/101795864/119713349/119715418/101801744/101805016/101791662/101804595/101800466/101790476/101793897/119717511/119718656/119714948/101801311/101804200/101795056/101801967/101804688/101795086/101798131/101799219/101799054/101798462/119713969/101797930/101802999/101790402/101800270	AQP4/CHRNG/KCNN1/PITPNM1/AQP9/KCNE4/SLC52A3/KCNH2/KCNH6/SLC7A2/SLC9A6/SLC39A13/CHRNA10/ABCB6/ATP6V1E1/ASIC4/LOC119718656/LOC119714948/LOC101801311/SLC15A4/SLC6A1/SLC2A12/ABCC8/KCNQ4/PITPNC1/SLC22A4/SCN5A/GABRG3/KCNC4/TMEM38A/ABCC1/ATP5PO/KCNH7	33	24	101798538/106020272/119714187/101802086/101795864/119713349/119715418/101801744/101805016/101790476/119717511/119714948/101801311/101795056/101801967/101804688/101798131/101799219/101799054/101798462/101797930/101802999/101790402/101800270	9	101802251/101791662/101804595/101800466/101793897/119718656/101804200/101795086/119713969
MF	GO:0008373	sialyltransferase activity	2/543	14/6527	0.32707	0.939257	101793440/101800484	ST6GALNAC6/ST8SIA6	2	2	101793440/101800484	0	
MF	GO:0016849	phosphorus-oxygen lyase activity	2/543	14/6527	0.32707	0.939257	101803512/101796516	ADCY2/LOC101796516	2	2	101803512/101796516	0	
MF	GO:0015078	proton transmembrane transporter activity	4/543	35/6527	0.331876	0.939257	101791662/101793897/119714948/101790402	SLC9A6/ATP6V1E1/LOC119714948/ATP5PO	4	2	119714948/101790402	2	101791662/101793897
MF	GO:0015318	inorganic molecular entity transmembrane transporter activity	20/543	215/6527	0.332788	0.939257	106020272/119714187/101795864/119715418/101801744/101791662/101804595/101800466/101793897/119717511/119714948/101801311/101795056/101795086/101799054/101798462/119713969/101797930/101790402/101800270	CHRNG/KCNN1/KCNE4/KCNH2/KCNH6/SLC9A6/SLC39A13/CHRNA10/ATP6V1E1/ASIC4/LOC119714948/LOC101801311/SLC6A1/KCNQ4/SCN5A/GABRG3/KCNC4/TMEM38A/ATP5PO/KCNH7	20	14	106020272/119714187/101795864/119715418/101801744/119717511/119714948/101801311/101795056/101799054/101798462/101797930/101790402/101800270	6	101791662/101804595/101800466/101793897/101795086/119713969
MF	GO:0015081	sodium ion transmembrane transporter activity	3/543	25/6527	0.34571	0.939257	119717511/101795056/101799054	ASIC4/SLC6A1/SCN5A	3	3	119717511/101795056/101799054	0	
MF	GO:0008233	peptidase activity	24/543	263/6527	0.346696	0.939257	101791511/101803168/101794528/101799480/101794584/101792575/101804552/101804246/113839605/101794820/101791397/101804534/101797901/101794268/101800115/119714444/101791234/101797577/101794402/101803700/101804308/101790574/101792943/101800508	PHEX/CTSL/LOC101794528/UCHL1/ADAMTS17/USP44/PLAU/CTSZ/LOC113839605/LOC101794820/DPP7/LOC101804534/DPEP1/CTSB/ADAMTS18/ADAMTS19/USP49/SCPEP1/TINAG/PSMB1/MMP23B/ADAMTS5/PIGK/LOC101800508	24	8	101791511/101804534/101797901/101800115/119714444/101804308/101792943/101800508	16	101803168/101794528/101799480/101794584/101792575/101804552/101804246/113839605/101794820/101791397/101794268/101791234/101797577/101794402/101803700/101790574
MF	GO:0070011	peptidase activity, acting on L-amino acid peptides	23/543	252/6527	0.350849	0.939257	101791511/101803168/101794528/101799480/101794584/101792575/101804552/101804246/113839605/101794820/101791397/101804534/101797901/101794268/101800115/119714444/101791234/101797577/101794402/101803700/101804308/101790574/101800508	PHEX/CTSL/LOC101794528/UCHL1/ADAMTS17/USP44/PLAU/CTSZ/LOC113839605/LOC101794820/DPP7/LOC101804534/DPEP1/CTSB/ADAMTS18/ADAMTS19/USP49/SCPEP1/TINAG/PSMB1/MMP23B/ADAMTS5/LOC101800508	23	7	101791511/101804534/101797901/101800115/119714444/101804308/101800508	16	101803168/101794528/101799480/101794584/101792575/101804552/101804246/113839605/101794820/101791397/101794268/101791234/101797577/101794402/101803700/101790574

MF	GO:0046906	tetrapyrrole binding	6/543	58/6527	0.351714	0.939257	101794631/101792573/101792912/101796516/101791081/101801058	LOC101794631/LOC101792573/LOC101792912/LOC101796516/LOC101791081/LOC101801058	6	4	101792912/101796516/101791081/101801058	2	101794631/101792573
MF	GO:0050662	coenzyme binding	10/543	103/6527	0.352847	0.939257	101798008/101790158/101803853/101797675/101798440/101790101/101789745/119714639/101789907/101794024	GADL1/HIBADH/ME1/D2HGDH/LOC101798440/MOCOS/ME3/ACADM/IVD/OAT	10	5	101798008/101803853/101797675/101790101/119714639	5	101790158/101798440/101789745/101789907/101794024
MF	GO:0005102	signaling receptor binding	14/543	149/6527	0.356959	0.939257	101795448/101791711/101795038/101800985/101803050/101800742/101803777/101793597/101799111/101799824/101792356/101803502/101799859/101796903	INHBA/WNT4/CX3CL1/LAMA4/CRH/MSTN/TNFSF13B/IGF1/IGF2/WNT2/WNT5B/PDGFA/RAPSN/LOC101796903	14	8	101795448/101791711/101795038/101800985/101800742/101793597/101799111/101803502	6	101803050/101803777/101799824/101792356/101799859/101796903
MF	GO:0005267	potassium channel activity	2/543	15/6527	0.358434	0.939257	119714187/101795864	KCNN1/KCNE4	2	2	119714187/101795864	0	
MF	GO:0005539	glycosaminoglycan binding	2/543	15/6527	0.358434	0.939257	101798168/101795277	TNFAIP6/APLP2	2	1	101795277	1	101798168
MF	GO:0015079	potassium ion transmembrane transporter activity	2/543	15/6527	0.358434	0.939257	119714187/101795864	KCNN1/KCNE4	2	2	119714187/101795864	0	
MF	GO:0019199	transmembrane receptor protein kinase activity	2/543	15/6527	0.358434	0.939257	101801641/101793707	LOC101801641/BMPR2	2	1	101793707	1	101801641
MF	GO:0008324	cation transmembrane transporter activity	11/543	116/6527	0.370791	0.939257	119714187/101795864/101791662/101804595/101793897/119717511/119714948/101795056/101799054/101797930/101790402	KCNN1/KCNE4/SLC9A6/SLC39A13/ATP6V1E1/ASIC4/LOC119714948/SLC6A1/SCN5A/TMEM38A/ATP5PO	11	8	119714187/101795864/119717511/119714948/101795056/101799054/101797930/101790402	3	101791662/101804595/101793897
MF	GO:0030170	pyridoxal phosphate binding	3/543	27/6527	0.392507	0.939257	101798008/101790101/101794024	GADL1/MOCOS/OAT	3	2	101798008/101790101	1	101794024
MF	GO:0070279	vitamin B6 binding	3/543	27/6527	0.392507	0.939257	101798008/101790101/101794024	GADL1/MOCOS/OAT	3	2	101798008/101790101	1	101794024
MF	GO:0016746	transferase activity, transferring acyl groups	5/543	50/6527	0.403641	0.939257	101797414/101802263/101801215/101803103/101791052	LOC101797414/LPCAT2/DGAT2/GPAM/ACAA2	5	2	101803103/101791052	3	101797414/101802263/101801215
MF	GO:0008238	exopeptidase activity	3/543	28/6527	0.415601	0.939257	101794820/101797901/101797577	LOC101794820/DPEP1/SCPEP1	3	1	101797901	2	101794820/101797577
MF	GO:0017048	Rho GTPase binding	5/543	51/6527	0.420594	0.939257	101795713/101792952/101799876/101790388/101792679	KALRN/ARHGEF3/LOC101799876/ARHGEF17/PLEKHG1	5	4	101792952/101799876/101790388/101792679	1	101795713
MF	GO:0015075	ion transmembrane transporter activity	21/543	238/6527	0.421793	0.939257	106020272/119714187/101795864/119713349/119715418/101801744/101791662/101804595/101800466/101793897/119717511/119714948/101801311/101795056/101795086/101799054/101798462/119713969/101797930/101790402/101800270	CHRNG/KCNN1/KCNE4/SLC52A3/KCNH2/KCNH6/SLC9A6/SLC39A13/CHRNA10/ATP6V1E1/ASIC4/LOC119714948/LOC101801311/SLC6A1/KCNQ4/SCN5A/GABRG3/KCNC4/TMEM38A/ATP5PO/KCNH7	21	15	106020272/119714187/101795864/119713349/119715418/101801744/119717511/119714948/101801311/101795056/101799054/101798462/101797930/101790402/101800270	6	101791662/101804595/101800466/101793897/101795086/119713969
MF	GO:0004553	hydrolase activity, hydrolyzing O-glycosyl compounds	4/543	40/6527	0.429026	0.939257	113845291/113840111/101792144/101800183	LOC113845291/LOC113840111/FUCA2/HYAL2	4	2	113845291/113840111	2	101792144/101800183
MF	GO:0016705	oxidoreductase activity, acting on paired donors, with incorporation or reduction of molecular oxygen	5/543	52/6527	0.437461	0.939257	101805057/101792573/101792912/101791081/101801058	HMOX1/LOC101792573/LOC101792912/LOC101791081/LOC101801058	5	3	101792912/101791081/101801058	2	101805057/101792573

MF	GO:0004930	G-protein-coupled receptor activity	18/543	206/6527	0.449846	0.939257	106020638/101789607/101789648/101796159/101803645/101804355/101793595/101802295/101803152/101802610/101797638/101793610/101790005/101803029/101794938/101801352/101804306/101801041	APLNR/HTR5A/GPR157/LOC101796159/ADGRL3/GPR176/LPAR3/UTS2R/GPR161/CCR9/LOC101797638/GPR182/LOC101790005/CELSR1/ADGRD1/CNR1/PTGFR/VIPR2	18	13	106020638/101789607/101796159/101803645/101804355/101793595/101802295/101803152/101797638/101793610/101803029/101801352/101804306	5	101789648/101802610/101790005/101794938/101801041
MF	GO:0030234	enzyme regulator activity	9/543	101/6527	0.466193	0.939257	101792619/101794822/101796096/101794158/101804490/101790881/101793795/101791694/101795277	TIMP4/TFPI/LOC101796096/COL6A3/GRPEL2/KNG1/RANGAP1/CDKN1B/APLP2	9	4	101794158/101804490/101790881/101795277	5	101792619/101794822/101796096/101793795/101791694
MF	GO:0003779	actin binding	5/543	54/6527	0.470832	0.939257	101793729/101791895/101799876/101797439/101789868	TWF2/LOC101791895/LOC101799876/SHROOM1/PXK	5	2	101799876/101797439	3	101793729/101791895/101789868
MF	GO:0005179	hormone activity	3/543	31/6527	0.482924	0.939257	101803050/101793597/101799111	CRH/IGF1/IGF2	3	2	101793597/101799111	1	101803050
MF	GO:0046873	metal ion transmembrane transporter activity	6/543	67/6527	0.488389	0.939257	119714187/101795864/101804595/119717511/101795056/101799054	KCNN1/KCNE4/SLC39A13/ASIC4/SLC6A1/SCN5A	6	5	119714187/101795864/119717511/101795056/101799054	1	101804595
MF	GO:0016874	ligase activity	6/543	69/6527	0.517748	0.939257	119714249/101801928/101799450/101797455/101796914/101795091	LOC119714249/ADSS1/GLUL/ASNS/LIG1/TARS3	6	3	119714249/101801928/101795091	3	101799450/101797455/101796914
MF	GO:0005088	Ras guanyl-nucleotide exchange factor activity	4/543	45/6527	0.522116	0.939257	101795713/101792952/101790388/101792679	KALRN/ARHGEF3/ARHGEF17/PLEKHG1	4	3	101792952/101790388/101792679	1	101795713
MF	GO:0005089	Rho guanyl-nucleotide exchange factor activity	4/543	45/6527	0.522116	0.939257	101795713/101792952/101790388/101792679	KALRN/ARHGEF3/ARHGEF17/PLEKHG1	4	3	101792952/101790388/101792679	1	101795713
MF	GO:0004888	transmembrane signaling receptor activity	21/543	252/6527	0.531195	0.939257	106020638/101789607/101789648/101796159/101803645/101804355/101793595/101802295/101791406/101803152/101802610/101797638/101793610/101790005/101803029/101794938/101801641/101793707/101801352/101804306/101801041	APLNR/HTR5A/GPR157/LOC101796159/ADGRL3/GPR176/LPAR3/UTS2R/CD247/GPR161/CCR9/LOC101797638/GPR182/LOC101790005/CELSR1/ADGRD1/LOC101801641/BMPR2/CNR1/PTGFR/VIPR2	21	14	106020638/101789607/101796159/101803645/101804355/101793595/101802295/101803152/101797638/101793610/101803029/101793707/101801352/101804306	7	101789648/101791406/101802610/101790005/101794938/101801641/101801041
MF	GO:0004540	ribonuclease activity	2/543	21/6527	0.531452	0.939257	119715945/101800595	LOC119715945/RNASEL	2	1	101800595	1	119715945
MF	GO:0009055	electron transfer activity	2/543	21/6527	0.531452	0.939257	101795743/101805119	NDUFAF2/NDUFS1	2	1	101805119	1	101795743
MF	GO:0016757	transferase activity, transferring glycosyl groups	10/543	119/6527	0.535447	0.939257	101803248/101801173/101799131/101793948/101796875/101793440/101800656/119716831/101792011/101800484	B3GALNT2/GLT8D2/CSGALNACT1/B3GNT2/GCNT1/ST6GALNAC6/LOC101800656/LOC119716831/UGGT2/ST8SIA6	10	6	101801173/101799131/101793440/101800656/101792011/101800484	4	101803248/101793948/101796875/119716831
MF	GO:0016758	transferase activity, transferring hexosyl groups	5/543	58/6527	0.53546	0.939257	101803248/101799131/101793948/101796875/101792011	B3GALNT2/CSGALNACT1/B3GNT2/GCNT1/UGGT2	5	2	101799131/101792011	3	101803248/101793948/101796875
MF	GO:0005506	iron ion binding	4/543	46/6527	0.539882	0.939257	101792573/101792912/101791081/101801058	LOC101792573/LOC101792912/LOC101791081/LOC101801058	4	3	101792912/101791081/101801058	1	101792573
MF	GO:0019842	vitamin binding	3/543	34/6527	0.546333	0.939257	101798008/101790101/101794024	GADL1/MOCOS/OAT	3	2	101798008/101790101	1	101794024
MF	GO:0140097	catalytic activity, acting on DNA	3/543	34/6527	0.546333	0.939257	101790058/101796914/101797870	BLM/LIG1/XRCC5	3	2	101790058/101797870	1	101796914
MF	GO:0005126	cytokine receptor binding	2/543	22/6527	0.557081	0.939257	101795038/101803777	CX3CL1/TNFSF13B	2	1	101795038	1	101803777

MF	GO:0016747	transferase activity, transferring acyl groups other than amino-acyl groups	2/543	22/6527	0.557081	0.939257	101801215/101791052	DGAT2/ACAA2	2	1	101791052	1	101801215
MF	GO:0017016	Ras GTPase binding	5/543	60/6527	0.5664	0.939257	101795713/101792952/101799876/101790388/101792679	KALRN/ARHGEF3/LOC101799876/ARHGEF17/PLEKHG1	5	4	101792952/101799876/101790388/101792679	1	101795713
MF	GO:0031267	small GTPase binding	5/543	60/6527	0.5664	0.939257	101795713/101792952/101799876/101790388/101792679	KALRN/ARHGEF3/LOC101799876/ARHGEF17/PLEKHG1	5	4	101792952/101799876/101790388/101792679	1	101795713
MF	GO:0050660	flavin adenine dinucleotide binding	3/543	35/6527	0.566439	0.939257	101797675/119714639/101789907	D2HGDH/ACADM/IVD	3	2	101797675/119714639	1	101789907
MF	GO:0001067	regulatory region nucleic acid binding	1/543	10/6527	0.580717	0.939257	101799633	IRF1	1	0		1	101799633
MF	GO:0003713	transcription coactivator activity	1/543	10/6527	0.580717	0.939257	101801468	CBFB	1	0		1	101801468
MF	GO:0008375	acetylglucosaminyltransferase activity	1/543	10/6527	0.580717	0.939257	101796875	GCNT1	1	0		1	101796875
MF	GO:0016278	lysine N-methyltransferase activity	1/543	10/6527	0.580717	0.939257	101791765	EZH1	1	1	101791765	0	
MF	GO:0016279	protein-lysine N-methyltransferase activity	1/543	10/6527	0.580717	0.939257	101791765	EZH1	1	1	101791765	0	
MF	GO:0016891	endoribonuclease activity, producing 5'-phosphomonoesters	1/543	10/6527	0.580717	0.939257	119715945	LOC119715945	1	0		1	119715945
MF	GO:0018024	histone-lysine N-methyltransferase activity	1/543	10/6527	0.580717	0.939257	101791765	EZH1	1	1	101791765	0	
MF	GO:0019887	protein kinase regulator activity	1/543	10/6527	0.580717	0.939257	101791694	CDKN1B	1	0		1	101791694
MF	GO:0042054	histone methyltransferase activity	1/543	10/6527	0.580717	0.939257	101791765	EZH1	1	1	101791765	0	
MF	GO:0044212	transcription regulatory region DNA binding	1/543	10/6527	0.580717	0.939257	101799633	IRF1	1	0		1	101799633
MF	GO:0008092	cytoskeletal protein binding	11/543	136/6527	0.585257	0.939257	101793729/101791895/101796751/101790443/119714941/106020577/101799876/101803768/101797439/101797044/101789868	TWF2/LOC101791895/DST/KIF26A/LOC119714941/KIF3C/LOC101799876/KIF16B/SHROOM1/LMOD2/PXK	11	5	101796751/101790443/106020577/101799876/101797439	6	101793729/101791895/119714941/101803768/101797044/101789868
MF	GO:0016798	hydrolase activity, acting on glycosyl bonds	4/543	49/6527	0.59106	0.939257	113845291/113840111/101792144/101800183	LOC113845291/LOC113840111/FUCA2/HYAL2	4	2	113845291/113840111	2	101792144/101800183
MF	GO:0008017	microtubule binding	5/543	62/6527	0.596255	0.939257	101796751/101790443/119714941/106020577/101803768	DST/KIF26A/LOC119714941/KIF3C/KIF16B	5	3	101796751/101790443/106020577	2	119714941/101803768
MF	GO:0005044	scavenger receptor activity	2/543	24/6527	0.605293	0.939257	119716554/101800542	SCARA5/ENPP3	2	1	101800542	1	119716554
MF	GO:0038024	cargo receptor activity	2/543	24/6527	0.605293	0.939257	119716554/101800542	SCARA5/ENPP3	2	1	101800542	1	119716554
MF	GO:0051536	iron-sulfur cluster binding	2/543	24/6527	0.605293	0.939257	113845564/101805119	RSAD1/NDUFS1	2	2	113845564/101805119	0	
MF	GO:0051540	metal cluster binding	2/543	24/6527	0.605293	0.939257	113845564/101805119	RSAD1/NDUFS1	2	2	113845564/101805119	0	
MF	GO:0038023	signaling receptor activity	21/543	262/6527	0.606003	0.939257	106020638/101789607/101789648/101796159/101803645/101804355/101793595/101802295/101791406/101803152/101802610/101797638/101793610/101790005/101803029/101794938/101801641/101793707/101801352/101804306/101801041	APLNR/HTR5A/GPR157/LOC101796159/ADGRL3/GPR176/LPAR3/UTS2R/CD247/GPR161/CCR9/LOC101797638/GPR182/LOC101790005/CELSR1/ADGRD1/LOC101801641/BMPR2/CNR1/PTGFR/VIPR2	21	14	106020638/101789607/101796159/101803645/101804355/101793595/101802295/101803152/101797638/101793610/101803029/101793707/101801352/101804306	7	101789648/101791406/101802610/101790005/101794938/101801641/101801041
MF	GO:0060089	molecular transducer activity	21/543	262/6527	0.606003	0.939257	106020638/101789607/101789648/101796159/101803645/101804355/101793595/101802295/101791406/101803152/101802610/101797638/101793610/101790005/101803029/101794938/101801641/101793707/101801352/101804306/101801041	APLNR/HTR5A/GPR157/LOC101796159/ADGRL3/GPR176/LPAR3/UTS2R/CD247/GPR161/CCR9/LOC101797638/GPR182/LOC101790005/CELSR1/ADGRD1/LOC101801641/BMPR2/CNR1/PTGFR/VIPR2	21	14	106020638/101789607/101796159/101803645/101804355/101793595/101802295/101803152/101797638/101793610/101803029/101793707/101801352/101804306	7	101789648/101791406/101802610/101790005/101794938/101801641/101801041

MF	GO:0046914	transition metal ion binding	24/543	300/6527	0.613027	0.939257	101801981/101794528/101802816/101796941/101790101/101803111/101792575/101802147/101802225/101793204/101792573/101803240/101792912/119718292/101789572/101804534/119713599/101791234/101795277/101791081/101804308/101801058/101802075/119714007	ZFAND5/LOC101794528/DMD/TRIM71/MOCOS/TRIM9/USP44/L3MBTL3/TES/ZFAND1/LOC101792573/FBXO30/LOC101792912/LOC119718292/PPARA/LOC101804534/TRIM63/USP49/APLP2/LOC101791081/MMP23B/LOC101801058/MIB1/LOC119714007	24	12	101802816/101790101/101803111/101792912/101789572/101804534/101795277/101791081/101804308/101801058/101802075/119714007	12	101801981/101794528/101796941/101792575/101802147/101802225/101793204/101792573/101803240/119718292/119713599/101791234
MF	GO:0005540	hyaluronic acid binding	1/543	11/6527	0.615651	0.939257	101798168	TNFAIP6	1	0		1	101798168
MF	GO:0008009	chemokine activity	1/543	11/6527	0.615651	0.939257	101795038	CX3CL1	1	1	101795038	0	
MF	GO:0008170	N-methyltransferase activity	1/543	11/6527	0.615651	0.939257	101791765	EZH1	1	1	101791765	0	
MF	GO:0008276	protein methyltransferase activity	1/543	11/6527	0.615651	0.939257	101791765	EZH1	1	1	101791765	0	
MF	GO:0016701	oxidoreductase activity, acting on single donors with incorporation of molecular oxygen	1/543	11/6527	0.615651	0.939257	101798614	BCO1	1	1	101798614	0	
MF	GO:0016702	oxidoreductase activity, acting on single donors with incorporation of molecular oxygen, incorporation of two atoms of oxygen	1/543	11/6527	0.615651	0.939257	101798614	BCO1	1	1	101798614	0	
MF	GO:0016831	carboxy-lyase activity	1/543	11/6527	0.615651	0.939257	101798008	GADL1	1	1	101798008	0	
MF	GO:0016893	endonuclease activity, active with either ribo- or deoxyribonucleic acids and producing 5'-phosphomonoesters	1/543	11/6527	0.615651	0.939257	119715945	LOC119715945	1	0		1	119715945
MF	GO:0019207	kinase regulator activity	1/543	11/6527	0.615651	0.939257	101791694	CDKN1B	1	0		1	101791694
MF	GO:0022843	voltage-gated cation channel activity	1/543	11/6527	0.615651	0.939257	101795864	KCNE4	1	1	101795864	0	
MF	GO:0042379	chemokine receptor binding	1/543	11/6527	0.615651	0.939257	101795038	CX3CL1	1	1	101795038	0	
MF	GO:0140101	catalytic activity, acting on a tRNA	4/543	51/6527	0.623252	0.939257	119714249/101803288/119716581/101795091	LOC119714249/LOC101803288/LOC119716581/TARS3	4	3	119714249/119716581/101795091	1	101803288
MF	GO:0005230	extracellular ligand-gated ion channel activity	3/543	38/6527	0.62336	0.939257	106020272/101800466/101798462	CHRNG/CHRNA10/GABRG3	3	2	106020272/101798462	1	101800466
MF	GO:0035091	phosphatidylinositol binding	3/543	38/6527	0.62336	0.939257	101792242/101803768/101789868	SNX16/KIF16B/PXK	3	0		3	101792242/101803768/101789868
MF	GO:0008270	zinc ion binding	18/543	228/6527	0.629378	0.939257	101801981/101794528/101802816/101796941/101803111/101792575/101802147/101802225/101793204/101803240/119718292/101789572/101804534/119713599/101791234/101804308/101802075/119714007	ZFAND5/LOC101794528/DMD/TRIM71/TRIM9/USP44/L3MBTL3/TES/ZFAND1/FBXO30/LOC119718292/PPARA/LOC101804534/TRIM63/USP49/MMP23B/MIB1/LOC119714007	18	7	101802816/101803111/101789572/101804534/101804308/101802075/119714007	11	101801981/101794528/101796941/101792575/101802147/101802225/101793204/101803240/119718292/119713599/101791234
MF	GO:0030545	receptor regulator activity	8/543	103/6527	0.633601	0.939257	101795448/101795038/101803050/101800742/101793597/101799111/101803502/101796903	INHBA/CX3CL1/CRH/MSTN/IGF1/IGF2/PDGFA/LOC101796903	8	6	101795448/101795038/101800742/101793597/101799111/101803502	2	101803050/101796903
MF	GO:0048018	receptor ligand activity	8/543	103/6527	0.633601	0.939257	101795448/101795038/101803050/101800742/101793597/101799111/101803502/101796903	INHBA/CX3CL1/CRH/MSTN/IGF1/IGF2/PDGFA/LOC101796903	8	6	101795448/101795038/101800742/101793597/101799111/101803502	2	101803050/101796903

MF	GO:0004871	signal transducer activity	21/543	266/6527	0.634509	0.939257	106020638/101789607/101789648/101796159/101803645/101804355/101793595/101802295/101791406/101803152/101802610/101797638/101793610/101790005/101803029/101794938/101801641/101793707/101801352/101804306/101801041	APLNR/HTR5A/GPR157/LOC101796159/ADGRL3/GPR176/LPAR3/UTS2R/CD247/GPR161/CCR9/LOC101797638/GPR182/LOC101790005/CELSR1/ADGRD1/LOC101801641/BMPR2/CNR1/PTGFR/VIPR2	21	14	106020638/101789607/101796159/101803645/101804355/101793595/101802295/101803152/101797638/101793610/101803029/101793707/101801352/101804306	7	101789648/101791406/101802610/101790005/101794938/101801641/101801041
MF	GO:0004298	threonine-type endopeptidase activity	1/543	12/6527	0.647681	0.939257	101803700	PSMB1	1	0		1	101803700
MF	GO:0004521	endoribonuclease activity	1/543	12/6527	0.647681	0.939257	119715945	LOC119715945	1	0		1	119715945
MF	GO:0008484	sulfuric ester hydrolase activity	1/543	12/6527	0.647681	0.939257	101802979	ARSI	1	1	101802979	0	
MF	GO:0051213	dioxygenase activity	1/543	12/6527	0.647681	0.939257	101798614	BCO1	1	1	101798614	0	
MF	GO:0070003	threonine-type peptidase activity	1/543	12/6527	0.647681	0.939257	101803700	PSMB1	1	0		1	101803700
MF	GO:1901681	sulfur compound binding	1/543	12/6527	0.647681	0.939257	101795277	APLP2	1	1	101795277	0	
MF	GO:0004386	helicase activity	2/543	26/6527	0.649422	0.939257	101790058/101797870	BLM/XRCC5	2	2	101790058/101797870	0	
MF	GO:0015291	secondary active transmembrane transporter activity	2/543	26/6527	0.649422	0.939257	101791662/101795056	SLC9A6/SLC6A1	2	1	101795056	1	101791662
MF	GO:0022836	gated channel activity	5/543	66/6527	0.652349	0.939257	106020272/119714187/101795864/101800466/101798462	CHRNG/KCNN1/KCNE4/CHRNA10/GABRG3	5	4	106020272/119714187/101795864/101798462	1	101800466
MF	GO:0022839	ion gated channel activity	5/543	66/6527	0.652349	0.939257	106020272/119714187/101795864/101800466/101798462	CHRNG/KCNN1/KCNE4/CHRNA10/GABRG3	5	4	106020272/119714187/101795864/101798462	1	101800466
MF	GO:0016779	nucleotidyltransferase activity	4/543	53/6527	0.653797	0.939257	101800366/101792149/101793407/101792961	TENT5B/POLR2B/TENT5C/TRNT1	4	4	101800366/101792149/101793407/101792961	0	
MF	GO:0015276	ligand-gated ion channel activity	3/543	40/6527	0.65836	0.939257	106020272/101800466/101798462	CHRNG/CHRNA10/GABRG3	3	2	106020272/101798462	1	101800466
MF	GO:0022834	ligand-gated channel activity	3/543	40/6527	0.65836	0.939257	106020272/101800466/101798462	CHRNG/CHRNA10/GABRG3	3	2	106020272/101798462	1	101800466
MF	GO:0008083	growth factor activity	4/543	54/6527	0.668434	0.939257	101795448/101800742/101803502/101796903	INHBA/MSTN/PDGFA/LOC101796903	4	3	101795448/101800742/101803502	1	101796903
MF	GO:0016763	transferase activity, transferring pentosyl groups	2/543	27/6527	0.669975	0.939257	101800656/119716831	LOC101800656/LOC119716831	2	1	101800656	1	119716831
MF	GO:0003743	translation initiation factor activity	1/543	13/6527	0.677045	0.939257	101793553	EIF1	1	0		1	101793553
MF	GO:0016830	carbon-carbon lyase activity	1/543	13/6527	0.677045	0.939257	101798008	GADL1	1	1	101798008	0	
MF	GO:0051015	actin filament binding	1/543	13/6527	0.677045	0.939257	101797439	SHROOM1	1	1	101797439	0	
MF	GO:0098772	molecular function regulator	21/543	275/6527	0.69486	0.939257	101795448/101795038/101792619/101794822/101803050/101800742/101796096/101795713/101792952/101794158/101804490/101790881/101793597/101799111/101793795/101791694/101790388/101803502/101795277/101796903/101792679	INHBA/CX3CL1/TIMP4/TFPI/CRH/MSTN/LOC101796096/KALRN/ARHGEF3/COL6A3/GRPEL2/KNG1/IGF1/IGF2/RANGAP1/CDKN1B/ARHGEF17/PDGFA/APLP2/LOC101796903/PLEKHG1	21	13	101795448/101795038/101800742/101792952/101794158/101804490/101790881/101793597/101799111/101790388/101803502/101795277/101792679	8	101792619/101794822/101803050/101796096/101795713/101793795/101791694/101796903
MF	GO:0004175	endopeptidase activity	11/543	148/6527	0.697305	0.939257	101791511/101794528/101794584/101804552/101794268/101800115/119714444/101803700/101804308/101790574/101800508	PHEX/LOC101794528/ADAMTS17/PLAU/CTSB/ADAMTS18/ADAMTS19/PSMB1/MMP23B/ADAMTS5/LOC101800508	11	5	101791511/101800115/119714444/101804308/101800508	6	101794528/101794584/101804552/101794268/101803700/101790574
MF	GO:0001664	G protein-coupled receptor binding	1/543	14/6527	0.703966	0.939257	101795038	CX3CL1	1	1	101795038	0	
MF	GO:0003690	double-stranded DNA binding	1/543	14/6527	0.703966	0.939257	101802532	MSH2	1	0		1	101802532
MF	GO:0005326	neurotransmitter transporter activity	1/543	14/6527	0.703966	0.939257	101795056	SLC6A1	1	1	101795056	0	
MF	GO:0005328	neurotransmitter:sodium symporter activity	1/543	14/6527	0.703966	0.939257	101795056	SLC6A1	1	1	101795056	0	

MF	GO:0015294	solute:cation symporter activity	1/543	14/6527	0.703966	0.939257	101795056	SLC6A1	1	1	101795056	0	
MF	GO:0015370	solute:sodium symporter activity	1/543	14/6527	0.703966	0.939257	101795056	SLC6A1	1	1	101795056	0	
MF	GO:0019783	ubiquitin-like protein-specific protease activity	3/543	43/6527	0.706352	0.939257	101799480/101792575/101791234	UCHL1/USP44/USP49	3	0		3	101799480/101792575/101791234
MF	GO:0036459	thiol-dependent ubiquitinyl hydrolase activity	3/543	43/6527	0.706352	0.939257	101799480/101792575/101791234	UCHL1/USP44/USP49	3	0		3	101799480/101792575/101791234
MF	GO:0101005	ubiquitinyl hydrolase activity	3/543	43/6527	0.706352	0.939257	101799480/101792575/101791234	UCHL1/USP44/USP49	3	0		3	101799480/101792575/101791234
MF	GO:0005509	calcium ion binding	20/543	265/6527	0.711733	0.941324	101790249/101794730/101802263/101792273/101795371/101800869/101791530/113840667/101793322/101796751/101790966/101795002/101803543/101803002/101803029/101789534/101800667/101789987/101796230/101794062	ANXA1/ANXA11/LPCAT2/ANXA2/LOC101795371/ANXA7/PLS3/LOC113840667/THBS1/DST/PLCB1/EGF/RASEF/PCDH7/CELSR1/CDHR5/CDH2/HMCN1/CDH8/SUSD1	20	7	113840667/101796751/101790966/101795002/101803002/101803029/101796230	13	101790249/101794730/101802263/101792273/101795371/101800869/101791530/101793322/101803543/101789534/101800667/101789987/101794062
MF	GO:0015631	tubulin binding	5/543	72/6527	0.726654	0.943406	101796751/101790443/119714941/106020577/101803768	DST/KIF26A/LOC119714941/KIF3C/KIF16B	5	3	101796751/101790443/106020577	2	119714941/101803768
MF	GO:0140098	catalytic activity, acting on RNA	7/543	99/6527	0.727719	0.943406	119715945/119714249/101803288/119716581/101792149/101800595/101795091	LOC119715945/LOC119714249/LOC101803288/LOC119716581/POLR2B/RNASEL/TARS3	7	5	119714249/119716581/101792149/101800595/101795091	2	119715945/101803288
MF	GO:0031406	carboxylic acid binding	1/543	15/6527	0.728647	0.943406	101798168	TNFAIP6	1	0		1	101798168
MF	GO:0043177	organic acid binding	1/543	15/6527	0.728647	0.943406	101798168	TNFAIP6	1	0		1	101798168
MF	GO:0004114	3',5'-cyclic-nucleotide phosphodiesterase activity	1/543	16/6527	0.751274	0.948966	101796581	PDE9A	1	1	101796581	0	
MF	GO:0008757	S-adenosylmethionine-dependent methyltransferase activity	1/543	16/6527	0.751274	0.948966	101791765	EZH1	1	1	101791765	0	
MF	GO:0015293	symporter activity	1/543	16/6527	0.751274	0.948966	101795056	SLC6A1	1	1	101795056	0	
MF	GO:0051082	unfolded protein binding	1/543	16/6527	0.751274	0.948966	101801226	DNAJA4	1	0		1	101801226
MF	GO:0003700	DNA-binding transcription factor activity	12/543	168/6527	0.752229	0.948966	101805187/101790639/106019185/101801698/101794314/119714375/101797962/101789572/101796626/101801576/113845655/119714007	ATF3/CREM/CEBPB/FOS/NFIL3/LOC119714375/CREB5/PPARA/JUN/E2F1/JUND/LOC119714007	12	4	119714375/101789572/101801576/119714007	8	101805187/101790639/106019185/101801698/101794314/101797962/101796626/113845655
MF	GO:0004112	cyclic-nucleotide phosphodiesterase activity	1/543	17/6527	0.772017	0.959172	101796581	PDE9A	1	1	101796581	0	
MF	GO:0004180	carboxypeptidase activity	1/543	17/6527	0.772017	0.959172	101797577	SCPEP1	1	0		1	101797577
MF	GO:0004519	endonuclease activity	1/543	17/6527	0.772017	0.959172	119715945	LOC119715945	1	0		1	119715945
MF	GO:0004842	ubiquitin-protein transferase activity	2/543	34/6527	0.787635	0.968791	101795149/101802075	LOC101795149/MIB1	2	1	101802075	1	101795149
MF	GO:0019787	ubiquitin-like protein transferase activity	2/543	34/6527	0.787635	0.968791	101795149/101802075	LOC101795149/MIB1	2	1	101802075	1	101795149
MF	GO:0005125	cytokine activity	1/543	19/6527	0.808466	0.973815	101795038	CX3CL1	1	1	101795038	0	
MF	GO:0140110	transcription regulator activity	14/543	205/6527	0.818251	0.973815	101805187/101801468/101790639/106019185/101801698/101794314/119714375/101797962/101802147/101789572/101796626/101801576/113845655/119714007	ATF3/CBFB/CREM/CEBPB/FOS/NFIL3/LOC119714375/CREB5/L3MBTL3/PPARA/JUN/E2F1/JUND/LOC119714007	14	4	119714375/101789572/101801576/119714007	10	101805187/101801468/101790639/106019185/101801698/101794314/101797962/101802147/101796626/113845655
MF	GO:0005244	voltage-gated ion channel activity	1/543	20/6527	0.824446	0.973815	101795864	KCNE4	1	1	101795864	0	
MF	GO:0008135	translation factor activity, RNA binding	1/543	20/6527	0.824446	0.973815	101793553	EIF1	1	0		1	101793553
MF	GO:0022832	voltage-gated channel activity	1/543	20/6527	0.824446	0.973815	101795864	KCNE4	1	1	101795864	0	
MF	GO:0016741	transferase activity, transferring one-carbon groups	4/543	68/6527	0.828922	0.973815	101803074/101800220/101797486/101791765	ALDH1L2/TRMT9B/METTL7A/EZH1	4	3	101803074/101800220/101791765	1	101797486
MF	GO:0060589	nucleoside-triphosphatase regulator activity	2/543	38/6527	0.836855	0.973815	101804490/101793795	GRPEL2/RANGAP1	2	1	101804490	1	101793795
MF	GO:0003899	DNA-directed 5'-3' RNA polymerase activity	1/543	21/6527	0.839096	0.973815	101792149	POLR2B	1	1	101792149	0	

MF	GO:0034062	5'-3' RNA polymerase activity	1/543	21/6527	0.839096	0.973815	101792149	POLR2B	1	1	101792149	0	
MF	GO:0097747	RNA polymerase activity	1/543	21/6527	0.839096	0.973815	101792149	POLR2B	1	1	101792149	0	
MF	GO:0004518	nuclease activity	2/543	39/6527	0.847433	0.973815	119715945/101800595	LOC119715945/RNASEL	2	1	101800595	1	119715945
MF	GO:0004812	aminoacyl-tRNA ligase activity	2/543	39/6527	0.847433	0.973815	119714249/101795091	LOC119714249/TARS3	2	2	119714249/101795091	0	
MF	GO:0016875	ligase activity, forming carbon-oxygen bonds	2/543	39/6527	0.847433	0.973815	119714249/101795091	LOC119714249/TARS3	2	2	119714249/101795091	0	
MF	GO:0003755	peptidyl-prolyl cis-trans isomerase activity	1/543	22/6527	0.852525	0.973815	101802609	PPIF	1	1	101802609	0	
MF	GO:0016859	cis-trans isomerase activity	1/543	22/6527	0.852525	0.973815	101802609	PPIF	1	1	101802609	0	
MF	GO:0051020	GTPase binding	5/543	87/6527	0.860724	0.973815	101795713/101792952/101799876/101790388/101792679	KALRN/ARHGEF3/LOC101799876/ARHGEF17/PLEKHG1	5	4	101792952/101799876/101790388/101792679	1	101795713
MF	GO:0005085	guanyl-nucleotide exchange factor activity	4/543	72/6527	0.860778	0.973815	101795713/101792952/101790388/101792679	KALRN/ARHGEF3/ARHGEF17/PLEKHG1	4	3	101792952/101790388/101792679	1	101795713
MF	GO:0003924	GTPase activity	10/543	159/6527	0.862974	0.973815	101799698/119714718/101798141/101803543/119718018/101803065/101790303/101802364/101804925/101799739	DIRAS2/LOC119714718/RRAD/RASEF/LOC119718018/RASL11A/RAB39A/MRAS/RAB9A/RAB40B	10	3	101799698/101802364/101799739	7	119714718/101798141/101803543/119718018/101803065/101790303/101804925
MF	GO:0005096	GTPase activator activity	1/543	25/6527	0.886464	0.985835	101793795	RANGAP1	1	0		1	101793795
MF	GO:0008509	anion transmembrane transporter activity	1/543	25/6527	0.886464	0.985835	119713349	SLC52A3	1	1	119713349	0	
MF	GO:0008168	methyltransferase activity	3/543	62/6527	0.899355	0.985835	101800220/101797486/101791765	TRMT9B/METTL7A/EZH1	3	2	101800220/101791765	1	101797486
MF	GO:0019899	enzyme binding	5/543	101/6527	0.931316	0.985835	101795713/101792952/101799876/101790388/101792679	KALRN/ARHGEF3/LOC101799876/ARHGEF17/PLEKHG1	5	4	101792952/101799876/101790388/101792679	1	101795713
MF	GO:0000988	transcription factor activity, protein binding	1/543	32/6527	0.938356	0.985835	101801468	CBFB	1	0		1	101801468
MF	GO:0000989	transcription factor activity, transcription factor binding	1/543	32/6527	0.938356	0.985835	101801468	CBFB	1	0		1	101801468
MF	GO:0003712	transcription cofactor activity	1/543	32/6527	0.938356	0.985835	101801468	CBFB	1	0		1	101801468
MF	GO:0008146	sulfotransferase activity	1/543	33/6527	0.94351	0.985835	101793969	HS3ST3B1	1	1	101793969	0	
MF	GO:0030695	GTPase regulator activity	1/543	33/6527	0.94351	0.985835	101793795	RANGAP1	1	0		1	101793795
MF	GO:0001883	purine nucleoside binding	11/543	198/6527	0.947647	0.985835	101799698/101801928/101798141/119713234/101803543/119718018/101803065/101790303/101802364/101804925/101799739	DIRAS2/ADSS1/RRAD/LOC119713234/RASEF/LOC119718018/RASL11A/RAB39A/MRAS/RAB9A/RAB40B	11	5	101799698/101801928/119713234/101802364/101799739	6	101798141/101803543/119718018/101803065/101790303/101804925
MF	GO:0005525	GTP binding	11/543	198/6527	0.947647	0.985835	101799698/101801928/101798141/119713234/101803543/119718018/101803065/101790303/101802364/101804925/101799739	DIRAS2/ADSS1/RRAD/LOC119713234/RASEF/LOC119718018/RASL11A/RAB39A/MRAS/RAB9A/RAB40B	11	5	101799698/101801928/119713234/101802364/101799739	6	101798141/101803543/119718018/101803065/101790303/101804925
MF	GO:0032549	ribonucleoside binding	11/543	198/6527	0.947647	0.985835	101799698/101801928/101798141/119713234/101803543/119718018/101803065/101790303/101802364/101804925/101799739	DIRAS2/ADSS1/RRAD/LOC119713234/RASEF/LOC119718018/RASL11A/RAB39A/MRAS/RAB9A/RAB40B	11	5	101799698/101801928/119713234/101802364/101799739	6	101798141/101803543/119718018/101803065/101790303/101804925
MF	GO:0032550	purine ribonucleoside binding	11/543	198/6527	0.947647	0.985835	101799698/101801928/101798141/119713234/101803543/119718018/101803065/101790303/101802364/101804925/101799739	DIRAS2/ADSS1/RRAD/LOC119713234/RASEF/LOC119718018/RASL11A/RAB39A/MRAS/RAB9A/RAB40B	11	5	101799698/101801928/119713234/101802364/101799739	6	101798141/101803543/119718018/101803065/101790303/101804925

MF	GO:0032561	guanyl ribonucleotide binding	11/543	198/6527	0.947647	0.985835	101799698/101801928/101798141/119713234/101803543/119718018/101803065/101790303/101802364/101804925/101799739	DIRAS2/ADSS1/RRAD/LOC119713234/RASEF/LOC119718018/RASL11A/RAB39A/MRAS/RAB9A/RAB40B	11	5	101799698/101801928/119713234/101802364/101799739	6	101798141/101803543/119718018/101803065/101790303/101804925
MF	GO:0008047	enzyme activator activity	1/543	34/6527	0.948233	0.985835	101793795	RANGAP1	1	0		1	101793795
MF	GO:0016782	transferase activity, transferring sulfur-containing groups	1/543	34/6527	0.948233	0.985835	101793969	HS3ST3B1	1	1	101793969	0	
MF	GO:0008236	serine-type peptidase activity	3/543	73/6527	0.949307	0.985835	101804552/101791397/101797577	PLAU/DPP7/SCPEP1	3	0		3	101804552/101791397/101797577
MF	GO:0017171	serine hydrolase activity	3/543	73/6527	0.949307	0.985835	101804552/101791397/101797577	PLAU/DPP7/SCPEP1	3	0		3	101804552/101791397/101797577
MF	GO:0001882	nucleoside binding	11/543	199/6527	0.949768	0.985835	101799698/101801928/101798141/119713234/101803543/119718018/101803065/101790303/101802364/101804925/101799739	DIRAS2/ADSS1/RRAD/LOC119713234/RASEF/LOC119718018/RASL11A/RAB39A/MRAS/RAB9A/RAB40B	11	5	101799698/101801928/119713234/101802364/101799739	6	101798141/101803543/119718018/101803065/101790303/101804925
MF	GO:0044877	protein-containing complex binding	1/543	37/6527	0.960167	0.992441	101797439	SHROOM1	1	1	101797439	0	
MF	GO:0019001	guanyl nucleotide binding	11/543	209/6527	0.967168	0.995495	101799698/101801928/101798141/119713234/101803543/119718018/101803065/101790303/101802364/101804925/101799739	DIRAS2/ADSS1/RRAD/LOC119713234/RASEF/LOC119718018/RASL11A/RAB39A/MRAS/RAB9A/RAB40B	11	5	101799698/101801928/119713234/101802364/101799739	6	101798141/101803543/119718018/101803065/101790303/101804925
MF	GO:0046983	protein dimerization activity	7/543	161/6527	0.984112	0.99779	101801755/101799116/101789522/101804490/101805368/101801576/101790556	MYF5/MEF2D/MYOD1/GRPEL2/BTBD11/E2F1/TFAP4	7	4	101799116/101804490/101805368/101801576	3	101801755/101789522/101790556
MF	GO:0005198	structural molecule activity	5/543	133/6527	0.988909	0.99779	101799662/101802140/101800228/101793387/106017870	COL4A5/LOC101802140/COL4A6/COL5A1/MRPL2	5	5	101799662/101802140/101800228/101793387/106017870	0	
MF	GO:0004252	serine-type endopeptidase activity	1/543	60/6527	0.99468	0.99779	101804552	PLAU	1	0		1	101804552
MF	GO:0046982	protein heterodimerization activity	1/543	62/6527	0.995536	0.99779	101805368	BTBD11	1	1	101805368	0	
MF	GO:0003735	structural constituent of ribosome	2/543	88/6527	0.995866	0.99779	101802140/106017870	LOC101802140/MRPL2	2	2	101802140/106017870	0	
MF	GO:0003723	RNA binding	5/543	153/6527	0.996819	0.99779	119717614/101793553/113841084/101792961/101801784	LOC119717614/EIF1/KHDC4/TRNT1/AKAP1	5	3	119717614/101792961/101801784	2	101793553/113841084
MF	GO:0043565	sequence-specific DNA binding	2/543	96/6527	0.99779	0.99779	101789572/119714007	PPARA/LOC119714007	2	2	101789572/119714007	0	
