# Supplementary material for: Identification of relevant differential genes to the divergent development of pectoral muscle in ducks by transcriptomic analysis
Source: Anim Biosci. 2024 Apr 1;37(8):1345–54. doi: 10.5713/ab.23.0505 (PMC11222850; doi:10.5713/ab.23.0505)
Supplement: Supplementary file 2 [file ab-23-0505-Supplementary-Table-2.pdf]

Supplemental Table 2：Upregulation of gene-related gene ontology (GO) pathways

Category	GOID	Description	GeneRatio	BgRatio	pvalue	padj	geneID	geneName	Count
BP	GO:0017144	drug metabolic process	8/177	39/3792	0.000341	0.05167	101799171/101789492/101790726/119714948/110351624/5405820/101797601/101790402	MAT1A/FH/PKM/LOC119714948/LOC110351624/ND5/GPI/ATP5PO	8
BP	GO:0006164	purine nucleotide biosynthetic process	7/177	35/3792	0.000949	0.05167	101801928/101803512/101790726/101796516/119714948/101797601/101790402	ADSS1/ADCY2/PKM/LOC101796516/LOC119714948/GPI/ATP5PO	7
BP	GO:0055114	oxidation-reduction process	25/177	281/3792	0.001093	0.05167	101803853/101790348/101801152/101799809/101803074/101789838/101789492/101794957/101802594/101797675/101799210/101801759/101805119/119714639/101798614/101804343/101793939/119717912/101804610/101792912/101804444/101791081/5405820/101804478/101801058	ME1/PTGR2/LDHB/HADHA/ALDH1L2/ETFDH/FH/EGLN1/DMGDH/D2HGDH/LOC101799210/ALDH1A2/NDUFS1/ACADM/BCO1/ACOX2/ALDH5A1/LOC119717912/MDH2/LOC101792912/L2HGDH/LOC101791081/ND5/PRDX6/LOC101801058	25
BP	GO:0006163	purine nucleotide metabolic process	8/177	46/3792	0.001093	0.05167	101801928/101803512/101790726/101796516/119714948/5405820/101797601/101790402	ADSS1/ADCY2/PKM/LOC101796516/LOC119714948/ND5/GPI/ATP5PO	8
BP	GO:0072522	purine-containing compound biosynthetic process	7/177	36/3792	0.001132	0.05167	101801928/101803512/101790726/101796516/119714948/101797601/101790402	ADSS1/ADCY2/PKM/LOC101796516/LOC119714948/GPI/ATP5PO	7
BP	GO:0072521	purine-containing compound metabolic process	8/177	47/3792	0.001265	0.05167	101801928/101803512/101790726/101796516/119714948/5405820/101797601/101790402	ADSS1/ADCY2/PKM/LOC101796516/LOC119714948/ND5/GPI/ATP5PO	8
BP	GO:0009152	purine ribonucleotide biosynthetic process	6/177	33/3792	0.00367	0.084291	101803512/101790726/101796516/119714948/101797601/101790402	ADCY2/PKM/LOC101796516/LOC119714948/GPI/ATP5PO	6
BP	GO:0009260	ribonucleotide biosynthetic process	6/177	33/3792	0.00367	0.084291	101803512/101790726/101796516/119714948/101797601/101790402	ADCY2/PKM/LOC101796516/LOC119714948/GPI/ATP5PO	6

BP	GO:0046390	ribose phosphate biosynthetic process	6/177	33/3792	0.00367	0.084291	101803512/101790726/101796516/119714948/101797601/101790402	ADCY2/PKM/LOC101796516/LOC119714948/GPI/ATP5PO	6
BP	GO:0009150	purine ribonucleotide metabolic process	7/177	44/3792	0.003792	0.084291	101803512/101790726/101796516/119714948/5405820/101797601/101790402	ADCY2/PKM/LOC101796516/LOC119714948/ND5/GPI/ATP5PO	7
BP	GO:0009259	ribonucleotide metabolic process	7/177	44/3792	0.003792	0.084291	101803512/101790726/101796516/119714948/5405820/101797601/101790402	ADCY2/PKM/LOC101796516/LOC119714948/ND5/GPI/ATP5PO	7
BP	GO:0019693	ribose phosphate metabolic process	7/177	46/3792	0.004896	0.084291	101803512/101790726/101796516/119714948/5405820/101797601/101790402	ADCY2/PKM/LOC101796516/LOC119714948/ND5/GPI/ATP5PO	7
BP	GO:0005975	carbohydrate metabolic process	10/177	85/3792	0.005669	0.084291	101801152/101799210/101798581/101790428/113845291/101790726/101804610/113840111/101795379/101797601	LDHB/LOC101799210/PGM2L1/PGM1/LOC113845291/PKM/MDH2/LOC113840111/PGM5/GPI	10
BP	GO:0006091	generation of precursor metabolites and energy	4/177	17/3792	0.006769	0.084291	101789492/101790726/5405820/101797601	FH/PKM/ND5/GPI	4
BP	GO:0032787	monocarboxylic acid metabolic process	4/177	17/3792	0.006769	0.084291	101799809/101804343/101790726/101797601	HADHA/ACOX2/PKM/GPI	4
BP	GO:0046034	ATP metabolic process	5/177	27/3792	0.007314	0.084291	101790726/119714948/5405820/101797601/101790402	PKM/LOC119714948/ND5/GPI/ATP5PO	5
BP	GO:0006754	ATP biosynthetic process	4/177	18/3792	0.008391	0.084291	101790726/119714948/101797601/101790402	PKM/LOC119714948/GPI/ATP5PO	4
BP	GO:0009142	nucleoside triphosphate biosynthetic process	4/177	18/3792	0.008391	0.084291	101790726/119714948/101797601/101790402	PKM/LOC119714948/GPI/ATP5PO	4
BP	GO:0009145	purine nucleoside triphosphate biosynthetic process	4/177	18/3792	0.008391	0.084291	101790726/119714948/101797601/101790402	PKM/LOC119714948/GPI/ATP5PO	4
BP	GO:0009201	ribonucleoside triphosphate biosynthetic process	4/177	18/3792	0.008391	0.084291	101790726/119714948/101797601/101790402	PKM/LOC119714948/GPI/ATP5PO	4
BP	GO:0009206	purine ribonucleoside triphosphate biosynthetic process	4/177	18/3792	0.008391	0.084291	101790726/119714948/101797601/101790402	PKM/LOC119714948/GPI/ATP5PO	4
BP	GO:0009144	purine nucleoside triphosphate metabolic process	5/177	28/3792	0.008574	0.084291	101790726/119714948/5405820/101797601/101790402	PKM/LOC119714948/ND5/GPI/ATP5PO	5

BP	GO:0009199	ribonucleoside triphosphate metabolic process	5/177	28/3792	0.008574	0.084291	101790726/119714948/5405820/101797601/101790402	PKM/LOC119714948/ND5/GPI/ATP5PO	5
BP	GO:0009205	purine ribonucleoside triphosphate metabolic process	5/177	28/3792	0.008574	0.084291	101790726/119714948/5405820/101797601/101790402	PKM/LOC119714948/ND5/GPI/ATP5PO	5
BP	GO:0009123	nucleoside monophosphate metabolic process	5/177	29/3792	0.009977	0.084291	101790726/119714948/5405820/101797601/101790402	PKM/LOC119714948/ND5/GPI/ATP5PO	5
BP	GO:0009126	purine nucleoside monophosphate metabolic process	5/177	29/3792	0.009977	0.084291	101790726/119714948/5405820/101797601/101790402	PKM/LOC119714948/ND5/GPI/ATP5PO	5
BP	GO:0009141	nucleoside triphosphate metabolic process	5/177	29/3792	0.009977	0.084291	101790726/119714948/5405820/101797601/101790402	PKM/LOC119714948/ND5/GPI/ATP5PO	5
BP	GO:0009161	ribonucleoside monophosphate metabolic process	5/177	29/3792	0.009977	0.084291	101790726/119714948/5405820/101797601/101790402	PKM/LOC119714948/ND5/GPI/ATP5PO	5
BP	GO:0009167	purine ribonucleoside monophosphate metabolic process	5/177	29/3792	0.009977	0.084291	101790726/119714948/5405820/101797601/101790402	PKM/LOC119714948/ND5/GPI/ATP5PO	5
BP	GO:0009165	nucleotide biosynthetic process	7/177	53/3792	0.010718	0.08471	101801928/101803512/101790726/101796516/119714948/101797601/101790402	ADSS1/ADCY2/PKM/LOC101796516/LOC119714948/GPI/ATP5PO	7
BP	GO:1901293	nucleoside phosphate biosynthetic process	7/177	53/3792	0.010718	0.08471	101801928/101803512/101790726/101796516/119714948/101797601/101790402	ADSS1/ADCY2/PKM/LOC101796516/LOC119714948/GPI/ATP5PO	7
BP	GO:0009124	nucleoside monophosphate biosynthetic process	4/177	20/3792	0.012353	0.086471	101790726/119714948/101797601/101790402	PKM/LOC119714948/GPI/ATP5PO	4
BP	GO:0009127	purine nucleoside monophosphate biosynthetic process	4/177	20/3792	0.012353	0.086471	101790726/119714948/101797601/101790402	PKM/LOC119714948/GPI/ATP5PO	4
BP	GO:0009156	ribonucleoside monophosphate biosynthetic process	4/177	20/3792	0.012353	0.086471	101790726/119714948/101797601/101790402	PKM/LOC119714948/GPI/ATP5PO	4
BP	GO:0009168	purine ribonucleoside monophosphate biosynthetic process	4/177	20/3792	0.012353	0.086471	101790726/119714948/101797601/101790402	PKM/LOC119714948/GPI/ATP5PO	4
BP	GO:0009117	nucleotide metabolic process	8/177	71/3792	0.016517	0.112409	101801928/101803512/101790726/101796516/119714948/5405820/101797601/101790402	ADSS1/ADCY2/PKM/LOC101796516/LOC119714948/ND5/GPI/ATP5PO	8

BP	GO:0006753	nucleoside phosphate metabolic process	8/177	72/3792	0.017864	0.118288	101801928/101803512/101790726/101796516/119714948/5405820/101797601/101790402	ADSS1/ADCY2/PKM/LOC101796516/LOC119714948/ND5/GPI/ATP5PO	8
BP	GO:0015672	monovalent inorganic cation transport	7/177	59/3792	0.018774	0.121044	119714187/119714808/119717511/119714948/101799054/101797930/101790402	KCNN1/ATP1B2/ASIC4/LOC119714948/SCN5A/TMEM38A/ATP5PO	7
BP	GO:0044281	small molecule metabolic process	15/177	181/3792	0.020386	0.128066	101798008/119714249/101799809/101789492/101801928/101803512/101804343/101790726/101796516/119714948/101795091/110351624/5405820/101797601/101790402	GADL1/LOC119714249/HADHA/FH/ADSS1/ADCY2/ACOX2/PKM/LOC101796516/LOC119714948/TARS3/LOC110351624/ND5/GPI/ATP5PO	15
BP	GO:1901137	carbohydrate derivative biosynthetic process	9/177	89/3792	0.021778	0.133388	101803512/101790726/101796516/101793440/119714948/101797601/101792011/101800484/101790402	ADCY2/PKM/LOC101796516/ST6GALNAC6/LOC119714948/GPI/UGGT2/ST8SIA6/ATP5PO	9
BP	GO:0019752	carboxylic acid metabolic process	8/177	82/3792	0.035923	0.209552	101798008/119714249/101799809/101789492/101804343/101790726/101795091/101797601	GADL1/LOC119714249/HADHA/FH/ACOX2/PKM/TARS3/GPI	8
BP	GO:0055086	nucleobase-containing small molecule metabolic process	8/177	82/3792	0.035923	0.209552	101801928/101803512/101790726/101796516/119714948/5405820/101797601/101790402	ADSS1/ADCY2/PKM/LOC101796516/LOC119714948/ND5/GPI/ATP5PO	8
BP	GO:0006082	organic acid metabolic process	8/177	83/3792	0.03823	0.212872	101798008/119714249/101799809/101789492/101804343/101790726/101795091/101797601	GADL1/LOC119714249/HADHA/FH/ACOX2/PKM/TARS3/GPI	8
BP	GO:0043436	oxoacid metabolic process	8/177	83/3792	0.03823	0.212872	101798008/119714249/101799809/101789492/101804343/101790726/101795091/101797601	GADL1/LOC119714249/HADHA/FH/ACOX2/PKM/TARS3/GPI	8
BP	GO:0019637	organophosphate metabolic process	11/177	131/3792	0.040574	0.214176	101795498/101801928/101803512/101790726/101805442/101796516/119714948/101804047/5405820/101797601/101790402	PIP4K2A/ADSS1/ADCY2/PKM/IMPA2/LOC101796516/LOC119714948/PTDSS1/ND5/GPI/ATP5PO	11

BP	GO:0090407	organophosphate biosynthetic process	8/177	84/3792	0.040635	0.214176	101801928/101803512/101790726/101796516/119714948/101804047/101797601/101790402	ADSS1/ADCY2/PKM/LOC101796516/LOC119714948/PTDSS1/GPI/ATP5PO	8
BP	GO:0035556	intracellular signal transduction	14/177	181/3792	0.041235	0.214176	119717440/101796231/101803647/101790966/101795576/101803512/101792952/101796516/101802044/101801143/101790388/101795945/101799739/101792679	LOC119717440/TNS3/PLCL2/PLCB1/DCX/ADCY2/ARHGEF3/LOC101796516/GPR155/DGKI/ARHGEF17/ASB4/RAB40B/PLEKHG1	14
BP	GO:0006814	sodium ion transport	3/177	17/3792	0.041961	0.214176	119714808/119717511/101799054	ATP1B2/ASIC4/SCN5A	3
BP	GO:0006629	lipid metabolic process	10/177	120/3792	0.05183	0.259149	101795498/101799809/119713935/101803647/101790966/101804343/101805442/101804047/101799397/101790208	PIP4K2A/HADHA/PNPLA1/PLCL2/PLCB1/ACOX2/IMPA2/PTDSS1/SRD5A2/SCD5	10
BP	GO:1901135	carbohydrate derivative metabolic process	10/177	122/3792	0.056851	0.278569	101803512/101790726/101796516/101793440/119714948/5405820/101797601/101792011/101800484/101790402	ADCY2/PKM/LOC101796516/ST6GALNAC6/LOC119714948/ND5/GPI/UGGT2/ST8SIA6/ATP5PO	10
BP	GO:0006470	protein dephosphorylation	4/177	32/3792	0.059535	0.286001	101789439/101804352/101803878/101803609	LOC101789439/PTPRD/PTPRT/PTPRM	4
BP	GO:0016311	dephosphorylation	6/177	64/3792	0.076108	0.35222	101789439/101804352/101803683/101803878/101804166/101803609	LOC101789439/PTPRD/DUSP28/PTPRT/DUSP3/PTPRM	6
BP	GO:1901292	nucleoside phosphate catabolic process	2/177	10/3792	0.076195	0.35222	101790726/101797601	PKM/GPI	2
BP	GO:0015985	energy coupled proton transport, down electrochemical gradient	2/177	11/3792	0.090349	0.363625	119714948/101790402	LOC119714948/ATP5PO	2
BP	GO:0015986	ATP synthesis coupled proton transport	2/177	11/3792	0.090349	0.363625	119714948/101790402	LOC119714948/ATP5PO	2
BP	GO:0019359	nicotinamide nucleotide biosynthetic process	2/177	11/3792	0.090349	0.363625	101790726/101797601	PKM/GPI	2
BP	GO:0019363	pyridine nucleotide biosynthetic process	2/177	11/3792	0.090349	0.363625	101790726/101797601	PKM/GPI	2
BP	GO:0034404	nucleobase-containing small molecule biosynthetic process	2/177	11/3792	0.090349	0.363625	101790726/101797601	PKM/GPI	2
BP	GO:0072330	monocarboxylic acid biosynthetic process	2/177	11/3792	0.090349	0.363625	101790726/101797601	PKM/GPI	2

BP	GO:0072525	pyridine-containing compound biosynthetic process	2/177	11/3792	0.090349	0.363625	101790726/101797601	PKM/GPI	2
BP	GO:0006928	movement of cell or subcellular component	6/177	67/3792	0.090535	0.363625	101797173/101800985/101795653/101790443/106020577/101790717	DNAH5/LAMA4/DNAH3/KIF26A/KIF3C/DNAH9	6
BP	GO:0006650	glycerophospholipid metabolic process	3/177	24/3792	0.098698	0.383824	101795498/101805442/101804047	PIP4K2A/IMPA2/PTDSS1	3
BP	GO:0046486	glycerolipid metabolic process	3/177	24/3792	0.098698	0.383824	101795498/101805442/101804047	PIP4K2A/IMPA2/PTDSS1	3
BP	GO:0019362	pyridine nucleotide metabolic process	2/177	13/3792	0.120647	0.447856	101790726/101797601	PKM/GPI	2
BP	GO:0046496	nicotinamide nucleotide metabolic process	2/177	13/3792	0.120647	0.447856	101790726/101797601	PKM/GPI	2
BP	GO:0072524	pyridine-containing compound metabolic process	2/177	13/3792	0.120647	0.447856	101790726/101797601	PKM/GPI	2
BP	GO:0055085	transmembrane transport	18/177	290/3792	0.127174	0.458475	101798538/101802086/119715418/101801744/101795198/101805016/101796355/101790476/101802044/119714948/101801311/101801967/101804688/101799219/101799054/101802999/101790402/101800270	AQP4/AQP9/KCNH2/KCNH6/SLC16A1/SLC7A2/SLC16A3/ABCB6/GPR155/LOC119714948/LOC101801311/SLC2A12/ABCC8/SLC22A4/SCN5A/ABCC1/ATP5PO/KCNH7	18
BP	GO:0006811	ion transport	16/177	253/3792	0.129114	0.458475	106020272/119714187/101795864/119713349/119715418/101801744/119714808/119717511/119714948/101801311/101798131/101799054/101798462/101797930/101790402/101800270	CHRNG/KCNN1/KCNE4/SLC52A3/KCNH2/KCNH6/ATP1B2/ASIC4/LOC119714948/LOC101801311/PITPNC1/SCN5A/GABRG3/TMEM38A/ATP5PO/KCNH7	16
BP	GO:0009108	coenzyme biosynthetic process	3/177	27/3792	0.129121	0.458475	101799171/101790726/101797601	MAT1A/PKM/GPI	3
BP	GO:0009190	cyclic nucleotide biosynthetic process	2/177	14/3792	0.136608	0.478128	101803512/101796516	ADCY2/LOC101796516	2
BP	GO:0007018	microtubule-based movement	5/177	60/3792	0.146325	0.499803	101797173/101795653/101790443/106020577/101790717	DNAH5/DNAH3/KIF26A/KIF3C/DNAH9	5
BP	GO:0006733	oxidoreduction coenzyme metabolic process	2/177	15/3792	0.153001	0.499803	101790726/101797601	PKM/GPI	2
BP	GO:0006813	potassium ion transport	2/177	15/3792	0.153001	0.499803	119714187/119714808	KCNN1/ATP1B2	2

BP	GO:0009187	cyclic nucleotide metabolic process	2/177	15/3792	0.153001	0.499803	101803512/101796516	ADCY2/LOC101796516	2
BP	GO:0034655	nucleobase-containing compound catabolic process	2/177	15/3792	0.153001	0.499803	101790726/101797601	PKM/GPI	2
BP	GO:1901566	organonitrogen compound biosynthetic process	16/177	261/3792	0.156152	0.503384	119714249/101801928/101802140/101803512/101790726/101796516/101793440/119714948/101795091/110351624/106017870/101804047/101797601/101792011/101800484/101790402	LOC119714249/ADSS1/LOC101802140/ADCY2/PKM/LOC101796516/ST6GALNAC6/LOC119714948/TARS3/LOC110351624/MRPL2/PTDSS1/GPI/UGGT2/ST8SIA6/ATP5PO	16
BP	GO:0016053	organic acid biosynthetic process	2/177	16/3792	0.169751	0.533193	101790726/101797601	PKM/GPI	2
BP	GO:0046394	carboxylic acid biosynthetic process	2/177	16/3792	0.169751	0.533193	101790726/101797601	PKM/GPI	2
BP	GO:0006732	coenzyme metabolic process	3/177	31/3792	0.173999	0.539616	101799171/101790726/101797601	MAT1A/PKM/GPI	3
BP	GO:0015711	organic anion transport	2/177	17/3792	0.18679	0.564982	119713349/101798131	SLC52A3/PITPNC1	2
BP	GO:0061024	membrane organization	2/177	17/3792	0.18679	0.564982	101792227/101801562	CAV3/APOOL	2
BP	GO:0044270	cellular nitrogen compound catabolic process	2/177	18/3792	0.204054	0.602329	101790726/101797601	PKM/GPI	2
BP	GO:0046700	heterocycle catabolic process	2/177	18/3792	0.204054	0.602329	101790726/101797601	PKM/GPI	2
BP	GO:0044255	cellular lipid metabolic process	5/177	68/3792	0.209785	0.605627	101795498/101799809/101804343/101805442/101804047	PIP4K2A/HADHA/ACOX2/IMPA2/PTDSS1	5
BP	GO:0044283	small molecule biosynthetic process	3/177	34/3792	0.210115	0.605627	101790726/110351624/101797601	PKM/LOC110351624/GPI	3
BP	GO:0019439	aromatic compound catabolic process	2/177	19/3792	0.221486	0.619482	101790726/101797601	PKM/GPI	2
BP	GO:1901361	organic cyclic compound catabolic process	2/177	19/3792	0.221486	0.619482	101790726/101797601	PKM/GPI	2
BP	GO:0051188	cofactor biosynthetic process	3/177	35/3792	0.222508	0.619482	101799171/101790726/101797601	MAT1A/PKM/GPI	3
BP	GO:0016042	lipid catabolic process	2/177	20/3792	0.239034	0.658014	101790966/101804343	PLCB1/ACOX2	2
BP	GO:0046488	phosphatidylinositol metabolic process	2/177	21/3792	0.256648	0.698652	101795498/101805442	PIP4K2A/IMPA2	2

BP	GO:0007186	G-protein-coupled receptor signaling pathway	14/177	249/3792	0.270815	0.729117	106020638/101789607/101796159/101803645/101804355/101793595/101802295/101803152/101797638/101793610/101803029/101801352/101801143/101804306	APLNR/HTR5A/LOC101796159/ADGRL3/GPR176/LPAR3/UTS2R/GPR161/LOC101797638/GPR182/CELSR1/CNR1/DGKI/PTGFR	14
BP	GO:0046434	organophosphate catabolic process	2/177	23/3792	0.291902	0.747943	101790726/101797601	PKM/GPI	2
BP	GO:0006812	cation transport	7/177	116/3792	0.296772	0.747943	119714187/119714808/119717511/119714948/101799054/101797930/101790402	KCNN1/ATP1B2/ASIC4/LOC119714948/SCN5A/TMEM38A/ATP5PO	7
BP	GO:0006486	protein glycosylation	3/177	41/3792	0.299177	0.747943	101793440/101792011/101800484	ST6GALNAC6/UGGT2/ST8SIA6	3
BP	GO:0009100	glycoprotein metabolic process	3/177	41/3792	0.299177	0.747943	101793440/101792011/101800484	ST6GALNAC6/UGGT2/ST8SIA6	3
BP	GO:0009101	glycoprotein biosynthetic process	3/177	41/3792	0.299177	0.747943	101793440/101792011/101800484	ST6GALNAC6/UGGT2/ST8SIA6	3
BP	GO:0043413	macromolecule glycosylation	3/177	41/3792	0.299177	0.747943	101793440/101792011/101800484	ST6GALNAC6/UGGT2/ST8SIA6	3
BP	GO:0070085	glycosylation	3/177	41/3792	0.299177	0.747943	101793440/101792011/101800484	ST6GALNAC6/UGGT2/ST8SIA6	3
BP	GO:0006869	lipid transport	2/177	24/3792	0.309466	0.758193	101797331/101798131	APOA4/PITPNC1	2
BP	GO:0010876	lipid localization	2/177	24/3792	0.309466	0.758193	101797331/101798131	APOA4/PITPNC1	2
BP	GO:0030001	metal ion transport	4/177	61/3792	0.31736	0.769835	119714187/119714808/119717511/101799054	KCNN1/ATP1B2/ASIC4/SCN5A	4
BP	GO:0007017	microtubule-based process	5/177	81/3792	0.326789	0.784934	101797173/101795653/101790443/106020577/101790717	DNAH5/DNAH3/KIF26A/KIF3C/DNAH9	5
BP	GO:0009966	regulation of signal transduction	5/177	83/3792	0.345582	0.804127	101803486/101792952/106015345/101790388/101792679	LOC101803486/ARHGEF3/GPC1/ARHGEF17/PLEKHG1	5
BP	GO:0010646	regulation of cell communication	5/177	83/3792	0.345582	0.804127	101803486/101792952/106015345/101790388/101792679	LOC101803486/ARHGEF3/GPC1/ARHGEF17/PLEKHG1	5
BP	GO:0023051	regulation of signaling	5/177	83/3792	0.345582	0.804127	101803486/101792952/106015345/101790388/101792679	LOC101803486/ARHGEF3/GPC1/ARHGEF17/PLEKHG1	5
BP	GO:0007266	Rho protein signal transduction	3/177	45/3792	0.35119	0.804127	101792952/101790388/101792679	ARHGEF3/ARHGEF17/PLEKHG1	3
BP	GO:0035023	regulation of Rho protein signal transduction	3/177	45/3792	0.35119	0.804127	101792952/101790388/101792679	ARHGEF3/ARHGEF17/PLEKHG1	3
BP	GO:0051186	cofactor metabolic process	3/177	47/3792	0.377092	0.817417	101799171/101790726/101797601	MAT1A/PKM/GPI	3
BP	GO:0006605	protein targeting	1/177	10/3792	0.380349	0.817417	101793444	AKAP12	1

BP	GO:0007005	mitochondrion organization	1/177	10/3792	0.380349	0.817417	101801562	APOOL	1
BP	GO:0007059	chromosome segregation	1/177	10/3792	0.380349	0.817417	101795780	MAD1L1	1
BP	GO:0010256	endomembrane system organization	1/177	10/3792	0.380349	0.817417	101792227	CAV3	1
BP	GO:0050793	regulation of developmental process	1/177	10/3792	0.380349	0.817417	101800985	LAMA4	1
BP	GO:1903047	mitotic cell cycle process	1/177	10/3792	0.380349	0.817417	101795780	MAD1L1	1
BP	GO:0006644	phospholipid metabolic process	3/177	48/3792	0.38997	0.830806	101795498/101805442/101804047	PIP4K2A/IMPA2/PTDSS1	3
BP	GO:1902600	proton transmembrane transport	2/177	29/3792	0.395417	0.835148	119714948/101790402	LOC119714948/ATP5PO	2
BP	GO:0000278	mitotic cell cycle	1/177	11/3792	0.409349	0.847474	101795780	MAD1L1	1
BP	GO:0048285	organelle fission	1/177	11/3792	0.409349	0.847474	101795780	MAD1L1	1
BP	GO:0048583	regulation of response to stimulus	5/177	90/3792	0.41163	0.847474	101803486/101792952/106015345/101790388/101792679	LOC101803486/ARHGEF3/GPC1/ARHGEF17/PLEKHG1	5
BP	GO:0006471	protein ADP-ribosylation	1/177	12/3792	0.436999	0.877581	101800656	LOC101800656	1
BP	GO:0045786	negative regulation of cell cycle	1/177	12/3792	0.436999	0.877581	101795780	MAD1L1	1
BP	GO:0051239	regulation of multicellular organismal process	1/177	12/3792	0.436999	0.877581	101800985	LAMA4	1
BP	GO:0007265	Ras protein signal transduction	3/177	53/3792	0.453178	0.888229	101792952/101790388/101792679	ARHGEF3/ARHGEF17/PLEKHG1	3
BP	GO:0007275	multicellular organism development	3/177	53/3792	0.453178	0.888229	101791711/101804245/101800985	WNT4/PPDPFL/LAMA4	3
BP	GO:0046578	regulation of Ras protein signal transduction	3/177	53/3792	0.453178	0.888229	101792952/101790388/101792679	ARHGEF3/ARHGEF17/PLEKHG1	3
BP	GO:0010639	negative regulation of organelle organization	1/177	13/3792	0.463362	0.893888	101795780	MAD1L1	1
BP	GO:0030154	cell differentiation	1/177	13/3792	0.463362	0.893888	101804245	PPDPFL	1
BP	GO:0006790	sulfur compound metabolic process	1/177	14/3792	0.488497	0.909235	101799171	MAT1A	1
BP	GO:0044242	cellular lipid catabolic process	1/177	14/3792	0.488497	0.909235	101804343	ACOX2	1
BP	GO:0048869	cellular developmental process	1/177	14/3792	0.488497	0.909235	101804245	PPDPFL	1
BP	GO:0051129	negative regulation of cellular component organization	1/177	14/3792	0.488497	0.909235	101795780	MAD1L1	1
BP	GO:0006418	tRNA aminoacylation for protein translation	2/177	35/3792	0.491608	0.909235	119714249/101795091	LOC119714249/TARS3	2
BP	GO:0048856	anatomical structure development	3/177	57/3792	0.501763	0.909235	101791711/101804245/101800985	WNT4/PPDPFL/LAMA4	3
BP	GO:0045017	glycerolipid biosynthetic process	1/177	15/3792	0.512461	0.909235	101804047	PTDSS1	1

BP	GO:0046474	glycerophospholipid biosynthetic process	1/177	15/3792	0.512461	0.909235	101804047	PTDSS1	1
BP	GO:0032502	developmental process	3/177	58/3792	0.513569	0.909235	101791711/101804245/101800985	WNT4/PPDPFL/LAMA4	3
BP	GO:0002376	immune system process	2/177	37/3792	0.5215	0.909235	101795038/101800542	CX3CL1/ENPP3	2
BP	GO:0006260	DNA replication	2/177	37/3792	0.5215	0.909235	101790058/101797382	BLM/POLD3	2
BP	GO:0006955	immune response	2/177	37/3792	0.5215	0.909235	101795038/101800542	CX3CL1/ENPP3	2
BP	GO:0016567	protein ubiquitination	1/177	16/3792	0.535308	0.909235	101802075	MIB1	1
BP	GO:0042157	lipoprotein metabolic process	1/177	16/3792	0.535308	0.909235	101797331	APOA4	1
BP	GO:0043038	amino acid activation	2/177	38/3792	0.536003	0.909235	119714249/101795091	LOC119714249/TARS3	2
BP	GO:0043039	tRNA aminoacylation	2/177	38/3792	0.536003	0.909235	119714249/101795091	LOC119714249/TARS3	2
BP	GO:0051056	regulation of small GTPase mediated signal transduction	3/177	60/3792	0.536733	0.909235	101792952/101790388/101792679	ARHGEF3/ARHGEF17/PLEKHG1	3
BP	GO:0016055	Wnt signaling pathway	1/177	17/3792	0.557091	0.909235	101791711	WNT4	1
BP	GO:0032446	protein modification by small protein conjugation	1/177	17/3792	0.557091	0.909235	101802075	MIB1	1
BP	GO:0198738	cell-cell signaling by wnt	1/177	17/3792	0.557091	0.909235	101791711	WNT4	1
BP	GO:1905114	cell surface receptor signaling pathway involved in cell-cell signaling	1/177	17/3792	0.557091	0.909235	101791711	WNT4	1
BP	GO:1901575	organic substance catabolic process	4/177	84/3792	0.557314	0.909235	101790966/101804343/101790726/101797601	PLCB1/ACOX2/PKM/GPI	4
BP	GO:0098655	cation transmembrane transport	2/177	40/3792	0.564097	0.909235	119714948/101790402	LOC119714948/ATP5PO	2
BP	GO:0098660	inorganic ion transmembrane transport	2/177	40/3792	0.564097	0.909235	119714948/101790402	LOC119714948/ATP5PO	2
BP	GO:0098662	inorganic cation transmembrane transport	2/177	40/3792	0.564097	0.909235	119714948/101790402	LOC119714948/ATP5PO	2
BP	GO:1902531	regulation of intracellular signal transduction	3/177	63/3792	0.570282	0.913197	101792952/101790388/101792679	ARHGEF3/ARHGEF17/PLEKHG1	3
BP	GO:0006820	anion transport	2/177	42/3792	0.590958	0.934095	119713349/101798131	SLC52A3/PITPNC1	2
BP	GO:0034220	ion transmembrane transport	2/177	42/3792	0.590958	0.934095	119714948/101790402	LOC119714948/ATP5PO	2
BP	GO:0007267	cell-cell signaling	1/177	20/3792	0.616531	0.965582	101791711	WNT4	1
BP	GO:0009056	catabolic process	4/177	91/3792	0.621538	0.965582	101790966/101804343/101790726/101797601	PLCB1/ACOX2/PKM/GPI	4
BP	GO:0000413	protein peptidyl-prolyl isomerization	1/177	21/3792	0.634525	0.965582	101802609	PPIF	1
BP	GO:0006457	protein folding	1/177	21/3792	0.634525	0.965582	101804490	GRPEL2	1
BP	GO:0018208	peptidyl-proline modification	1/177	21/3792	0.634525	0.965582	101802609	PPIF	1
BP	GO:0022402	cell cycle process	1/177	21/3792	0.634525	0.965582	101795780	MAD1L1	1

BP	GO:0008654	phospholipid biosynthetic process	1/177	22/3792	0.65168	0.977928	101804047	PTDSS1	1
BP	GO:0051726	regulation of cell cycle	1/177	22/3792	0.65168	0.977928	101795780	MAD1L1	1
BP	GO:0016310	phosphorylation	16/177	367/3792	0.654613	0.977928	101801853/101793152/101802614/101802855/101799089/101791519/101799561/101790726/101805442/101800595/101804452/101801648/101793707/5405820/101797601/101797327	TNIK/WNK2/PRKG1/CAMK1G/MAST4/MAP3K15/PRKAA2/PKM/IMPA2/RNASEL/MUSK/CAMK2D/BMPR2/ND5/GPI/LOC101797327	16
BP	GO:0032501	multicellular organismal process	3/177	72/3792	0.661526	0.982266	101791711/101804245/101800985	WNT4/PPDPFL/LAMA4	3
BP	GO:0007166	cell surface receptor signaling pathway	2/177	51/3792	0.69657	0.998822	101791711/101799597	WNT4/FZD9	2
BP	GO:0033043	regulation of organelle organization	1/177	25/3792	0.698485	0.998822	101795780	MAD1L1	1
BP	GO:0042221	response to chemical	1/177	25/3792	0.698485	0.998822	119713349	SLC52A3	1
BP	GO:0007156	homophilic cell adhesion via plasma membrane adhesion molecules	3/177	78/3792	0.714151	0.998822	101803002/101803029/101796230	PCDH7/CELSR1/CDH8	3
BP	GO:0044248	cellular catabolic process	3/177	78/3792	0.714151	0.998822	101804343/101790726/101797601	ACOX2/PKM/GPI	3
BP	GO:0098609	cell-cell adhesion	3/177	78/3792	0.714151	0.998822	101803002/101803029/101796230	PCDH7/CELSR1/CDH8	3
BP	GO:0098742	cell-cell adhesion via plasma-membrane adhesion molecules	3/177	78/3792	0.714151	0.998822	101803002/101803029/101796230	PCDH7/CELSR1/CDH8	3
BP	GO:0007264	small GTPase mediated signal transduction	3/177	79/3792	0.722284	0.998822	101792952/101790388/101792679	ARHGEF3/ARHGEF17/PLEKHG1	3
BP	GO:0030029	actin filament-based process	1/177	27/3792	0.726157	0.998822	101799876	LOC101799876	1
BP	GO:0030036	actin cytoskeleton organization	1/177	27/3792	0.726157	0.998822	101799876	LOC101799876	1
BP	GO:0051128	regulation of cellular component organization	1/177	27/3792	0.726157	0.998822	101795780	MAD1L1	1
BP	GO:0006281	DNA repair	2/177	55/3792	0.735797	0.998822	101790058/101797870	BLM/XRCC5	2
BP	GO:0006520	cellular amino acid metabolic process	2/177	57/3792	0.753749	0.998822	119714249/101795091	LOC119714249/TARS3	2
BP	GO:0006399	tRNA metabolic process	2/177	61/3792	0.78653	0.998822	119714249/101795091	LOC119714249/TARS3	2
BP	GO:0006974	cellular response to DNA damage stimulus	2/177	62/3792	0.794106	0.998822	101790058/101797870	BLM/XRCC5	2
BP	GO:0033554	cellular response to stress	2/177	62/3792	0.794106	0.998822	101790058/101797870	BLM/XRCC5	2
BP	GO:0006397	mRNA processing	1/177	33/3792	0.794912	0.998822	101800595	RNASEL	1
BP	GO:0007049	cell cycle	1/177	35/3792	0.813773	0.998822	101795780	MAD1L1	1

BP	GO:0007155	cell adhesion	4/177	119/3792	0.815551	0.998822	101800985/101803002/101803029/101796230	LAMA4/PCDH7/CELSR1/CDH8	4
BP	GO:0022610	biological adhesion	4/177	119/3792	0.815551	0.998822	101800985/101803002/101803029/101796230	LAMA4/PCDH7/CELSR1/CDH8	4
BP	GO:0016071	mRNA metabolic process	1/177	38/3792	0.83888	0.998822	101800595	RNASEL	1
BP	GO:0071702	organic substance transport	4/177	125/3792	0.844435	0.998822	119713349/101797331/101793444/101798131	SLC52A3/APOA4/AKAP12/PITPNC1	4
BP	GO:0018193	peptidyl-amino acid modification	1/177	39/3792	0.846477	0.998822	101802609	PPIF	1
BP	GO:0048523	negative regulation of cellular process	1/177	41/3792	0.860618	0.998822	101795780	MAD1L1	1
BP	GO:0008610	lipid biosynthetic process	1/177	42/3792	0.867195	0.998822	101804047	PTDSS1	1
BP	GO:0006259	DNA metabolic process	3/177	103/3792	0.868014	0.998822	101790058/101797870/101797382	BLM/XRCC5/POLD3	3
BP	GO:0034660	ncRNA metabolic process	2/177	77/3792	0.882326	0.998822	119714249/101795091	LOC119714249/TARS3	2
BP	GO:0048519	negative regulation of biological process	1/177	46/3792	0.890557	0.998822	101795780	MAD1L1	1
BP	GO:0006412	translation	4/177	137/3792	0.890939	0.998822	119714249/101802140/101795091/106017870	LOC119714249/LOC101802140/TARS3/MRPL2	4
BP	GO:0043043	peptide biosynthetic process	4/177	140/3792	0.900487	0.998822	119714249/101802140/101795091/106017870	LOC119714249/LOC101802140/TARS3/MRPL2	4
BP	GO:0006468	protein phosphorylation	12/177	348/3792	0.901441	0.998822	101801853/101793152/101802614/101802855/101799089/101791519/101799561/101800595/101804452/101801648/101793707/101797327	TNIK/WNK2/PRKG1/CAMK1G/MAST4/MAP3K15/PRKAA2/RNASEL/MUSK/CAMK2D/BMPR2/LOC101797327	12
BP	GO:0006518	peptide metabolic process	4/177	143/3792	0.909295	0.998822	119714249/101802140/101795091/106017870	LOC119714249/LOC101802140/TARS3/MRPL2	4
BP	GO:0043604	amide biosynthetic process	4/177	143/3792	0.909295	0.998822	119714249/101802140/101795091/106017870	LOC119714249/LOC101802140/TARS3/MRPL2	4
BP	GO:0033036	macromolecule localization	3/177	119/3792	0.923161	0.998822	101797331/101793444/101798131	APOA4/AKAP12/PITPNC1	3
BP	GO:0043603	cellular amide metabolic process	4/177	149/3792	0.924872	0.998822	119714249/101802140/101795091/106017870	LOC119714249/LOC101802140/TARS3/MRPL2	4
BP	GO:0006950	response to stress	2/177	89/3792	0.926253	0.998822	101790058/101797870	BLM/XRCC5	2
BP	GO:0007010	cytoskeleton organization	1/177	55/3792	0.92924	0.998822	101799876	LOC101799876	1
BP	GO:0070647	protein modification by small protein conjugation or removal	1/177	56/3792	0.932591	0.998822	101802075	MIB1	1

BP	GO:0018130	heterocycle biosynthetic process	16/177	469/3792	0.937516	0.998822	101799370/119715483/101801928/101803512/101792149/119714375/101790726/101796516/101789572/119714948/110351624/101801576/101795523/101797601/101790402/119714007	LOC101799370/LOC119715483/ADSS1/ADCY2/POLR2B/LOC119714375/PKM/LOC101796516/PPARA/LOC119714948/LOC110351624/E2F1/MEIS1/GPI/ATP5PO/LOC119714007	16
BP	GO:0051276	chromosome organization	1/177	59/3792	0.941728	0.998822	101795780	MAD1L1	1
BP	GO:1901362	organic cyclic compound biosynthetic process	16/177	475/3792	0.944806	0.998822	101799370/119715483/101801928/101803512/101792149/119714375/101790726/101796516/101789572/119714948/110351624/101801576/101795523/101797601/101790402/119714007	LOC101799370/LOC119715483/ADSS1/ADCY2/POLR2B/LOC119714375/PKM/LOC101796516/PPARA/LOC119714948/LOC110351624/E2F1/MEIS1/GPI/ATP5PO/LOC119714007	16
BP	GO:0034654	nucleobase-containing compound biosynthetic process	15/177	452/3792	0.94697	0.998822	101799370/119715483/101801928/101803512/101792149/119714375/101790726/101796516/101789572/119714948/101801576/101795523/101797601/101790402/119714007	LOC101799370/LOC119715483/ADSS1/ADCY2/POLR2B/LOC119714375/PKM/LOC101796516/PPARA/LOC119714948/E2F1/MEIS1/GPI/ATP5PO/LOC119714007	15
BP	GO:0071705	nitrogen compound transport	2/177	102/3792	0.956242	0.998822	119713349/101793444	SLC52A3/AKAP12	2
BP	GO:0019438	aromatic compound biosynthetic process	15/177	464/3792	0.959206	0.998822	101799370/119715483/101801928/101803512/101792149/119714375/101790726/101796516/101789572/119714948/101801576/101795523/101797601/101790402/119714007	LOC101799370/LOC119715483/ADSS1/ADCY2/POLR2B/LOC119714375/PKM/LOC101796516/PPARA/LOC119714948/E2F1/MEIS1/GPI/ATP5PO/LOC119714007	15
BP	GO:0006396	RNA processing	2/177	105/3792	0.961286	0.998822	101800595/101792961	RNASEL/TRNT1	2
BP	GO:0006508	proteolysis	7/177	260/3792	0.96566	0.998822	101791511/101797901/101800115/119714444/101804308/101792943/101800508	PHEX/DPEP1/ADAMTS18/ADAMTS19/MMP23B/PIGK/LOC101800508	7

BP	GO:0006996	organelle organization	3/177	142/3792	0.966363	0.998822	101801562/101799876/101795780	APOOL/LOC101799876/MAD1L1	3
BP	GO:0006886	intracellular protein transport	1/177	76/3792	0.97453	0.998822	101793444	AKAP12	1
BP	GO:0034613	cellular protein localization	1/177	83/3792	0.981906	0.998822	101793444	AKAP12	1
BP	GO:0070727	cellular macromolecule localization	1/177	83/3792	0.981906	0.998822	101793444	AKAP12	1
BP	GO:0015031	protein transport	1/177	88/3792	0.985832	0.998822	101793444	AKAP12	1
BP	GO:0015833	peptide transport	1/177	88/3792	0.985832	0.998822	101793444	AKAP12	1
BP	GO:0042886	amide transport	1/177	89/3792	0.986509	0.998822	101793444	AKAP12	1
BP	GO:0045184	establishment of protein localization	1/177	89/3792	0.986509	0.998822	101793444	AKAP12	1
BP	GO:0046907	intracellular transport	1/177	90/3792	0.987154	0.998822	101793444	AKAP12	1
BP	GO:0051649	establishment of localization in cell	1/177	91/3792	0.987768	0.998822	101793444	AKAP12	1
BP	GO:0008104	protein localization	1/177	94/3792	0.989441	0.998822	101793444	AKAP12	1
BP	GO:0051641	cellular localization	1/177	109/3792	0.994948	0.998822	101793444	AKAP12	1
BP	GO:0016043	cellular component organization	4/177	230/3792	0.995696	0.998822	101792227/101801562/101799876/101795780	CAV3/APOOL/LOC101799876/MAD1L1	4
BP	GO:0022607	cellular component assembly	1/177	113/3792	0.995851	0.998822	101792227	CAV3	1
BP	GO:0006355	regulation of transcription, DNA-templated	7/177	332/3792	0.99645	0.998822	101799370/119715483/119714375/101789572/101801576/101795523/119714007	LOC101799370/LOC119715483/LOC119714375/PPARA/E2F1/MEIS1/LOC119714007	7
BP	GO:0051252	regulation of RNA metabolic process	7/177	332/3792	0.99645	0.998822	101799370/119715483/119714375/101789572/101801576/101795523/119714007	LOC101799370/LOC119715483/LOC119714375/PPARA/E2F1/MEIS1/LOC119714007	7
BP	GO:1903506	regulation of nucleic acid-templated transcription	7/177	332/3792	0.99645	0.998822	101799370/119715483/119714375/101789572/101801576/101795523/119714007	LOC101799370/LOC119715483/LOC119714375/PPARA/E2F1/MEIS1/LOC119714007	7
BP	GO:2001141	regulation of RNA biosynthetic process	7/177	332/3792	0.99645	0.998822	101799370/119715483/119714375/101789572/101801576/101795523/119714007	LOC101799370/LOC119715483/LOC119714375/PPARA/E2F1/MEIS1/LOC119714007	7

BP	GO:0019219	regulation of nucleobase-containing compound metabolic process	7/177	336/3792	0.996902	0.998822	101799370/119715483/119714375/101789572/101801576/101795523/119714007	LOC101799370/LOC119715483/LOC119714375/PPARA/E2F1/MEIS1/LOC119714007	7
BP	GO:0010556	regulation of macromolecule biosynthetic process	7/177	339/3792	0.997205	0.998822	101799370/119715483/119714375/101789572/101801576/101795523/119714007	LOC101799370/LOC119715483/LOC119714375/PPARA/E2F1/MEIS1/LOC119714007	7
BP	GO:0031326	regulation of cellular biosynthetic process	7/177	339/3792	0.997205	0.998822	101799370/119715483/119714375/101789572/101801576/101795523/119714007	LOC101799370/LOC119715483/LOC119714375/PPARA/E2F1/MEIS1/LOC119714007	7
BP	GO:2000112	regulation of cellular macromolecule biosynthetic process	7/177	339/3792	0.997205	0.998822	101799370/119715483/119714375/101789572/101801576/101795523/119714007	LOC101799370/LOC119715483/LOC119714375/PPARA/E2F1/MEIS1/LOC119714007	7
BP	GO:0009889	regulation of biosynthetic process	7/177	340/3792	0.997299	0.998822	101799370/119715483/119714375/101789572/101801576/101795523/119714007	LOC101799370/LOC119715483/LOC119714375/PPARA/E2F1/MEIS1/LOC119714007	7
BP	GO:0010468	regulation of gene expression	7/177	341/3792	0.997391	0.998822	101799370/119715483/119714375/101789572/101801576/101795523/119714007	LOC101799370/LOC119715483/LOC119714375/PPARA/E2F1/MEIS1/LOC119714007	7
BP	GO:0051171	regulation of nitrogen compound metabolic process	7/177	348/3792	0.997953	0.998822	101799370/119715483/119714375/101789572/101801576/101795523/119714007	LOC101799370/LOC119715483/LOC119714375/PPARA/E2F1/MEIS1/LOC119714007	7
BP	GO:0080090	regulation of primary metabolic process	7/177	348/3792	0.997953	0.998822	101799370/119715483/119714375/101789572/101801576/101795523/119714007	LOC101799370/LOC119715483/LOC119714375/PPARA/E2F1/MEIS1/LOC119714007	7
BP	GO:0031323	regulation of cellular metabolic process	7/177	353/3792	0.998282	0.998822	101799370/119715483/119714375/101789572/101801576/101795523/119714007	LOC101799370/LOC119715483/LOC119714375/PPARA/E2F1/MEIS1/LOC119714007	7

BP	GO:0060255	regulation of macromolecule metabolic process	7/177	353/3792	0.998282	0.998822	101799370/119715483/119714375/101789572/101801576/101795523/119714007	LOC101799370/LOC119715483/LOC119714375/PPARA/E2F1/MEIS1/LOC119714007	7
BP	GO:0071840	cellular component organization or biogenesis	4/177	254/3792	0.998309	0.998822	101792227/101801562/101799876/101795780	CAV3/APOOL/LOC101799876/MAD1L1	4
BP	GO:0019222	regulation of metabolic process	7/177	360/3792	0.998658	0.998822	101799370/119715483/119714375/101789572/101801576/101795523/119714007	LOC101799370/LOC119715483/LOC119714375/PPARA/E2F1/MEIS1/LOC119714007	7
BP	GO:0044085	cellular component biogenesis	1/177	137/3792	0.998734	0.998822	101792227	CAV3	1
BP	GO:0006351	transcription, DNA-templated	8/177	393/3792	0.998737	0.998822	101799370/119715483/101792149/119714375/101789572/101801576/101795523/119714007	LOC101799370/LOC119715483/POLR2B/LOC119714375/PPARA/E2F1/MEIS1/LOC119714007	8
BP	GO:0097659	nucleic acid-templated transcription	8/177	393/3792	0.998737	0.998822	101799370/119715483/101792149/119714375/101789572/101801576/101795523/119714007	LOC101799370/LOC119715483/POLR2B/LOC119714375/PPARA/E2F1/MEIS1/LOC119714007	8
BP	GO:0032774	RNA biosynthetic process	8/177	395/3792	0.998822	0.998822	101799370/119715483/101792149/119714375/101789572/101801576/101795523/119714007	LOC101799370/LOC119715483/POLR2B/LOC119714375/PPARA/E2F1/MEIS1/LOC119714007	8
CC	GO:0031226	intrinsic component of plasma membrane	5/103	17/2288	0.000674	0.043119	119713349/119714808/101803486/106015345/101799054	SLC52A3/ATP1B2/LOC101803486/GPC1/SCN5A	5
CC	GO:0005886	plasma membrane	7/103	40/2288	0.001669	0.053398	119713349/119714808/101803486/101803512/106015345/101799054/101798389	SLC52A3/ATP1B2/LOC101803486/ADCY2/GPC1/SCN5A/SGCG	7
CC	GO:0044459	plasma membrane part	6/103	34/2288	0.00346	0.073803	119713349/119714808/101803486/106015345/101799054/101798389	SLC52A3/ATP1B2/LOC101803486/GPC1/SCN5A/SGCG	6
CC	GO:0071944	cell periphery	7/103	50/2288	0.006171	0.098744	119713349/119714808/101803486/101803512/106015345/101799054/101798389	SLC52A3/ATP1B2/LOC101803486/ADCY2/GPC1/SCN5A/SGCG	7

CC	GO:0005856	cytoskeleton	11/103	119/2288	0.016009	0.194412	101804531/101797173/101795653/110353953/101796751/101803548/101790717/101802165/113841953/101790909/101798589	MYO16/DNAH5/DNAH3/LOC110353953/DST/TNNI1/DNAH9/LOC101802165/LOC113841953/LOC101790909/MYO5C	11
CC	GO:0005887	integral component of plasma membrane	3/103	13/2288	0.018226	0.194412	119713349/119714808/101799054	SLC52A3/ATP1B2/SCN5A	3
CC	GO:0044430	cytoskeletal part	9/103	100/2288	0.033249	0.303991	101804531/101797173/101795653/110353953/101803548/101790717/101802165/101790909/101798589	MYO16/DNAH5/DNAH3/LOC110353953/TNNI1/DNAH9/LOC101802165/LOC101790909/MYO5C	9
CC	GO:0030286	dynein complex	3/103	17/2288	0.038039	0.304315	101797173/101795653/101790717	DNAH5/DNAH3/DNAH9	3
CC	GO:0005875	microtubule associated complex	3/103	18/2288	0.044192	0.314255	101797173/101795653/101790717	DNAH5/DNAH3/DNAH9	3
CC	GO:0015629	actin cytoskeleton	6/103	62/2288	0.057351	0.367046	101804531/110353953/101803548/101802165/101790909/101798589	MYO16/LOC110353953/TNNI1/LOC101802165/LOC101790909/MYO5C	6
CC	GO:0016459	myosin complex	5/103	50/2288	0.071373	0.415259	101804531/110353953/101802165/101790909/101798589	MYO16/LOC110353953/LOC101802165/LOC101790909/MYO5C	5
CC	GO:0031300	intrinsic component of organelle membrane	2/103	11/2288	0.084703	0.416998	101801562/101789523	APOOL/CHCHD3	2
CC	GO:0031301	integral component of organelle membrane	2/103	11/2288	0.084703	0.416998	101801562/101789523	APOOL/CHCHD3	2
CC	GO:0015630	microtubule cytoskeleton	3/103	28/2288	0.128862	0.542638	101797173/101795653/101790717	DNAH5/DNAH3/DNAH9	3
CC	GO:0098797	plasma membrane protein complex	3/103	28/2288	0.128862	0.542638	119714808/101799054/101798389	ATP1B2/SCN5A/SGCG	3
CC	GO:1902495	transmembrane transporter complex	2/103	15/2288	0.144138	0.542638	119714808/101799054	ATP1B2/SCN5A	2
CC	GO:1990351	transporter complex	2/103	15/2288	0.144138	0.542638	119714808/101799054	ATP1B2/SCN5A	2
CC	GO:0098800	inner mitochondrial membrane protein complex	2/103	21/2288	0.243441	0.865569	101801562/101789523	APOOL/CHCHD3	2
CC	GO:0005743	mitochondrial inner membrane	2/103	27/2288	0.345085	0.907557	101801562/101789523	APOOL/CHCHD3	2
CC	GO:0019866	organelle inner membrane	2/103	27/2288	0.345085	0.907557	101801562/101789523	APOOL/CHCHD3	2
CC	GO:0044455	mitochondrial membrane part	2/103	27/2288	0.345085	0.907557	101801562/101789523	APOOL/CHCHD3	2

CC	GO:0098796	membrane protein complex	6/103	109/2288	0.366652	0.907557	119714808/101801562/119714948/101789523/101799054/101798389	ATP1B2/APOOL/LOC119714948/CHCHD3/SCN5A/SGCG	6
CC	GO:0005777	peroxisome	1/103	10/2288	0.369695	0.907557	101804343	ACOX2	1
CC	GO:0042579	microbody	1/103	10/2288	0.369695	0.907557	101804343	ACOX2	1
CC	GO:0000139	Golgi membrane	1/103	11/2288	0.398195	0.907557	101799131	CSGALNACT1	1
CC	GO:0034702	ion channel complex	1/103	11/2288	0.398195	0.907557	101799054	SCN5A	1
CC	GO:0034703	cation channel complex	1/103	11/2288	0.398195	0.907557	101799054	SCN5A	1
CC	GO:0098798	mitochondrial protein complex	2/103	31/2288	0.410609	0.907557	101801562/101789523	APOOL/CHCHD3	2
CC	GO:0005576	extracellular region	8/103	158/2288	0.418766	0.907557	101803411/101791711/101795038/101793480/101797331/101793597/101799111/101804308	IGFBP5/WNT4/CX3CL1/ADA2/APOA4/IGF1/IGF2/MMP23B	8
CC	GO:0098791	Golgi subcompartment	1/103	12/2288	0.425417	0.907557	101799131	CSGALNACT1	1
CC	GO:0031012	extracellular matrix	1/103	14/2288	0.476257	0.91444	101804308	MMP23B	1
CC	GO:0033177	proton-transporting two-sector ATPase complex, proton-transporting domain	1/103	16/2288	0.522638	0.91444	119714948	LOC119714948	1
CC	GO:0044421	extracellular region part	2/103	39/2288	0.531031	0.91444	101793480/101804308	ADA2/MMP23B	2
CC	GO:0005667	transcription factor complex	1/103	17/2288	0.544279	0.91444	101801576	E2F1	1
CC	GO:0031966	mitochondrial membrane	2/103	40/2288	0.544892	0.91444	101801562/101789523	APOOL/CHCHD3	2
CC	GO:0043228	non-membrane-bounded organelle	13/103	292/2288	0.564088	0.91444	101804531/101797173/101795653/110353953/101796751/101802140/101803548/106017870/101790717/101802165/113841953/101790909/101798589	MYO16/DNAH5/DNAH3/LOC110353953/DST/LOC101802140/TNNI1/MRPL2/DNAH9/LOC101802165/LOC113841953/LOC101790909/MYO5C	13
CC	GO:0043232	intracellular non-membrane-bounded organelle	13/103	292/2288	0.564088	0.91444	101804531/101797173/101795653/110353953/101796751/101802140/101803548/106017870/101790717/101802165/113841953/101790909/101798589	MYO16/DNAH5/DNAH3/LOC110353953/DST/LOC101802140/TNNI1/MRPL2/DNAH9/LOC101802165/LOC113841953/LOC101790909/MYO5C	13
CC	GO:0005740	mitochondrial envelope	2/103	44/2288	0.597478	0.91444	101801562/101789523	APOOL/CHCHD3	2
CC	GO:1902494	catalytic complex	4/103	95/2288	0.628806	0.91444	101797173/101795653/119714808/101790717	DNAH5/DNAH3/ATP1B2/DNAH9	4
CC	GO:0016469	proton-transporting two-sector ATPase complex	1/103	23/2288	0.655162	0.91444	119714948	LOC119714948	1

CC	GO:0099080	supramolecular complex	1/103	23/2288	0.655162	0.91444	101803548	TNNI1	1
CC	GO:0099081	supramolecular polymer	1/103	23/2288	0.655162	0.91444	101803548	TNNI1	1
CC	GO:0099512	supramolecular fiber	1/103	23/2288	0.655162	0.91444	101803548	TNNI1	1
CC	GO:0031967	organelle envelope	2/103	50/2288	0.667698	0.91444	101801562/101789523	APOOL/CHCHD3	2
CC	GO:0031975	envelope	2/103	50/2288	0.667698	0.91444	101801562/101789523	APOOL/CHCHD3	2
CC	GO:0031090	organelle membrane	3/103	76/2288	0.675145	0.91444	101801562/101799131/101789523	APOOL/CSGALNACT1/CHCHD3	3
CC	GO:0005615	extracellular space	1/103	25/2288	0.685818	0.91444	101793480	ADA2	1
CC	GO:0044429	mitochondrial part	2/103	52/2288	0.688829	0.91444	101801562/101789523	APOOL/CHCHD3	2
CC	GO:0044431	Golgi apparatus part	1/103	26/2288	0.700118	0.91444	101799131	CSGALNACT1	1
CC	GO:0005794	Golgi apparatus	1/103	28/2288	0.726812	0.930319	101799131	CSGALNACT1	1
CC	GO:0005739	mitochondrion	2/103	65/2288	0.800724	0.999697	101801562/101789523	APOOL/CHCHD3	2
CC	GO:0031984	organelle subcompartment	1/103	38/2288	0.828815	0.999697	101799131	CSGALNACT1	1
CC	GO:0098588	bounding membrane of organelle	1/103	49/2288	0.897883	0.999697	101799131	CSGALNACT1	1
CC	GO:0044446	intracellular organelle part	13/103	381/2288	0.899511	0.999697	101804531/101797173/101795653/110353953/101801562/101803548/101799131/101793627/101789523/101790717/101802165/101790909/101798589	MYO16/DNAH5/DNAH3/LOC110353953/APOOL/TNNI1/CSGALNACT1/BABAM2/CHCHD3/DNAH9/LOC101802165/LOC101790909/MYO5C	13
CC	GO:0044422	organelle part	13/103	383/2288	0.903569	0.999697	101804531/101797173/101795653/110353953/101801562/101803548/101799131/101793627/101789523/101790717/101802165/101790909/101798589	MYO16/DNAH5/DNAH3/LOC110353953/APOOL/TNNI1/CSGALNACT1/BABAM2/CHCHD3/DNAH9/LOC101802165/LOC101790909/MYO5C	13
CC	GO:0005840	ribosome	2/103	89/2288	0.918023	0.999697	101802140/106017870	LOC101802140/MRPL2	2
CC	GO:1990904	ribonucleoprotein complex	2/103	111/2288	0.965599	0.999697	101802140/106017870	LOC101802140/MRPL2	2
CC	GO:0044444	cytoplasmic part	7/103	281/2288	0.97785	0.999697	101801562/101802140/101803548/101799131/101804343/101789523/106017870	APOOL/LOC101802140/TNNI1/CSGALNACT1/ACOX2/CHCHD3/MRPL2	7
CC	GO:0005737	cytoplasm	9/103	351/2288	0.984859	0.999697	119714249/101801562/119716581/101802140/101803548/101799131/101804343/101789523/106017870	LOC119714249/APOOL/LOC119716581/LOC101802140/TNNI1/CSGALNACT1/ACOX2/CHCHD3/MRPL2	9
CC	GO:0012505	endomembrane system	1/103	90/2288	0.985453	0.999697	101799131	CSGALNACT1	1

CC	GO:0044428	nuclear part	1/103	98/2288	0.990098	0.999697	101793627	BABAM2	1
CC	GO:0005634	nucleus	6/103	334/2288	0.998724	0.999697	119714375/101789572/101793627/101790058/101797382/119714007	LOC119714375/PPARA/BABAM2/BLM/POLD3/LOC119714007	6
CC	GO:0043231	intracellular membrane-bounded organelle	10/103	483/2288	0.999477	0.999697	101801562/119714375/101799131/101804343/101789572/101793627/101790058/101789523/101797382/119714007	APOOL/LOC119714375/CSGALNACT1/ACOX2/PPARA/BABAM2/BLM/CHCHD3/POLD3/LOC119714007	10
CC	GO:0043227	membrane-bounded organelle	10/103	498/2288	0.999697	0.999697	101801562/119714375/101799131/101804343/101789572/101793627/101790058/101789523/101797382/119714007	APOOL/LOC119714375/CSGALNACT1/ACOX2/PPARA/BABAM2/BLM/CHCHD3/POLD3/LOC119714007	10
MF	GO:0016491	oxidoreductase activity	24/293	286/6527	0.00216	0.442555	101803853/101801152/101799809/101803074/101789838/101794957/101802594/101797675/101799210/101801759/101805119/119714639/101798614/101804343/101793939/119717912/101804610/101792912/101804444/101791081/5405820/101804478/101799397/101801058	ME1/LDHB/HADHA/ALDH1L2/ETFDH/EGLN1/DMGDH/D2HGDH/LOC101799210/ALDH1A2/NDUFS1/ACADM/BCO1/ACOX2/ALDH5A1/LOC119717912/MDH2/LOC101792912/L2HGDH/LOC101791081/ND5/PRDX6/SRD5A2/LOC101801058	24
MF	GO:0016887	ATPase activity	8/293	67/6527	0.009758	0.442555	101799966/101790476/101804022/101790058/101797870/101804688/101802999/101790402	ABCG8/ABCB6/VWA8/BLM/XRCC5/ABCC8/ABCC1/ATP5PO	8
MF	GO:0016616	oxidoreductase activity, acting on the CH-OH group of donors, NAD or NADP as acceptor	5/293	33/6527	0.014877	0.442555	101803853/101801152/101799809/101799210/101804610	ME1/LDHB/HADHA/LOC101799210/MDH2	5
MF	GO:0042623	ATPase activity, coupled	6/293	46/6527	0.016045	0.442555	101790476/101790058/101797870/101804688/101802999/101790402	ABCB6/BLM/XRCC5/ABCC8/ABCC1/ATP5PO	6

MF	GO:0015267	channel activity	13/293	149/6527	0.016062	0.442555	101798538/106020272/119714187/101802086/101795864/119715418/101801744/119717511/101801311/101799054/101798462/101797930/101800270	AQP4/CHRNG/KCNN1/AQP9/KCNE4/KCNH2/KCNH6/ASIC4/LOC101801311/SCN5A/GABRG3/TMEM38A/KCNH7	13
MF	GO:0022803	passive transmembrane transporter activity	13/293	149/6527	0.016062	0.442555	101798538/106020272/119714187/101802086/101795864/119715418/101801744/119717511/101801311/101799054/101798462/101797930/101800270	AQP4/CHRNG/KCNN1/AQP9/KCNE4/KCNH2/KCNH6/ASIC4/LOC101801311/SCN5A/GABRG3/TMEM38A/KCNH7	13
MF	GO:0005261	cation channel activity	5/293	35/6527	0.018924	0.442555	119714187/101795864/119717511/101799054/101797930	KCNN1/KCNE4/ASIC4/SCN5A/TMEM38A	5
MF	GO:0016614	oxidoreductase activity, acting on CH-OH group of donors	5/293	35/6527	0.018924	0.442555	101803853/101801152/101799809/101799210/101804610	ME1/LDHB/HADHA/LOC101799210/MDH2	5
MF	GO:0003774	motor activity	10/293	105/6527	0.018967	0.442555	101804531/101797173/101795653/110353953/101790443/106020577/101790717/101802165/101790909/101798589	MYO16/DNAH5/DNAH3/LOC110353953/KIF26A/KIF3C/DNAH9/LOC101802165/LOC101790909/MYO5C	10
MF	GO:0022857	transmembrane transporter activity	23/293	333/6527	0.025216	0.520652	101798538/106020272/119714187/101802086/101795864/119713349/119715418/101801744/101805016/101790476/119717511/119714948/101801311/101795056/101801967/101804688/101799219/101799054/101798462/101797930/101802999/101790402/101800270	AQP4/CHRNG/KCNN1/AQP9/KCNE4/SLC52A3/KCNH2/KCNH6/SLC7A2/ABCB6/ASIC4/LOC119714948/LOC101801311/SLC6A1/SLC2A12/ABCC8/SLC22A4/SCN5A/GABRG3/TMEM38A/ABCC1/ATP5PO/KCNH7	23
MF	GO:0005201	extracellular matrix structural constituent	3/293	15/6527	0.027272	0.520652	101799662/101800228/101793387	COL4A5/COL4A6/COL5A1	3
MF	GO:0016853	isomerase activity	6/293	55/6527	0.035591	0.538367	101798581/101790428/101795379/101790798/101797601/101802609	PGM2L1/PGM1/PGM5/TPI1/GPI/PPIF	6

MF	GO:0005215	transporter activity	24/293	364/6527	0.036394	0.538367	101798538/106020272/119714187/101802086/101795864/119713349/119715418/101801744/101805016/101790476/119717511/119714948/101801311/101795056/101801967/101804688/101798131/101799219/101799054/101798462/101797930/101802999/101790402/101800270	AQP4/CHRNG/KCNN1/AQP9/KCNE4/SLC52A3/KCNH2/KCNH6/SLC7A2/ABCB6/ASIC4/LOC119714948/LOC101801311/SLC6A1/SLC2A12/ABCC8/PITPNC1/SLC22A4/SCN5A/GABRG3/TMEM38A/ABCC1/ATP5PO/KCNH7	24
MF	GO:0005216	ion channel activity	11/293	137/6527	0.043372	0.538367	106020272/119714187/101795864/119715418/101801744/119717511/101801311/101799054/101798462/101797930/101800270	CHRNG/KCNN1/KCNE4/KCNH2/KCNH6/ASIC4/LOC101801311/SCN5A/GABRG3/TMEM38A/KCNH7	11
MF	GO:0022838	substrate-specific channel activity	11/293	137/6527	0.043372	0.538367	106020272/119714187/101795864/119715418/101801744/119717511/101801311/101799054/101798462/101797930/101800270	CHRNG/KCNN1/KCNE4/KCNH2/KCNH6/ASIC4/LOC101801311/SCN5A/GABRG3/TMEM38A/KCNH7	11
MF	GO:0016866	intramolecular transferase activity	3/293	18/6527	0.044316	0.538367	101798581/101790428/101795379	PGM2L1/PGM1/PGM5	3
MF	GO:0016829	lyase activity	5/293	44/6527	0.045771	0.538367	101798008/101789492/101803512/101803045/101796516	GADL1/FH/ADCY2/DERA/LOC101796516	5
MF	GO:0004725	protein tyrosine phosphatase activity	4/293	31/6527	0.048221	0.538367	101789439/101804352/101803878/101803609	LOC101789439/PTPRD/PTPRT/PTPRM	4
MF	GO:0015077	monovalent inorganic cation transmembrane transporter activity	7/293	75/6527	0.050273	0.538367	119714187/101795864/119717511/119714948/101795056/101799054/101790402	KCNN1/KCNE4/ASIC4/LOC119714948/SLC6A1/SCN5A/ATP5PO	7
MF	GO:0030246	carbohydrate binding	4/293	32/6527	0.05324	0.538367	101803645/113845291/101790157/101800542	ADGRL3/LOC113845291/STBD1/ENPP3	4
MF	GO:0004721	phosphoprotein phosphatase activity	6/293	63/6527	0.062212	0.538367	101789439/101804352/101803683/101803878/101804166/101803609	LOC101789439/PTPRD/DUSP28/PTPRT/DUSP3/PTPRM	6
MF	GO:0015399	primary active transmembrane transporter activity	4/293	34/6527	0.064093	0.538367	101790476/101804688/101802999/101790402	ABCB6/ABCC8/ABCC1/ATP5PO	4

MF	GO:0015405	P-P-bond-hydrolysis-driven transmembrane transporter activity	4/293	34/6527	0.064093	0.538367	101790476/101804688/101802999/101790402	ABCB6/ABCC8/ABCC1/ATP5PO	4
MF	GO:0042626	ATPase activity, coupled to transmembrane movement of substances	4/293	34/6527	0.064093	0.538367	101790476/101804688/101802999/101790402	ABCB6/ABCC8/ABCC1/ATP5PO	4
MF	GO:0043492	ATPase activity, coupled to movement of substances	4/293	34/6527	0.064093	0.538367	101790476/101804688/101802999/101790402	ABCB6/ABCC8/ABCC1/ATP5PO	4
MF	GO:0042578	phosphoric ester hydrolase activity	9/293	113/6527	0.066655	0.538367	101789439/101803647/101804352/101790966/101796581/101803683/101803878/101804166/101803609	LOC101789439/PLCL2/PTPRD/PLCB1/PDE9A/DUSP28/PTPRT/DUSP3/PTPRM	9
MF	GO:0019239	deaminase activity	2/293	10/6527	0.071226	0.553981	101794720/101793480	AMPD1/ADA2	2
MF	GO:0005272	sodium channel activity	2/293	11/6527	0.084545	0.580577	119717511/101799054	ASIC4/SCN5A	2
MF	GO:0016651	oxidoreductase activity, acting on NAD(P)H	2/293	11/6527	0.084545	0.580577	101805119/5405820	NDUFS1/ND5	2
MF	GO:0022890	inorganic cation transmembrane transporter activity	8/293	104/6527	0.094587	0.580577	119714187/101795864/119717511/119714948/101795056/101799054/101797930/101790402	KCNN1/KCNE4/ASIC4/LOC119714948/SLC6A1/SCN5A/TMEM38A/ATP5PO	8
MF	GO:0004435	phosphatidylinositol phospholipase C activity	2/293	12/6527	0.098541	0.580577	101803647/101790966	PLCL2/PLCB1	2
MF	GO:0004629	phospholipase C activity	2/293	12/6527	0.098541	0.580577	101803647/101790966	PLCL2/PLCB1	2
MF	GO:0003777	microtubule motor activity	5/293	55/6527	0.099193	0.580577	101797173/101795653/101790443/106020577/101790717	DNAH5/DNAH3/KIF26A/KIF3C/DNAH9	5
MF	GO:0015081	sodium ion transmembrane transporter activity	3/293	25/6527	0.099524	0.580577	119717511/101795056/101799054	ASIC4/SLC6A1/SCN5A	3
MF	GO:0016627	oxidoreductase activity, acting on the CH-CH group of donors	3/293	25/6527	0.099524	0.580577	119714639/101804343/101799397	ACADM/ACOX2/SRD5A2	3
MF	GO:0015318	inorganic molecular entity transmembrane transporter activity	14/293	215/6527	0.102791	0.580577	106020272/119714187/101795864/119715418/101801744/119717511/119714948/101801311/101795056/101799054/101798462/101797930/101790402/101800270	CHRNG/KCNN1/KCNE4/KCNH2/KCNH6/ASIC4/LOC119714948/LOC101801311/SLC6A1/SCN5A/GABRG3/TMEM38A/ATP5PO/KCNH7	14
MF	GO:0016791	phosphatase activity	6/293	72/6527	0.103195	0.580577	101789439/101804352/101803683/101803878/101804166/101803609	LOC101789439/PTPRD/DUSP28/PTPRT/DUSP3/PTPRM	6

MF	GO:0017111	nucleoside-triphosphatase activity	21/293	350/6527	0.105057	0.580577	101804531/101797173/101795653/110353953/101799698/101799966/101790443/101790476/101804022/106020577/101790058/101797870/101790717/101802165/101802364/101804688/101790909/101798589/101802999/101799739/101790402	MYO16/DNAH5/DNAH3/LOC110353953/DIRAS2/ABCG8/KIF26A/ABCB6/VWA8/KIF3C/BLM/XRCC5/DNAH9/LOC101802165/MRAS/ABCC8/LOC101790909/MYO5C/ABCC1/RAB40B/ATP5PO	21
MF	GO:0015075	ion transmembrane transporter activity	15/293	238/6527	0.114781	0.589264	106020272/119714187/101795864/119713349/119715418/101801744/119717511/119714948/101801311/101795056/101799054/101798462/101797930/101790402/101800270	CHRNG/KCNN1/KCNE4/SLC52A3/KCNH2/KCNH6/ASIC4/LOC119714948/LOC101801311/SLC6A1/SCN5A/GABRG3/TMEM38A/ATP5PO/KCNH7	15
MF	GO:0016462	pyrophosphatase activity	21/293	356/6527	0.119307	0.589264	101804531/101797173/101795653/110353953/101799698/101799966/101790443/101790476/101804022/106020577/101790058/101797870/101790717/101802165/101802364/101804688/101790909/101798589/101802999/101799739/101790402	MYO16/DNAH5/DNAH3/LOC110353953/DIRAS2/ABCG8/KIF26A/ABCB6/VWA8/KIF3C/BLM/XRCC5/DNAH9/LOC101802165/MRAS/ABCC8/LOC101790909/MYO5C/ABCC1/RAB40B/ATP5PO	21
MF	GO:0004222	metalloendopeptidase activity	5/293	59/6527	0.124048	0.589264	101791511/101800115/119714444/101804308/101800508	PHEX/ADAMTS18/ADAMTS19/MMP23B/LOC101800508	5
MF	GO:0016818	hydrolase activity, acting on acid anhydrides, in phosphorus-containing anhydrides	21/293	358/6527	0.124309	0.589264	101804531/101797173/101795653/110353953/101799698/101799966/101790443/101790476/101804022/106020577/101790058/101797870/101790717/101802165/101802364/101804688/101790909/101798589/101802999/101799739/101790402	MYO16/DNAH5/DNAH3/LOC110353953/DIRAS2/ABCG8/KIF26A/ABCB6/VWA8/KIF3C/BLM/XRCC5/DNAH9/LOC101802165/MRAS/ABCC8/LOC101790909/MYO5C/ABCC1/RAB40B/ATP5PO	21

MF	GO:0048037	cofactor binding	12/293	185/6527	0.127009	0.589264	101798008/101803853/113845564/101797675/101790101/101805119/119714639/101792912/101796516/101799937/101791081/101801058	GADL1/ME1/RSAD1/D2HGDH/MOCOS/NDUFS1/ACADM/LOC101792912/LOC101796516/SUCLG1/LOC101791081/LOC101801058	12
MF	GO:0008373	sialyltransferase activity	2/293	14/6527	0.128223	0.589264	101793440/101800484	ST6GALNAC6/ST8SIA6	2
MF	GO:0016849	phosphorus-oxygen lyase activity	2/293	14/6527	0.128223	0.589264	101803512/101796516	ADCY2/LOC101796516	2
MF	GO:0022804	active transmembrane transporter activity	5/293	60/6527	0.130675	0.589264	101790476/101795056/101804688/101802999/101790402	ABCB6/SLC6A1/ABCC8/ABCC1/ATP5PO	5
MF	GO:0004930	G-protein-coupled receptor activity	13/293	206/6527	0.13443	0.589264	106020638/101789607/101796159/101803645/101804355/101793595/101802295/101803152/101797638/101793610/101803029/101801352/101804306	APLNR/HTR5A/LOC101796159/ADGRL3/GPR176/LPAR3/UTS2R/GPR161/LOC101797638/GPR182/CELSR1/CNR1/PTGFR	13
MF	GO:0016817	hydrolase activity, acting on acid anhydrides	21/293	362/6527	0.134689	0.589264	101804531/101797173/101795653/110353953/101799698/101799966/101790443/101790476/101804022/106020577/101790058/101797870/101790717/101802165/101802364/101804688/101790909/101798589/101802999/101799739/101790402	MYO16/DNAH5/DNAH3/LOC110353953/DIRAS2/ABCG8/KIF26A/ABCB6/VWA8/KIF3C/BLM/XRCC5/DNAH9/LOC101802165/MRAS/ABCC8/LOC101790909/MYO5C/ABCC1/RAB40B/ATP5PO	21
MF	GO:0005267	potassium channel activity	2/293	15/6527	0.143754	0.603767	119714187/101795864	KCNN1/KCNE4	2
MF	GO:0015079	potassium ion transmembrane transporter activity	2/293	15/6527	0.143754	0.603767	119714187/101795864	KCNN1/KCNE4	2
MF	GO:0008324	cation transmembrane transporter activity	8/293	116/6527	0.149337	0.614916	119714187/101795864/119717511/119714948/101795056/101799054/101797930/101790402	KCNN1/KCNE4/ASIC4/LOC119714948/SLC6A1/SCN5A/TMEM38A/ATP5PO	8
MF	GO:0004867	serine-type endopeptidase inhibitor activity	2/293	16/6527	0.159651	0.644743	101794158/101795277	COL6A3/APLP2	2

MF	GO:0008237	metallopeptidase activity	6/293	84/6527	0.174732	0.692336	101791511/101804534/101800115/119714444/101804308/101800508	PHEX/LOC101804534/ADAMTS18/ADAMTS19/MMP23B/LOC101800508	6
MF	GO:0046873	metal ion transmembrane transporter activity	5/293	67/6527	0.181201	0.704671	119714187/101795864/119717511/101795056/101799054	KCNN1/KCNE4/ASIC4/SLC6A1/SCN5A	5
MF	GO:0016788	hydrolase activity, acting on ester bonds	12/293	201/6527	0.191616	0.729404	101802979/101789439/101803647/101804352/101790966/101796581/119716581/101803683/101803878/101800595/101804166/101803609	ARSI/LOC101789439/PLCL2/PTPRD/PLCB1/PDE9A/LOC119716581/DUSP28/PTPRT/RNASEL/DUSP3/PTPRM	12
MF	GO:0017048	Rho GTPase binding	4/293	51/6527	0.194508	0.729404	101792952/101799876/101790388/101792679	ARHGEF3/LOC101799876/ARHGEF17/PLEKHG1	4
MF	GO:0004674	protein serine/threonine kinase activity	4/293	53/6527	0.213324	0.77238	101793152/101799089/101801648/101793707	WNK2/MAST4/CAMK2D/BMPR2	4
MF	GO:0016779	nucleotidyltransferase activity	4/293	53/6527	0.213324	0.77238	101800366/101792149/101793407/101792961	TENT5B/POLR2B/TENT5C/TRNT1	4
MF	GO:0000287	magnesium ion binding	2/293	20/6527	0.225691	0.778701	101799089/101790726	MAST4/PKM	2
MF	GO:0008081	phosphoric diester hydrolase activity	3/293	37/6527	0.230094	0.778701	101803647/101790966/101796581	PLCL2/PLCB1/PDE9A	3
MF	GO:0004888	transmembrane signaling receptor activity	14/293	252/6527	0.241178	0.778701	106020638/101789607/101796159/101803645/101804355/101793595/101802295/101803152/101797638/101793610/101803029/101793707/101801352/101804306	APLNR/HTR5A/LOC101796159/ADGRL3/GPR176/LPAR3/UTS2R/GPR161/LOC101797638/GPR182/CELSR1/BMPR2/CNR1/PTGFR	14
MF	GO:0004866	endopeptidase inhibitor activity	3/293	38/6527	0.242191	0.778701	101794158/101790881/101795277	COL6A3/KNG1/APLP2	3
MF	GO:0061135	endopeptidase regulator activity	3/293	38/6527	0.242191	0.778701	101794158/101790881/101795277	COL6A3/KNG1/APLP2	3
MF	GO:0020037	heme binding	4/293	56/6527	0.242426	0.778701	101792912/101796516/101791081/101801058	LOC101792912/LOC101796516/LOC101791081/LOC101801058	4
MF	GO:0030414	peptidase inhibitor activity	3/293	39/6527	0.254389	0.778701	101794158/101790881/101795277	COL6A3/KNG1/APLP2	3
MF	GO:0061134	peptidase regulator activity	3/293	39/6527	0.254389	0.778701	101794158/101790881/101795277	COL6A3/KNG1/APLP2	3
MF	GO:0046906	tetrapyrrole binding	4/293	58/6527	0.262305	0.778701	101792912/101796516/101791081/101801058	LOC101792912/LOC101796516/LOC101791081/LOC101801058	4

MF	GO:0008194	UDP-glycosyltransferase activity	2/293	23/6527	0.276394	0.778701	101799131/101792011	CSGALNACT1/UGGT2		2
MF	GO:0017016	Ras GTPase binding	4/293	60/6527	0.28248	0.778701	101792952/101799876/101790388/101792679	ARHGEF3/LOC101799876/ARHGEF17/PLEKHG1		4
MF	GO:0031267	small GTPase binding	4/293	60/6527	0.28248	0.778701	101792952/101799876/101790388/101792679	ARHGEF3/LOC101799876/ARHGEF17/PLEKHG1		4
MF	GO:0038023	signaling receptor activity	14/293	262/6527	0.287747	0.778701	106020638/101789607/101796159/101803645/101804355/101793595/101802295/101803152/101797638/101793610/101803029/101793707/101801352/101804306	APLNR/HTR5A/LOC101796159/ADGRL3/GPR176/LPAR3/UTS2R/GPR161/LOC101797638/GPR182/CELSR1/BMPR2/CNR1/PTGFR		14
MF	GO:0060089	molecular transducer activity	14/293	262/6527	0.287747	0.778701	106020638/101789607/101796159/101803645/101804355/101793595/101802295/101803152/101797638/101793610/101803029/101793707/101801352/101804306	APLNR/HTR5A/LOC101796159/ADGRL3/GPR176/LPAR3/UTS2R/GPR161/LOC101797638/GPR182/CELSR1/BMPR2/CNR1/PTGFR		14
MF	GO:0051536	iron-sulfur cluster binding	2/293	24/6527	0.293298	0.778701	113845564/101805119	RSAD1/NDUFS1		2
MF	GO:0051540	metal cluster binding	2/293	24/6527	0.293298	0.778701	113845564/101805119	RSAD1/NDUFS1		2
MF	GO:0004871	signal transducer activity	14/293	266/6527	0.307138	0.778701	106020638/101789607/101796159/101803645/101804355/101793595/101802295/101803152/101797638/101793610/101803029/101793707/101801352/101804306	APLNR/HTR5A/LOC101796159/ADGRL3/GPR176/LPAR3/UTS2R/GPR161/LOC101797638/GPR182/CELSR1/BMPR2/CNR1/PTGFR		14
MF	GO:0030545	receptor regulator activity	6/293	103/6527	0.316196	0.778701	101795448/101795038/101800742/101793597/101799111/101803502	INHBA/CX3CL1/MSTN/IGF1/IGF2/PDGFA		6
MF	GO:0048018	receptor ligand activity	6/293	103/6527	0.316196	0.778701	101795448/101795038/101800742/101793597/101799111/101803502	INHBA/CX3CL1/MSTN/IGF1/IGF2/PDGFA		6
MF	GO:0004386	helicase activity	2/293	26/6527	0.326915	0.778701	101790058/101797870	BLM/XRCC5		2
MF	GO:0005088	Ras guanyl-nucleotide exchange factor activity	3/293	45/6527	0.32881	0.778701	101792952/101790388/101792679	ARHGEF3/ARHGEF17/PLEKHG1		3
MF	GO:0005089	Rho guanyl-nucleotide exchange factor activity	3/293	45/6527	0.32881	0.778701	101792952/101790388/101792679	ARHGEF3/ARHGEF17/PLEKHG1		3
MF	GO:0005506	iron ion binding	3/293	46/6527	0.341281	0.778701	101792912/101791081/101801058	LOC101792912/LOC101791081/LOC101801058		3

MF	GO:0030170	pyridoxal phosphate binding	2/293	27/6527	0.343571	0.778701	101798008/101790101	GADL1/MOCOS	2
MF	GO:0070279	vitamin B6 binding	2/293	27/6527	0.343571	0.778701	101798008/101790101	GADL1/MOCOS	2
MF	GO:0022836	gated channel activity	4/293	66/6527	0.344096	0.778701	106020272/119714187/101795864/101798462	CHRNG/KCNN1/KCNE4/GABRG3	4
MF	GO:0022839	ion gated channel activity	4/293	66/6527	0.344096	0.778701	106020272/119714187/101795864/101798462	CHRNG/KCNN1/KCNE4/GABRG3	4
MF	GO:0005102	signaling receptor binding	8/293	149/6527	0.353279	0.778701	101795448/101791711/101795038/101800985/101800742/101793597/101799111/101803502	INHBA/WNT4/CX3CL1/LAMA4/MSTN/IGF1/IGF2/PDGFA	8
MF	GO:0016278	lysine N-methyltransferase activity	1/293	10/6527	0.368474	0.778701	101791765	EZH1	1
MF	GO:0016279	protein-lysine N-methyltransferase activity	1/293	10/6527	0.368474	0.778701	101791765	EZH1	1
MF	GO:0018024	histone-lysine N-methyltransferase activity	1/293	10/6527	0.368474	0.778701	101791765	EZH1	1
MF	GO:0042054	histone methyltransferase activity	1/293	10/6527	0.368474	0.778701	101791765	EZH1	1
MF	GO:0004857	enzyme inhibitor activity	3/293	50/6527	0.390882	0.778701	101794158/101790881/101795277	COL6A3/KNG1/APLP2	3
MF	GO:0004620	phospholipase activity	2/293	30/6527	0.392655	0.778701	101803647/101790966	PLCL2/PLCB1	2
MF	GO:0008138	protein tyrosine/serine/threonine phosphatase activity	2/293	30/6527	0.392655	0.778701	101803683/101804166	DUSP28/DUSP3	2
MF	GO:0016298	lipase activity	2/293	30/6527	0.392655	0.778701	101803647/101790966	PLCL2/PLCB1	2
MF	GO:0005319	lipid transporter activity	1/293	11/6527	0.396867	0.778701	101798131	PITPNC1	1
MF	GO:0008009	chemokine activity	1/293	11/6527	0.396867	0.778701	101795038	CX3CL1	1
MF	GO:0008170	N-methyltransferase activity	1/293	11/6527	0.396867	0.778701	101791765	EZH1	1
MF	GO:0008276	protein methyltransferase activity	1/293	11/6527	0.396867	0.778701	101791765	EZH1	1
MF	GO:0016684	oxidoreductase activity, acting on peroxide as acceptor	1/293	11/6527	0.396867	0.778701	101804478	PRDX6	1
MF	GO:0016701	oxidoreductase activity, acting on single donors with incorporation of molecular oxygen	1/293	11/6527	0.396867	0.778701	101798614	BCO1	1
MF	GO:0016702	oxidoreductase activity, acting on single donors with incorporation of molecular oxygen, incorporation of two atoms of oxygen	1/293	11/6527	0.396867	0.778701	101798614	BCO1	1
MF	GO:0016831	carboxy-lyase activity	1/293	11/6527	0.396867	0.778701	101798008	GADL1	1

MF	GO:0022843	voltage-gated cation channel activity	1/293	11/6527	0.396867	0.778701	101795864	KCNE4	1
MF	GO:0042379	chemokine receptor binding	1/293	11/6527	0.396867	0.778701	101795038	CX3CL1	1
MF	GO:0140101	catalytic activity, acting on a tRNA	3/293	51/6527	0.403155	0.778701	119714249/119716581/101795091	LOC119714249/LOC119716581/TARS3	3
MF	GO:0005179	hormone activity	2/293	31/6527	0.408656	0.778701	101793597/101799111	IGF1/IGF2	2
MF	GO:0016705	oxidoreductase activity, acting on paired donors, with incorporation or reduction of molecular oxygen	3/293	52/6527	0.415358	0.778701	101792912/101791081/101801058	LOC101792912/LOC101791081/LOC101801058	3
MF	GO:0008484	sulfuric ester hydrolase activity	1/293	12/6527	0.423988	0.778701	101802979	ARSI	1
MF	GO:0016209	antioxidant activity	1/293	12/6527	0.423988	0.778701	101804478	PRDX6	1
MF	GO:0051213	dioxygenase activity	1/293	12/6527	0.423988	0.778701	101798614	BCO1	1
MF	GO:1901681	sulfur compound binding	1/293	12/6527	0.423988	0.778701	101795277	APLP2	1
MF	GO:0008083	growth factor activity	3/293	54/6527	0.439513	0.778701	101795448/101800742/101803502	INHBA/MSTN/PDGFA	3
MF	GO:0016757	transferase activity, transferring glycosyl groups	6/293	119/6527	0.44565	0.778701	101801173/101799131/101793440/101800656/101792011/101800484	GLT8D2/CSGALNACT1/ST6GALNAC6/LOC101800656/UGGT2/ST8SIA6	6
MF	GO:0016830	carbon-carbon lyase activity	1/293	13/6527	0.449893	0.778701	101798008	GADL1	1
MF	GO:0051015	actin filament binding	1/293	13/6527	0.449893	0.778701	101797439	SHROOM1	1
MF	GO:0019842	vitamin binding	2/293	34/6527	0.455362	0.778701	101798008/101790101	GADL1/MOCOS	2
MF	GO:0140097	catalytic activity, acting on DNA	2/293	34/6527	0.455362	0.778701	101790058/101797870	BLM/XRCC5	2
MF	GO:0140098	catalytic activity, acting on RNA	5/293	99/6527	0.459799	0.778701	119714249/119716581/101792149/101800595/101795091	LOC119714249/LOC119716581/POLR2B/RNASEL/TARS3	5
MF	GO:0098772	molecular function regulator	13/293	275/6527	0.46505	0.778701	101795448/101795038/101800742/101792952/101794158/101804490/101790881/101793597/101799111/101790388/101803502/101795277/101792679	INHBA/CX3CL1/MSTN/ARHGEF3/COL6A3/GRPEL2/KNG1/IGF1/IGF2/ARHGEF17/PDGFA/APLP2/PLEKHG1	13
MF	GO:0015078	proton transmembrane transporter activity	2/293	35/6527	0.470458	0.778701	119714948/101790402	LOC119714948/ATP5PO	2
MF	GO:0050660	flavin adenine dinucleotide binding	2/293	35/6527	0.470458	0.778701	101797675/119714639	D2HGDH/ACADM	2
MF	GO:0001664	G protein-coupled receptor binding	1/293	14/6527	0.474637	0.778701	101795038	CX3CL1	1

MF	GO:0005326	neurotransmitter transporter activity	1/293	14/6527	0.474637	0.778701	101795056	SLC6A1	1
MF	GO:0005328	neurotransmitter:sodium symporter activity	1/293	14/6527	0.474637	0.778701	101795056	SLC6A1	1
MF	GO:0005520	insulin-like growth factor binding	1/293	14/6527	0.474637	0.778701	101803411	IGFBP5	1
MF	GO:0015294	solute:cation symporter activity	1/293	14/6527	0.474637	0.778701	101795056	SLC6A1	1
MF	GO:0015370	solute:sodium symporter activity	1/293	14/6527	0.474637	0.778701	101795056	SLC6A1	1
MF	GO:0019838	growth factor binding	1/293	14/6527	0.474637	0.778701	101803411	IGFBP5	1
MF	GO:0050662	coenzyme binding	5/293	103/6527	0.494764	0.791012	101798008/101803853/101797675/101790101/119714639	GADL1/ME1/D2HGDH/MOCOS/ACADM	5
MF	GO:0005539	glycosaminoglycan binding	1/293	15/6527	0.498271	0.791012	101795277	APLP2	1
MF	GO:0019199	transmembrane receptor protein kinase activity	1/293	15/6527	0.498271	0.791012	101793707	BMPR2	1
MF	GO:0005230	extracellular ligand-gated ion channel activity	2/293	38/6527	0.514202	0.791012	106020272/101798462	CHRNG/GABRG3	2
MF	GO:0004114	3',5'-cyclic-nucleotide phosphodiesterase activity	1/293	16/6527	0.520846	0.791012	101796581	PDE9A	1
MF	GO:0008757	S-adenosylmethionine-dependent methyltransferase activity	1/293	16/6527	0.520846	0.791012	101791765	EZH1	1
MF	GO:0015293	symporter activity	1/293	16/6527	0.520846	0.791012	101795056	SLC6A1	1
MF	GO:0016879	ligase activity, forming carbon-nitrogen bonds	1/293	16/6527	0.520846	0.791012	101801928	ADSS1	1
MF	GO:0004812	aminoacyl-tRNA ligase activity	2/293	39/6527	0.528246	0.791012	119714249/101795091	LOC119714249/TARS3	2
MF	GO:0016875	ligase activity, forming carbon-oxygen bonds	2/293	39/6527	0.528246	0.791012	119714249/101795091	LOC119714249/TARS3	2
MF	GO:0008017	microtubule binding	3/293	62/6527	0.531711	0.791012	101796751/101790443/106020577	DST/KIF26A/KIF3C	3
MF	GO:0004553	hydrolase activity, hydrolyzing O-glycosyl compounds	2/293	40/6527	0.542013	0.791012	113845291/113840111	LOC113845291/LOC113840111	2
MF	GO:0015276	ligand-gated ion channel activity	2/293	40/6527	0.542013	0.791012	106020272/101798462	CHRNG/GABRG3	2
MF	GO:0022834	ligand-gated channel activity	2/293	40/6527	0.542013	0.791012	106020272/101798462	CHRNG/GABRG3	2
MF	GO:0004112	cyclic-nucleotide phosphodiesterase activity	1/293	17/6527	0.542408	0.791012	101796581	PDE9A	1
MF	GO:0052689	carboxylic ester hydrolase activity	1/293	17/6527	0.542408	0.791012	119716581	LOC119716581	1

MF	GO:0051020	GTPase binding	4/293	87/6527	0.553192	0.801175	101792952/101799876/101790388/101792679	ARHGEF3/LOC101799876/ARHGEF17/PLEKHG1	4
MF	GO:0005125	cytokine activity	1/293	19/6527	0.582675	0.827189	101795038	CX3CL1	1
MF	GO:0016741	transferase activity, transferring one-carbon groups	3/293	68/6527	0.594994	0.827189	101803074/101800220/101791765	ALDH1L2/TRMT9B/EZH1	3
MF	GO:0005244	voltage-gated ion channel activity	1/293	20/6527	0.601463	0.827189	101795864	KCNE4	1
MF	GO:0022832	voltage-gated channel activity	1/293	20/6527	0.601463	0.827189	101795864	KCNE4	1
MF	GO:0016874	ligase activity	3/293	69/6527	0.604989	0.827189	119714249/101801928/101795091	LOC119714249/ADSS1/TARS3	3
MF	GO:0003899	DNA-directed 5'-3' RNA polymerase activity	1/293	21/6527	0.619409	0.827189	101792149	POLR2B	1
MF	GO:0004540	ribonuclease activity	1/293	21/6527	0.619409	0.827189	101800595	RNASEL	1
MF	GO:0009055	electron transfer activity	1/293	21/6527	0.619409	0.827189	101805119	NDUFS1	1
MF	GO:0034062	5'-3' RNA polymerase activity	1/293	21/6527	0.619409	0.827189	101792149	POLR2B	1
MF	GO:0097747	RNA polymerase activity	1/293	21/6527	0.619409	0.827189	101792149	POLR2B	1
MF	GO:0005085	guanyl-nucleotide exchange factor activity	3/293	72/6527	0.633983	0.827189	101792952/101790388/101792679	ARHGEF3/ARHGEF17/PLEKHG1	3
MF	GO:0015631	tubulin binding	3/293	72/6527	0.633983	0.827189	101796751/101790443/106020577	DST/KIF26A/KIF3C	3
MF	GO:0003755	peptidyl-prolyl cis-trans isomerase activity	1/293	22/6527	0.636549	0.827189	101802609	PPIF	1
MF	GO:0005126	cytokine receptor binding	1/293	22/6527	0.636549	0.827189	101795038	CX3CL1	1
MF	GO:0016747	transferase activity, transferring acyl groups other than amino-acyl groups	1/293	22/6527	0.636549	0.827189	101791052	ACAA2	1
MF	GO:0016859	cis-trans isomerase activity	1/293	22/6527	0.636549	0.827189	101802609	PPIF	1
MF	GO:0016772	transferase activity, transferring phosphorus-containing groups	20/293	471/6527	0.638117	0.827189	101801853/101793152/101802614/101802855/101799089/101795498/101800366/101791519/101799561/101792149/101793407/101790726/101800595/101804452/110351624/101801648/101793707/101801143/101792961/101797327	TNIK/WNK2/PRKG1/CAMK1G/MAST4/PIP4K2A/TENT5B/MAP3K15/PRKAA2/POLR2B/TENT5C/PKM/RNASEL/MUSK/LOC110351624/CAMK2D/BMPR2/DGKI/TRNT1/LOC101797327	20
MF	GO:0051287	NAD binding	1/293	23/6527	0.65292	0.830829	101803853	ME1	1
MF	GO:0016798	hydrolase activity, acting on glycosyl bonds	2/293	49/6527	0.653133	0.830829	113845291/113840111	LOC113845291/LOC113840111	2

MF	GO:0016746	transferase activity, transferring acyl groups	2/293	50/6527	0.66406	0.830829	101803103/101791052	GPAM/ACAA2	2
MF	GO:0005044	scavenger receptor activity	1/293	24/6527	0.668555	0.830829	101800542	ENPP3	1
MF	GO:0038024	cargo receptor activity	1/293	24/6527	0.668555	0.830829	101800542	ENPP3	1
MF	GO:0019899	enzyme binding	4/293	101/6527	0.671253	0.830829	101792952/101799876/101790388/101792679	ARHGEF3/LOC101799876/ARHGEF17/PLEKHG1	4
MF	GO:0030234	enzyme regulator activity	4/293	101/6527	0.671253	0.830829	101794158/101804490/101790881/101795277	COL6A3/GRPEL2/KNG1/APLP2	4
MF	GO:0016773	phosphotransferase activity, alcohol group as acceptor	16/293	387/6527	0.672576	0.830829	101801853/101793152/101802614/101802855/101799089/101795498/101791519/101799561/101790726/101800595/101804452/110351624/101801648/101793707/101801143/101797327	TNIK/WNK2/PRKG1/CAMK1G/MAST4/PIP4K2A/MAP3K15/PRKAA2/PKM/RNASEL/MUSK/LOC110351624/CAMK2D/BMPR2/DGKI/LOC101797327	16
MF	GO:0008509	anion transmembrane transporter activity	1/293	25/6527	0.683489	0.839372	119713349	SLC52A3	1
MF	GO:0015291	secondary active transmembrane transporter activity	1/293	26/6527	0.697752	0.850884	101795056	SLC6A1	1
MF	GO:0046914	transition metal ion binding	12/293	300/6527	0.703256	0.850884	101802816/101790101/101803111/101792912/101789572/101804534/101795277/101791081/101804308/101801058/101802075/119714007	DMD/MOCOS/TRIM9/LOC101792912/PPARA/LOC101804534/APLP2/LOC101791081/MMP23B/LOC101801058/MIB1/LOC119714007	12
MF	GO:0003779	actin binding	2/293	54/6527	0.705018	0.850884	101799876/101797439	LOC101799876/SHROOM1	2
MF	GO:0016763	transferase activity, transferring pentosyl groups	1/293	27/6527	0.711374	0.853649	101800656	LOC101800656	1
MF	GO:0005198	structural molecule activity	5/293	133/6527	0.719804	0.858857	101799662/101802140/101800228/101793387/106017870	COL4A5/LOC101802140/COL4A6/COL5A1/MRPL2	5
MF	GO:0008238	exopeptidase activity	1/293	28/6527	0.724384	0.859439	101797901	DPEP1	1
MF	GO:0008092	cytoskeletal protein binding	5/293	136/6527	0.737846	0.870202	101796751/101790443/106020577/101799876/101797439	DST/KIF26A/KIF3C/LOC101799876/SHROOM1	5
MF	GO:0016758	transferase activity, transferring hexosyl groups	2/293	58/6527	0.741744	0.870202	101799131/101792011	CSGALNACT1/UGGT2	2
MF	GO:0008168	methyltransferase activity	2/293	62/6527	0.774497	0.897946	101800220/101791765	TRMT9B/EZH1	2
MF	GO:0008146	sulfotransferase activity	1/293	33/6527	0.781174	0.897946	101793969	HS3ST3B1	1

MF	GO:0016301	kinase activity	15/293	395/6527	0.787838	0.897946	101801853/101793152/101802614/101802855/101799089/101795498/101791519/101799561/101800595/101804452/110351624/101801648/101793707/101801143/101797327	TNIK/WNK2/PRKG1/CAMK1G/MAST4/PIP4K2A/MAP3K15/PRKAA2/RNASEL/MUSK/LOC110351624/CAMK2D/BMPR2/DGKI/LOC101797327	15
MF	GO:0004842	ubiquitin-protein transferase activity	1/293	34/6527	0.791047	0.897946	101802075	MIB1	1
MF	GO:0016782	transferase activity, transferring sulfur-containing groups	1/293	34/6527	0.791047	0.897946	101793969	HS3ST3B1	1
MF	GO:0019787	ubiquitin-like protein transferase activity	1/293	34/6527	0.791047	0.897946	101802075	MIB1	1
MF	GO:0004175	endopeptidase activity	5/293	148/6527	0.801526	0.904948	101791511/101800115/119714444/101804308/101800508	PHEX/ADAMTS18/ADAMTS19/MMP23B/LOC101800508	5
MF	GO:0044877	protein-containing complex binding	1/293	37/6527	0.818081	0.918701	101797439	SHROOM1	1
MF	GO:0060589	nucleoside-triphosphatase regulator activity	1/293	38/6527	0.826294	0.922988	101804490	GRPEL2	1
MF	GO:0004518	nuclease activity	1/293	39/6527	0.834138	0.92682	101800595	RNASEL	1
MF	GO:0004672	protein kinase activity	12/293	353/6527	0.877784	0.970182	101801853/101793152/101802614/101802855/101799089/101791519/101799561/101800595/101804452/101801648/101793707/101797327	TNIK/WNK2/PRKG1/CAMK1G/MAST4/MAP3K15/PRKAA2/RNASEL/MUSK/CAMK2D/BMPR2/LOC101797327	12
MF	GO:0008270	zinc ion binding	7/293	228/6527	0.8937	0.97707	101802816/101803111/101789572/101804534/101804308/101802075/119714007	DMD/TRIM9/PPARA/LOC101804534/MMP23B/MIB1/LOC119714007	7
MF	GO:0003735	structural constituent of ribosome	2/293	88/6527	0.911254	0.97707	101802140/106017870	LOC101802140/MRPL2	2
MF	GO:0008233	peptidase activity	8/293	263/6527	0.911346	0.97707	101791511/101804534/101797901/101800115/119714444/101804308/101792943/101800508	PHEX/LOC101804534/DPEP1/ADAMTS18/ADAMTS19/MMP23B/PIGK/LOC101800508	8
MF	GO:0043565	sequence-specific DNA binding	2/293	96/6527	0.934313	0.97707	101789572/119714007	PPARA/LOC119714007	2
MF	GO:0046983	protein dimerization activity	4/293	161/6527	0.936004	0.97707	101799116/101804490/101805368/101801576	MEF2D/GRPEL2/BTBD11/E2F1	4

MF	GO:0070011	peptidase activity, acting on L-amino acid peptides	7/293	252/6527	0.941135	0.97707	101791511/101804534/101797901/101800115/119714444/101804308/101800508	PHEX/LOC101804534/DPEP1/ADAMTS18/ADAMTS19/MMP23B/LOC101800508	7
MF	GO:0046982	protein heterodimerization activity	1/293	62/6527	0.942807	0.97707	101805368	BTBD11	1
MF	GO:0001883	purine nucleoside binding	5/293	198/6527	0.947654	0.97707	101799698/101801928/119713234/101802364/101799739	DIRAS2/ADSS1/LOC119713234/MRAS/RAB40B	5
MF	GO:0005525	GTP binding	5/293	198/6527	0.947654	0.97707	101799698/101801928/119713234/101802364/101799739	DIRAS2/ADSS1/LOC119713234/MRAS/RAB40B	5
MF	GO:0032549	ribonucleoside binding	5/293	198/6527	0.947654	0.97707	101799698/101801928/119713234/101802364/101799739	DIRAS2/ADSS1/LOC119713234/MRAS/RAB40B	5
MF	GO:0032550	purine ribonucleoside binding	5/293	198/6527	0.947654	0.97707	101799698/101801928/119713234/101802364/101799739	DIRAS2/ADSS1/LOC119713234/MRAS/RAB40B	5
MF	GO:0032561	guanyl ribonucleotide binding	5/293	198/6527	0.947654	0.97707	101799698/101801928/119713234/101802364/101799739	DIRAS2/ADSS1/LOC119713234/MRAS/RAB40B	5
MF	GO:0003700	DNA-binding transcription factor activity	4/293	168/6527	0.948454	0.97707	119714375/101789572/101801576/119714007	LOC119714375/PPARA/E2F1/LOC119714007	4
MF	GO:0001882	nucleoside binding	5/293	199/6527	0.949153	0.97707	101799698/101801928/119713234/101802364/101799739	DIRAS2/ADSS1/LOC119713234/MRAS/RAB40B	5
MF	GO:0005509	calcium ion binding	7/293	265/6527	0.958075	0.980826	113840667/101796751/101790966/101795002/101803002/101803029/101796230	LOC113840667/DST/PLCB1/EGF/PCDH7/CELSR1/CDH8	7
MF	GO:0019001	guanyl nucleotide binding	5/293	209/6527	0.962143	0.980826	101799698/101801928/119713234/101802364/101799739	DIRAS2/ADSS1/LOC119713234/MRAS/RAB40B	5
MF	GO:0003723	RNA binding	3/293	153/6527	0.971261	0.98084	119717614/101792961/101801784	LOC119717614/TRNT1/AKAP1	3
MF	GO:0008289	lipid binding	1/293	77/6527	0.971498	0.98084	101797331	APOA4	1
MF	GO:0003924	GTPase activity	3/293	159/6527	0.976789	0.981463	101799698/101802364/101799739	DIRAS2/MRAS/RAB40B	3
MF	GO:0140110	transcription regulator activity	4/293	205/6527	0.984585	0.984585	119714375/101789572/101801576/119714007	LOC119714375/PPARA/E2F1/LOC119714007	4
