# Supplementary material for: Identification of relevant differential genes to the divergent development of pectoral muscle in ducks by transcriptomic analysis
Source: Anim Biosci. 2024 Apr 1;37(8):1345–54. doi: 10.5713/ab.23.0505 (PMC11222850; doi:10.5713/ab.23.0505)
Supplement: Supplementary file 3 [file ab-23-0505-Supplementary-Table-3.pdf]

Supplemental Table 3 : Downregulation of gene-related gene ontology (GO) pathways

Category	GOID	Description	GeneRatio	BgRatio	pvalue	padj	geneID	geneName	Count
BP	GO:0048513	animal organ development	3/152	10/3792	0.006156	0.648516	101800783/101801755/101789522	LOC101800783/MYF5/MYOD1	3
BP	GO:0007267	cell-cell signaling	4/152	20/3792	0.007277	0.648516	101799824/101792754/101792356/101799859	WNT2/BAMBI/WNT5B/RAPSN	4
BP	GO:0044283	small molecule biosynthetic process	5/152	34/3792	0.010524	0.648516	101799450/101797455/101803472/101797078/101800658	GLUL/ASNS/ITPA/IP6K2/PCBD1	5
BP	GO:0048523	negative regulation of cellular process	5/152	41/3792	0.02266	0.648516	101798286/101792754/101791694/101797044/101791211	EIF4EBP1/BAMBI/CDKN1B/LMOD2/ZWILCH	5
BP	GO:0015711	organic anion transport	3/152	17/3792	0.028405	0.648516	101802251/101798013/101804872	PITPNM1/MPC1/LOC101804872	3
BP	GO:0016055	Wnt signaling pathway	3/152	17/3792	0.028405	0.648516	101799824/101792754/101792356	WNT2/BAMBI/WNT5B	3
BP	GO:0198738	cell-cell signaling by wnt	3/152	17/3792	0.028405	0.648516	101799824/101792754/101792356	WNT2/BAMBI/WNT5B	3
BP	GO:1905114	cell surface receptor signaling pathway involved in cell-cell signaling	3/152	17/3792	0.028405	0.648516	101799824/101792754/101792356	WNT2/BAMBI/WNT5B	3
BP	GO:0006508	proteolysis	17/152	260/3792	0.029279	0.648516	101803168/101794528/101799480/101794584/101792575/101804552/101804246/113839605/101794820/101791397/101794268/119714472/101791234/101797577/101794402/101803700/101790574	CTSL/LOC101794528/UCHL1/ADAMTS17/USP44/PLAU/CTSZ/LOC113839605/LOC101794820/DPP7/CTSB/LOC119714472/USP49/SCPEP1/TINAG/PSMB1/ADAMTS5	17
BP	GO:0048519	negative regulation of biological process	5/152	46/3792	0.035361	0.648516	101798286/101792754/101791694/101797044/101791211	EIF4EBP1/BAMBI/CDKN1B/LMOD2/ZWILCH	5
BP	GO:0006468	protein phosphorylation	21/152	348/3792	0.035507	0.648516	101793905/101803371/101797616/101803562/101803181/101791030/101804342/101800942/101802737/101793068/101795713/101790169/101792645/101797446/101795633/101801641/101791769/106017602/101791037/101796806/101789868	ULK2/MAP2K3/LOC101797616/TRIB1/MAP3K6/STYK1/SIK1/RPS6KA1/CLK1/PHKG1/KALRN/LOC101790169/PIM3/OXSR1/RAF1/LOC101801641/MAP3K8/TSSK2/MAP2K1/CDK10/PXK	21
BP	GO:0048731	system development	3/152	19/3792	0.038189	0.648516	101800783/101801755/101789522	LOC101800783/MYF5/MYOD1	3
BP	GO:0007166	cell surface receptor signaling pathway	5/152	51/3792	0.051744	0.648516	101791406/101799824/101792754/101792356/101791584	CD247/WNT2/BAMBI/WNT5B/SMAD6	5

BP	GO:0051726	regulation of cell cycle	3/152	22/3792	0.055647	0.648516	101803833/101791694/101791211	RGCC/CDKN1B/ZWILCH	3
BP	GO:0016310	phosphorylation	21/152	367/3792	0.05762	0.648516	101793905/101803371/101797616/101803562/101803181/101791030/101804342/101800942/101802737/101793068/101795713/101790169/101792645/101797446/101795633/101801641/101791769/106017602/101791037/101796806/101789868	ULK2/MAP2K3/LOC101797616/TRIB1/MAP3K6/STYK1/SIK1/RPS6KA1/CLK1/PHKG1/KALRN/LOC101790169/PIM3/OXSR1/RAF1/LOC101801641/MAP3K8/TSSK2/MAP2K1/CDK10/PXK	21
BP	GO:0006066	alcohol metabolic process	2/152	10/3792	0.058153	0.648516	101797078/101800658	IP6K2/PCBD1	2
BP	GO:1901605	alpha-amino acid metabolic process	2/152	10/3792	0.058153	0.648516	101799450/101797455	GLUL/ASNS	2
BP	GO:1901615	organic hydroxy compound metabolic process	2/152	10/3792	0.058153	0.648516	101797078/101800658	IP6K2/PCBD1	2
BP	GO:0007275	multicellular organism development	5/152	53/3792	0.059357	0.648516	101800783/101801755/101789522/101799824/101792356	LOC101800783/MYF5/MYOD1/WNT2/WNT5B	5
BP	GO:0006950	response to stress	7/152	89/3792	0.064295	0.648516	101800755/101794631/101800783/101795336/novel.277/101796914/101802532	SESN1/LOC101794631/LOC101800783/SHLD1/-/LIG1/MSH2	7
BP	GO:0006869	lipid transport	3/152	24/3792	0.069046	0.648516	101802251/101804872/101798726	PITPNM1/LOC101804872/APOLD1	3
BP	GO:0010876	lipid localization	3/152	24/3792	0.069046	0.648516	101802251/101804872/101798726	PITPNM1/LOC101804872/APOLD1	3
BP	GO:0006413	translational initiation	2/152	11/3792	0.06925	0.648516	101798286/101793553	EIF4EBP1/EIF1	2
BP	GO:0015718	monocarboxylic acid transport	2/152	11/3792	0.06925	0.648516	101798013/101804872	MPC1/LOC101804872	2
BP	GO:0015849	organic acid transport	2/152	11/3792	0.06925	0.648516	101798013/101804872	MPC1/LOC101804872	2
BP	GO:0046942	carboxylic acid transport	2/152	11/3792	0.06925	0.648516	101798013/101804872	MPC1/LOC101804872	2
BP	GO:0010941	regulation of cell death	4/152	40/3792	0.074262	0.648516	101798341/101797616/101789899/101796820	BNIP3/LOC101797616/LOC101789899/LOC101	4
BP	GO:0042981	regulation of apoptotic process	4/152	40/3792	0.074262	0.648516	101798341/101797616/101789899/101796820	BNIP3/LOC101797616/LOC101789899/LOC101	4
BP	GO:0043067	regulation of programmed cell death	4/152	40/3792	0.074262	0.648516	101798341/101797616/101789899/101796820	BNIP3/LOC101797616/LOC101789899/LOC101	4
BP	GO:0042221	response to chemical	3/152	25/3792	0.076243	0.648516	101800755/101792754/101791584	SESN1/BAMBI/SMAD6	3
BP	GO:0048856	anatomical structure development	5/152	57/3792	0.0764	0.648516	101800783/101801755/101789522/101799824/101792356	LOC101800783/MYF5/MYOD1/WNT2/WNT5B	5
BP	GO:0045786	negative regulation of cell cycle	2/152	12/3792	0.080972	0.648516	101791694/101791211	CDKN1B/ZWILCH	2
BP	GO:0032502	developmental process	5/152	58/3792	0.081035	0.648516	101800783/101801755/101789522/101799824/101792356	LOC101800783/MYF5/MYOD1/WNT2/WNT5B	5
BP	GO:0050790	regulation of catalytic activity	3/152	26/3792	0.083757	0.648516	101795631/101794268/119714472	LOC101795631/CTSB/LOC119714472	3
BP	GO:0065009	regulation of molecular function	3/152	26/3792	0.083757	0.648516	101795631/101794268/119714472	LOC101795631/CTSB/LOC119714472	3

BP	GO:0007167	enzyme linked receptor protein signaling pathway	2/152	13/3792	0.093252	0.701979	101792754/101791584	BAMBI/SMAD6	2
BP	GO:0009719	response to endogenous stimulus	2/152	14/3792	0.106027	0.751484	101792754/101791584	BAMBI/SMAD6	2
BP	GO:0071495	cellular response to endogenous stimulus	2/152	14/3792	0.106027	0.751484	101792754/101791584	BAMBI/SMAD6	2
BP	GO:0006915	apoptotic process	4/152	47/3792	0.117425	0.751484	101798341/101797616/101789899/101796820	BNIP3/LOC101797616/LOC101789899/LOC101	4
BP	GO:0008219	cell death	4/152	47/3792	0.117425	0.751484	101798341/101797616/101789899/101796820	BNIP3/LOC101797616/LOC101789899/LOC101	4
BP	GO:0012501	programmed cell death	4/152	47/3792	0.117425	0.751484	101798341/101797616/101789899/101796820	BNIP3/LOC101797616/LOC101789899/LOC101	4
BP	GO:0070887	cellular response to chemical stimulus	2/152	15/3792	0.119239	0.751484	101792754/101791584	BAMBI/SMAD6	2
BP	GO:0071310	cellular response to organic substance	2/152	15/3792	0.119239	0.751484	101792754/101791584	BAMBI/SMAD6	2
BP	GO:0016053	organic acid biosynthetic process	2/152	16/3792	0.132833	0.799948	101799450/101797455	GLUL/ASNS	2
BP	GO:0046394	carboxylic acid biosynthetic process	2/152	16/3792	0.132833	0.799948	101799450/101797455	GLUL/ASNS	2
BP	GO:0010033	response to organic substance	2/152	17/3792	0.146756	0.846748	101792754/101791584	BAMBI/SMAD6	2
BP	GO:0051171	regulation of nitrogen compound metabolic process	18/152	348/3792	0.154129	0.846748	101805187/101798286/101794377/101799581/101801567/101790639/106019185/101801755/101801698/101795336/101794314/101797962/101802147/101792591/101789522/101796626/113845655/101791584	ATF3/EIF4EBP1/MAFK/LOC101799581/TAF1D/CREM/CEBPB/MYF5/FOS/SHLD1/NFIL3/CREB5/L3MBTL3/LRRFIP1/MYOD1/JUN/JUND/SMAD6	18
BP	GO:0080090	regulation of primary metabolic process	18/152	348/3792	0.154129	0.846748	101805187/101798286/101794377/101799581/101801567/101790639/106019185/101801755/101801698/101795336/101794314/101797962/101802147/101792591/101789522/101796626/113845655/101791584	ATF3/EIF4EBP1/MAFK/LOC101799581/TAF1D/CREM/CEBPB/MYF5/FOS/SHLD1/NFIL3/CREB5/L3MBTL3/LRRFIP1/MYOD1/JUN/JUND/SMAD6	18
BP	GO:0032501	multicellular organismal process	5/152	72/3792	0.160437	0.846748	101800783/101801755/101789522/101799824/101792356	LOC101800783/MYF5/MYOD1/WNT2/WNT5B	5
BP	GO:0044270	cellular nitrogen compound catabolic process	2/152	18/3792	0.160962	0.846748	101805057/101803472	HMOX1/ITPA	2
BP	GO:0046700	heterocycle catabolic process	2/152	18/3792	0.160962	0.846748	101805057/101803472	HMOX1/ITPA	2
BP	GO:0007049	cell cycle	3/152	35/3792	0.163463	0.846748	101803833/101791694/101791211	RGCC/CDKN1B/ZWILCH	3
BP	GO:0031323	regulation of cellular metabolic process	18/152	353/3792	0.168725	0.846748	101805187/101798286/101794377/101799581/101801567/101790639/106019185/101801755/101801698/101795336/101794314/101797962/101802147/101792591/101789522/101796626/113845655/101791584	ATF3/EIF4EBP1/MAFK/LOC101799581/TAF1D/CREM/CEBPB/MYF5/FOS/SHLD1/NFIL3/CREB5/L3MBTL3/LRRFIP1/MYOD1/JUN/JUND/SMAD6	18

BP	GO:0060255	regulation of macromolecule metabolic process	18/152	353/3792	0.168725	0.846748	101805187/101798286/101794377/101799581/101801567/101790639/106019185/101801755/101801698/101795336/101794314/101797962/101802147/101792591/101789522/101796626/113845655/101791584	ATF3/EIF4EBP1/MAFK/LOC101799581/TAF1D/CREM/CEBPB/MYF5/FOS/SHLD1/NFIL3/CREB5/L3MBTL3/LRRFIP1/MYOD1/JUN/JUND/SMAD6	18
BP	GO:0019439	aromatic compound catabolic process	2/152	19/3792	0.175406	0.848841	101805057/101803472	HMOX1/ITPA	2
BP	GO:1901361	organic cyclic compound catabolic process	2/152	19/3792	0.175406	0.848841	101805057/101803472	HMOX1/ITPA	2
BP	GO:0019219	regulation of nucleobase-containing compound metabolic process	17/152	336/3792	0.186094	0.860867	101805187/101794377/101799581/101801567/101790639/106019185/101801755/101801698/101795336/101794314/101797962/101802147/101792591/101789522/101796626/113845655/101791584	ATF3/MAFK/LOC101799581/TAF1D/CREM/CEBPB/MYF5/FOS/SHLD1/NFIL3/CREB5/L3MBTL3/LRRFIP1/MYOD1/JUN/JUND/SMAD6	17
BP	GO:0019222	regulation of metabolic process	18/152	360/3792	0.190361	0.860867	101805187/101798286/101794377/101799581/101801567/101790639/106019185/101801755/101801698/101795336/101794314/101797962/101802147/101792591/101789522/101796626/113845655/101791584	ATF3/EIF4EBP1/MAFK/LOC101799581/TAF1D/CREM/CEBPB/MYF5/FOS/SHLD1/NFIL3/CREB5/L3MBTL3/LRRFIP1/MYOD1/JUN/JUND/SMAD6	18
BP	GO:0010556	regulation of macromolecule biosynthetic process	17/152	339/3792	0.195974	0.860867	101805187/101798286/101794377/101799581/101801567/101790639/106019185/101801755/101801698/101794314/101797962/101802147/101792591/101789522/101796626/113845655/101791584	ATF3/EIF4EBP1/MAFK/LOC101799581/TAF1D/CREM/CEBPB/MYF5/FOS/NFIL3/CREB5/L3MBTL3/LRRFIP1/MYOD1/JUN/JUND/SMAD6	17
BP	GO:0031326	regulation of cellular biosynthetic process	17/152	339/3792	0.195974	0.860867	101805187/101798286/101794377/101799581/101801567/101790639/106019185/101801755/101801698/101794314/101797962/101802147/101792591/101789522/101796626/113845655/101791584	ATF3/EIF4EBP1/MAFK/LOC101799581/TAF1D/CREM/CEBPB/MYF5/FOS/NFIL3/CREB5/L3MBTL3/LRRFIP1/MYOD1/JUN/JUND/SMAD6	17

BP	GO:2000112	regulation of cellular macromolecule biosynthetic process	17/152	339/3792	0.195974	0.860867	101805187/101798286/101794377/101799581/101801567/101790639/106019185/101801755/101801698/101794314/101797962/101802147/101792591/101789522/101796626/113845655/101791584	ATF3/EIF4EBP1/MAFK/LOC101799581/TAF1D/CREM/CEBPB/MYF5/FOS/NFIL3/CREB5/L3MBTL3/LRRFIP1/MYOD1/JUN/JUND/SMAD6	17
BP	GO:0009889	regulation of biosynthetic process	17/152	340/3792	0.199325	0.860867	101805187/101798286/101794377/101799581/101801567/101790639/106019185/101801755/101801698/101794314/101797962/101802147/101792591/101789522/101796626/113845655/101791584	ATF3/EIF4EBP1/MAFK/LOC101799581/TAF1D/CREM/CEBPB/MYF5/FOS/NFIL3/CREB5/L3MBTL3/LRRFIP1/MYOD1/JUN/JUND/SMAD6	17
BP	GO:0010468	regulation of gene expression	17/152	341/3792	0.202703	0.860867	101805187/101798286/101794377/101799581/101801567/101790639/106019185/101801755/101801698/101794314/101797962/101802147/101792591/101789522/101796626/113845655/101791584	ATF3/EIF4EBP1/MAFK/LOC101799581/TAF1D/CREM/CEBPB/MYF5/FOS/NFIL3/CREB5/L3MBTL3/LRRFIP1/MYOD1/JUN/JUND/SMAD6	17
BP	GO:0022402	cell cycle process	2/152	21/3792	0.204844	0.860867	101791694/101791211	CDKN1B/ZWILCH	2
BP	GO:0055114	oxidation-reduction process	14/152	281/3792	0.233366	0.860867	101790158/101793380/101794631/113839590/101798440/101805057/101789745/101793275/119713271/101789907/101792573/101801193/101791774/101797377	HIBADH/AASS/LOC101794631/LOC113839590/LOC101798440/HMOX1/ME3/ALDH7A1/LOC119713271/IVD/LOC101792573/ALDH8A1/ALDH4A1/GLUD1	14
BP	GO:0046434	organophosphate catabolic process	2/152	23/3792	0.234772	0.860867	101798440/101803472	LOC101798440/ITPA	2
BP	GO:0006820	anion transport	3/152	42/3792	0.236139	0.860867	101802251/101798013/101804872	PITPNM1/MPC1/LOC101804872	3
BP	GO:1901575	organic substance catabolic process	5/152	84/3792	0.245848	0.860867	101799480/101798440/101805057/101803472/101803700	UCHL1/LOC101798440/HMOX1/ITPA/PSMB1	5
BP	GO:0048522	positive regulation of cellular process	2/152	24/3792	0.249838	0.860867	101798341/101792754	BNIP3/BAMBI	2
BP	GO:0006355	regulation of transcription, DNA-templated	16/152	332/3792	0.253389	0.860867	101805187/101794377/101799581/101801567/101790639/106019185/101801755/101801698/101794314/101797962/101802147/101792591/101789522/101796626/113845655/101791584	ATF3/MAFK/LOC101799581/TAF1D/CREM/CEBPB/MYF5/FOS/NFIL3/CREB5/L3MBTL3/LRRFIP1/MYOD1/JUN/JUND/SMAD6	16

BP	GO:0051252	regulation of RNA metabolic process	16/152	332/3792	0.253389	0.860867	101805187/101794377/101799581/101801567/101790639/106019185/101801755/101801698/101794314/101797962/101802147/101792591/101789522/101796626/113845655/101791584	ATF3/MAFK/LOC101799581/TAF1D/CREM/CEBPB/MYF5/FOS/NFIL3/CREB5/L3MBTL3/LRRFIP1/MYOD1/JUN/JUND/SMAD6	16
BP	GO:1903506	regulation of nucleic acid-templated transcription	16/152	332/3792	0.253389	0.860867	101805187/101794377/101799581/101801567/101790639/106019185/101801755/101801698/101794314/101797962/101802147/101792591/101789522/101796626/113845655/101791584	ATF3/MAFK/LOC101799581/TAF1D/CREM/CEBPB/MYF5/FOS/NFIL3/CREB5/L3MBTL3/LRRFIP1/MYOD1/JUN/JUND/SMAD6	16
BP	GO:2001141	regulation of RNA biosynthetic process	16/152	332/3792	0.253389	0.860867	101805187/101794377/101799581/101801567/101790639/106019185/101801755/101801698/101794314/101797962/101802147/101792591/101789522/101796626/113845655/101791584	ATF3/MAFK/LOC101799581/TAF1D/CREM/CEBPB/MYF5/FOS/NFIL3/CREB5/L3MBTL3/LRRFIP1/MYOD1/JUN/JUND/SMAD6	16
BP	GO:0016311	dephosphorylation	4/152	64/3792	0.253616	0.860867	101802393/101803221/101792804/101791271	DUSP1/PTPN14/DUSP15/PTPN6	4
BP	GO:0048518	positive regulation of biological process	2/152	26/3792	0.280032	0.860867	101798341/101792754	BNIP3/BAMBI	2
BP	GO:1901565	organonitrogen compound catabolic process	3/152	47/3792	0.290994	0.860867	101799480/101805057/101803700	UCHL1/HMOX1/PSMB1	3
BP	GO:0009056	catabolic process	5/152	91/3792	0.300326	0.860867	101799480/101798440/101805057/101803472/101803700	UCHL1/LOC101798440/HMOX1/ITPA/PSMB1	5
BP	GO:0006952	defense response	1/152	10/3792	0.336081	0.860867	novel.277	-	1
BP	GO:0010558	negative regulation of macromolecule biosynthetic process	1/152	10/3792	0.336081	0.860867	101798286	EIF4EBP1	1
BP	GO:0031327	negative regulation of cellular biosynthetic process	1/152	10/3792	0.336081	0.860867	101798286	EIF4EBP1	1
BP	GO:0043244	regulation of protein complex disassembly	1/152	10/3792	0.336081	0.860867	101797044	LMOD2	1
BP	GO:1901292	nucleoside phosphate catabolic process	1/152	10/3792	0.336081	0.860867	101803472	ITPA	1
BP	GO:1903047	mitotic cell cycle process	1/152	10/3792	0.336081	0.860867	101791211	ZWILCH	1
BP	GO:2000113	negative regulation of cellular macromolecule biosynthetic process	1/152	10/3792	0.336081	0.860867	101798286	EIF4EBP1	1
BP	GO:0000278	mitotic cell cycle	1/152	11/3792	0.362764	0.860867	101791211	ZWILCH	1
BP	GO:0009890	negative regulation of biosynthetic process	1/152	11/3792	0.362764	0.860867	101798286	EIF4EBP1	1
BP	GO:0032268	regulation of cellular protein metabolic process	1/152	11/3792	0.362764	0.860867	101798286	EIF4EBP1	1
BP	GO:0033013	tetrapyrrole metabolic process	1/152	11/3792	0.362764	0.860867	101805057	HMOX1	1
BP	GO:0034404	nucleobase-containing small molecule biosynthetic process	1/152	11/3792	0.362764	0.860867	101803472	ITPA	1
BP	GO:0051172	negative regulation of nitrogen compound metabolic process	1/152	11/3792	0.362764	0.860867	101798286	EIF4EBP1	1

BP	GO:0051494	negative regulation of cytoskeleton organization	1/152	11/3792	0.362764	0.860867	101797044	LMOD2	1
BP	GO:0072330	monocarboxylic acid biosynthetic process	1/152	11/3792	0.362764	0.860867	101797455	ASNS	1
BP	GO:0006470	protein dephosphorylation	2/152	32/3792	0.369446	0.860867	101803221/101791271	PTPN14/PTPN6	2
BP	GO:0006281	DNA repair	3/152	55/3792	0.379956	0.860867	101795336/101796914/101802532	SHLD1/LIG1/MSH2	3
BP	GO:0044248	cellular catabolic process	4/152	78/3792	0.381857	0.860867	101799480/101805057/101803472/101803700	UCHL1/HMOX1/ITPA/PSMB1	4
BP	GO:0071702	organic substance transport	6/152	125/3792	0.385799	0.860867	101802251/101798013/101804872/101792755/101798726/101801245	PITPNM1/MPC1/LOC101804872/LOC101792755/APOLD1/SELENOS	6
BP	GO:0006471	protein ADP-ribosylation	1/152	12/3792	0.388382	0.860867	119716831	LOC119716831	1
BP	GO:0031324	negative regulation of cellular metabolic process	1/152	12/3792	0.388382	0.860867	101798286	EIF4EBP1	1
BP	GO:0051246	regulation of protein metabolic process	1/152	12/3792	0.388382	0.860867	101798286	EIF4EBP1	1
BP	GO:0006520	cellular amino acid metabolic process	3/152	57/3792	0.40196	0.860867	101799450/101797455/101797377	GLUL/ASNS/GLUD1	3
BP	GO:0044257	cellular protein catabolic process	2/152	35/3792	0.412612	0.860867	101799480/101803700	UCHL1/PSMB1	2
BP	GO:0051603	proteolysis involved in cellular protein catabolic process	2/152	35/3792	0.412612	0.860867	101799480/101803700	UCHL1/PSMB1	2
BP	GO:0006334	nucleosome assembly	1/152	13/3792	0.412976	0.860867	101798499	LOC101798499	1
BP	GO:0010629	negative regulation of gene expression	1/152	13/3792	0.412976	0.860867	101798286	EIF4EBP1	1
BP	GO:0010639	negative regulation of organelle organization	1/152	13/3792	0.412976	0.860867	101797044	LMOD2	1
BP	GO:0015988	energy coupled proton transmembrane transport, against electrochemical gradient	1/152	13/3792	0.412976	0.860867	101793897	ATP6V1E1	1
BP	GO:0015991	ATP hydrolysis coupled proton transport	1/152	13/3792	0.412976	0.860867	101793897	ATP6V1E1	1
BP	GO:0030154	cell differentiation	1/152	13/3792	0.412976	0.860867	101800783	LOC101800783	1
BP	GO:0031497	chromatin assembly	1/152	13/3792	0.412976	0.860867	101798499	LOC101798499	1
BP	GO:0090662	ATP hydrolysis coupled transmembrane transport	1/152	13/3792	0.412976	0.860867	101793897	ATP6V1E1	1
BP	GO:0099131	ATP hydrolysis coupled ion transmembrane transport	1/152	13/3792	0.412976	0.860867	101793897	ATP6V1E1	1
BP	GO:0099132	ATP hydrolysis coupled cation transmembrane transport	1/152	13/3792	0.412976	0.860867	101793897	ATP6V1E1	1
BP	GO:0006323	DNA packaging	1/152	14/3792	0.436587	0.860867	101798499	LOC101798499	1
BP	GO:0006333	chromatin assembly or disassembly	1/152	14/3792	0.436587	0.860867	101798499	LOC101798499	1
BP	GO:0008064	regulation of actin polymerization or depolymerization	1/152	14/3792	0.436587	0.860867	101797044	LMOD2	1
BP	GO:0008154	actin polymerization or depolymerization	1/152	14/3792	0.436587	0.860867	101797044	LMOD2	1
BP	GO:0030041	actin filament polymerization	1/152	14/3792	0.436587	0.860867	101797044	LMOD2	1
BP	GO:0030832	regulation of actin filament length	1/152	14/3792	0.436587	0.860867	101797044	LMOD2	1
BP	GO:0030833	regulation of actin filament polymerization	1/152	14/3792	0.436587	0.860867	101797044	LMOD2	1
BP	GO:0032271	regulation of protein polymerization	1/152	14/3792	0.436587	0.860867	101797044	LMOD2	1
BP	GO:0032535	regulation of cellular component size	1/152	14/3792	0.436587	0.860867	101797044	LMOD2	1
BP	GO:0032956	regulation of actin cytoskeleton organization	1/152	14/3792	0.436587	0.860867	101797044	LMOD2	1
BP	GO:0032970	regulation of actin filament-based process	1/152	14/3792	0.436587	0.860867	101797044	LMOD2	1
BP	GO:0032984	protein-containing complex disassembly	1/152	14/3792	0.436587	0.860867	101797044	LMOD2	1
BP	GO:0034728	nucleosome organization	1/152	14/3792	0.436587	0.860867	101798499	LOC101798499	1
BP	GO:0043087	regulation of GTPase activity	1/152	14/3792	0.436587	0.860867	101795631	LOC101795631	1
BP	GO:0043254	regulation of protein complex assembly	1/152	14/3792	0.436587	0.860867	101797044	LMOD2	1
BP	GO:0043624	cellular protein complex disassembly	1/152	14/3792	0.436587	0.860867	101797044	LMOD2	1
BP	GO:0048869	cellular developmental process	1/152	14/3792	0.436587	0.860867	101800783	LOC101800783	1

BP	GO:0051129	negative regulation of cellular component organization	1/152	14/3792	0.436587	0.860867	101797044	LMOD2	1
BP	GO:0110053	regulation of actin filament organization	1/152	14/3792	0.436587	0.860867	101797044	LMOD2	1
BP	GO:1902903	regulation of supramolecular fiber organization	1/152	14/3792	0.436587	0.860867	101797044	LMOD2	1
BP	GO:0030163	protein catabolic process	2/152	37/3792	0.440578	0.860867	101799480/101803700	UCHL1/PSMB1	2
BP	GO:0005975	carbohydrate metabolic process	4/152	85/3792	0.445946	0.860867	101795985/101798440/101792144/101800183	LOC101795985/LOC101798440/FUCA2/HYAL2	4
BP	GO:0006974	cellular response to DNA damage stimulus	3/152	62/3792	0.455904	0.860867	101795336/101796914/101802532	SHLD1/LIG1/MSH2	3
BP	GO:0033554	cellular response to stress	3/152	62/3792	0.455904	0.860867	101795336/101796914/101802532	SHLD1/LIG1/MSH2	3
BP	GO:0010605	negative regulation of macromolecule metabolic process	1/152	15/3792	0.459255	0.860867	101798286	EIF4EBP1	1
BP	GO:0022411	cellular component disassembly	1/152	15/3792	0.459255	0.860867	101797044	LMOD2	1
BP	GO:0034655	nucleobase-containing compound catabolic process	1/152	15/3792	0.459255	0.860867	101803472	ITPA	1
BP	GO:0044087	regulation of cellular component biogenesis	1/152	15/3792	0.459255	0.860867	101797044	LMOD2	1
BP	GO:0016579	protein deubiquitination	2/152	39/3792	0.467806	0.860867	101792575/101791234	USP44/USP49	2
BP	GO:0070646	protein modification by small protein removal	2/152	39/3792	0.467806	0.860867	101792575/101791234	USP44/USP49	2
BP	GO:0007015	actin filament organization	1/152	16/3792	0.481017	0.860867	101797044	LMOD2	1
BP	GO:0042157	lipoprotein metabolic process	1/152	16/3792	0.481017	0.860867	101798726	APOLD1	1
BP	GO:0097435	supramolecular fiber organization	1/152	16/3792	0.481017	0.860867	101797044	LMOD2	1
BP	GO:0044265	cellular macromolecule catabolic process	2/152	40/3792	0.481126	0.860867	101799480/101803700	UCHL1/PSMB1	2
BP	GO:0048583	regulation of response to stimulus	4/152	90/3792	0.49051	0.860867	101800755/101795336/101795713/101792754	SESN1/SHLD1/KALRN/BAMBI	4
BP	GO:0006486	protein glycosylation	2/152	41/3792	0.49424	0.860867	101803248/101793948	B3GALNT2/B3GNT2	2
BP	GO:0009100	glycoprotein metabolic process	2/152	41/3792	0.49424	0.860867	101803248/101793948	B3GALNT2/B3GNT2	2
BP	GO:0009101	glycoprotein biosynthetic process	2/152	41/3792	0.49424	0.860867	101803248/101793948	B3GALNT2/B3GNT2	2
BP	GO:0043413	macromolecule glycosylation	2/152	41/3792	0.49424	0.860867	101803248/101793948	B3GALNT2/B3GNT2	2
BP	GO:0070085	glycosylation	2/152	41/3792	0.49424	0.860867	101803248/101793948	B3GALNT2/B3GNT2	2
BP	GO:0055085	transmembrane transport	12/152	290/3792	0.499138	0.860867	101801029/101790978/101798013/101791662/101804595/101793897/101795224/119718656/101804200/101795086/101795852/119713969	SLC16A6/FLVCR1/MPC1/SLC9A6/SLC39A13/ATP6V1E1/SLC43A2/LOC119718656/SLC15A4/KCNQ4/SLC16A10/KCNC4	12
BP	GO:0009892	negative regulation of metabolic process	1/152	17/3792	0.501908	0.860867	101798286	EIF4EBP1	1
BP	GO:0032787	monocarboxylic acid metabolic process	1/152	17/3792	0.501908	0.860867	101797455	ASNS	1
BP	GO:0048193	Golgi vesicle transport	1/152	17/3792	0.501908	0.860867	101792755	LOC101792755	1
BP	GO:0051258	protein polymerization	1/152	17/3792	0.501908	0.860867	101797044	LMOD2	1
BP	GO:0090066	regulation of anatomical structure size	1/152	17/3792	0.501908	0.860867	101797044	LMOD2	1
BP	GO:0034220	ion transmembrane transport	2/152	42/3792	0.507145	0.86438	101798013/101793897	MPC1/ATP6V1E1	2
BP	GO:0006351	transcription, DNA-templated	16/152	393/3792	0.514163	0.86478	101805187/101794377/101799581/101801567/101790639/106019185/101801755/101801698/101794314/101797962/101802147/101792591/101789522/101796626/113845655/101791584	ATF3/MAFK/LOC101799581/TAF1D/CREM/CEBPB/MYF5/FOS/NFIL3/CREB5/L3MBTL3/LRRFIP1/MYOD1/JUN/JUND/SMAD6	16

BP	GO:0097659	nucleic acid-templated transcription	16/152	393/3792	0.514163	0.86478	101805187/101794377/101799581/101801567/101790639/106019185/101801755/101801698/101794314/101797962/101802147/101792591/101789522/101796626/113845655/101791584	ATF3/MAFK/LOC101799581/TAF1D/CREM/CEBPB/MYF5/FOS/NFIL3/CREB5/L3MBTL3/LRRFIP1/MYOD1/JUN/JUND/SMAD6	16
BP	GO:0009057	macromolecule catabolic process	2/152	43/3792	0.519836	0.86478	101799480/101803700	UCHL1/PSMB1	2
BP	GO:0032774	RNA biosynthetic process	16/152	395/3792	0.522837	0.86478	101805187/101794377/101799581/101801567/101790639/106019185/101801755/101801698/101794314/101797962/101802147/101792591/101789522/101796626/113845655/101791584	ATF3/MAFK/LOC101799581/TAF1D/CREM/CEBPB/MYF5/FOS/NFIL3/CREB5/L3MBTL3/LRRFIP1/MYOD1/JUN/JUND/SMAD6	16
BP	GO:0033036	macromolecule localization	5/152	119/3792	0.523335	0.86478	101802251/101804872/101792755/101798726/101801245	PITPNM1/LOC101804872/LOC101792755/APOLD1/SELENOS	5
BP	GO:0006629	lipid metabolic process	5/152	120/3792	0.530899	0.871961	101796431/101796497/101804872/101799246/101796880	PNPLA8/PNPLA2/LOC101804872/PLCG2/PLCD3	5
BP	GO:0051336	regulation of hydrolase activity	1/152	19/3792	0.541217	0.878262	101795631	LOC101795631	1
BP	GO:0065004	protein-DNA complex assembly	1/152	19/3792	0.541217	0.878262	101798499	LOC101798499	1
BP	GO:0051493	regulation of cytoskeleton organization	1/152	20/3792	0.559699	0.897506	101797044	LMOD2	1
BP	GO:0071824	protein-DNA complex subunit organization	1/152	20/3792	0.559699	0.897506	101798499	LOC101798499	1
BP	GO:0006811	ion transport	10/152	253/3792	0.567321	0.900791	101802251/101798013/101804872/101791662/101804595/101800466/101793897/101793641/101795086/119713969	PITPNM1/MPC1/LOC101804872/SLC9A6/SLC39A13/CHRNA10/ATP6V1E1/ATP13A2/KCNQ4/KCNC4	10
BP	GO:0051186	cofactor metabolic process	2/152	47/3792	0.568396	0.900791	101805057/101800658	HMOX1/PCBD1	2
BP	GO:0006457	protein folding	1/152	21/3792	0.577442	0.904548	101801226	DNAJA4	1
BP	GO:0071103	DNA conformation change	1/152	21/3792	0.577442	0.904548	101798499	LOC101798499	1
BP	GO:0035556	intracellular signal transduction	7/152	181/3792	0.59563	0.921926	101795713/101799246/101799960/101795631/101795633/101796880/101791200	KALRN/PLCG2/RCAN1/LOC101795631/RAF1/PLCD3/SOCS3	7
BP	GO:0044281	small molecule metabolic process	7/152	181/3792	0.59563	0.921926	101795985/101799450/101797455/101803472/101797377/101797078/101800658	LOC101795985/GLUL/ASNS/ITPA/GLUD1/IP6K2/PCBD1	7
BP	GO:0019438	aromatic compound biosynthetic process	18/152	464/3792	0.598741	0.921926	101805187/101794377/101799581/101801567/101790639/106019185/101801755/101801698/101794314/101797962/101802147/101792591/101789522/101803472/101796626/113845655/101800658/101791584	ATF3/MAFK/LOC101799581/TAF1D/CREM/CEBPB/MYF5/FOS/NFIL3/CREB5/L3MBTL3/LRRFIP1/MYOD1/ITPA/JUN/JUND/PCBD1/SMAD6	18

BP	GO:0018130	heterocycle biosynthetic process	18/152	469/3792	0.618011	0.94622	101805187/101794377/101799581/101801567/101790639/106019185/101801755/101801698/101794314/101797962/101802147/101792591/101789522/101803472/101796626/113845655/101800658/101791584	ATF3/MAFK/LOC101799581/TAF1D/CREM/CEBPB/MYF5/FOS/NFIL3/CREB5/L3MBTL3/LRRFIP1/MYOD1/ITPA/JUN/JUND/PCBD1/SMAD6	18
BP	GO:0006357	regulation of transcription by RNA polymerase II	1/152	24/3792	0.62652	0.953859	101799581	LOC101799581	1
BP	GO:1901362	organic cyclic compound biosynthetic process	18/152	475/3792	0.640597	0.955532	101805187/101794377/101799581/101801567/101790639/106019185/101801755/101801698/101794314/101797962/101802147/101792591/101789522/101803472/101796626/113845655/101800658/101791584	ATF3/MAFK/LOC101799581/TAF1D/CREM/CEBPB/MYF5/FOS/NFIL3/CREB5/L3MBTL3/LRRFIP1/MYOD1/ITPA/JUN/JUND/PCBD1/SMAD6	18
BP	GO:0033043	regulation of organelle organization	1/152	25/3792	0.641586	0.955532	101797044	LMOD2	1
BP	GO:0019752	carboxylic acid metabolic process	3/152	82/3792	0.646059	0.955532	101799450/101797455/101797377	GLUL/ASNS/GLUD1	3
BP	GO:0034654	nucleobase-containing compound biosynthetic process	17/152	452/3792	0.650995	0.955532	101805187/101794377/101799581/101801567/101790639/106019185/101801755/101801698/101794314/101797962/101802147/101792591/101789522/101803472/101796626/113845655/101791584	ATF3/MAFK/LOC101799581/TAF1D/CREM/CEBPB/MYF5/FOS/NFIL3/CREB5/L3MBTL3/LRRFIP1/MYOD1/ITPA/JUN/JUND/SMAD6	17
BP	GO:0006082	organic acid metabolic process	3/152	83/3792	0.654244	0.955532	101799450/101797455/101797377	GLUL/ASNS/GLUD1	3
BP	GO:0043436	oxoacid metabolic process	3/152	83/3792	0.654244	0.955532	101799450/101797455/101797377	GLUL/ASNS/GLUD1	3
BP	GO:0046903	secretion	1/152	26/3792	0.656048	0.955532	101804872	LOC101804872	1
BP	GO:0070647	protein modification by small protein conjugation or removal	2/152	56/3792	0.664417	0.955532	101792575/101791234	USP44/USP49	2
BP	GO:0009108	coenzyme biosynthetic process	1/152	27/3792	0.66993	0.955532	101800658	PCBD1	1
BP	GO:0030029	actin filament-based process	1/152	27/3792	0.66993	0.955532	101797044	LMOD2	1
BP	GO:0030036	actin cytoskeleton organization	1/152	27/3792	0.66993	0.955532	101797044	LMOD2	1
BP	GO:0051128	regulation of cellular component organization	1/152	27/3792	0.66993	0.955532	101797044	LMOD2	1
BP	GO:0034622	cellular protein-containing complex assembly	2/152	58/3792	0.683285	0.963466	101798499/101797044	LOC101798499/LMOD2	2
BP	GO:0006812	cation transport	4/152	116/3792	0.691823	0.963466	101791662/101804595/101793897/101793641	SLC9A6/SLC39A13/ATP6V1E1/ATP13A2	4
BP	GO:0009141	nucleoside triphosphate metabolic process	1/152	29/3792	0.696047	0.963466	101803472	ITPA	1
BP	GO:1902600	proton transmembrane transport	1/152	29/3792	0.696047	0.963466	101793897	ATP6V1E1	1
BP	GO:0007018	microtubule-based movement	2/152	60/3792	0.701283	0.963466	119714941/101803768	LOC119714941/KIF16B	2

BP	GO:0006511	ubiquitin-dependent protein catabolic process	1/152	30/3792	0.708324	0.963466	101799480	UCHL1	1
BP	GO:0019941	modification-dependent protein catabolic process	1/152	30/3792	0.708324	0.963466	101799480	UCHL1	1
BP	GO:0043632	modification-dependent macromolecule catabolic process	1/152	30/3792	0.708324	0.963466	101799480	UCHL1	1
BP	GO:0007155	cell adhesion	4/152	119/3792	0.711045	0.963466	101793322/101798168/101789534/101800667	THBS1/TNFAIP6/CDHR5/CDH2	4
BP	GO:0022610	biological adhesion	4/152	119/3792	0.711045	0.963466	101793322/101798168/101789534/101800667	THBS1/TNFAIP6/CDHR5/CDH2	4
BP	GO:0006732	coenzyme metabolic process	1/152	31/3792	0.720109	0.970894	101800658	PCBD1	1
BP	GO:0006325	chromatin organization	1/152	32/3792	0.731421	0.977537	101798499	LOC101798499	1
BP	GO:0045454	cell redox homeostasis	1/152	33/3792	0.742278	0.977537	101804092	TXN	1
BP	GO:0051260	protein homooligomerization	1/152	33/3792	0.742278	0.977537	119713969	KCNC4	1
BP	GO:0006928	movement of cell or subcellular component	2/152	67/3792	0.757722	0.977537	119714941/101803768	LOC119714941/KIF16B	2
BP	GO:0051188	cofactor biosynthetic process	1/152	35/3792	0.762702	0.977537	101800658	PCBD1	1
BP	GO:0065003	protein-containing complex assembly	3/152	100/3792	0.772971	0.977537	101798499/101797044/119713969	LOC101798499/LMOD2/KCNC4	3
BP	GO:0065008	regulation of biological quality	2/152	70/3792	0.778969	0.977537	101797044/101804092	LMOD2/TXN	2
BP	GO:0019637	organophosphate metabolic process	4/152	131/3792	0.779221	0.977537	101798440/101804872/101803472/101797078	LOC101798440/LOC101804872/ITPA/IP6K2	4
BP	GO:0002376	immune system process	1/152	37/3792	0.781517	0.977537	101803777	TNFSF13B	1
BP	GO:0006366	transcription by RNA polymerase II	1/152	37/3792	0.781517	0.977537	101799581	LOC101799581	1
BP	GO:0006955	immune response	1/152	37/3792	0.781517	0.977537	101803777	TNFSF13B	1
BP	GO:0019725	cellular homeostasis	1/152	37/3792	0.781517	0.977537	101804092	TXN	1
BP	GO:0006259	DNA metabolic process	3/152	103/3792	0.790072	0.977537	101795336/101796914/101802532	SHLD1/LIG1/MSH2	3
BP	GO:0017144	drug metabolic process	1/152	39/3792	0.79885	0.977537	101800658	PCBD1	1
BP	GO:0043933	protein-containing complex subunit organization	3/152	106/3792	0.8061	0.977537	101798499/101797044/119713969	LOC101798499/LMOD2/KCNC4	3
BP	GO:0051259	protein complex oligomerization	1/152	40/3792	0.806997	0.977537	119713969	KCNC4	1
BP	GO:0098655	cation transmembrane transport	1/152	40/3792	0.806997	0.977537	101793897	ATP6V1E1	1
BP	GO:0098660	inorganic ion transmembrane transport	1/152	40/3792	0.806997	0.977537	101793897	ATP6V1E1	1
BP	GO:0098662	inorganic cation transmembrane transport	1/152	40/3792	0.806997	0.977537	101793897	ATP6V1E1	1
BP	GO:0042592	homeostatic process	1/152	41/3792	0.814815	0.977537	101804092	TXN	1
BP	GO:0006886	intracellular protein transport	2/152	76/3792	0.816642	0.977537	101792755/101801245	LOC101792755/SELEN	2
BP	GO:0007156	homophilic cell adhesion via plasma membrane adhesion molecules	2/152	78/3792	0.827871	0.977537	101789534/101800667	CDHR5/CDH2	2
BP	GO:0098609	cell-cell adhesion	2/152	78/3792	0.827871	0.977537	101789534/101800667	CDHR5/CDH2	2
BP	GO:0098742	cell-cell adhesion via plasma-membrane adhesion molecules	2/152	78/3792	0.827871	0.977537	101789534/101800667	CDHR5/CDH2	2
BP	GO:0022607	cellular component assembly	3/152	113/3792	0.839554	0.977537	101798499/101797044/119713969	LOC101798499/LMOD2/KCNC4	3
BP	GO:0007266	Rho protein signal transduction	1/152	45/3792	0.843067	0.977537	101795713	KALRN	1
BP	GO:0035023	regulation of Rho protein signal transduction	1/152	45/3792	0.843067	0.977537	101795713	KALRN	1
BP	GO:0007017	microtubule-based process	2/152	81/3792	0.843564	0.977537	119714941/101803768	LOC119714941/KIF16B	2
BP	GO:0009966	regulation of signal transduction	2/152	83/3792	0.853298	0.977537	101795713/101792754	KALRN/BAMBI	2
BP	GO:0010646	regulation of cell communication	2/152	83/3792	0.853298	0.977537	101795713/101792754	KALRN/BAMBI	2
BP	GO:0023051	regulation of signaling	2/152	83/3792	0.853298	0.977537	101795713/101792754	KALRN/BAMBI	2
BP	GO:0034613	cellular protein localization	2/152	83/3792	0.853298	0.977537	101792755/101801245	LOC101792755/SELEN	2
BP	GO:0070727	cellular macromolecule localization	2/152	83/3792	0.853298	0.977537	101792755/101801245	LOC101792755/SELEN	2
BP	GO:0006644	phospholipid metabolic process	1/152	48/3792	0.861406	0.977537	101804872	LOC101804872	1

BP	GO:1901135	carbohydrate derivative metabolic process	3/152	122/3792	0.875203	0.977537	101798440/101803248/101793948	LOC101798440/B3GALNT2/B3GNT2	3
BP	GO:0015031	protein transport	2/152	88/3792	0.875278	0.977537	101792755/101801245	LOC101792755/SELEN	2
BP	GO:0015833	peptide transport	2/152	88/3792	0.875278	0.977537	101792755/101801245	LOC101792755/SELEN	2
BP	GO:0042886	amide transport	2/152	89/3792	0.879295	0.977537	101792755/101801245	LOC101792755/SELEN	2
BP	GO:0045184	establishment of protein localization	2/152	89/3792	0.879295	0.977537	101792755/101801245	LOC101792755/SELEN	2
BP	GO:1901137	carbohydrate derivative biosynthetic process	2/152	89/3792	0.879295	0.977537	101803248/101793948	B3GALNT2/B3GNT2	2
BP	GO:0046907	intracellular transport	2/152	90/3792	0.883193	0.977537	101792755/101801245	LOC101792755/SELEN	2
BP	GO:0016192	vesicle-mediated transport	2/152	91/3792	0.886976	0.977537	101800849/101792755	VAMP4/LOC101792755	2
BP	GO:0051649	establishment of localization in cell	2/152	91/3792	0.886976	0.977537	101792755/101801245	LOC101792755/SELEN	2
BP	GO:0007265	Ras protein signal transduction	1/152	53/3792	0.887358	0.977537	101795713	KALRN	1
BP	GO:0046578	regulation of Ras protein signal transduction	1/152	53/3792	0.887358	0.977537	101795713	KALRN	1
BP	GO:0007010	cytoskeleton organization	1/152	55/3792	0.896332	0.979576	101797044	LMOD2	1
BP	GO:0008104	protein localization	2/152	94/3792	0.897657	0.979576	101792755/101801245	LOC101792755/SELEN	2
BP	GO:1901566	organonitrogen compound biosynthetic process	7/152	261/3792	0.909062	0.979576	101798286/101803248/101799450/101793948/101797455/101793553/101800658	EIF4EBP1/B3GALNT2/GLUL/B3GNT2/ASNS/EIF1/PCBD1	7
BP	GO:0015672	monovalent inorganic cation transport	1/152	59/3792	0.912203	0.979576	101793897	ATP6V1E1	1
BP	GO:0051276	chromosome organization	1/152	59/3792	0.912203	0.979576	101798499	LOC101798499	1
BP	GO:0051056	regulation of small GTPase mediated signal transduction	1/152	60/3792	0.915778	0.979576	101795713	KALRN	1
BP	GO:0030001	metal ion transport	1/152	61/3792	0.919208	0.979576	101804595	SLC39A13	1
BP	GO:0044085	cellular component biogenesis	3/152	137/3792	0.919313	0.979576	101798499/101797044/119713969	LOC101798499/LMOD2/KCNC4	3
BP	GO:0071705	nitrogen compound transport	2/152	102/3792	0.921741	0.979576	101792755/101801245	LOC101792755/SELEN	2
BP	GO:1902531	regulation of intracellular signal transduction	1/152	63/3792	0.925658	0.979895	101795713	KALRN	1
BP	GO:0043604	amide biosynthetic process	3/152	143/3792	0.932598	0.983165	101798286/101797455/101793553	EIF4EBP1/ASNS/EIF1	3
BP	GO:0051641	cellular localization	2/152	109/3792	0.938359	0.983165	101792755/101801245	LOC101792755/SELEN	2
BP	GO:0044255	cellular lipid metabolic process	1/152	68/3792	0.93963	0.983165	101804872	LOC101804872	1
BP	GO:0043603	cellular amide metabolic process	3/152	149/3792	0.943856	0.983788	101798286/101797455/101793553	EIF4EBP1/ASNS/EIF1	3
BP	GO:0006753	nucleoside phosphate metabolic process	1/152	72/3792	0.948903	0.985259	101803472	ITPA	1
BP	GO:0007264	small GTPase mediated signal transduction	1/152	79/3792	0.961853	0.988777	101795713	KALRN	1
BP	GO:0055086	nucleobase-containing small molecule metabolic process	1/152	82/3792	0.96635	0.988777	101803472	ITPA	1
BP	GO:0090407	organophosphate biosynthetic process	1/152	84/3792	0.969051	0.988777	101797078	IP6K2	1
BP	GO:0007186	G-protein-coupled receptor signaling pathway	5/152	249/3792	0.976182	0.988777	101789648/101802610/101790005/101794938/101801041	GPR157/CCR9/LOC101790005/ADGRD1/VIPR2	5
BP	GO:0006412	translation	2/152	137/3792	0.976996	0.988777	101798286/101793553	EIF4EBP1/EIF1	2
BP	GO:0043043	peptide biosynthetic process	2/152	140/3792	0.979355	0.988777	101798286/101793553	EIF4EBP1/EIF1	2
BP	GO:0006996	organelle organization	2/152	142/3792	0.980796	0.988777	101798499/101797044	LOC101798499/LMOD2	2
BP	GO:0006518	peptide metabolic process	2/152	143/3792	0.981479	0.988777	101798286/101793553	EIF4EBP1/EIF1	2
BP	GO:0016043	cellular component organization	3/152	230/3792	0.996173	0.998375	101798499/101797044/119713969	LOC101798499/LMOD2/KCNC4	3
BP	GO:0071840	cellular component organization or biogenesis	3/152	254/3792	0.998375	0.998375	101798499/101797044/119713969	LOC101798499/LMOD2/KCNC4	3

CC	GO:0005576	extracellular region	11/89	158/2288	0.039578	0.992587	101794528/101790193/101799001/101793322/101803050/113839877/101803685/novel.277/101799824/101792356/101798726	LOC101794528/CCN2/CCN1/THBS1/CRH/ECM1/IGFBP3/-/WNT2/WNT5B/APOLD1	11
CC	GO:0005634	nucleus	16/89	334/2288	0.217121	0.992587	101800755/101801468/101794377/101800783/101799581/101798499/101801567/101801755/101792368/101794314/101802147/101789522/101802149/106018371/101791694/101796848	SESN1/CBFB/MAFK/LOC101800783/LOC101799581/LOC101798499/TAF1D/MYF5/CCNA2/NFIL3/L3MBTL3/MYOD1/ADRM1/NOL7/CDKN1B/CCNL2	16
CC	GO:0031410	cytoplasmic vesicle	2/89	23/2288	0.224466	0.992587	101790234/101792755	CHGA/LOC101792755	2
CC	GO:0031982	vesicle	2/89	23/2288	0.224466	0.992587	101790234/101792755	CHGA/LOC101792755	2
CC	GO:0097708	intracellular vesicle	2/89	23/2288	0.224466	0.992587	101790234/101792755	CHGA/LOC101792755	2
CC	GO:0043227	membrane-bounded organelle	22/89	498/2288	0.283285	0.992587	101798341/101800755/101801468/101794377/101800783/101799581/101790565/101798499/101801567/101798013/101801755/101792368/101794314/101790234/101802147/101792755/101789522/101802149/106018371/101791694/101796848/101801245	BNIP3/SESN1/CBFB/MAFK/LOC101800783/LOC101799581/LOC101790565/LOC101798499/TAF1D/MPC1/MYF5/CCNA2/NFIL3/CHGA/L3MBTL3/LOC101792755/MYOD1/ADRM1/NOL7/CDKN1B/CCNL2/SELENOS	22
CC	GO:0044429	mitochondrial part	3/89	52/2288	0.3295	0.992587	101798341/101790565/101798013	BNIP3/LOC101790565/MPC1	3
CC	GO:0000775	chromosome, centromeric region	1/89	11/2288	0.35429	0.992587	101791211	ZWILCH	1
CC	GO:0030658	transport vesicle membrane	1/89	11/2288	0.35429	0.992587	101792755	LOC101792755	1
CC	GO:0005839	proteasome core complex	1/89	12/2288	0.379529	0.992587	101803700	PSMB1	1
CC	GO:0030133	transport vesicle	1/89	12/2288	0.379529	0.992587	101792755	LOC101792755	1
CC	GO:0098687	chromosomal region	1/89	13/2288	0.403791	0.992587	101791211	ZWILCH	1
CC	GO:0043231	intracellular membrane-bounded organelle	20/89	483/2288	0.416349	0.992587	101798341/101800755/101801468/101794377/101800783/101799581/101790565/101798499/101801567/101798013/101801755/101792368/101794314/101802147/101789522/101802149/106018371/101791694/101796848/101801245	BNIP3/SESN1/CBFB/MAFK/LOC101800783/LOC101799581/LOC101790565/LOC101798499/TAF1D/MPC1/MYF5/CCNA2/NFIL3/L3MBTL3/MYOD1/ADRM1/NOL7/CDKN1B/CCNL2/SELENOS	20
CC	GO:0031012	extracellular matrix	1/89	14/2288	0.427116	0.992587	101794528	LOC101794528	1
CC	GO:0000502	proteasome complex	1/89	15/2288	0.449537	0.992587	101803700	PSMB1	1
CC	GO:0005798	Golgi-associated vesicle	1/89	15/2288	0.449537	0.992587	101792755	LOC101792755	1
CC	GO:0005882	intermediate filament	1/89	15/2288	0.449537	0.992587	119717442	DES	1
CC	GO:0012506	vesicle membrane	1/89	15/2288	0.449537	0.992587	101792755	LOC101792755	1
CC	GO:0030120	vesicle coat	1/89	15/2288	0.449537	0.992587	101792755	LOC101792755	1
CC	GO:0030135	coated vesicle	1/89	15/2288	0.449537	0.992587	101792755	LOC101792755	1

CC	GO:0030659	cytoplasmic vesicle membrane	1/89	15/2288	0.449537	0.992587	101792755	LOC101792755	1
CC	GO:0030660	Golgi-associated vesicle membrane	1/89	15/2288	0.449537	0.992587	101792755	LOC101792755	1
CC	GO:0030662	coated vesicle membrane	1/89	15/2288	0.449537	0.992587	101792755	LOC101792755	1
CC	GO:0045111	intermediate filament cytoskeleton	1/89	15/2288	0.449537	0.992587	119717442	DES	1
CC	GO:1905369	endopeptidase complex	1/89	15/2288	0.449537	0.992587	101803700	PSMB1	1
CC	GO:0044421	extracellular region part	2/89	39/2288	0.452671	0.992587	101794528/113839877	LOC101794528/ECM1	2
CC	GO:0005739	mitochondrion	3/89	65/2288	0.467958	0.992587	101798341/101790565/101798013	BNIP3/LOC101790565/MPC1	3
CC	GO:0005667	transcription factor complex	1/89	17/2288	0.491809	0.992587	101801567	TAF1D	1
CC	GO:0099513	polymeric cytoskeletal fiber	1/89	17/2288	0.491809	0.992587	119717442	DES	1
CC	GO:1905368	peptidase complex	1/89	17/2288	0.491809	0.992587	101803700	PSMB1	1
CC	GO:0005740	mitochondrial envelope	2/89	44/2288	0.516757	0.992587	101798341/101798013	BNIP3/MPC1	2
CC	GO:0044433	cytoplasmic vesicle part	1/89	19/2288	0.530869	0.992587	101792755	LOC101792755	1
CC	GO:0031967	organelle envelope	2/89	50/2288	0.586939	0.992587	101798341/101798013	BNIP3/MPC1	2
CC	GO:0031975	envelope	2/89	50/2288	0.586939	0.992587	101798341/101798013	BNIP3/MPC1	2
CC	GO:0016469	proton-transporting two-sector ATPase complex	1/89	23/2288	0.600299	0.992587	101793897	ATP6V1E1	1
CC	GO:0099080	supramolecular complex	1/89	23/2288	0.600299	0.992587	119717442	DES	1
CC	GO:0099081	supramolecular polymer	1/89	23/2288	0.600299	0.992587	119717442	DES	1
CC	GO:0099512	supramolecular fiber	1/89	23/2288	0.600299	0.992587	119717442	DES	1
CC	GO:0031974	membrane-enclosed lumen	3/89	82/2288	0.627373	0.992587	101799581/101790565/101801567	LOC101799581/LOC101790565/TAF1D	3
CC	GO:0043233	organelle lumen	3/89	82/2288	0.627373	0.992587	101799581/101790565/101801567	LOC101799581/LOC101790565/TAF1D	3
CC	GO:0070013	intracellular organelle lumen	3/89	82/2288	0.627373	0.992587	101799581/101790565/101801567	LOC101799581/LOC101790565/TAF1D	3
CC	GO:0005615	extracellular space	1/89	25/2288	0.6311	0.992587	113839877	ECM1	1
CC	GO:0005789	endoplasmic reticulum membrane	1/89	26/2288	0.645608	0.992587	101801245	SELENOS	1
CC	GO:0030117	membrane coat	1/89	26/2288	0.645608	0.992587	101792755	LOC101792755	1
CC	GO:0048475	coated membrane	1/89	26/2288	0.645608	0.992587	101792755	LOC101792755	1
CC	GO:0098827	endoplasmic reticulum subcompartment	1/89	26/2288	0.645608	0.992587	101801245	SELENOS	1
CC	GO:0005743	mitochondrial inner membrane	1/89	27/2288	0.659552	0.992587	101798013	MPC1	1
CC	GO:0019866	organelle inner membrane	1/89	27/2288	0.659552	0.992587	101798013	MPC1	1
CC	GO:0042175	nuclear outer membrane-endoplasmic reticulum membrane network	1/89	27/2288	0.659552	0.992587	101801245	SELENOS	1
CC	GO:0044432	endoplasmic reticulum part	1/89	27/2288	0.659552	0.992587	101801245	SELENOS	1
CC	GO:0012505	endomembrane system	3/89	90/2288	0.690324	0.992587	101790234/101792755/101801245	CHGA/LOC101792755/SELENOS	3
CC	GO:0098798	mitochondrial protein complex	1/89	31/2288	0.710105	0.992587	101790565	LOC101790565	1
CC	GO:0044427	chromosomal part	2/89	69/2288	0.759174	0.992587	101798499/101791211	LOC101798499/ZWILC	2
CC	GO:0031981	nuclear lumen	2/89	72/2288	0.779909	0.992587	101799581/101801567	LOC101799581/TAF1D	2
CC	GO:0031984	organelle subcompartment	1/89	38/2288	0.781341	0.992587	101801245	SELENOS	1
CC	GO:0098805	whole membrane	1/89	38/2288	0.781341	0.992587	101792755	LOC101792755	1
CC	GO:0005783	endoplasmic reticulum	1/89	40/2288	0.7983	0.992587	101801245	SELENOS	1
CC	GO:0031966	mitochondrial membrane	1/89	40/2288	0.7983	0.992587	101798013	MPC1	1
CC	GO:0031090	organelle membrane	2/89	76/2288	0.805145	0.992587	101798013/101792755	MPC1/LOC101792755	2
CC	GO:0000786	nucleosome	1/89	41/2288	0.806286	0.992587	101798499	LOC101798499	1
CC	GO:0032993	protein-DNA complex	1/89	42/2288	0.813959	0.992587	101798499	LOC101798499	1
CC	GO:0044815	DNA packaging complex	1/89	42/2288	0.813959	0.992587	101798499	LOC101798499	1
CC	GO:0005694	chromosome	2/89	78/2288	0.816787	0.992587	101798499/101791211	LOC101798499/ZWILC	2
CC	GO:0000785	chromatin	1/89	46/2288	0.841757	0.992587	101798499	LOC101798499	1
CC	GO:0098588	bounding membrane of organelle	1/89	49/2288	0.859872	0.992587	101792755	LOC101792755	1

CC	GO:0005654	nucleoplasm	1/89	53/2288	0.880872	0.992587	101799581	LOC101799581	1
CC	GO:0044451	nucleoplasm part	1/89	53/2288	0.880872	0.992587	101799581	LOC101799581	1
CC	GO:0005737	cytoplasm	10/89	351/2288	0.897724	0.992587	101798341/101794693/101790565/101803288/101798013/101790234/106014280/101792755/101802149/101801245	BNIP3/DBNDD2/LOC101790565/LOC101803288/MPC1/CHGA/PPA2/LOC101792755/ADRM1/SELENOS	10
CC	GO:0044428	nuclear part	2/89	98/2288	0.903277	0.992587	101799581/101801567	LOC101799581/TAF1D	2
CC	GO:0098796	membrane protein complex	2/89	109/2288	0.932958	0.992587	101793897/101792755	ATP6V1E1/LOC1017927	2
CC	GO:0044446	intracellular organelle part	10/89	381/2288	0.944775	0.992587	101798341/119717442/101799581/101790565/101798499/101801567/101798013/101792755/101801245/101791211	BNIP3/DES/LOC101799581/LOC101790565/LOC101798499/TAF1D/MPC1/LOC101792755/SELENOS/ZWILCH	10
CC	GO:0044422	organelle part	10/89	383/2288	0.947118	0.992587	101798341/119717442/101799581/101790565/101798499/101801567/101798013/101792755/101801245/101791211	BNIP3/DES/LOC101799581/LOC101790565/LOC101798499/TAF1D/MPC1/LOC101792755/SELENOS/ZWILCH	10
CC	GO:0044444	cytoplasmic part	6/89	281/2288	0.971545	0.992587	101798341/101790565/101798013/101790234/101792755/101801245	BNIP3/LOC101790565/MPC1/CHGA/LOC101792755/SELENOS	6
CC	GO:0005840	ribosome	1/89	89/2288	0.972738	0.992587	101790565	LOC101790565	1
CC	GO:1902494	catalytic complex	1/89	95/2288	0.978729	0.992587	101803700	PSMB1	1
CC	GO:0044430	cytoskeletal part	1/89	100/2288	0.982713	0.992587	119717442	DES	1
CC	GO:1990904	ribonucleoprotein complex	1/89	111/2288	0.989063	0.992587	101790565	LOC101790565	1
CC	GO:0005856	cytoskeleton	1/89	119/2288	0.992173	0.992587	119717442	DES	1
CC	GO:0043228	non-membrane-bounded organelle	5/89	292/2288	0.992587	0.992587	119717442/101790565/101798499/101801567/101791211	DES/LOC101790565/LOC101798499/TAF1D/ZWILCH	5
CC	GO:0043232	intracellular non-membrane-bounded organelle	5/89	292/2288	0.992587	0.992587	119717442/101790565/101798499/101801567/101791211	DES/LOC101790565/LOC101798499/TAF1D/ZWILCH	5
MF	GO:0008289	lipid binding	9/250	77/6527	0.002542	0.239281	101790249/101794730/101792273/101800869/101791151/101792242/101803768/101798726/101789868	ANXA1/ANXA11/ANXA2/ANXA7/STARD6/SNX16/KIF16B/APOLD1/PXK	9
MF	GO:0005543	phospholipid binding	7/250	51/6527	0.003044	0.239281	101790249/101794730/101792273/101800869/101792242/101803768/101789868	ANXA1/ANXA11/ANXA2/ANXA7/SNX16/KIF16B/PXK	7
MF	GO:0008234	cysteine-type peptidase activity	8/250	67/6527	0.003818	0.239281	101803168/101799480/101792575/101804246/113839605/101794268/101791234/101794402	CTSL/UCHL1/USP44/CTSZ/LOC113839605/CTSB/USP49/TINAG	8
MF	GO:0005520	insulin-like growth factor binding	3/250	14/6527	0.014772	0.55543	101790193/101799001/101803685	CCN2/CCN1/IGFBP3	3
MF	GO:0019838	growth factor binding	3/250	14/6527	0.014772	0.55543	101790193/101799001/101803685	CCN2/CCN1/IGFBP3	3

MF	GO:0016301	kinase activity	23/250	395/6527	0.028331	0.748671	101793905/101803371/101795985/101797616/101803562/101803181/101791030/101804342/101800942/101802737/101793068/101795713/101790169/101792645/101797446/101795633/101801641/101791769/106017602/101791037/101796806/101797078/101789868	ULK2/MAP2K3/LOC101795985/LOC101797616/TRIB1/MAP3K6/STYK1/SIK1/RPS6KA1/CLK1/PHKG1/KALRN/LOC101790169/PIM3/OXSR1/RAF1/LOC101801641/MAP3K8/TSSK2/MAP2K1/CDK10/IP6K2/PXK	23
MF	GO:0004672	protein kinase activity	21/250	353/6527	0.028861	0.748671	101793905/101803371/101797616/101803562/101803181/101791030/101804342/101800942/101802737/101793068/101795713/101790169/101792645/101797446/101795633/101801641/101791769/106017602/101791037/101796806/101789868	ULK2/MAP2K3/LOC101797616/TRIB1/MAP3K6/STYK1/SIK1/RPS6KA1/CLK1/PHKG1/KALRN/LOC101790169/PIM3/OXSR1/RAF1/LOC101801641/MAP3K8/TSSK2/MAP2K1/CDK10/PXK	21
MF	GO:0070011	peptidase activity, acting on L-amino acid peptides	16/250	252/6527	0.031858	0.748671	101803168/101794528/101799480/101794584/101792575/101804552/101804246/113839605/101794820/101791397/101794268/101791234/101797577/101794402/101803700/101790574	CTSL/LOC101794528/UCHL1/ADAMTS17/USP44/PLAU/CTSZ/LOC113839605/LOC101794820/DPP7/CTSB/USP49/SCPEP1/TINAG/PSMB1/ADAMTS5	16
MF	GO:0016773	phosphotransferase activity, alcohol group as acceptor	22/250	387/6527	0.039745	0.830237	101793905/101803371/101795985/101797616/101803562/101803181/101791030/101804342/101800942/101802737/101793068/101795713/101790169/101792645/101797446/101795633/101801641/101791769/106017602/101791037/101796806/101789868	ULK2/MAP2K3/LOC101795985/LOC101797616/TRIB1/MAP3K6/STYK1/SIK1/RPS6KA1/CLK1/PHKG1/KALRN/LOC101790169/PIM3/OXSR1/RAF1/LOC101801641/MAP3K8/TSSK2/MAP2K1/CDK10/PXK	22
MF	GO:0008233	peptidase activity	16/250	263/6527	0.044422	0.835136	101803168/101794528/101799480/101794584/101792575/101804552/101804246/113839605/101794820/101791397/101794268/101791234/101797577/101794402/101803700/101790574	CTSL/LOC101794528/UCHL1/ADAMTS17/USP44/PLAU/CTSZ/LOC113839605/LOC101794820/DPP7/CTSB/USP49/SCPEP1/TINAG/PSMB1/ADAMTS5	16
MF	GO:0008378	galactosyltransferase activity	2/250	10/6527	0.053681	0.875467	101803248/101793948	B3GALNT2/B3GNT2	2

MF	GO:0051287	NAD binding	3/250	23/6527	0.055881	0.875467	101790158/101798440/101789745	HIBADH/LOC101798440/ME3	3
MF	GO:0004435	phosphatidylinositol phospholipase C activity	2/250	12/6527	0.074899	0.965641	101799246/101796880	PLCG2/PLCD3	2
MF	GO:0004629	phospholipase C activity	2/250	12/6527	0.074899	0.965641	101799246/101796880	PLCG2/PLCD3	2
MF	GO:0004620	phospholipase activity	3/250	30/6527	0.105691	0.965641	101804872/101799246/101796880	LOC101804872/PLCG2/PLCD3	3
MF	GO:0016298	lipase activity	3/250	30/6527	0.105691	0.965641	101804872/101799246/101796880	LOC101804872/PLCG2/PLCD3	3
MF	GO:0016879	ligase activity, forming carbon-nitrogen bonds	2/250	16/6527	0.123369	0.965641	101799450/101797455	GLUL/ASNS	2
MF	GO:0004857	enzyme inhibitor activity	4/250	50/6527	0.123443	0.965641	101792619/101794822/101796096/101791694	TIMP4/TFPI/LOC101796096/CDKN1B	4
MF	GO:0016772	transferase activity, transferring phosphorus-containing groups	23/250	471/6527	0.134317	0.965641	101793905/101803371/101795985/101797616/101803562/101803181/101791030/101804342/101800942/101802737/101793068/101795713/101790169/101792645/101797446/101795633/101801641/101791769/106017602/101791037/101796806/101797078/101789868	ULK2/MAP2K3/LOC101795985/LOC101797616/TRIB1/MAP3K6/STYK1/SIK1/RPS6KA1/CLK1/PHKG1/KALRN/LOC101790169/PIM3/OXSR1/RAF1/LOC101801641/MAP3K8/TSSK2/MAP2K1/CDK10/IP6K2/PXK	23
MF	GO:0052689	carboxylic ester hydrolase activity	2/250	17/6527	0.136437	0.965641	101803288/101804872	LOC101803288/LOC101804872	2
MF	GO:0004674	protein serine/threonine kinase activity	4/250	53/6527	0.144054	0.965641	101793905/101800942/101797446/101801641	ULK2/RPS6KA1/OXSR1/LOC101801641	4
MF	GO:0004866	endopeptidase inhibitor activity	3/250	38/6527	0.176947	0.965641	101792619/101794822/101796096	TIMP4/TFPI/LOC101796096	3
MF	GO:0035091	phosphatidylinositol binding	3/250	38/6527	0.176947	0.965641	101792242/101803768/101789868	SNX16/KIF16B/PXK	3
MF	GO:0061135	endopeptidase regulator activity	3/250	38/6527	0.176947	0.965641	101792619/101794822/101796096	TIMP4/TFPI/LOC101796096	3
MF	GO:0030414	peptidase inhibitor activity	3/250	39/6527	0.186641	0.965641	101792619/101794822/101796096	TIMP4/TFPI/LOC101796096	3
MF	GO:0061134	peptidase regulator activity	3/250	39/6527	0.186641	0.965641	101792619/101794822/101796096	TIMP4/TFPI/LOC101796096	3
MF	GO:0016491	oxidoreductase activity	14/250	286/6527	0.206438	0.965641	101793380/101794631/113839590/101795743/101798440/101805057/101789745/101793275/119713271/101789907/101792573/101801193/101791774/101797377	AASS/LOC101794631/LOC113839590/NDUFAF2/LOC101798440/HMOX1/ME3/ALDH7A1/LOC119713271/IVD/LOC101792573/ALDH8A1/ALDH4A1/GLUD1	14
MF	GO:0005509	calcium ion binding	13/250	265/6527	0.215426	0.965641	101790249/101794730/101802263/101792273/101795371/101800869/101791530/101793322/101803543/101789534/101800667/101789987/101794062	ANXA1/ANXA11/LPCAT2/ANXA2/LOC101795371/ANXA7/PLS3/THBS1/RASEF/CDHR5/CDH2/HMCN1/SUSD1	13

MF	GO:0004721	phosphoprotein phosphatase activity	4/250	63/6527	0.220905	0.965641	101802393/101803221/101792804/101791271	DUSP1/PTPN14/DUSP15/PTPN6	4
MF	GO:0019783	ubiquitin-like protein-specific protease activity	3/250	43/6527	0.226654	0.965641	101799480/101792575/101791234	UCHL1/USP44/USP49	3
MF	GO:0036459	thiol-dependent ubiquitinyl hydrolase activity	3/250	43/6527	0.226654	0.965641	101799480/101792575/101791234	UCHL1/USP44/USP49	3
MF	GO:0101005	ubiquitinyl hydrolase activity	3/250	43/6527	0.226654	0.965641	101799480/101792575/101791234	UCHL1/USP44/USP49	3
MF	GO:0016788	hydrolase activity, acting on ester bonds	10/250	201/6527	0.241174	0.965641	119715945/101802393/101803288/101803221/101804872/101792804/101799246/101791271/101796880/101791157	LOC119715945/DUSP1/LOC101803288/PTPN14/LOC101804872/DUSP15/PLCG2/PTPN6/PLCD3/TATDN3	10
MF	GO:0016887	ATPase activity	4/250	67/6527	0.254257	0.965641	119713166/101793897/119718656/101793641	LOC119713166/ATP6V1E1/LOC119718656/ATP	4
MF	GO:0008270	zinc ion binding	11/250	228/6527	0.257254	0.965641	101801981/101794528/101796941/101792575/101802147/101802225/101793204/101803240/119718292/119713599/101791234	ZFAND5/LOC101794528/TRIM71/USP44/L3MBTL3/TES/ZFAND1/FBXO30/LOC119718292/TRIM63/USP49	11
MF	GO:0140110	transcription regulator activity	10/250	205/6527	0.260063	0.965641	101805187/101801468/101790639/106019185/101801698/101794314/101797962/101802147/101796626/113845655	ATF3/CBFB/CREM/CEBPB/FOS/NFIL3/CREB5/L3MBTL3/JUN/JUND	10
MF	GO:0042578	phosphoric ester hydrolase activity	6/250	113/6527	0.264737	0.965641	101802393/101803221/101792804/101799246/101791271/101796880	DUSP1/PTPN14/DUSP15/PLCG2/PTPN6/PLCD3	6
MF	GO:0008238	exopeptidase activity	2/250	28/6527	0.291386	0.965641	101794820/101797577	LOC101794820/SCPEP1	2
MF	GO:0016791	phosphatase activity	4/250	72/6527	0.297239	0.965641	101802393/101803221/101792804/101791271	DUSP1/PTPN14/DUSP15/PTPN6	4
MF	GO:0016746	transferase activity, transferring acyl groups	3/250	50/6527	0.299744	0.965641	101797414/101802263/101801215	LOC101797414/LPCAT2/DGAT2	3
MF	GO:0003700	DNA-binding transcription factor activity	8/250	168/6527	0.314914	0.965641	101805187/101790639/106019185/101801698/101794314/101797962/101796626/113845655	ATF3/CREM/CEBPB/FOS/NFIL3/CREB5/JUN/JUND	8
MF	GO:0008138	protein tyrosine/serine/threonine phosphatase activity	2/250	30/6527	0.320018	0.965641	101802393/101792804	DUSP1/DUSP15	2
MF	GO:0001067	regulatory region nucleic acid binding	1/250	10/6527	0.323503	0.965641	101799633	IRF1	1
MF	GO:0003713	transcription coactivator activity	1/250	10/6527	0.323503	0.965641	101801468	CBFB	1
MF	GO:0008375	acetylglucosaminyltransferase activity	1/250	10/6527	0.323503	0.965641	101796875	GCNT1	1
MF	GO:0016891	endoribonuclease activity, producing 5'-phosphomonoesters	1/250	10/6527	0.323503	0.965641	119715945	LOC119715945	1
MF	GO:0019239	deaminase activity	1/250	10/6527	0.323503	0.965641	101803104	AMPD3	1
MF	GO:0019887	protein kinase regulator activity	1/250	10/6527	0.323503	0.965641	101791694	CDKN1B	1
MF	GO:0044212	transcription regulatory region DNA binding	1/250	10/6527	0.323503	0.965641	101799633	IRF1	1
MF	GO:0004725	protein tyrosine phosphatase activity	2/250	31/6527	0.334235	0.965641	101803221/101791271	PTPN14/PTPN6	2
MF	GO:0003779	actin binding	3/250	54/6527	0.342176	0.965641	101793729/101791895/101789868	TWF2/LOC101791895/PXK	3

MF	GO:0030234	enzyme regulator activity	5/250	101/6527	0.344701	0.965641	101792619/101794822/101796096/101793795/101791694	TIMP4/TFPI/LOC101796096/RANGAP1/CDKN1B	5
MF	GO:0005319	lipid transporter activity	1/250	11/6527	0.349454	0.965641	101802251	PITPNM1	1
MF	GO:0005540	hyaluronic acid binding	1/250	11/6527	0.349454	0.965641	101798168	TNFAIP6	1
MF	GO:0016651	oxidoreductase activity, acting on NAD(P)H	1/250	11/6527	0.349454	0.965641	101795743	NDUFAF2	1
MF	GO:0016684	oxidoreductase activity, acting on peroxide as acceptor	1/250	11/6527	0.349454	0.965641	101794631	LOC101794631	1
MF	GO:0016893	endonuclease activity, active with either ribo- or deoxyribonucleic acids and producing 5'-phosphomonoesters	1/250	11/6527	0.349454	0.965641	119715945	LOC119715945	1
MF	GO:0019207	kinase regulator activity	1/250	11/6527	0.349454	0.965641	101791694	CDKN1B	1
MF	GO:0050662	coenzyme binding	5/250	103/6527	0.36006	0.965641	101790158/101798440/101789745/101789907/101794024	HIBADH/LOC101798440/ME3/IVD/OAT	5
MF	GO:0016616	oxidoreductase activity, acting on the CH-OH group of donors, NAD or NADP as acceptor	2/250	33/6527	0.362393	0.965641	101798440/101789745	LOC101798440/ME3	2
MF	GO:0004298	threonine-type endopeptidase activity	1/250	12/6527	0.374414	0.965641	101803700	PSMB1	1
MF	GO:0004521	endoribonuclease activity	1/250	12/6527	0.374414	0.965641	119715945	LOC119715945	1
MF	GO:0016209	antioxidant activity	1/250	12/6527	0.374414	0.965641	101794631	LOC101794631	1
MF	GO:0070003	threonine-type peptidase activity	1/250	12/6527	0.374414	0.965641	101803700	PSMB1	1
MF	GO:0015399	primary active transmembrane transporter activity	2/250	34/6527	0.376308	0.965641	101793897/119718656	ATP6V1E1/LOC119718656	2
MF	GO:0015405	P-P-bond-hydrolysis-driven transmembrane transporter activity	2/250	34/6527	0.376308	0.965641	101793897/119718656	ATP6V1E1/LOC119718656	2
MF	GO:0042626	ATPase activity, coupled to transmembrane movement of substances	2/250	34/6527	0.376308	0.965641	101793897/119718656	ATP6V1E1/LOC119718656	2
MF	GO:0043492	ATPase activity, coupled to movement of substances	2/250	34/6527	0.376308	0.965641	101793897/119718656	ATP6V1E1/LOC119718656	2
MF	GO:0016758	transferase activity, transferring hexosyl groups	3/250	58/6527	0.384417	0.965641	101803248/101793948/101796875	B3GALNT2/B3GNT2/GCNT1	3
MF	GO:0016462	pyrophosphatase activity	15/250	356/6527	0.388981	0.965641	119714718/101798141/106014280/119713166/101793897/119714941/101803472/101803543/101803768/119718656/119718018/101793641/101803065/101790303/101804925	LOC119714718/RRAD/PPA2/LOC119713166/ATP6V1E1/LOC119714941/ITPA/RASEF/KIF16B/LOC119718656/LOC119718018/ATP13A2/RASL11A/RAB39A/RAB9A	15
MF	GO:0015078	proton transmembrane transporter activity	2/250	35/6527	0.390097	0.965641	101791662/101793897	SLC9A6/ATP6V1E1	2
MF	GO:0016614	oxidoreductase activity, acting on CH-OH group of donors	2/250	35/6527	0.390097	0.965641	101798440/101789745	LOC101798440/ME3	2
MF	GO:0004222	metalloendopeptidase activity	3/250	59/6527	0.394893	0.965641	101794528/101794584/101790574	LOC101794528/ADAMTS17/ADAMTS5	3

MF	GO:0016818	hydrolase activity, acting on acid anhydrides, in phosphorus-containing anhydrides	15/250	358/6527	0.397453	0.965641	119714718/101798141/106014280/119713166/101793897/119714941/101803472/101803543/101803768/119718656/119718018/101793641/101803065/101790303/101804925	LOC119714718/RRAD/PPA2/LOC119713166/ATP6V1E1/LOC119714941/ITPA/RASEF/KIF16B/LOC119718656/LOC119718018/ATP13A2/RASL11A/RAB39A/RAB9A	15
MF	GO:0003743	translation initiation factor activity	1/250	13/6527	0.39842	0.965641	101793553	EIF1	1
MF	GO:0022804	active transmembrane transporter activity	3/250	60/6527	0.405323	0.965641	101791662/101793897/119718656	SLC9A6/ATP6V1E1/LOC119718656	3
MF	GO:0003924	GTPase activity	7/250	159/6527	0.408527	0.965641	119714718/101798141/101803543/119718018/101803065/101790303/101804925	LOC119714718/RRAD/RASEF/LOC119718018/RASL11A/RAB39A/RAB9A	7
MF	GO:0016817	hydrolase activity, acting on acid anhydrides	15/250	362/6527	0.414447	0.965641	119714718/101798141/106014280/119713166/101793897/119714941/101803472/101803543/101803768/119718656/119718018/101793641/101803065/101790303/101804925	LOC119714718/RRAD/PPA2/LOC119713166/ATP6V1E1/LOC119714941/ITPA/RASEF/KIF16B/LOC119718656/LOC119718018/ATP13A2/RASL11A/RAB39A/RAB9A	15
MF	GO:0008081	phosphoric diester hydrolase activity	2/250	37/6527	0.417255	0.965641	101799246/101796880	PLCG2/PLCD3	2
MF	GO:0003690	double-stranded DNA binding	1/250	14/6527	0.421508	0.965641	101802532	MSH2	1
MF	GO:0008092	cytoskeletal protein binding	6/250	136/6527	0.421698	0.965641	101793729/101791895/119714941/101803768/101797044/101789868	TWF2/LOC101791895/LOC119714941/KIF16B/LMOD2/PXK	6
MF	GO:0005539	glycosaminoglycan binding	1/250	15/6527	0.443713	0.965641	101798168	TNFAIP6	1
MF	GO:0019199	transmembrane receptor protein kinase activity	1/250	15/6527	0.443713	0.965641	101801641	LOC101801641	1
MF	GO:0031406	carboxylic acid binding	1/250	15/6527	0.443713	0.965641	101798168	TNFAIP6	1
MF	GO:0043177	organic acid binding	1/250	15/6527	0.443713	0.965641	101798168	TNFAIP6	1
MF	GO:0004553	hydrolase activity, hydrolyzing O-glycosyl compounds	2/250	40/6527	0.456809	0.965641	101792144/101800183	FUCA2/HYAL2	2
MF	GO:0004867	serine-type endopeptidase inhibitor activity	1/250	16/6527	0.465069	0.965641	101794822	TFPI	1
MF	GO:0051082	unfolded protein binding	1/250	16/6527	0.465069	0.965641	101801226	DNAJA4	1
MF	GO:0046914	transition metal ion binding	12/250	300/6527	0.481513	0.965641	101801981/101794528/101796941/101792575/101802147/101802225/101793204/101792573/101803240/119718292/119713599/101791234	ZFAND5/LOC101794528/TRIM71/USP44/L3MBTL3/TES/ZFAND1/LOC101792573/FBXO30/LOC119718292/TRIM63/USP49	12
MF	GO:0004180	carboxypeptidase activity	1/250	17/6527	0.485609	0.965641	101797577	SCPEP1	1
MF	GO:0004519	endonuclease activity	1/250	17/6527	0.485609	0.965641	119715945	LOC119715945	1
MF	GO:0016874	ligase activity	3/250	69/6527	0.496213	0.965641	101799450/101797455/101796914	GLUL/ASNS/LIG1	3
MF	GO:0004175	endopeptidase activity	6/250	148/6527	0.503206	0.965641	101794528/101794584/101804552/101794268/101803700/101790574	LOC101794528/ADAMTS17/PLAU/CTSB/PSMB1/ADAMTS5	6
MF	GO:0016829	lyase activity	2/250	44/6527	0.507058	0.965641	101799385/101800658	CENPV/PCBD1	2

MF	GO:0005102	signaling receptor binding	6/250	149/6527	0.509836	0.965641	101803050/101803777/101799824/101792356/101799859/101796903	CRH/TNFSF13B/WNT2/WNT5B/RAPSN/LOC101796903	6
MF	GO:0042623	ATPase activity, coupled	2/250	46/6527	0.531029	0.965641	101793897/119718656	ATP6V1E1/LOC1197186	2
MF	GO:0008236	serine-type peptidase activity	3/250	73/6527	0.534362	0.965641	101804552/101791397/101797577	PLAU/DPP7/SCPEP1	3
MF	GO:0017171	serine hydrolase activity	3/250	73/6527	0.534362	0.965641	101804552/101791397/101797577	PLAU/DPP7/SCPEP1	3
MF	GO:0000287	magnesium ion binding	1/250	20/6527	0.542632	0.965641	106014280	PPA2	1
MF	GO:0008135	translation factor activity, RNA binding	1/250	20/6527	0.542632	0.965641	101793553	EIF1	1
MF	GO:0004540	ribonuclease activity	1/250	21/6527	0.560204	0.965641	119715945	LOC119715945	1
MF	GO:0009055	electron transfer activity	1/250	21/6527	0.560204	0.965641	101795743	NDUFAF2	1
MF	GO:0016798	hydrolase activity, acting on glycosyl bonds	2/250	49/6527	0.565475	0.965641	101792144/101800183	FUCA2/HYAL2	2
MF	GO:0048037	cofactor binding	7/250	185/6527	0.568407	0.965641	101790158/101794631/101798440/101789745/101789907/101792573/101794024	HIBADH/LOC101794631/LOC101798440/ME3/IVD/LOC101792573/OAT	7
MF	GO:0005126	cytokine receptor binding	1/250	22/6527	0.577104	0.965641	101803777	TNFSF13B	1
MF	GO:0016747	transferase activity, transferring acyl groups other than amino-acyl groups	1/250	22/6527	0.577104	0.965641	101801215	DGAT2	1
MF	GO:0017111	nucleoside-triphosphatase activity	13/250	350/6527	0.587753	0.965641	119714718/101798141/119713166/101793897/119714941/101803543/101803768/119718656/119718018/101793641/101803065/101790303/101804925	LOC119714718/RRAD/LOC119713166/ATP6V1E1/LOC119714941/RASEF/KIF16B/LOC119718656/LOC119718018/ATP13A2/RASL11A/RAB39A/RAB9A	13
MF	GO:0008194	UDP-glycosyltransferase activity	1/250	23/6527	0.593357	0.965641	101796875	GCNT1	1
MF	GO:0016705	oxidoreductase activity, acting on paired donors, with incorporation or reduction of molecular oxygen	2/250	52/6527	0.598071	0.965641	101805057/101792573	HMOX1/LOC101792573	2
MF	GO:0005044	scavenger receptor activity	1/250	24/6527	0.608987	0.965641	119716554	SCARA5	1
MF	GO:0038024	cargo receptor activity	1/250	24/6527	0.608987	0.965641	119716554	SCARA5	1
MF	GO:0005096	GTPase activator activity	1/250	25/6527	0.624019	0.965641	101793795	RANGAP1	1
MF	GO:0016627	oxidoreductase activity, acting on the CH-CH group of donors	1/250	25/6527	0.624019	0.965641	101789907	IVD	1
MF	GO:0003777	microtubule motor activity	2/250	55/6527	0.628803	0.965641	119714941/101803768	LOC119714941/KIF16B	2
MF	GO:0008237	metallopeptidase activity	3/250	84/6527	0.630262	0.965641	101794528/101794584/101790574	LOC101794528/ADAMTS17/ADAMTS5	3
MF	GO:0015291	secondary active transmembrane transporter activity	1/250	26/6527	0.638475	0.965641	101791662	SLC9A6	1
MF	GO:0020037	heme binding	2/250	56/6527	0.638635	0.965641	101794631/101792573	LOC101794631/LOC101792573	2
MF	GO:0016763	transferase activity, transferring pentosyl groups	1/250	27/6527	0.652378	0.965641	119716831	LOC119716831	1
MF	GO:0030170	pyridoxal phosphate binding	1/250	27/6527	0.652378	0.965641	101794024	OAT	1
MF	GO:0070279	vitamin B6 binding	1/250	27/6527	0.652378	0.965641	101794024	OAT	1
MF	GO:0046906	tetrapyrrole binding	2/250	58/6527	0.657685	0.965641	101794631/101792573	LOC101794631/LOC101792573	2
MF	GO:0016757	transferase activity, transferring glycosyl groups	4/250	119/6527	0.674781	0.965641	101803248/101793948/101796875/119716831	B3GALNT2/B3GNT2/GCNT1/LOC119716831	4
MF	GO:0008017	microtubule binding	2/250	62/6527	0.693371	0.965641	119714941/101803768	LOC119714941/KIF16B	2

MF	GO:0005179	hormone activity	1/250	31/6527	0.702862	0.965641	101803050	CRH	1
MF	GO:0000988	transcription factor activity, protein binding	1/250	32/6527	0.714298	0.965641	101801468	CBFB	1
MF	GO:0000989	transcription factor activity, transcription factor binding	1/250	32/6527	0.714298	0.965641	101801468	CBFB	1
MF	GO:0003712	transcription cofactor activity	1/250	32/6527	0.714298	0.965641	101801468	CBFB	1
MF	GO:0030246	carbohydrate binding	1/250	32/6527	0.714298	0.965641	101791338	EVA1C	1
MF	GO:0030695	GTPase regulator activity	1/250	33/6527	0.725295	0.965641	101793795	RANGAP1	1
MF	GO:0004842	ubiquitin-protein transferase activity	1/250	34/6527	0.73587	0.965641	101795149	LOC101795149	1
MF	GO:0008047	enzyme activator activity	1/250	34/6527	0.73587	0.965641	101793795	RANGAP1	1
MF	GO:0019787	ubiquitin-like protein transferase activity	1/250	34/6527	0.73587	0.965641	101795149	LOC101795149	1
MF	GO:0019842	vitamin binding	1/250	34/6527	0.73587	0.965641	101794024	OAT	1
MF	GO:0140097	catalytic activity, acting on DNA	1/250	34/6527	0.73587	0.965641	101796914	LIG1	1
MF	GO:0050660	flavin adenine dinucleotide binding	1/250	35/6527	0.74604	0.965641	101789907	IVD	1
MF	GO:0022890	inorganic cation transmembrane transporter activity	3/250	104/6527	0.767494	0.965641	101791662/101804595/101793897	SLC9A6/SLC39A13/ATP6V1E1	3
MF	GO:0015631	tubulin binding	2/250	72/6527	0.769293	0.965641	119714941/101803768	LOC119714941/KIF16B	2
MF	GO:0005230	extracellular ligand-gated ion channel activity	1/250	38/6527	0.774268	0.965641	101800466	CHRNA10	1
MF	GO:0060589	nucleoside-triphosphatase regulator activity	1/250	38/6527	0.774268	0.965641	101793795	RANGAP1	1
MF	GO:0001883	purine nucleoside binding	6/250	198/6527	0.777166	0.965641	101798141/101803543/119718018/101803065/101790303/101804925	RRAD/RASEF/LOC119718018/RASL11A/RAB39A/RAB9A	6
MF	GO:0005525	GTP binding	6/250	198/6527	0.777166	0.965641	101798141/101803543/119718018/101803065/101790303/101804925	RRAD/RASEF/LOC119718018/RASL11A/RAB39A/RAB9A	6
MF	GO:0032549	ribonucleoside binding	6/250	198/6527	0.777166	0.965641	101798141/101803543/119718018/101803065/101790303/101804925	RRAD/RASEF/LOC119718018/RASL11A/RAB39A/RAB9A	6
MF	GO:0032550	purine ribonucleoside binding	6/250	198/6527	0.777166	0.965641	101798141/101803543/119718018/101803065/101790303/101804925	RRAD/RASEF/LOC119718018/RASL11A/RAB39A/RAB9A	6
MF	GO:0032561	guanyl ribonucleotide binding	6/250	198/6527	0.777166	0.965641	101798141/101803543/119718018/101803065/101790303/101804925	RRAD/RASEF/LOC119718018/RASL11A/RAB39A/RAB9A	6
MF	GO:0001882	nucleoside binding	6/250	199/6527	0.781247	0.965641	101798141/101803543/119718018/101803065/101790303/101804925	RRAD/RASEF/LOC119718018/RASL11A/RAB39A/RAB9A	6
MF	GO:0004518	nuclease activity	1/250	39/6527	0.782965	0.965641	119715945	LOC119715945	1
MF	GO:0015077	monovalent inorganic cation transmembrane transporter activity	2/250	75/6527	0.788652	0.965641	101791662/101793897	SLC9A6/ATP6V1E1	2
MF	GO:0015276	ligand-gated ion channel activity	1/250	40/6527	0.791328	0.965641	101800466	CHRNA10	1
MF	GO:0022834	ligand-gated channel activity	1/250	40/6527	0.791328	0.965641	101800466	CHRNA10	1
MF	GO:0019001	guanyl nucleotide binding	6/250	209/6527	0.819004	0.965641	101798141/101803543/119718018/101803065/101790303/101804925	RRAD/RASEF/LOC119718018/RASL11A/RAB39A/RAB9A	6
MF	GO:0008324	cation transmembrane transporter activity	3/250	116/6527	0.827925	0.965641	101791662/101804595/101793897	SLC9A6/SLC39A13/ATP6V1E1	3
MF	GO:0005088	Ras guanyl-nucleotide exchange factor activity	1/250	45/6527	0.828566	0.965641	101795713	KALRN	1
MF	GO:0005089	Rho guanyl-nucleotide exchange factor activity	1/250	45/6527	0.828566	0.965641	101795713	KALRN	1

MF	GO:0098772	molecular function regulator	8/250	275/6527	0.834956	0.965641	101792619/101794822/101803050/101796096/101795713/101793795/101791694/101796903	TIMP4/TFPI/CRH/LOC101796096/KALRN/RANGAP1/CDKN1B/LOC101796903	8
MF	GO:0005506	iron ion binding	1/250	46/6527	0.835178	0.965641	101792573	LOC101792573	1
MF	GO:0015318	inorganic molecular entity transmembrane transporter activity	6/250	215/6527	0.839087	0.965641	101791662/101804595/101800466/101793897/101795086/119713969	SLC9A6/SLC39A13/CHRNA10/ATP6V1E1/KCNQ4/KCNC4	6
MF	GO:0004888	transmembrane signaling receptor activity	7/250	252/6527	0.856579	0.965641	101789648/101791406/101802610/101790005/101794938/101801641/101801041	GPR157/CD247/CCR9/LOC101790005/ADGRD1/LOC101801641/VIPR2	7
MF	GO:0017048	Rho GTPase binding	1/250	51/6527	0.864616	0.965641	101795713	KALRN	1
MF	GO:0140101	catalytic activity, acting on a tRNA	1/250	51/6527	0.864616	0.965641	101803288	LOC101803288	1
MF	GO:0008083	growth factor activity	1/250	54/6527	0.8797	0.965641	101796903	LOC101796903	1
MF	GO:0038023	signaling receptor activity	7/250	262/6527	0.881943	0.965641	101789648/101791406/101802610/101790005/101794938/101801641/101801041	GPR157/CD247/CCR9/LOC101790005/ADGRD1/LOC101801641/VIPR2	7
MF	GO:0060089	molecular transducer activity	7/250	262/6527	0.881943	0.965641	101789648/101791406/101802610/101790005/101794938/101801641/101801041	GPR157/CD247/CCR9/LOC101790005/ADGRD1/LOC101801641/VIPR2	7
MF	GO:0004871	signal transducer activity	7/250	266/6527	0.890993	0.965641	101789648/101791406/101802610/101790005/101794938/101801641/101801041	GPR157/CD247/CCR9/LOC101790005/ADGRD1/LOC101801641/VIPR2	7
MF	GO:0140098	catalytic activity, acting on RNA	2/250	99/6527	0.898312	0.965641	119715945/101803288	LOC119715945/LOC101803288	2
MF	GO:0015075	ion transmembrane transporter activity	6/250	238/6527	0.900041	0.965641	101791662/101804595/101800466/101793897/101795086/119713969	SLC9A6/SLC39A13/CHRNA10/ATP6V1E1/KCNQ4/KCNC4	6
MF	GO:0005216	ion channel activity	3/250	137/6527	0.901664	0.965641	101800466/101795086/119713969	CHRNA10/KCNQ4/KCNC4	3
MF	GO:0022838	substrate-specific channel activity	3/250	137/6527	0.901664	0.965641	101800466/101795086/119713969	CHRNA10/KCNQ4/KCNC4	3
MF	GO:0004930	G-protein-coupled receptor activity	5/250	206/6527	0.902119	0.965641	101789648/101802610/101790005/101794938/101801041	GPR157/CCR9/LOC101790005/ADGRD1/VIPR2	5
MF	GO:0004252	serine-type endopeptidase activity	1/250	60/6527	0.905029	0.965641	101804552	PLAU	1
MF	GO:0017016	Ras GTPase binding	1/250	60/6527	0.905029	0.965641	101795713	KALRN	1
MF	GO:0031267	small GTPase binding	1/250	60/6527	0.905029	0.965641	101795713	KALRN	1
MF	GO:0030545	receptor regulator activity	2/250	103/6527	0.910383	0.965641	101803050/101796903	CRH/LOC101796903	2
MF	GO:0048018	receptor ligand activity	2/250	103/6527	0.910383	0.965641	101803050/101796903	CRH/LOC101796903	2
MF	GO:0008168	methyltransferase activity	1/250	62/6527	0.91223	0.965641	101797486	METTL7A	1
MF	GO:0003774	motor activity	2/250	105/6527	0.915905	0.965641	119714941/101803768	LOC119714941/KIF16B	2
MF	GO:0022836	gated channel activity	1/250	66/6527	0.925042	0.965641	101800466	CHRNA10	1
MF	GO:0022839	ion gated channel activity	1/250	66/6527	0.925042	0.965641	101800466	CHRNA10	1
MF	GO:0046873	metal ion transmembrane transporter activity	1/250	67/6527	0.927942	0.965641	101804595	SLC39A13	1
MF	GO:0015267	channel activity	3/250	149/6527	0.929697	0.965641	101800466/101795086/119713969	CHRNA10/KCNQ4/KCNC4	3

MF	GO:0022803	passive transmembrane transporter activity	3/250	149/6527	0.929697	0.965641	101800466/101795086/119713969	CHRNA10/KCNQ4/KCNC4	3
MF	GO:0016741	transferase activity, transferring one-carbon groups	1/250	68/6527	0.930731	0.965641	101797486	METTL7A	1
MF	GO:0005085	guanyl-nucleotide exchange factor activity	1/250	72/6527	0.940851	0.965641	101795713	KALRN	1
MF	GO:0005215	transporter activity	9/250	364/6527	0.944753	0.965641	101802251/101791662/101804595/101800466/101793897/119718656/101804200/101795086/119713969	PITPNM1/SLC9A6/SLC39A13/CHRNA10/ATP6V1E1/LOC119718656/SLC15A4/KCNQ4/KCNC4	9
MF	GO:0022857	transmembrane transporter activity	8/250	333/6527	0.946632	0.965641	101791662/101804595/101800466/101793897/119718656/101804200/101795086/119713969	SLC9A6/SLC39A13/CHRNA10/ATP6V1E1/LOC119718656/SLC15A4/KCNQ4/KCNC4	8
MF	GO:0046983	protein dimerization activity	3/250	161/6527	0.950232	0.965641	101801755/101789522/101790556	MYF5/MYOD1/TFAP4	3
MF	GO:0051020	GTPase binding	1/250	87/6527	0.967315	0.977716	101795713	KALRN	1
MF	GO:0019899	enzyme binding	1/250	101/6527	0.981234	0.982876	101795713	KALRN	1
MF	GO:0003723	RNA binding	2/250	153/6527	0.982876	0.982876	101793553/113841084	EIF1/KHDC4	2
