# Supplementary material for: Identification of relevant differential genes to the divergent development of pectoral muscle in ducks by transcriptomic analysis
Source: Anim Biosci. 2024 Apr 1;37(8):1345–54. doi: 10.5713/ab.23.0505 (PMC11222850; doi:10.5713/ab.23.0505)
Supplement: Supplementary file 4 [file ab-23-0505-Supplementary-Table-4.pdf]

Supplemental Table 4：All Kyoto Encyclopedia of Genes and Genomes (KEGG) pathways enrichment

KEGGID	Description	GeneRatio	BgRatio	pvalue	padj	geneID	geneName	keggID	Count	Up	Up_	Down_Gene_id
apla04512	ECM-receptor interaction	22/452	88/4924	8.87E-06	0.001277	101802858/101800267/101798679/101799524/119714715/101800985/101799662/101793322/101799485/101794144/101791187/101803666/101796164/101801687/101796495/101800228/101794275/101794158/113845089/101796802/101791104/101801954	FREM2/FRAS1/FREM1/AGRN/ITGA7/LAMA4/COL4A5/THBS1/ITGA4/SDC4/LAMB2/ITGB3/ITGA8/LAMB4/CD36/COL4A6/LOC101794275/COL6A3/LOC113845089/CHAD/ITGA6/LAMA5	apla:101802858/apla:101800267/apla:101798679/apla:101799524/apla:101791104/apla:101800985/apla:101799662/apla:101793322/apla:101799485/apla:101794144/apla:101791187/apla:101803666/apla:101796164/apla:101801687/apla:101796495/apla:101803874/apla:101794275/apla:101794158/apla:113845089/apla:101796802/apla:101791104/apla:101801954	22	17	101	101793322/101799485/101794144/101803666/101794275
apla00071	Fatty acid degradation	11/452	33/4924	0.000103	0.007427	101799889/113839590/101804289/101798003/101793275/119713271/119714639/101791052/101803526/101798039/101795358	ACSBG2/LOC113839590/CPT2/ACSL4/ALDH7A1/LOC119713271/ACADM/ACAA2/ECI1/ACSL1/ACSBG1	apla:101799889/apla:113839590/apla:101804289/apla:101798003/apla:101793275/apla:101792386/apla:101801937/apla:101791052/apla:101803526/apla:101798039/apla:101795358	11	5	101	101799889/113839590/101798003/101793275/119713271/101795358
apla00620	Pyruvate metabolism	11/452	35/4924	0.000188	0.009009	101803853/113839590/101801152/101789492/101803542/101799210/101789745/101793275/119713271/101790726/101804610	ME1/LOC113839590/LDHB/FH/ACSS2/LOC101799210/ME3/ALDH7A1/LOC119713271/PKM/MDH2	apla:101803853/apla:113839590/apla:101801152/apla:101789492/apla:101803542/apla:101799210/apla:101789745/apla:101793275/apla:101792386/apla:101790726/apla:101804610	11	7	101	113839590/101789745/101793275/119713271
apla03320	PPAR signaling pathway	16/452	67/4924	0.000266	0.009584	101803853/101796218/101799889/101805391/101804289/101798003/101789745/101797549/101804343/119713836/101796495/101789572/101798039/101801349/101795358/101790208	ME1/LPL/ACSBG2/SLC27A1/CPT2/ACSL4/ME3/ANGPTL4/ACOX2/SLC27A3/CD36/PPARA/ACSL1/SCD/ACSBG1/SCD5	apla:101803853/apla:101796218/apla:101799889/apla:101805391/apla:101804289/apla:101798003/apla:101789745/apla:101797549/apla:101804343/apla:101799927/apla:101796495/apla:101789572/apla:101798039/apla:101801349/apla:101795358/apla:101790208	16	9	101	101796218/101799889/101805391/101798003/101789745/101801349/101795358
apla00010	Glycolysis / Gluconeogenesis	12/452	47/4924	0.000816	0.023512	113839590/101803151/101801152/101803542/101799210/101793275/119713271/101790428/101790726/119713467/101790798/101797601	LOC113839590/BPGM/LDHB/ACSS2/LOC101799210/ALDH7A1/LOC119713271/PGM1/PKM/HK2/TPI1/GPI	apla:113839590/apla:101803151/apla:101801152/apla:101803542/apla:101799210/apla:101793275/apla:101792386/apla:101790428/apla:101790726/apla:101794107/apla:101790798/apla:101797601	12	8	101	113839590/101793275/119713271/119713467
apla00910	Nitrogen metabolism	6/452	17/4924	0.002973	0.063426	101795734/101796737/101799450/101801612/101797377/101789643	CA12/CA8/GLUL/LOC101801612/GLUD1/CA4	apla:101795734/apla:101796737/apla:101799450/apla:101801612/apla:101797377/apla:101789643	6	3	101	101799450/101797377/101789643
apla04260	Cardiac muscle contraction	13/452	62/4924	0.003472	0.063426	101805261/106016351/119714808/101802358/101791662/101796232/5405810/113845023/101798929/101801311/101803316/101793350/5405813	ATP2A2/TRDN/ATP1B2/CACNG4/SLC9A6/LOC101796232/COX1/CACNA2D2/SLC8A3/LOC101801311/LOC101803316/CACNA2D1/CYTB	apla:101805261/apla:101795831/apla:101795008/apla:101802358/apla:101791662/apla:101796232/apla:5405810/apla:101793350/apla:101798929/apla:101801311/apla:101803316/apla:101793350/apla:5405813	13	11	101	101791662/101803316
apla00640	Propanoate metabolism	8/452	29/4924	0.003593	0.063426	101801152/101803542/101799210/119714639/101800289/101790102/101798817/101799937	LDHB/ACSS2/LOC101799210/ACADM/SUCLG2/BCKDHB/ACSS3/SUCLG1	apla:101801152/apla:101803542/apla:101799210/apla:101801937/apla:101800289/apla:101790102/apla:101798817/apla:101799937	8	7	101	101790102
apla00561	Glycerolipid metabolism	13/452	63/4924	0.00402	0.063426	101797414/101795185/101796218/113839590/101801215/119713935/101793275/119713271/101803103/101796497/101799752/101801143/101800297	LOC101797414/LPIN1/LPL/LOC113839590/DGAT2/PNPLA1/ALDH7A1/LOC119713271/GPAM/PNPLA2/LIPG/DGKI/MBOAT2	apla:101797414/apla:101795185/apla:101796218/apla:113839590/apla:101801215/apla:101796497/apla:101793275/apla:101792386/apla:101803103/apla:101796497/apla:101799752/apla:101801143/apla:101800297	13	3	119	101797414/101795185/101796218/113839590/101801215/101793275/119713271/101796497/101799752/101800297
apla04510	Focal adhesion	30/452	200/4924	0.004405	0.063426	119714715/101795371/101800985/101796600/101799662/101793322/101799485/101792227/101791187/101795002/101803666/101796164/101793664/101801687/101800228/101794275/119715213/101794158/101802656/113845089/101793597/101796626/101796802/101791104/101795633/101800900/101801954/101803502/101791037/101797327	ITGA7/LOC101795371/LAMA4/PAK3/COL4A5/THBS1/ITGA4/CAV3/LAMB2/EGF/ITGB3/ITGA8/HGF/LAMB4/COL4A6/LOC101794275/LOC119715213/COL6A3/VAV2/LOC113845089/IGF1/JUN/CHAD/ITGA6/RAF1/PIK3CB/LAMA5/PDGFA/MAP2K1/LOC101797327	apla:101791104/apla:101795371/apla:101800985/apla:101796600/apla:101799662/apla:101793322/apla:101799485/apla:101792227/apla:101791187/apla:101795002/apla:101803666/apla:101796164/apla:101793664/apla:101801687/apla:101803874/apla:101794275/apla:101796074/apla:101794158/apla:101802656/apla:113845089/apla:101793597/apla:101796626/apla:101796802/apla:101791104/apla:101795633/apla:101800900/apla:101801954/apla:101803502/apla:101791037/apla:101797327	30	19	119	101795371/101793322/101799485/101803666/101793664/101794275/119715213/101796626/101795633/101800900/101791037
apla04920	Adipocytokine signaling pathway	12/452	61/4924	0.00835	0.109308	101799889/101789439/101798003/101804354/101799561/101796495/101801086/101789572/101804723/101798039/101795358/101791200	ACSBG2/LOC101789439/ACSL4/PPARGC1A/PRKAA2/CD36/ADIPOR1/PPARA/IRS2/ACSL1/ACSBG1/SOCS3	apla:101799889/apla:101789439/apla:101798003/apla:101804354/apla:101799561/apla:101796495/apla:101801086/apla:101789572/apla:101804723/apla:101798039/apla:101795358/apla:101791200	12	6	101	101799889/101798003/101801086/101804723/101795358/101791200

apla00330	Arginine and proline metabolism	9/452	43/4924	0.014264	0.171163	101789528/101795318/113839590/novel.282/101793275/119713271/101794024/101790132/101791774	LOC101789528/SAT1/LOC113839590/-/ALDH7A1/LOC119713271/OAT/ODC1/ALDH4A1	apla:101789528/apla:101795318/apla:113839590/apla:101804818/apla:101793275/apla:101792386/apla:101794024/apla:101790132/apla:101791774	9	0		101789528/101795318/113839590/novel.282/101793275/119713271/101794024/101790132/101791774
apla00250	Alanine, aspartate and glutamate metabolism	8/452	37/4924	0.016869	0.186859	101801928/101799450/101793939/101797455/101793941/101791774/101797377/101801878	ADSS1/GLUL/ALDH5A1/ASNS/NAT8L/ALDH4A1/GLUD1/ADSL	apla:101801928/apla:101799450/apla:101793939/apla:101797455/apla:101793941/apla:101791774/apla:101797377/apla:101801878	8	4	101	101799450/101797455/101791774/101797377
apla00280	Valine, leucine and isoleucine degradation	9/452	45/4924	0.019035	0.191572	101790158/113839590/101800887/101793275/119713271/119714639/101791052/101789907/101790102	HIBADH/LOC113839590/LOC101800887/ALDH7A1/LOC119713271/ACADM/ACAA2/IVD/BCKDHB	apla:101790158/apla:113839590/apla:101800887/apla:101793275/apla:101792386/apla:101801937/apla:101791052/apla:101789907/apla:101790102	9	3	101	101790158/113839590/101793275/119713271/101789907/101790102
apla00770	Pantothenate and CoA biosynthesis	5/452	18/4924	0.019955	0.191572	101798008/113839590/119713271/101798651/101800542	GADL1/LOC113839590/LOC119713271/LOC101798651/ENPP3	apla:101798008/apla:113839590/apla:101792386/apla:101798651/apla:101800542	5	2	101	113839590/119713271/101798651
apla01200	Carbon metabolism	15/452	98/4924	0.03238	0.291418	101803853/101789492/101803542/101789745/119714639/101800289/101790726/101804610/101799937/119713467/101802992/101790798/101794860/101797377/101797601	ME1/FH/ACSS2/ME3/ACADM/SUCLG2/PKM/MDH2/SUCLG1/HK2/LOC101802992/TPI1/PSAT1/GLUD1/GPI	apla:101803853/apla:101789492/apla:101803542/apla:101789745/apla:101801937/apla:101800289/apla:101790726/apla:101804610/apla:101799937/apla:101794107/apla:101802992/apla:101790798/apla:101794860/apla:101797377/apla:101797601	15	11	101	101789745/119713467/101794860/101797377
apla04012	ErbB signaling pathway	12/452	76/4924	0.042577	0.357696	101798286/101796600/101795002/101791354/101799246/101796626/101795633/101800900/101801648/101791694/101801144/101791037	EIF4EBP1/PAK3/EGF/NRG1/PLCG2/JUN/RAF1/PIK3CB/CAMK2D/CDKN1B/ERBB4/MAP2K1	apla:101798286/apla:101796600/apla:101795002/apla:101791354/apla:101799246/apla:101796626/apla:101795633/apla:101800900/apla:101801648/apla:101791694/apla:101801144/apla:101791037	12	5	101	101798286/101799246/101796626/101795633/101800900/101791694/101791037
apla04625	C-type lectin receptor signaling pathway	13/452	85/4924	0.044712	0.357696	101789439/101799633/101794933/101789899/101799246/101796626/101795633/101800900/101804036/101791236/101793659/101802364/110353775	LOC101789439/IRF1/LOC101794933/LOC101789899/PLCG2/JUN/RAF1/PIK3CB/MALT1/MAP3K14/LSP1/MRAS/LOC110353775	apla:101789439/apla:101799633/apla:101794933/apla:101789899/apla:101799246/apla:101796626/apla:101795633/apla:101800900/apla:101804036/apla:101791236/apla:101793659/apla:101802364/apla:101796703	13	2	101	101799633/101794933/101789899/101799246/101796626/101795633/101800900/101804036/101791236/101793659/110353775
apla04514	Cell adhesion molecules	17/452	121/4924	0.048909	0.369289	101802722/101799604/119714715/101797730/101799485/101794144/101804352/101799421/101796164/101796942/101800673/101794066/101791104/101803609/101800667/101796803/101791967	CDH4/ALCAM/ITGA7/CADM1/ITGA4/SDC4/PTPRD/LOC101799421/ITGA8/CNTN1/NRXN1/IGSF11/ITGA6/PTPRM/CDH2/ITGB2/CNTNAP1	apla:101802722/apla:101799604/apla:101791104/apla:101797730/apla:101799485/apla:101794144/apla:101804352/apla:101799421/apla:101796164/apla:101796942/apla:101800673/apla:101794066/apla:101791104/apla:101803609/apla:101800667/apla:101796803/apla:101791967	17	13	101	101799485/101794144/101800667/101796803
apla04010	MAPK signaling pathway	33/452	270/4924	0.05129	0.369289	110354206/101803371/101802393/101802390/101797658/101801698/101803181/113845585/101795002/101802358/101800942/119715240/101793664/119713089/119715213/101793597/101796626/113845023/101804166/101795633/101799111/101801311/101801641/101791769/101791236/101801144/101792182/101803502/101791037/101802364/113845655/101793350/101797327	FGFR4/MAP2K3/DUSP1/DDIT3/HSPA2/FOS/MAP3K6/HSPB1/EGF/CACNG4/RPS6KA1/LOC119715240/HGF/HSPA5/LOC119715213/IGF1/JUN/CACNA2D2/DUSP3/RAF1/IGF2/LOC101801311/LOC101801641/MAP3K8/MAP3K14/ERBB4/NRK/PDGFA/MAP2K1/MRAS/JUND/CACNA2D1/LOC101797327	apla:110354206/apla:101803371/apla:101802393/apla:101802390/apla:101797658/apla:101801698/apla:101803181/apla:113845585/apla:101795002/apla:101802358/apla:101800942/apla:113841088/apla:101793664/apla:101801738/apla:101796074/apla:101793597/apla:101796626/apla:101793350/apla:101804166/apla:101795633/apla:101799111/apla:101801311/apla:101801641/apla:101791769/apla:101791236/apla:101801144/apla:101792182/apla:101803502/apla:101791037/apla:101802364/apla:113845655/apla:101793350/apla:101797327	33	14	110	101803371/101802393/101802390/101797658/101801698/101803181/113845585/101800942/119715240/101793664/119713089/119715213/101796626/101795633/101801641/101791769/101791236/101791037/113845655
apla00500	Starch and sucrose metabolism	6/452	31/4924	0.059181	0.397737	101798581/101790428/113845291/119713467/101797601/101800542	PGM2L1/PGM1/LOC113845291/HK2/GPI/ENPP3	apla:101798581/apla:101790428/apla:113845291/apla:101794107/apla:101797601/apla:101800542	6	5	101	119713467
apla01212	Fatty acid metabolism	9/452	55/4924	0.060765	0.397737	101799889/101804289/101798003/119714639/101791052/101798039/101801349/101795358/101790208	ACSBG2/CPT2/ACSL4/ACADM/ACAA2/ACSL1/SCD/ACSBG1/SCD5	apla:101799889/apla:101804289/apla:101798003/apla:101801937/apla:101791052/apla:101798039/apla:101801349/apla:101795358/apla:101790208	9	5	101	101799889/101798003/101801349/101795358
apla00190	Oxidative phosphorylation	16/452	118/4924	0.071487	0.40861	119714869/119714946/5405818/101805119/5405821/106014280/101802167/101793897/101795476/101796232/5405810/119714948/5405811/5405820/5405813/101790402	LOC119714869/LOC119714946/ATP8/NDUFS1/ND2/PPA2/ATP6V0D2/ATP6V1E1/NDUFA10/LOC101796232/COX1/LOC119714948/ND4/ND5/CYTB/ATP5PO	apla:5405821/apla:5405821/apla:5405818/apla:101805119/apla:5405821/apla:106014280/apla:101802167/apla:101793897/apla:101795476/apla:101796232/apla:5405810/apla:5405819/apla:5405811/apla:5405820/apla:5405813/apla:101790402	16	14	119	106014280/101793897

apla04068	FoxO signaling pathway	16/452	118/4924	0.071487	0.40861	101791448/101798341/101797555/101795002/101799561/101799252/101793597/101795633/101800900/101801641/113845197/101804723/101791694/101800042/101794326/101791037	GABARAPL1/BNIP3/KLF2/EGF/PRKAA2/FBXO32/IGF1/RAF1/PIK3CB/LOC101801641/KLF17/IRS2/CDKN1B/IL7R/HOMER2/MAP2K1	apla:101791448/apla:101798341/apla:101797555/apla:101795002/apla:101799561/apla:101799252/apla:101793597/apla:101795633/apla:101800900/apla:101801641/apla:113845197/apla:101804723/apla:101791694/apla:101800042/apla:101794326/apla:101791037	16	3	101	101791448/101798341/101797555/101799252/101795633/101800900/101801641/113845197/101804723/101791694/101800042/101794326/101791037
apla02010	ABC transporters	9/452	57/4924	0.07323	0.40861	101802246/101799966/119713166/119717010/110354063/119718656/101801002/101804688/101802999	ABCB10/ABCG8/LOC119713166/LOC119717010/LOC110354063/LOC119718656/ABCA2/ABCC8/ABCC1	apla:101802246/apla:101799966/apla:101795490/apla:101804688/apla:101793054/apla:101797091/apla:101801002/apla:101804688/apla:101802999	9	7	101	119713166/119718656
apla00061	Fatty acid biosynthesis	4/452	18/4924	0.076249	0.40861	101799889/101798003/101798039/101795358	ACSBG2/ACSL4/ACSL1/ACSBG1	apla:101799889/apla:101798003/apla:101798039/apla:101795358	4	1	##	101799889/101798003/101795358
apla00260	Glycine, serine and threonine metabolism	7/452	41/4924	0.07675	0.40861	101803151/101802407/113842961/101802594/101793275/101802992/101794860	BPGM/LOC101802407/GNMT/DMGDH/ALDH7A1/LOC101802992/PSAT1	apla:101803151/apla:101802407/apla:113842961/apla:101802594/apla:101793275/apla:101802992/apla:101794860	7	4	101	113842961/101793275/101794860
apla04060	Cytokine-cytokine receptor interaction	21/452	166/4924	0.079452	0.40861	101798302/101800940/101794837/101795448/101795038/101800483/101800742/101798887/101793878/101802610/101791747/119713491/101803777/119717811/101794826/101801641/101797725/101793707/101800042/101796903/101804501	BMP3/LOC101800940/CCL22/INHBA/CX3CL1/GHR/MSTN/EDAR/LOC101793878/CCR9/CSF3R/LOC119713491/TNFSF13B/EDA2R/ACVR1C/LOC101801641/TNFRSF19/BMPR2/IL7R/LOC101796903/IL10RB	apla:101798302/apla:101800940/apla:101794837/apla:101795448/apla:101795038/apla:101800483/apla:101800742/apla:101798887/apla:101793878/apla:101802610/apla:101791747/apla:101799861/apla:101803777/apla:101797725/apla:101794826/apla:101801641/apla:101797725/apla:101793707/apla:101800042/apla:101796903/apla:101804501	21	8	101	101794837/101798887/101793878/101802610/101791747/119713491/101803777/119717811/101794826/101801641/101800042/101796903/101804501
apla04350	TGF-beta signaling pathway	12/452	85/4924	0.086067	0.427368	101795448/101792191/101803434/101793322/101789702/101802543/101794826/101801641/101792754/101793707/101796903/101791584	INHBA/GREM2/PITX2/THBS1/GREM1/LOC101802543/ACVR1C/LOC101801641/BAMBI/BMPR2/LOC101796903/SMAD6	apla:101795448/apla:101792191/apla:101803434/apla:101793322/apla:101789702/apla:101802543/apla:101794826/apla:101801641/apla:101792754/apla:101793707/apla:101796903/apla:101791584	12	5	101	101793322/101802543/101794826/101801641/101792754/101796903/101791584
apla04140	Autophagy - animal	18/452	141/4924	0.092745	0.443704	101791448/101798341/101793905/101803168/101799561/113839605/113841783/101802164/101798359/101795633/101794268/101800900/106016621/101804723/101791037/101802364/110353775/101790152	GABARAPL1/BNIP3/ULK2/CTSL/PRKAA2/LOC113839605/ATG4A/MTMR4/RAB7B/RAF1/CTSB/PIK3CB/TP53INP2/IRS2/MAP2K1/MRAS/LOC110353775/ATG2B	apla:101791448/apla:101798341/apla:101793905/apla:101803168/apla:101799561/apla:101790109/apla:101791479/apla:101802164/apla:101798359/apla:101795633/apla:101794268/apla:101800900/apla:106016621/apla:101804723/apla:101791037/apla:101802364/apla:101796703/apla:101790152	18	4	101	101791448/101798341/101793905/101803168/113839605/101802164/101798359/101795633/101794268/101800900/101804723/101791037/110353775/101790152
apla00410	beta-Alanine metabolism	5/452	27/4924	0.09552	0.443704	101798008/113839590/101793275/119713271/119714639	GADL1/LOC113839590/ALDH7A1/LOC119713271/ACADM	apla:101798008/apla:113839590/apla:101793275/apla:101792386/apla:101801937	5	2	101	113839590/101793275/119713271
apla04371	Apelin signaling pathway	16/452	124/4924	0.101019	0.454584	101790193/101797555/106020638/101800617/101804354/101790966/101803512/101799561/101799116/101795118/119718626/101798929/101795633/113845197/101791037/101802364	CCN2/KLF2/APLNR/ADCY9/PPARGC1A/PLCB1/ADCY2/PRKAA2/MEF2D/MEF2A/KREMEN1/SLC8A3/RAF1/KLF17/MAP2K1/MRAS	apla:101790193/apla:101797555/apla:106020638/apla:101800617/apla:101804354/apla:101790966/apla:101803512/apla:101799561/apla:101799116/apla:101795118/apla:101790558/apla:101798929/apla:101795633/apla:113845197/apla:101791037/apla:101802364	16	11	106	101790193/101797555/101795633/113845197/101791037
apla00053	Ascorbate and aldarate metabolism	3/452	13/4924	0.110016	0.467353	113839590/101793275/119713271	LOC113839590/ALDH7A1/LOC119713271	apla:113839590/apla:101793275/apla:101792386	3	0		113839590/101793275/119713271
apla04020	Calcium signaling pathway	27/452	231/4924	0.110533	0.467353	101805261/110354206/101802855/101800617/101789607/101794809/106016351/101790659/101790966/101795002/101803512/101793664/101795278/101793068/119717133/101802849/101799246/101798929/119713505/101801311/101801648/101801144/101803502/101796880/101802609/101804306/101797327	ATP2A2/FGFR4/CAMK1G/ADCY9/HTR5A/MCOLN3/TRDN/NTRK3/PLCB1/EGF/ADCY2/HGF/LOC101795278/PHKG1/P2RX3/PHKA2/PLCG2/SLC8A3/TACR1/LOC101801311/CAMK2D/ERBB4/PDGFA/PLCD3/PPIF/PTGFR/LOC101797327	apla:101805261/apla:110354206/apla:101802855/apla:101800617/apla:101789607/apla:101794809/apla:101795831/apla:101790659/apla:101790966/apla:101795002/apla:101803512/apla:101793664/apla:101795278/apla:101793068/apla:101797369/apla:101802849/apla:101799246/apla:101798929/apla:101797865/apla:101801311/apla:101801648/apla:101801144/apla:101803502/apla:101796325/apla:101802609/apla:101804306/apla:101797327	27	21	101	101794809/101793664/101795278/101793068/101799246/101796880
apla00270	Cysteine and methionine metabolism	7/452	45/4924	0.113593	0.467353	101799171/101802407/101801152/101799210/119716363/101804610/101794860	MAT1A/LOC101802407/LDHB/LOC101799210/DNMT3A/MDH2/PSAT1	apla:101799171/apla:101802407/apla:101801152/apla:101799210/apla:101794583/apla:101804610/apla:101794860	7	6	101	101794860
apla00340	Histidine metabolism	4/452	21/4924	0.120344	0.481374	113839590/113844155/101793275/119713271	LOC113839590/LOC113844155/ALDH7A1/LOC119713271	apla:113839590/apla:113844155/apla:101793275/apla:101792386	4	1	##	113839590/101793275/119713271
apla04916	Melanogenesis	11/452	83/4924	0.135914	0.528963	101791711/101800617/101790966/101803512/101799597/101795633/101799824/101801648/101795047/101792356/101791037	WNT4/ADCY9/PLCB1/ADCY2/FZD9/RAF1/WNT2/CAMK2D/MITF/WNT5B/MAP2K1	apla:101791711/apla:101800617/apla:101790966/apla:101803512/apla:101799597/apla:101795633/apla:101800865/apla:101801648/apla:101795047/apla:101792356/apla:101791037	11	6	101	101795633/101799824/101795047/101792356/101791037

apla00533	Glycosaminoglycan biosynthesis - keratan sulfate	3/452	15/4924	0.153092	0.580139	101792176/101793948/119717607	B4GALT4/B3GNT2/B4GALT2	apla:101792176/apla:101793948/apla:101792817	3	2	101	101793948
apla00650	Butanoate metabolism	4/452	24/4924	0.172907	0.6271	101797999/119714639/101793939/101804444	BDH1/ACADM/ALDH5A1/L2HGDH	apla:101797999/apla:101801937/apla:101793939/apla:101804444	4	3	119	101797999
apla00830	Retinol metabolism	5/452	33/4924	0.180803	0.6271	101801759/101798614/101792573/101801058/101792259	ALDH1A2/BCO1/LOC101792573/LOC101801058/DHRS3	apla:101801759/apla:101798614/apla:101792573/apla:101801058/apla:101792259	5	3	101	101792573/101792259
apla00230	Purine metabolism	14/452	117/4924	0.182904	0.6271	101803104/101794720/101800617/101801928/101796581/101793480/101803512/101790428/101803472/101796516/101792445/101800542/101801878/101790379	AMPD3/AMPD1/ADCY9/ADSS1/PDE9A/ADA2/ADCY2/PGM1/ITPA/LOC101796516/NUDT5/ENPP3/ADSL/ENPP4	apla:101803104/apla:101794720/apla:101800617/apla:101801928/apla:101796581/apla:101793480/apla:101803512/apla:101790428/apla:101803472/apla:101796516/apla:101792445/apla:101800542/apla:101801878/apla:101790379	14	11	101	101803104/101803472/101790379
apla04261	Adrenergic signaling in cardiomyocytes	14/452	117/4924	0.182904	0.6271	101805261/101798527/101800617/119714808/101790966/101803512/101802358/101797962/113845023/101798929/101801311/101801648/101803316/101793350	ATP2A2/PPP2R2D/ADCY9/ATP1B2/PLCB1/ADCY2/CACNG4/CREB5/CACNA2D2/SLC8A3/LOC101801311/CAMK2D/LOC101803316/CACNA2D1	apla:101805261/apla:101797307/apla:101800617/apla:101795008/apla:101790966/apla:101803512/apla:101802358/apla:101797962/apla:101793350/apla:101798929/apla:101801311/apla:101801648/apla:101803316/apla:101793350	14	11	101	101798527/101797962/101803316
apla00020	Citrate cycle (TCA cycle)	4/452	25/4924	0.191946	0.628188	101789492/101800289/101804610/101799937	FH/SUCLG2/MDH2/SUCLG1	apla:101789492/apla:101800289/apla:101804610/apla:101799937	4	4	101789492/101800289/101804610/101799937	
apla00790	Folate biosynthesis	4/452	25/4924	0.191946	0.628188	101790101/101794661/101790897/101800658	MOCOS/LOC101794661/GGH/PCBD1	apla:101790101/apla:101794661/apla:101790897/apla:101800658	4	3	101	101800658
apla03050	Proteasome	5/452	34/4924	0.197164	0.630925	101798380/101802149/101798317/101794257/101803700	PSMC3/ADRM1/PSME4/PSMD6/PSMB1	apla:101798380/apla:101802149/apla:101798317/apla:101794257/apla:101803700	5	0		101798380/101802149/101798317/101794257/101803700
apla04150	mTOR signaling pathway	16/452	139/4924	0.203218	0.636161	101795185/101798286/101793905/101791711/101799561/101800942/101804773/101793897/101799597/101793597/101795633/101800900/101799824/101792356/101791037/101790650	LPIN1/EIF4EBP1/ULK2/WNT4/PRKAA2/RPS6KA1/CLIP1/ATP6V1E1/FZD9/IGF1/RAF1/PIK3CB/WNT2/WNT5B/MAP2K1/LRP5	apla:101795185/apla:101798286/apla:101793905/apla:101791711/apla:101799561/apla:101800942/apla:101804773/apla:101793897/apla:101799597/apla:101793597/apla:101795633/apla:101800900/apla:101800865/apla:101792356/apla:101791037/apla:101790650	16	5	101	101795185/101798286/101793905/101800942/101804773/101793897/101795633/101800900/101799824/101792356/101791037
apla00562	Inositol phosphate metabolism	9/452	73/4924	0.2235	0.666125	101795498/101790966/101805442/101799246/101802164/101798206/101800900/101790798/101796880	PIP4K2A/PLCB1/IMPA2/PLCG2/MTMR4/INPP5K/PIK3CB/TPI1/PLCD3	apla:101795498/apla:101790966/apla:101805442/apla:101799246/apla:101802164/apla:101798206/apla:101800900/apla:101790798/apla:101796325	9	4	101	101799246/101802164/101798206/101800900/101796880
apla03430	Mismatch repair	3/452	18/4924	0.225296	0.666125	101796914/101802532/101797382	LIG1/MSH2/POLD3	apla:101796914/apla:101802532/apla:101797382	3	1	##	101796914/101802532
apla04912	GnRH signaling pathway	11/452	93/4924	0.230953	0.666125	101803371/101800617/101790966/101803512/101796626/101795633/101801311/101801648/110353809/101791037/110353775	MAP2K3/ADCY9/PLCB1/ADCY2/JUN/RAF1/LOC101801311/CAMK2D/LOC110353809/MAP2K1/LOC110353775	apla:101803371/apla:101800617/apla:101790966/apla:101803512/apla:101796626/apla:101795633/apla:101801311/apla:101801648/apla:101793608/apla:101791037/apla:101796703	11	6	101	101803371/101796626/101795633/101791037/110353775
apla04216	Ferroptosis	5/452	36/4924	0.231293	0.666125	101795318/101803068/101798003/101805057/101798039	SAT1/TFRC/ACSL4/HMOX1/ACSL1	apla:101795318/apla:101803068/apla:101798003/apla:101805057/apla:101798039	5	2	101	101795318/101798003/101805057
apla04810	Regulation of actin cytoskeleton	22/452	205/4924	0.248247	0.684114	110354206/101803833/119714715/101795498/101795371/101796600/101800557/101799485/101803245/101795002/101803666/101796164/101802656/101800906/101790881/101791104/101795633/101800900/101796803/101803502/101791037/101802364	FGFR4/RGCC/ITGA7/PIP4K2A/LOC101795371/PAK3/ENAH/ITGA4/SSH2/EGF/ITGB3/ITGA8/VAV2/CYFIP2/KNG1/ITGA6/RAF1/PIK3CB/ITGB2/PDGFA/MAP2K1/MRAS	apla:110354206/apla:101803833/apla:101791104/apla:101795498/apla:101795371/apla:101796600/apla:101800557/apla:101799485/apla:101803245/apla:101795002/apla:101803666/apla:101796164/apla:101802656/apla:101800906/apla:101790881/apla:101791104/apla:101795633/apla:101800900/apla:101796803/apla:101803502/apla:101791037/apla:101802364	22	12	110	101803833/101795371/101800557/101799485/101803245/101803666/101795633/101800900/101796803/101791037
apla04540	Gap junction	10/452	85/4924	0.250586	0.684114	101802614/101800617/101790966/119714718/101795002/101803512/101796516/101795633/101803502/101791037	PRKG1/ADCY9/PLCB1/LOC119714718/EGF/ADCY2/LOC101796516/RAF1/PDGFA/MAP2K1	apla:101802614/apla:101800617/apla:101790966/apla:101795602/apla:101795002/apla:101803512/apla:101796516/apla:101795633/apla:101803502/apla:101791037	10	7	101	119714718/101795633/101791037
apla04210	Apoptosis	14/452	125/4924	0.254684	0.684114	101803168/101802390/101801698/119714718/101804246/113839605/119713491/119717912/101796626/101795633/101794268/101800900/101791236/101791037	CTSL/DDIT3/FOS/LOC119714718/CTSZ/LOC113839605/LOC119713491/LOC119717912/JUN/RAF1/CTSB/PIK3CB/MAP3K14/MAP2K1	apla:101803168/apla:101802390/apla:101801698/apla:101795602/apla:101804246/apla:101790109/apla:101799861/apla:101798194/apla:101796626/apla:101795633/apla:101794268/apla:101800900/apla:101791236/apla:101791037	14	1	##	101803168/101802390/101801698/119714718/101804246/113839605/119713491/101796626/101795633/101794268/101800900/101791236/101791037
apla01230	Biosynthesis of amino acids	8/452	66/4924	0.256543	0.684114	101799171/novel.282/101799450/101797455/101790726/101802992/101790798/101794860	MAT1A/-/GLUL/ASNS/PKM/LOC101802992/TPI1/PSAT1	apla:101799171/apla:101804818/apla:101799450/apla:101797455/apla:101790726/apla:101802992/apla:101790798/apla:101794860	8	4	101	novel.282/101799450/101797455/101794860

apla00630	Glyoxylate and dicarboxylate metabolism	4/452	29/4924	0.273422	0.715868	101803542/101799450/101804610/101796139	ACSS2/GLUL/MDH2/HYI	apla:101803542/apla:101799450/apla:101804610/apla:101796139	4	3	101	101799450
apla04310	Wnt signaling pathway	16/452	149/4924	0.290606	0.747272	101791711/101794947/119712976/101804305/101790966/113840125/101791294/101799597/101796626/101799824/101801648/101792754/101792356/101791686/101793483/101790650	WNT4/SFRP2/CXXC4/RSPO3/PLCB1/TLE4/FRZB/FZD9/JUN/WNT2/CAMK2D/BAMBI/WNT5B/CCDC88C/NKD1/LRP5	apla:101791711/apla:101794947/apla:106020086/apla:101804305/apla:101790966/apla:113840125/apla:101791294/apla:101799597/apla:101796626/apla:101800865/apla:101801648/apla:101792754/apla:101792356/apla:101791686/apla:101793483/apla:101790650	16	11	101	113840125/101796626/101799824/101792754/101792356
apla00601	Glycosphingolipid biosynthesis - lacto and neolacto series	3/452	21/4924	0.302482	0.7598	101792176/101793948/119717607	B4GALT4/B3GNT2/B4GALT2	apla:101792176/apla:101793948/apla:101792817	3	2	101	101793948
apla04910	Insulin signaling pathway	13/452	120/4924	0.306061	0.7598	101793994/101798286/101804354/101790963/101799561/101793068/101802849/101795633/101800900/119713467/101804723/101791037/101791200	PPP1R3C/EIF4EBP1/PPARGC1A/PPP1R3D/PRKAA2/PHKG1/PHKA2/RAF1/PIK3CB/HK2/IRS2/MAP2K1/SOCS3	apla:101793994/apla:101798286/apla:101804354/apla:101790963/apla:101799561/apla:101793068/apla:101802849/apla:101795633/apla:101800900/apla:101794107/apla:101804723/apla:101791037/apla:101791200	13	5	101	101798286/101793068/101795633/101800900/119713467/101804723/101791037/101791200
apla04914	Progesterone-mediated oocyte maturation	9/452	80/4924	0.311307	0.7598	101800617/101792368/101803512/101800942/101793597/101795633/101800900/101791037/101795780	ADCY9/CCNA2/ADCY2/RPS6KA1/IGF1/RAF1/PIK3CB/MAP2K1/MAD1L1	apla:101800617/apla:101792368/apla:101803512/apla:101800942/apla:101793597/apla:101795633/apla:101800900/apla:101791037/apla:101795780	9	4	101	101792368/101800942/101795633/101800900/101791037
apla04137	Mitophagy - animal	7/452	61/4924	0.325895	0.782147	101791448/101798341/101798359/101796626/101795047/101801576/101802364	GABARAPL1/BNIP3/RAB7B/JUN/MITF/E2F1/MRAS	apla:101791448/apla:101798341/apla:101798359/apla:101796626/apla:101795047/apla:101801576/apla:101802364	7	2	101	101791448/101798341/101798359/101796626/101795047
apla00051	Fructose and mannose metabolism	4/452	33/4924	0.359421	0.813146	101795985/119713467/101790798/101793695	LOC101795985/HK2/TPI1/TIGAR	apla:101795985/apla:101794107/apla:101790798/apla:101793695	4	1	##	101795985/119713467/101793695
apla04145	Phagosome	14/452	136/4924	0.366605	0.813146	101803168/101803068/101793322/119714718/101803666/113839605/101796495/101802167/101793897/101805179/101798359/119718656/101796803/101794894	CTSL/TFRC/THBS1/LOC119714718/ITGB3/LOC113839605/CD36/ATP6V0D2/ATP6V1E1/EEA1/RAB7B/LOC119718656/ITGB2/SEC61B	apla:101803168/apla:101803068/apla:101793322/apla:101795602/apla:101803666/apla:101790109/apla:101796495/apla:101802167/apla:101793897/apla:101805179/apla:101798359/apla:101797091/apla:101796803/apla:101794894	14	4	101	101803168/101793322/119714718/101803666/113839605/101793897/101798359/119718656/101796803/101794894
apla04146	Peroxisome	8/452	74/4924	0.36846	0.813146	101791336/101798003/101803656/101803303/101804343/101799736/101798712/101798039	CRAT/ACSL4/MPV17L2/SOD1/ACOX2/FAR2/PECR/ACSL1	apla:101791336/apla:101798003/apla:101803656/apla:101803303/apla:101804343/apla:101799736/apla:101798712/apla:101798039	8	5	101	101798003/101803656/101803303
apla00120	Primary bile acid biosynthesis	2/452	14/4924	0.372776	0.813146	101804343/101791081	ACOX2/LOC101791081	apla:101804343/apla:101791081	2	2	101804343/101791081	
apla00534	Glycosaminoglycan biosynthesis - heparan sulfate / heparin	3/452	24/4924	0.380685	0.813146	101800670/101790534/101793969	NDST1/HS6ST2/HS3ST3B1	apla:101800670/apla:101790534/apla:101793969	3	3	101800670/101790534/101793969	
apla00052	Galactose metabolism	4/452	34/4924	0.381056	0.813146	101790428/113845291/119717607/119713467	PGM1/LOC113845291/B4GALT2/HK2	apla:101790428/apla:113845291/apla:101792817/apla:101794107	4	3	101	119713467
apla00380	Tryptophan metabolism	4/452	34/4924	0.381056	0.813146	113839590/101793275/119713271/101801193	LOC113839590/ALDH7A1/LOC119713271/ALDH8A1	apla:113839590/apla:101793275/apla:101792386/apla:101801193	4	0		113839590/101793275/119713271/101801193
apla00310	Lysine degradation	6/452	55/4924	0.392832	0.813146	101793380/113839590/101793275/119713271/101791765/101799596	AASS/LOC113839590/ALDH7A1/LOC119713271/EZH1/CAMKMT	apla:101793380/apla:113839590/apla:101793275/apla:101792386/apla:101791765/apla:101799596	6	2	101	101793380/113839590/101793275/119713271
apla01250	Biosynthesis of nucleotide sugars	4/452	35/4924	0.40261	0.813146	101790428/119713467/101791122/101797601	PGM1/HK2/GNPNAT1/GPI	apla:101790428/apla:101794107/apla:101791122/apla:101797601	4	2	101	119713467/101791122
apla04136	Autophagy - other	4/452	35/4924	0.40261	0.813146	101791448/101793905/113841783/101790152	GABARAPL1/ULK2/ATG4A/ATG2B	apla:101791448/apla:101793905/apla:101791479/apla:101790152	4	1	##	101791448/101793905/101790152
apla00030	Pentose phosphate pathway	3/452	25/4924	0.406422	0.813146	101790428/101803045/101797601	PGM1/DERA/GPI	apla:101790428/apla:101803045/apla:101797601	3	3	101790428/101803045/101797601	
apla00511	Other glycan degradation	2/452	15/4924	0.406573	0.813146	101798829/101792144	NEU2/FUCA2	apla:101798829/apla:101792144	2	1	##	101792144
apla04070	Phosphatidylinositol signaling system	9/452	89/4924	0.4323	0.852756	101795498/101790966/101805442/101799246/101802164/101800900/101801143/101796880/101797078	PIP4K2A/PLCB1/IMPA2/PLCG2/MTMR4/PIK3CB/DGKI/PLCD3/IP6K2	apla:101795498/apla:101790966/apla:101805442/apla:101799246/apla:101802164/apla:101800900/apla:101801143/apla:101796325/apla:101797078	9	4	101	101799246/101802164/101800900/101796880/101797078
apla04115	p53 signaling pathway	6/452	59/4924	0.460385	0.889332	101800755/101793322/101803685/119713491/101793597/101804011	SESN1/THBS1/IGFBP3/LOC119713491/IGF1/SESN3	apla:101800755/apla:101793322/apla:101803685/apla:101799861/apla:101793597/apla:101804011	6	2	101	101800755/101793322/101803685/119713491
apla03440	Homologous recombination	4/452	38/4924	0.46622	0.889332	113845646/101793627/101790058/101797382	BABAM1/BABAM2/BLM/POLD3	apla:113845646/apla:101793627/apla:101790058/apla:101797382	4	4	113845646/101793627/101790058/101797382	
apla01232	Nucleotide metabolism	8/452	81/4924	0.46937	0.889332	101803104/101794720/101801928/101793480/101803472/101804715/101800542/101801878	AMPD3/AMPD1/ADSS1/ADA2/ITPA/DTYMK/ENPP3/ADSL	apla:101803104/apla:101794720/apla:101801928/apla:101793480/apla:101803472/apla:101804715/apla:101800542/apla:101801878	8	6	101	101803104/101803472
apla04270	Vascular smooth muscle contraction	12/452	125/4924	0.480464	0.89853	113844456/101802614/101800617/101792241/101792394/101790966/101803512/101796516/101795633/101801311/101791037/110353775	NPPC/PRKG1/ADCY9/KCNMA1/RAMP1/PLCB1/ADCY2/LOC101796516/RAF1/LOC101801311/MAP2K1/LOC110353775	apla:113844456/apla:101802614/apla:101800617/apla:101792241/apla:101792394/apla:101790966/apla:101803512/apla:101796516/apla:101795633/apla:101801311/apla:101791037/apla:101796703	12	8	101	113844456/101795633/101791037/110353775

apla00564	Glycerophospholipid metabolism	10/452	106/4924	0.513356	0.947735	101797414/101795185/101802263/101798440/101803103/101790851/101799735/101801143/101804047/101800297	LOC101797414/LPIN1/LPCAT2/LOC101798440/GPAM/CHKA/GPD2/DGKI/PTDSS1/MBOAT2	apla:101797414/apla:101795185/apla:101802263/apla:101798440/apla:101803103/apla:101790851/apla:101799735/apla:101801143/apla:101804047/apla:101800297	10	3	101	101797414/101795185/101802263/101798440/101790851/101799735/101800297
apla00512	Mucin type O-glycan biosynthesis	3/452	30/4924	0.528769	0.956997	101796875/101805478/101792568	GCNT1/GALNT12/GALNT18	apla:101796875/apla:101805478/apla:101792568	3	1	##	101796875/101805478
apla00532	Glycosaminoglycan biosynthesis - chondroitin sulfate / dermatan sulfate	2/452	19/4924	0.531665	0.956997	113840298/101799131	CHST7/CSGALNACT1	apla:113840298/apla:101799131	2	2	113840298/101799131	
apla01040	Biosynthesis of unsaturated fatty acids	3/452	31/4924	0.551547	0.980528	101796694/101801349/101790208	LOC101796694/SCD/SCD5	apla:101796694/apla:101801349/apla:101790208	3	2	101	101801349
apla04672	Intestinal immune network for IgA production	4/452	43/4924	0.565987	0.993929	101799485/101802610/101803777/101791236	ITGA4/CCR9/TNFSF13B/MAP3K14	apla:101799485/apla:101802610/apla:101803777/apla:101791236	4	0		101799485/101802610/101803777/101791236
apla04141	Protein processing in endoplasmic reticulum	13/452	145/4924	0.57943	0.999967	101798444/101803640/101802390/101798550/101790223/101797658/119713089/106017251/101792755/101801245/101792011/101794894/101799929	BAG2/DERL3/DDIT3/HSPH1/UBE2D3/HSPA2/HSPA5/HERPUD1/LOC101792755/SELENOS/UGGT2/SEC61B/UBXN4	apla:101798444/apla:101803640/apla:101802390/apla:101798550/apla:101790223/apla:101797658/apla:101801738/apla:106017251/apla:101792755/apla:101801245/apla:101792011/apla:101794894/apla:101799929	13	1	##	101798444/101803640/101802390/101798550/101790223/101797658/119713089/106017251/101792755/101801245/101794894/101799929
apla00220	Arginine biosynthesis	2/452	21/4924	0.58713	0.999967	101799450/101797377	GLUL/GLUD1	apla:101799450/apla:101797377	2	0		101799450/101797377
apla04080	Neuroactive ligand-receptor interaction	27/452	304/4924	0.604683	0.999967	106020272/110354431/101805348/101800483/106020638/101789607/101792889/101803050/101796159/101793595/101802295/119715526/101792479/101800466/101804394/101795278/119717133/101794378/101790881/119713505/110353809/101803483/101801352/119714451/101798462/101804306/101801041	CHRNG/LOC110354431/TSPO2/GHR/APLNR/HTR5A/S1PR3/CRH/LOC101796159/LPAR3/UTS2R/LOC119715526/THRB/CHRNA10/P2RY1/LOC101795278/P2RX3/NPY2R/KNG1/TACR1/LOC110353809/LOC101803483/CNR1/CHRNB3/GABRG3/PTGFR/VIPR2	apla:106020272/apla:101803263/apla:101805348/apla:101800483/apla:106020638/apla:101789607/apla:101792889/apla:101803050/apla:101796159/apla:101793595/apla:101802295/apla:101803263/apla:113839582/apla:101800466/apla:101804394/apla:101795278/apla:101797369/apla:101794378/apla:101790881/apla:101797865/apla:101793608/apla:101803483/apla:101801352/apla:101794760/apla:101798462/apla:101804306/apla:101801041	27	22	106	101805348/101803050/101800466/101795278/101801041
apla00480	Glutathione metabolism	4/452	46/4924	0.620657	0.999967	101790132/101804534/101804478/119713930	ODC1/LOC101804534/PRDX6/LOC119713930	apla:101790132/apla:101804534/apla:101804478/apla:113839739	4	3	101	101790132
apla00520	Amino sugar and nucleotide sugar metabolism	4/452	46/4924	0.620657	0.999967	101790428/119713467/101791122/101797601	PGM1/HK2/GNPNAT1/GPI	apla:101790428/apla:101794107/apla:101791122/apla:101797601	4	2	101	119713467/101791122
apla04142	Lysosome	10/452	116/4924	0.631582	0.999967	101803168/101803026/101804246/113845291/113839605/101802167/101794268/101801002/101792144/101800183	CTSL/NPC1/CTSZ/LOC113845291/LOC113839605/ATP6V0D2/CTSB/ABCA2/FUCA2/HYAL2	apla:101803168/apla:101803026/apla:101804246/apla:113845291/apla:101790109/apla:101802167/apla:101794268/apla:101801002/apla:101792144/apla:101800183	10	3	113	101803168/101803026/101804246/113839605/101794268/101792144/101800183
apla00515	Mannose type O-glycan biosynthesis	2/452	23/4924	0.637579	0.999967	101803248/119717607	B3GALNT2/B4GALT2	apla:101803248/apla:101792817	2	1	##	101803248
apla03410	Base excision repair	2/452	23/4924	0.637579	0.999967	101796914/101797382	LIG1/POLD3	apla:101796914/apla:101797382	2	1	##	101796914
apla01240	Biosynthesis of cofactors	10/452	118/4924	0.653364	0.999967	101799171/113839590/101801928/119713271/101798614/110351624/101794860/101790897/101792259/101801878	MAT1A/LOC113839590/ADSS1/LOC119713271/BCO1/LOC110351624/PSAT1/GGH/DHRS3/ADSL	apla:101799171/apla:113839590/apla:101801928/apla:101792386/apla:101798614/apla:110351624/apla:101794860/apla:101790897/apla:101792259/apla:101801878	10	6	101	113839590/119713271/101794860/101792259
apla00062	Fatty acid elongation	2/452	24/4924	0.660933	0.999967	101796694/101791052	LOC101796694/ACAA2	apla:101796694/apla:101791052	2	2	101796694/101791052	
apla03450	Non-homologous end-joining	1/452	12/4924	0.685504	0.999967	101797870	XRCC5	apla:101797870	1	1	##	
apla04620	Toll-like receptor signaling pathway	6/452	75/4924	0.698205	0.999967	101803371/101801698/101796626/101800900/101791769/101791037	MAP2K3/FOS/JUN/PIK3CB/MAP3K8/MAP2K1	apla:101803371/apla:101801698/apla:101796626/apla:101800900/apla:101791769/apla:101791037	6	0		101803371/101801698/101796626/101800900/101791769/101791037
apla00980	Metabolism of xenobiotics by cytochrome P450	2/452	26/4924	0.704001	0.999967	101794661/119713930	LOC101794661/LOC119713930	apla:101794661/apla:113839739	2	2	101794661/119713930	
apla04621	NOD-like receptor signaling pathway	10/452	123/4924	0.70454	0.999967	101791448/101797616/101790966/101789899/113839639/101800595/101796626/101794268/110353775/101804092	GABARAPL1/LOC101797616/PLCB1/LOC101789899/LOC113839639/RNASEL/JUN/CTSB/LOC110353775/TXN	apla:101791448/apla:101797616/apla:101790966/apla:101789899/apla:113839639/apla:101800595/apla:101796626/apla:101794268/apla:101796703/apla:101804092	10	2	101	101791448/101797616/101789899/113839639/101796626/101794268/110353775/101804092

apla04218	Cellular senescence	11/452	135/4924	0.707077	0.999967	101798286/101803371/101803685/101792368/101795633/101800900/101801641/101797121/101791037/101801576/101802364	EIF4EBP1/MAP2K3/IGFBP3/CCNA2/RAF1/PIK3CB/LOC101801641/ZFP36L1/MAP2K1/E2F1/MRAS	apla:101798286/apla:101803371/apla:101803685/apla:101792368/apla:101795633/apla:101800900/apla:101801641/apla:101797121/apla:101791037/apla:101801576/apla:101802364	11	2	101	101798286/101803371/101803685/101792368/101795633/101800900/101801641/101797121/101791037
apla04530	Tight junction	12/452	147/4924	0.709776	0.999967	101794157/101798527/101795371/101791895/101799421/119714718/101794589/101799561/119714211/101799514/101796626/101798171	WHAMM/PPP2R2D/LOC101795371/LOC101791895/LOC101799421/LOC119714718/YBX3/PRKAA2/LOC119714211/AMOTL2/JUN/LLGL2	apla:101794157/apla:101797307/apla:101795371/apla:101791895/apla:101799421/apla:101795602/apla:101794589/apla:101799561/apla:101794832/apla:101799514/apla:101796626/apla:101798171	12	2	101	101794157/101798527/101795371/101791895/119714718/101794589/119714211/101799514/101796626/101798171
apla00604	Glycosphingolipid biosynthesis - ganglio series	1/452	13/4924	0.714443	0.999967	101793440	ST6GALNAC6	apla:101793440	1	1	##	
apla00514	Other types of O-glycan biosynthesis	3/452	40/4924	0.724657	0.999967	119717607/101805478/101792568	B4GALT2/GALNT12/GALNT18	apla:101792817/apla:101805478/apla:101792568	3	2	119	101805478
apla00430	Taurine and hypotaurine metabolism	1/452	14/4924	0.740726	0.999967	101798008	GADL1	apla:101798008	1	1	##	
apla03030	DNA replication	2/452	28/4924	0.74242	0.999967	101796914/101797382	LIG1/POLD3	apla:101796914/apla:101797382	2	1	##	101796914
apla04130	SNARE interactions in vesicular transport	2/452	28/4924	0.74242	0.999967	101800849/101802775	VAMP4/BNIP1	apla:101800849/apla:101802775	2	0		101800849/101802775
apla05132	Salmonella infection	19/452	236/4924	0.763495	0.999967	101790249/101803371/101797616/101792273/101795371/101796600/101801698/119714718/101789899/119713491/119715213/101800906/101798359/101796626/101795633/101800900/101791037/101804092/101804925	ANXA1/MAP2K3/LOC101797616/ANXA2/LOC101795371/PAK3/FOS/LOC119714718/LOC101789899/LOC119713491/LOC119715213/CYFIP2/RAB7B/JUN/RAF1/PIK3CB/MAP2K1/TXN/RAB9A	apla:101792273/apla:101803371/apla:101797616/apla:101792273/apla:101795371/apla:101796600/apla:101801698/apla:101795602/apla:101789899/apla:101799861/apla:101796074/apla:101800906/apla:101798359/apla:101796626/apla:101795633/apla:101800900/apla:101791037/apla:101804092/apla:101804925	19	2	101	101790249/101803371/101797616/101792273/101795371/101801698/119714718/101789899/119713491/119715213/101798359/101796626/101795633/101800900/101791037/101804092/101804925
apla00590	Arachidonic acid metabolism	4/452	56/4924	0.769324	0.999967	101790348/101795486/101792912/101794661	PTGR2/HPGD/LOC101792912/LOC101794661	apla:101790348/apla:101795486/apla:101792912/apla:101794661	4	3	101	101795486
apla04330	Notch signaling pathway	4/452	56/4924	0.769324	0.999967	113840125/119714472/101789669/101804919	TLE4/LOC119714472/NUMB/HEYL	apla:113840125/apla:113839891/apla:101789669/apla:101804919	4	2	101	113840125/119714472
apla04370	VEGF signaling pathway	5/452	70/4924	0.783218	0.999967	113845585/101799246/101795633/101800900/101791037	HSPB1/PLCG2/RAF1/PIK3CB/MAP2K1	apla:113845585/apla:101799246/apla:101795633/apla:101800900/apla:101791037	5	0		113845585/101799246/101795633/101800900/101791037
apla00140	Steroid hormone biosynthesis	2/452	32/4924	0.806565	0.999967	101791081/101799397	LOC101791081/SRD5A2	apla:101791081/apla:101799397	2	2	101791081/101799397	
apla00531	Glycosaminoglycan degradation	1/452	18/4924	0.823827	0.999967	101800183	HYAL2	apla:101800183	1	0		101800183
apla04114	Oocyte meiosis	7/452	101/4924	0.832515	0.999967	101800617/101803512/101800942/101793597/101801648/101791037/101795780	ADCY9/ADCY2/RPS6KA1/IGF1/CAMK2D/MAP2K1/MAD1L1	apla:101800617/apla:101803512/apla:101800942/apla:101793597/apla:101801648/apla:101791037/apla:101795780	7	5	101	101800942/101791037
apla00760	Nicotinate and nicotinamide metabolism	2/452	34/4924	0.832985	0.999967	101803138/101800542	NNT/ENPP3	apla:101803138/apla:101800542	2	2	101803138/101800542	
apla00670	One carbon pool by folate	1/452	19/4924	0.840058	0.999967	101803074	ALDH1L2	apla:101803074	1	1	##	
apla03060	Protein export	1/452	21/4924	0.86818	0.999967	101794894	SEC61B	apla:101794894	1	0		101794894
apla03420	Nucleotide excision repair	2/452	38/4924	0.876273	0.999967	101796914/101797382	LIG1/POLD3	apla:101796914/apla:101797382	2	1	##	101796914
apla00350	Tyrosine metabolism	1/452	22/4924	0.880332	0.999967	101794881	FAHD1	apla:101794881	1	1	##	
apla05164	Influenza A	7/452	110/4924	0.89027	0.999967	101789899/119713491/101800595/101795633/101800900/101791037/101791200	LOC101789899/LOC119713491/RNASEL/RAF1/PIK3CB/MAP2K1/SOCS3	apla:101789899/apla:101799861/apla:101800595/apla:101795633/apla:101800900/apla:101791037/apla:101791200	7	1	##	101789899/119713491/101795633/101800900/101791037/101791200
apla00563	Glycosylphosphatidylinositol (GPI)-anchor biosynthesis	1/452	23/4924	0.891367	0.999967	101792943	PIGK	apla:101792943	1	1	##	
apla00860	Porphyrin metabolism	1/452	23/4924	0.891367	0.999967	101805057	HMOX1	apla:101805057	1	0		101805057
apla00982	Drug metabolism - cytochrome P450	1/452	25/4924	0.910482	0.999967	119713930	LOC119713930	apla:113839739	1	1	##	
apla03020	RNA polymerase	1/452	26/4924	0.918741	0.999967	101792149	POLR2B	apla:101792149	1	1	##	
apla00970	Aminoacyl-tRNA biosynthesis	2/452	45/4924	0.928043	0.999967	119714249/101795091	LOC119714249/TARS3	apla:101800397/apla:101795091	2	2	119714249/101795091	
apla05168	Herpes simplex virus 1 infection	8/452	135/4924	0.938086	0.999967	101798286/101789439/101803666/101800449/119718656/101800595/101800900/101791200	EIF4EBP1/LOC101789439/ITGB3/LOC101800449/LOC119718656/RNASEL/PIK3CB/SOCS3	apla:101798286/apla:101789439/apla:101803666/apla:101800449/apla:101797091/apla:101800595/apla:101800900/apla:101791200	8	3	101	101798286/101803666/119718656/101800900/101791200
apla00983	Drug metabolism - other enzymes	2/452	50/4924	0.951671	0.999967	101803472/119713930	ITPA/LOC119713930	apla:101803472/apla:113839739	2	1	##	101803472
apla00565	Ether lipid metabolism	2/452	55/4924	0.967783	0.999967	101790184/101802263	ENPP6/LPCAT2	apla:101790184/apla:101802263	2	1	##	101802263

apla00591	Linoleic acid metabolism	1/452	36/4924	0.96917	0.999967	101792912	LOC101792912	apla:101792912	1	1	##	
apla04623	Cytosolic DNA-sensing pathway	1/452	37/4924	0.972021	0.999967	101789899	LOC101789899	apla:101789899	1	0		101789899
apla00240	Pyrimidine metabolism	2/452	57/4924	0.972659	0.999967	101804715/101800542	DTYMK/ENPP3	apla:101804715/apla:101800542	2	2	101804715/101800542	
apla04520	Adherens junction	3/452	75/4924	0.973821	0.999967	101791271/101801641/101803609	PTPN6/LOC101801641/PTPRM	apla:101791271/apla:101801641/apla:101803609	3	1	##	101791271/101801641
apla00513	Various types of N-glycan biosynthesis	1/452	40/4924	0.97909	0.999967	119717607	B4GALT2	apla:101792817	1	1	##	
apla04217	Necroptosis	6/452	126/4924	0.979392	0.999967	101799450/101789899/119713491/119717912/101801648/101797377	GLUL/LOC101789899/LOC119713491/LOC119717912/CAMK2D/GLUD1	apla:101799450/apla:101789899/apla:101799861/apla:101798194/apla:101801648/apla:101797377	6	2	119	101799450/101789899/119713491/101797377
apla04144	Endocytosis	12/452	215/4924	0.982249	0.999967	110354206/101803068/101792227/101797658/119713089/101805179/101799120/101801641/101795497/101801705/101796604/101803197	FGFR4/TFRC/CAV3/HSPA2/HSPA5/EEA1/AGAP1/LOC101801641/RAB10/WWP1/EPN3/DNAJC6	apla:110354206/apla:101803068/apla:101792227/apla:101797658/apla:101801738/apla:101805179/apla:101799120/apla:101801641/apla:101795497/apla:101801705/apla:101796604/apla:101803197	12	9	110	101797658/119713089/101801641
apla00510	N-Glycan biosynthesis	1/452	44/4924	0.985823	0.999967	119717607	B4GALT2	apla:101792817	1	1	##	
apla03015	mRNA surveillance pathway	2/452	66/4924	0.987082	0.999967	101798527/101801785	PPP2R2D/LOC101801785	apla:101797307/apla:101801785	2	0		101798527/101801785
apla00600	Sphingolipid metabolism	1/452	48/4924	0.990391	0.999967	101798829	NEU2	apla:101798829	1	1	##	
apla03460	Fanconi anemia pathway	1/452	50/4924	0.99209	0.999967	101790058	BLM	apla:101790058	1	1	##	
apla04340	Hedgehog signaling pathway	1/452	52/4924	0.993489	0.999967	101803152	GPR161	apla:101803152	1	1	##	
apla04110	Cell cycle	4/452	111/4924	0.993511	0.999967	101792368/101791694/101801576/101795780	CCNA2/CDKN1B/E2F1/MAD1L1	apla:101792368/apla:101791694/apla:101801576/apla:101795780	4	2	101	101792368/101791694
apla03250	Viral life cycle - HIV-1	1/452	57/4924	0.995999	0.999967	113840221	MAP1B	apla:101796523	1	1	##	
apla04120	Ubiquitin mediated proteolysis	4/452	130/4924	0.998446	0.999967	101790223/101801705/101803032/101791200	UBE2D3/WWP1/UBE2B/SOCS3	apla:101790223/apla:101801705/apla:101799305/apla:101791200	4	1	##	101790223/101803032/101791200
apla03008	Ribosome biogenesis in eukaryotes	1/452	69/4924	0.99876	0.999967	119713234	LOC119713234	apla:101792624	1	1	##	
apla03018	RNA degradation	1/452	71/4924	0.99898	0.999967	101793601	BTG2	apla:101793601	1	0		101793601
apla03040	Spliceosome	2/452	100/4924	0.999327	0.999967	101797658/119713089	HSPA2/HSPA5	apla:101797658/apla:101801738	2	0		101797658/119713089
apla03013	Nucleocytoplasmic transport	2/452	105/4924	0.999569	0.999967	101793795/101803999	RANGAP1/IPO13	apla:101793795/apla:101803999	2	1	##	101793795
apla03010	Ribosome	1/452	106/4924	0.999967	0.999967	106017870	MRPL2	apla:106017870	1	1	##	
